# Identification of Emergency Care–Sensitive Conditions and Characteristics of Emergency Department Utilization

**DOI:** 10.1001/jamanetworkopen.2019.8642

**Published:** 2019-08-07

**Authors:** Anita A. Vashi, Tracy Urech, Brendan Carr, Liberty Greene, Theodore Warsavage, Renee Hsia, Steven M. Asch

**Affiliations:** 1 Department of Emergency Medicine, Stanford University, Stanford, California; 2 Center for Innovation to Implementation, VA Palo Alto Health Care System, Palo Alto, California; 3 Department of Emergency Medicine, Sidney Kimmel Medical College, Thomas Jefferson University, Philadelphia, Pennsylvania; 4 Division of Primary Care and Population Health, Stanford University, Stanford, California; 5 Denver Center of Innovation for Veteran Centered and Value Driven Care, Rocky Mountain Regional VA Medical Center, Denver, Colorado; 6 Philip R. Lee Institute for Health Policy Studies, Department of Emergency Medicine, University of California, San Francisco

## Abstract

**Importance:**

Monitoring emergency care quality requires understanding which conditions benefit most from timely, quality emergency care.

**Objectives:**

To identify a set of emergency care–sensitive conditions (ECSCs) that are treated in most emergency departments (EDs), are associated with a spectrum of adult age groups, and represent common reasons for seeking emergency care and to provide benchmark national estimates of ECSC acute care utilization.

**Design, Setting, and Participants:**

A modified Delphi method was used to identify ECSCs. In a cross-sectional analysis, ECSC-associated visits by adults (aged ≥18 years) were identified based on *International Statistical Classification of Diseases, Tenth Revision, Clinical Modification* diagnosis codes and analyzed with nationally representative data from the 2016 US Nationwide Emergency Department Sample. Data analysis was conducted from January 2018 to December 2018.

**Main Outcomes and Measures:**

Identification of ECSCs and ECSC-associated ED utilization patterns, length of stay, and charges.

**Results:**

An expert panel rated 51 condition groups as emergency care sensitive. Emergency care–sensitive conditions represented 16 033 359 of 114 323 044 ED visits (14.0%) in 2016. On average, 8 535 261 of 17 886 220 ED admissions (47.7%) were attributed to ECSCs. The most common ECSC ED visits were for sepsis (1 716 004 [10.7%]), chronic obstructive pulmonary disease (1 273 319 [7.9%]), pneumonia (1 263 971 [7.9%]), asthma (970 829 [6.1%]), and heart failure (911 602 [5.7%]) but varied by age group. Median (interquartile range) length of stay for ECSC ED admissions was longer than non-ECSC ED admissions (3.2 [1.7-5.8] days vs 2.7 [1.4-4.9] days; *P* < .001). In 2016, median (interquartile range) ED charges per visit for ECSCs were $2736 ($1684-$4605) compared with $2179 ($1118-$4359) per visit for non-ECSC ED visits (*P* < .001).

**Conclusions and Relevance:**

This comprehensive list of ECSCs can be used to guide indicator development for pre-ED, intra-ED, and post-ED care and overall assessment of the adult, non–mental health, acute care system. Health care utilization and costs among patients with ECSCs are substantial and warrant future study of validation, variations in care, and outcomes associated with ECSCs.

## Key Points

**Question:**

For which conditions is timely access to high-quality emergency care associated with morbidity and mortality, and what are the characteristics of emergency department visits for these conditions?

**Findings:**

In this cross-sectional study, an expert panel identified 51 condition groups as emergency care sensitive. These conditions were associated with substantial emergency department use, inpatient hospitalization, and cost.

**Meaning:**

Emergency care–sensitive conditions may be used in future efforts to measure the quality of emergency care within a systems framework.

## Teaser

This cross-sectional study identifies a set of emergency care–sensitive conditions to provide benchmark US national estimates of acute care utilization for these conditions.

## Introduction

The emergency care system plays a crucial role in the chain of survival for acute conditions such as myocardial infarction,^1^ stroke,^2^ and trauma.^3^ Swift in-hospital diagnosis and prompt treatment are its hallmarks, but over the past 2 decades, emergency care has expanded to encompass a broader continuum of services. These activities range from those that precede the emergency department (ED) visit (eg, prevention and the 911 system) to those that are the consequence of the visit (eg, urgent care pathways for lower-acuity patients and transfers of patients to higher-acuity inpatient settings or back to their usual source of care). The National Academy of Medicine has recommended that the quality of emergency care be examined within a systems framework, ie, how the many components of the system, such as EDs, emergency medical services, acute care and primary care clinicians, and on-call specialists, work together—or frequently fail to work together—to achieve a level of performance for the system as a whole.^4^

Unfortunately, most emergency care quality measurement systems fail to take such a systems perspective. Instead, existing indicators, such as door-to-balloon time in acute myocardial infarction or time to antibiotics in sepsis, narrowly examine aspects of the visit to the ED itself, ignoring other aspects of acute care delivery. For example, with acute myocardial infarction, antecedent ambulance transport time and subsequent catheterization laboratory care and quality may affect care delivery as much as door-to-balloon time. What is glaringly absent from the acute care quality measurement landscape is robust outcome measures, such as morbidity and mortality, that can be associated with acute care quality.

Moreover, in existing quality measure frameworks, ED utilization is often used as a measure of failure of the antecedent primary or specialty care. For example, rates of ED visits for ambulatory care–sensitive conditions (ACSCs) are used to evaluate access to and quality of outpatient primary care.^5^ Similarly, the Agency for Healthcare Research and Quality ED Prevention Quality Indicators provide a window into community health by measuring rates of potentially preventable ED visits; for example, visits for nontraumatic dental pain suggest a lack of adequate community oral health preventive services.^6^

A first step in building a broader, systems-focused emergency care quality monitoring framework is to develop a list of conditions that are most sensitive to timely, quality emergency care across the continuum. We can then use such emergency care–sensitive conditions (ECSCs) to guide indicator development for pre-ED, intra-ED, and post-ED care.^7^ Focused on the acute care system rather than other care settings, these measures may be used to study regional and facility variations in acute care–associated processes and outcomes.^8^ Consistent with recent efforts at the National Quality Forum to identify population-level measures for injury care,^9^ our hope is that ECSC-based measures will encourage health care organizations to adopt a population perspective and inspire innovation at the clinical microsystem level to improve acute, unscheduled care for patients that urgently need it.

This study builds on prior efforts and provides what we believe is the first comprehensive list of ECSCs with explicit diagnosis code inclusions and exclusions that can be measured using readily available hospital administrative data. To better contextualize the burden of ECSCs on the US health care system, we also present national estimates of acute care utilization and demographic characteristics of adults experiencing ECSCs.

## Methods

To define an explicit set of ECSCs, we convened a multidisciplinary expert panel with members from emergency medicine, primary care medicine, and hospitalist medicine to represent the emergency care delivery continuum. Relevant specialty societies nominated potential panelists to participate. The selection process was designed to maintain diversity in geography, clinical expertise, and practice setting among the group (eTable 1 in the Supplement). To determine the national prevalence of ECSCs, we conducted a retrospective study using data from the National Emergency Department Sample (NEDS) database.^10^ We followed the Strengthening the Reporting of Observational Studies in Epidemiology (STROBE) reporting guideline for cross-sectional studies. This study was approved by the Stanford University institutional review board. We were not required to obtain informed consent from the members of the expert panel.

### Defining ECSCs

First described by Carr et al^7^ in 2010, a conceptual model using ECSCs provides a novel framework to study emergency care quality from a systems perspective. The concept for this framework was derived from the development, measurement, and widespread use of ACSCs.^11^ Analogous to ACSCs, ECSCs are “conditions for which rapid diagnosis and early intervention in acute illness or acutely decompensated chronic illness improve patient outcomes.”^7^ However, there is a notable difference between ACSCs and ECSCs. Quality indicators related to ACSCs are designed to measure rates of admission with the underlying assumption that high rates of hospitalization for ACSCs indicate that patients are not receiving high-quality ambulatory care.^12,13^ Conversely, higher rates of hospitalization or ED utilization for ECSCs are not an indicator of poor emergency care. Rather, the idea of ECSCs is that acute illness and acute exacerbations of chronic disease are inevitable, and when they occur, the emergency care system should be able to rapidly identify and treat these episodes in a coordinated and effective way.

### Identifying Candidate Conditions for Expert Panel Review

To identify the candidate conditions to be rated by the panel, the following explicit principles were established for the selection of conditions: (1) they should be treated in most EDs, (2) they should be associated with a spectrum of adult age groups, (3) they should represent common reasons for which patients seek emergency care, and (4) some evidence must exist to suggest that quality clinical care in the ED may impact morbidity or mortality. Conditions for which improving ED care would be unlikely to substantially change patient outcomes were excluded. Diagnosis codes describing mental health conditions were considered out of scope for the current study and not included.

Next, 2 of us (A.A.V. and S.M.A.), an emergency medicine physician and an internist, created an initial list of conditions (ie, alphanumeric parent-level *International Statistical Classification of Diseases, Tenth Revision, Clinical Modification *[*ICD*-*10*-*CM*] codes^14^) that met the preestablished criteria from a review of the emergency care literature^15,16^ (n = 160) and suggestions from panelists (n = 7) prior to the rating process.

The same 2 physicians reviewed all *ICD*-*10*-*CM* diagnosis subcodes (n = 13 937) for the 167 diagnosis groups to ensure they represented conditions where rapid diagnosis and timely emergency care intervention could affect patient outcomes for acute illness and acutely decompensated chronic illness. Subcodes were excluded if they indicated pediatric (eg, G93.7, Reye syndrome), chronic (eg, G03.1, chronic meningitis), or subacute (eg, K63.3, ulcer of intestine) conditions (n = 524). Subcodes strictly describing subsequent encounters or sequelae were also excluded (n = 8714). After exclusions, 4699 subcodes from 167 parent diagnosis groups remained. Subsequently, to present a cohesive list of clinically discrete condition groups to the panelists, the 2 physicians combined diagnosis subcodes that were clinically similar with comparable emergency treatment pathways. This aggregation resulted in 66 condition groups (Figure). Thus, condition groups included subcodes from multiple *ICD*-*10*-*CM* categories. For example, the condition group intracranial hemorrhage included diagnosis subcodes from 3 *ICD*-*10*-*CM* categories: (1) I60, nontraumatic subarachnoid hemorrhage; (2) I61, nontraumatic intracerebral hemorrhage; and (3) I62, other and unspecified nontraumatic intracranial hemorrhage (eAppendix in the Supplement).

**Figure.**
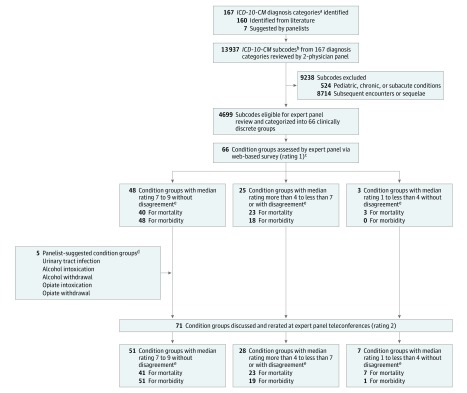
Process of Identifying and Rating Emergency Care–Sensitive Condition Groups Number of condition groups categorized into 3 rating groups do not sum to total condition groups because some were categorized differently for mortality and morbidity (ie, acute appendicitis was rated a median of 6.5 for mortality, putting it in the middle group, and rated a median of 7 for morbidity, putting it in the emergency care–sensitive group). *ICD*-*10*-*CM* indicates *International Statistical Classification of Diseases, Tenth Revision, Clinical Modification*. ^a^Refers to the 3-character label for a disease category (eg, I21, acute myocardial infarction). ^b^Subcodes describe the disease or injury etiology, anatomic site, severity, and encounter type (eg, I21.09: ST-elevation myocardial infarction [STEMI] involving other coronary artery of anterior wall).All included and excluded subcodes rated as highly sensitive to emergency care are listed in the eAppendix in the Supplement. ^c^Panelists were asked the 2 following questions: (1) for the average patient with this condition, to what extent does timely emergency care affect subsequent mortality, and (2) for the average patient with this condition, to what extent does timely emergency care affect subsequent morbidity. Panelists responded with a rating from 1 (no or little impact) to 9 (strong impact). ^d^For the 5 panelist-suggested condition groups, 119 *ICD*-*10*-*CM* subcodes were reviewed. Overall, 30 new subcodes were included, and 89 were excluded because they indicated pediatric, chronic, or subacute conditions. None of the subcodes indicated subsequent encounters or sequelae. ^e^Disagreement was defined as least 3 panelists rating the condition group in the range of 7 to 9 and at least 3 panelists rating the condition group in the range of 1 to less than 4.

### Expert Panel Review to Define an Explicit Set of ECSCs

We used the modified Delphi method to rate each candidate condition group.^17^ This consisted of a formal group process in which an expert panel discussed and iteratively rated the appropriateness of candidate conditions using a 2-round process. This method of selecting quality indicators is reliable and has been shown to have content, construct, and predictive validity.^18,19^

Panelists were oriented to study objectives, relevant literature, and methods during a round 0 meeting. Literature and evidence review was used to compile relevant clinical information regarding incidence, prevalence, morbidity, and mortality rates and key aspects of emergency care management for each candidate condition group.

In January 2016, panelists independently rated the 66 condition groups using an online survey hosted in Qualtrics (rating 1). For each condition group, panelists were provided the clinical information described above and the list of included and excluded *ICD*-*10*-*CM* diagnosis subcodes. Panelists were asked to separately rate to what extent, for the average patient with this condition, timely emergency care affects subsequent mortality and morbidity. The rating scale ranged from 1 to 9, with 1 indicating no or little impact, 5 indicating moderate impact, and 9 indicating strong impact. During this round, panelists could suggest new conditions to be rated in the second round.

Prior to the virtual meeting in February 2016, panelists received personalized rating summary sheets showing their rating 1 scores, the median score, and the distribution of the panelists’ ratings. During 2 half-day meetings, panelists reviewed, discussed, and rerated the 66 conditions groups and rated 5 panelist-suggested conditions (rating 2). A trauma surgeon introduced all surgical and trauma-related conditions and answered panelists’ questions. The ratings were anonymous, each panelist received equal weight, and consensus was not required or achieved. We analyzed the panelists’ rating 2 scores using previously defined operational thresholds as follows: (1) highly sensitive to emergency care (ECSC), defined as a median rating from 7 to 9 without disagreement; (2) intermediate, defined as a median rating from 4 or more to less than 7 or any median with disagreement; and (3) weakly sensitive to emergency care, defined as a median rating from 1 to less than 4 without disagreement. Disagreement was defined as at least 3 panelists rating the condition group in the range of 7 to 9 and at least 3 panelists rating the condition group in the range of 1 to less than 4 (Figure).^17^

### Measuring the Prevalence of ECSCs Nationally

#### Study Design and Data Sources

The NEDS database represents the largest all-payer ED database in the United States and was constructed using records from the State Emergency Department Databases and the State Inpatient Databases.^10^ The State Emergency Department Databases capture information on ED visits that do not result in an admission. The State Inpatient Databases contain information on patients initially seen in the ED and then admitted to the same hospital. These data are collected at the state level and are made publicly available by the Agency for Healthcare Research and Quality. The NEDS contains 25 to 30 million (unweighted) records of ED visits for more than 950 hospitals and approximates a 20% stratified sample of US hospital-based EDs. The NEDS database includes weights for calculating national estimates.

#### Study Population

We analyzed ED visits from 2016 for patients 18 years and older. If an ED visit, whether admitted or discharged, was associated with a primary diagnosis for a condition group rated as highly sensitive to emergency care by the expert panel, then it was classified as an ECSC visit. All other visits were classified as non-ECSC visits. Emergency care–sensitive conditions were defined using the *ICD*-*10*-*CM* inclusion and exclusion subcodes as previously described. Non-ECSCs were classified using primary diagnosis into meaningful categories via the Agency for Healthcare Research and Quality Clinical Classifications Software for *ICD*-*10*-*CM*.^20^

#### Patient-Level and Hospital-Level Variables

Available patient variables included age, sex, insurance status, rurality, and median household income for the patient’s zip code. Agency for Healthcare Research and Quality–defined comorbidities were calculated based on *ICD*-*10*-*CM* codes developed by Elixhauser et al.^21^ These included dichotomous indicators for the presence or absence of 29 comorbidities and were summed to create a comorbidity score. Comorbidities could only be calculated for patients with ED visits that led to admission because comorbidity variables appear in the State Inpatient Databases but not the State Emergency Department Databases.^10^ Encounter variables included date of ED visit, patient disposition from the ED, length of stay for admissions, and total ED and hospital charges. No charge and ED charges that were excessively low or high were considered inconsistent by NEDS in approximately 14% of ED visits and considered missing in this study. Consistent with previous studies, missing charge values were treated as missing at random and estimated based on a regression model using known variables.^22,23^ Hospital characteristics included variables for region, trauma center indicator, urban or rural classification, teaching status, and ownership.

### Statistical Analysis

We used data from 2016 to describe and compare (1) the proportion of ED visits associated with ECSCs; (2) disposition from the ED; (3) length of hospital stay; and (4) total charges. Proportions, weighted frequencies, and medians were computed from a weighted analysis accounting for the NEDS sampling design.^24^ *P *values were calculated using χ^2^ tests to compare proportions and an extension of Mood median test to compare medians across the groups.^25^ Median tests were 2-sided, and *P* < .05 was considered statistically significant. All analyses were done using SAS version 9.4 (SAS Institute).

## Results

Our expert panel rated 51 of 71 condition groups (72%) as ECSCs: 41 conditions (80%) for morbidity and mortality, and 10 (20%) conditions for morbidity only (Table 1). Of the 114 323 044 estimated ED visits in 2016, there were 16 033 359 estimated ED visits for ECSCs meeting our inclusion criteria, representing 14.0% of all ED visits. Characteristics of these visits are presented in Table 2. Emergency care–sensitive conditions accounted for 6 544 983 of 94 286 898 ED treat-and-release visits (6.9%) and 8 535 261 of 17 886 220 ED admissions (47.7%). On average, 8 535 261 ECSC ED visits (53.2%) resulted in hospital admission, and 164 603 patients (1.0%) died in the ED. Emergency care–sensitive condition ED visits were more common among adults 65 years and older, accounting for 26.2% (7 098 827 of 27 133 915) of all ED visits in this group. Unadjusted proportions of ECSC ED visits compared with non-ECSC ED visits were higher for certain groups, such as men (7 864 028 [49.0%] vs 41 051 794 [41.8%]), those with 5 or more comorbid conditions (2 384 637 [14.9%] vs 1 885 457 [1.9%]), those living in nonmetropolitan counties (3 102 904 [19.4%] vs 17 223 895 [17.5%]), those living in the highest–income quartile areas (2 820 483 [17.6%] vs 15 764 155 [16.0%]), and those with Medicare insurance coverage (7 861 578 [49.0%] vs 25 329 833 [25.8%]). Unadjusted proportions of ECSC ED visits were similar across regions, hospital locations, types of hospital ownership, trauma center statuses, and teaching affiliations.

**Table 1.** Final Panel Ratings for Mortality and Morbidity of the 71 Condition Groups

Condition Group	Panel Rating, Median (IQR)	
	Mortality	Morbidity
Rated as highly sensitive to emergency care for both mortality and morbidity (n = 41)^a^		
Abdominal, lower back, pelvic, and external genitalia injuries	8 (8-9)	9 (8-9)
Acute respiratory distress syndrome	7 (6-8)	7 (6.5-8)
Alcohol withdrawal	7 (5.5-8)	7 (6-8)
Anaphylaxis	9 (8.5-9)	8 (7.5-9)
Angina and other acute ischemic heart diseases	7 (7-8)	8 (7-8)
Aortic aneurysm and dissection	9 (8-9)	8.5 (8-9)
Arterial embolism and thrombosis	8 (7-9)	8 (8-9)
Asthma	7 (6-8)	8 (7-8)
Cardiac arrest and severe arrhythmias	9 (9-9)	9 (8.5-9)
Cerebral infarction	7.5 (7-8.5)	9 (9-9)
Chronic obstructive pulmonary disease	7.5 (6.5-8)	8 (8-8.5)
Complications of cardiac and vascular prosthetic devices or grafts	7 (6-7)	7 (6.5-7)
Complications of procedures	7 (6-7)	7 (6-7.5)
Acute diabetes	8.5 (7.5-9)	7.5 (7-9)
Early complications of trauma	8 (7-8)	8 (7.5-8.5)
Ectopic pregnancy	7 (6.5-8)	7 (7-8.5)
Encephalitis, myelitis, and encephalomyelitis	7 (6.5-8)	8 (7-8)
Environmental exposures	8.5 (7-9)	8 (6.5-9)
Gastrointestinal tract bleeding and/or perforation	8.5 (7.5-9)	7 (7-9)
Heart failure	7 (6.5-7.5)	8 (7.5-9)
Infectious fasciitis	9 (9-9)	9 (9-9)
Intracranial hemorrhage	8 (7-8.5)	8 (7.5-9)
Intracranial injury	7 (7-7.5)	8 (7-8.5)
Meningitis	8.5 (8-9)	9 (8.5-9)
Moderate to severe burns and corrosions	8 (8-8.5)	8 (7-8.5)
Myocardial infarction	9 (8.5-9)	9 (9-9)
Neck injuries	7 (7-8)	8 (7.5-8.5)
Other diseases of the intestine	7.5 (7-8.5)	7.5 (7-8.5)
Overdose or poisoning	7.5 (6.5-9)	7 (7-8)
Paralytic ileus and intestinal obstruction without hernia	7 (7-7.5)	7 (7-7)
Pericardial disease, endocarditis, and myocarditis	8 (8-8.5)	8 (7.5-8.5)
Peritonitis	8 (7-8.5)	8 (6.5-8)
Pneumonia	7 (6-8)	7 (7-8.5)
Pneumothorax	8.5 (7-9)	8.5 (7-9)
Postpartum hemorrhage	8 (6.5-8.5)	7 (6-8)
Preeclampsia or eclampsia	7 (6.5-8.5)	8 (7-8.5)
Pulmonary embolism	9 (8-9)	7.5 (7-9)
Respiratory failure	9 (8-9)	8 (7-9)
Sepsis and SIRS	9 (9-9)	9 (8.5-9)
Shock	9 (9-9)	9 (8.5-9)
Thoracic Injuries	8 (8-9)	8.5 (8-9)
Rated as highly sensitive to emergency care for morbidity only (n = 10)^b^		
Acute angle closure glaucoma	1 (1-1.5)	9 (7.5-9)
Acute appendicitis	6.5 (5.5-7.5)	7 (6-8)
Acute pancreatitis	6 (5.5-7)	7.5 (7-8)
Cholecystitis and perforation of the gallbladder	6 (5-7)	7 (6-7.5)
Disorders of the brain	6 (6-7)	7.5 (7-8)
Femur fracture	6 (5.5-7)	8 (7-8)
Other cardiac arrhythmia	6 (5.5-7)	7 (6.5-7.5)
Other tachyarrhythmias	6 (5-6)	7 (6-7.5)
Septic arthritis	6.5 (5.5-7)	7 (7-8.5)
Volume depletion	6 (6-7)	7 (7-8.5)
All other condition groups (n = 20)		
Acute kidney failure	5 (5-6)	6.5 (6-8)
Adult abuse, neglect, or maltreatment	4.5 (3-6)	6 (5-7)
Alcohol intoxication	3 (3-4)	4.5 (4-6)
Bleeding in early pregnancy	4 (3.5-6)	6 (6-6.5)
Cellulitis and lymphangitis	4.5 (3-5.5)	5.5 (5-6.5)
Delirium	3.5 (3-6)	6 (5.5-7)
Subacute diabetes	4.5 (3-5.5)	5.5 (4.5-6.5)
Disorders of fluid, electrolyte, and acid-base	6 (5-6.5)	6 (5.5-6)
Disseminated intravascular coagulation	6 (6-7)	6 (6-6.5)
Diverticulitis	5 (4-6)	6 (5.5-6.5)
Hepatic failure (acute and chronic)	5 (4.5-5)	5 (5-5)
Intestinal infectious diseases	5 (3.5-5)	6 (5-6)
Malignant neoplasm of brain	5 (5-5.5)	6 (5-7)
Mild burns and corrosions	2.5 (1.5-3)	3.5 (3-4)
Opiate intoxication	6.5 (4-7.5)	6.5 (4.5-7)
Opiate withdrawal	2.5 (1.5-4)	5.5 (4.5-6)
Pleural effusion	5 (4-6)	5.5 (5-6)
Pneumonitis and other interstitial pulmonary diseases	6 (5-7)	6 (5-6)
Renal calculus and colic	3 (2-3)	6.5 (5.5-7.5)
Urinary tract infections	2 (2-3.5)	5 (4.5-5.5)

Abbreviations: IQR, interquartile range; SIRS, systemic inflammatory response syndrome.

^a^ Condition groups had a panel median rating from 7 to 9 without disagreement for mortality and morbidity questions.

^b^ Condition groups had a panel median rating from 7 to 9 without disagreement for morbidity question only.

**Table 2.** Sociodemographic and Visit Characteristics of Patients Presenting to US EDs in 2016^a^

Characteristic	ED Visits, No. (%)			*P* Value
	Total (N = 114 323 044)	ECSC (n = 16 033 359)	Non-ECSC (n = 98 289 685)	
Age, y				
18-44	53 349 370 (46.7)	3 809 536 (23.8)	49 539 834 (50.4)	<.001
45-64	33 839 758 (29.6)	5 124 995 (32.0)	28 714 763 (29.2)	
≥65	27 133 915 (23.7)	7 098 827 (44.3)	20 035 088 (20.4)	
Sex				
Male	48 915 822 (42.8)	7 864 028 (49.0)	41 051 794 (41.8)	<.001
Female	65 387 451 (57.2)	8 164 134 (50.9)	57 223 317 (58.2)	
Missing	19 771 (<0.1)	5197 (<0.1)	14 574 (<0.1)	
Comorbid conditions, No.				
0	1 709 339 (1.5)	530 779 (3.3)	1 178 560 (1.2)	<.001
1-2	5 884 563 (5.1)	2 609 244 (16.3)	3 275 319 (3.3)	
3-4	6 022 224 (5.3)	3 010 601 (18.8)	3 011 623 (3.1)	
≥5	4 270 094 (3.7)	2 384 637 (14.9)	1 885 457 (1.9)	
Unknown^b^	96 436 823 (84.4)	7 498 098 (46.8)	88 938 726 (90.5)	
Disposition following ED visit				
Treated and released	94 286 898 (82.5)	6 544 983 (40.8)	87 741 915 (89.3)	<.001
Admitted	17 886 220 (15.6)	8 535 261 (53.2)	9 350 959 (9.5)	
Transferred	1 960 230 (1.7)	788 512 (4.9)	1 171 719 (1.2)	
Died in the ED	189 695 (0.2)	164 603 (1.0)	25 092 (<0.1)	
Patient residence				
Metropolitan counties	93 325 990 (81.6)	12 842 382 (80.1)	80 483 608 (81.9)	<.001
Nonmetropolitan counties	20 326 799 (17.8)	3 102 904 (19.4)	17 223 895 (17.5)	
Missing	670 255 (0.6)	88 073 (0.5)	582 182 (0.6)	
Median household income percentile				
≤25th	39 282 465 (34.4)	5 186 780 (32.3)	34 095 685 (34.7)	<.001
26th-50th	30 718 519 (26.9)	4 288 323 (26.7)	26 430 196 (26.9)	
51st-75th	23 543 105 (20.6)	3 428 741 (21.4)	20 114 364 (20.5)	
≥76th	18 584 638 (16.3)	2 820 483 (17.6)	15 764 155 (16.0)	
Missing	2 194 317 (1.9)	309 032 (1.9)	1 885 285 (1.9)	
Insurance				
Private	32 620 995 (28.5)	3 666 167 (22.9)	28 954 828 (29.5)	<.001
Medicare	33 191 411 (29.0)	7 861 578 (49.0)	25 329 833 (25.8)	
Medicaid	28 165 562 (24.6)	2 698 677 (16.8)	25 466 885 (25.9)	
Uninsured^c^	15 102 230 (13.2)	1 254 140 (7.8)	13 848 090 (14.1)	
Other^d^	5 093 674 (4.5)	537 679 (3.4)	4 555 995 (4.6)	
Missing	149 173 (0.1)	15 119 (0.1)	134 054 (0.1)	
Hospital region				
Northeast	21 067 969 (18.4)	2 871 038 (17.9)	18 196 931 (18.5)	.04
Midwest	26 170 557 (22.9)	3 738 489 (23.3)	22 432 068 (22.8)	
South	45 520 172 (39.8)	6 289 625 (39.2)	39 230 547 (39.9)	
West	21 564 347 (18.9)	3 134 208 (19.5)	18 430 139 (18.8)	
Trauma center designation				
Trauma center	49 355 447 (43.2)	7 208 750 (45.0)	42 146 697 (42.9)	<.001
Nontrauma center	63 754 236 (55.8)	8 660 345 (54.0)	55 093 891 (56.1)	
Not classified	1 213 361 (1.1)	164 264 (1.0)	1 049 097 (1.1)	
Hospital location				
Metropolitan	96 527 992 (84.4)	13 523 365 (84.3)	83 004 627 (84.4)	.47
Micropolitan	9 621 132 (8.4)	1 347 523 (8.4)	8 273 609 (8.4)	
Nonurban	5 661 456 (5.0)	774 746 (4.8)	4 886 710 (5.0)	
Not classified	2 512 464 (2.2)	387 725 (2.4)	2 124 739 (2.2)	
Hospital teaching status				
Teaching	51 699 798 (45.2)	7 269 813 (45.3)	44 429 985 (45.2)	.77
Nonteaching	62 623 246 (54.8)	8 763 546 (54.7)	53 859 700 (54.8)	
Hospital ownership				
Private	27 892 757 (24.4)	3 924 071 (24.5)	23 968 686 (24.4)	.10
Public	5 779 626 (5.1)	761 243 (4.7)	5 018 383 (5.1)	
Not classified	80 650 660 (70.5)	11 348 045 (70.8)	69 302 615 (70.5)	

Abbreviations: ECSC, emergency care–sensitive condition; ED, emergency department.

^a^ Based on the Nationwide Emergency Department Sample. Numbers of visits and percentages are based on weighted population estimates. Percentages may not add to 100% because of rounding.

^b^ Number of comorbid conditions only available for the ED visits that led to admission.

^c^ Includes self-pay and no-charge ED visits.

^d^ Includes worker’s compensation, Civilian Health and Medical Program of the Uniformed Services, Civilian Health and Medical Program of the Department of Veterans Affairs, Title V, and other government programs.

### Diagnoses

The most common ECSC ED visits were for sepsis or systematic inflammatory response syndrome (SIRS) (1 716 004 [10.7%]), chronic obstructive pulmonary disease (1 273 319 [7.9%]), pneumonia (1 263 971 [7.9%]), asthma (970 829 [6.1%]), and heart failure (911 602 [5.7%]) (Table 3) but varied by age group (Table 4). Overall, 3 conditions (sepsis or SIRS, pneumonia, and acute diabetes) ranked in the top 10 ECSCs for each age category. The 25 most frequent ECSCs accounted for 91.8% of all ECSC ED visits. Emergency care–sensitive conditions with highest rates of admission included sepsis or SIRS (1 626 911 [94.8%]), respiratory failure (360 744 [88.1%]), and encephalitis, myelitis, and encephalomyelitis (2415 [84.7%]). The ECSCs resulting in the highest rates of death included cardiac arrest and severe arrhythmias (136 498 [62.6%]), shock (446 [8.2%]), and aortic aneurysm and dissection (1056 [2.8%]). Among non-ECSC ED visits, 17 of the 285 Clinical Classifications Software categories accounted for 51.2% of visits, with abdominal pain, nonspecific chest pain, and sprains and strains being the most common (eTable 2 in the Supplement).

**Table 3.** Characteristics of ED Visits by ECSC Condition Group in 2016^a^

ECSC Condition Group	No. (%)					ED Charges, Median (IQR), $
	ECSC ED Visits^b^	Disposition Following ECSC ED Visit				
		Treated and Released^c^	Admitted^c^	Transferred^c^	Died in the ED^c^	
Abdominal, lower back, pelvic, and external genitalia injuries	373 947 (2.3)	220 715 (59.0)	129 644 (34.7)	22 849 (6.1)	740 (0.2)	3014 (1628-6727)
Acute angle closure glaucoma	3053 (<0.1)	2483 (81.3)	181 (5.9)	386 (12.7)	3 (0.1)	2527 (1302-4331)
Acute appendicitis	248 869 (1.6)	125 307 (50.4)	112 888 (45.4)	10 670 (4.3)	4 (<0.1)	14 453 (2771-28 084)
Acute diabetes	905 050 (5.6)	581 138 (64.2)	308 874 (34.1)	14 904 (1.6)	135 (<0.1)	2729 (1656-4356)
Acute pancreatitis	368 124 (2.3)	106 950 (29.1)	245 255 (66.6)	15 891 (4.3)	27 (<0.1)	2866 (1776-4892)
Acute respiratory distress syndrome	8211 (0.1)	4013 (48.9)	1788 (21.8)	2186 (26.6)	224 (2.7)	4740 (2695-8672)
Alcohol withdrawal	263 879 (1.6)	92 554 (35.1)	167 433 (63.5)	3882 (1.5)	10 (<0.1)	2343 (1473-3788)
Anaphylaxis	32 067 (0.2)	28 405 (88.6)	3159 (9.9)	484 (1.5)	20 (0.1)	2574 (1569-4086)
Angina and other acute ischemic heart diseases	117 448 (0.7)	58 229 (49.6)	27 602 (23.5)	31 501 (26.8)	117 (0.1)	4040 (2421-8335)
Aortic aneurysm and dissection	37 453 (0.2)	11 896 (31.8)	16 806 (44.9)	7695 (20.5)	1056 (2.8)	5056 (2500-10 309)
Arterial embolism and thrombosis	20 656 (0.1)	2961 (14.3)	14 595 (70.7)	3062 (14.8)	37 (0.2)	2644 (1688-4256)
Asthma	970 829 (6.1)	871 507 (89.8)	94 767 (9.8)	4495 (0.5)	59 (<0.1)	2157 (1314-3615)
Cardiac arrest and severe arrhythmias	218 200 (1.4)	19 591 (9.0)	49 590 (22.7)	12 521 (5.7)	136 498 (62.6)	3675 (2203-6319)
Cerebral infarction	530 654 (3.3)	53 861 (10.1)	424 584 (80.0)	51 860 (9.8)	350 (0.1)	2547 (1746-4218)
Cholecystitis and perforation of the gallbladder	70 112 (0.4)	22 079 (31.5)	40 913 (58.4)	7101 (10.1)	19 (<0.1)	3511 (2007-7729)
Chronic obstructive pulmonary disease	1 273 319 (7.9)	700 622 (55.0)	541 924 (42.6)	30 394 (2.4)	378 (<0.1)	2837 (1761-4770)
Complications of cardiac and vascular prosthetic devices or grafts	178 592 (1.1)	82 702 (46.3)	91 648 (51.3)	4163 (2.3)	79 (<0.1)	2270 (1342-3788)
Complications of procedures	222 559 (1.4)	118 049 (53.0)	97 495 (43.8)	6993 (3.1)	23 (<0.1)	1857 (992-3370)
Disorders of the brain	149 567 (0.9)	46 078 (30.8)	95 691 (64.0)	7619 (5.1)	179 (0.1)	3226 (1910-8211)
Early complications of trauma	18 971 (0.1)	4965 (26.2)	12 437 (65.6)	1200 (6.3)	370 (2.0)	2390 (1477-4143)
Ectopic pregnancy	51 426 (0.3)	39 301 (76.4)	10 400 (20.2)	1725 (3.4)	0	5494 (2524-20 247)
Encephalitis, myelitis, and encephalomyelitis	2852 (<0.1)	294 (10.3)	2415 (84.7)	144 (5.0)	0	2505 (1656-4051)
Environmental exposures	51 298 (0.3)	44 951 (87.6)	5389 (10.5)	842 (1.6)	115 (0.2)	3022 (1872-4891)
Femur fracture	380 893 (2.4)	25 975 (6.8)	319 461 (83.9)	35 320 (9.3)	137 (<0.1)	2348 (1600-3440)
Gastrointestinal tract bleeding and/or perforation	588 644 (3.7)	187 999 (31.9)	359 739 (61.1)	39 941 (6.8)	965 (0.2)	2608 (1669-4420)
Heart failure	911 602 (5.7)	213 432 (23.4)	655 739 (71.9)	41 750 (4.6)	682 (0.1)	2531 (1678-3884)
Infectious fasciitis	3851 (<0.1)	455 (11.8)	2949 (76.6)	443 (11.5)	4 (0.1)	2420 (1508-3928)
Intracranial hemorrhage	147 558 (0.9)	15 971 (10.8)	91 402 (61.9)	38 218 (25.9)	1967 (1.3)	3786 (2287-6607)
Intracranial injury	543 244 (3.4)	344 513 (63.4)	162 061 (29.8)	35 201 (6.5)	1468 (0.3)	3933 (2236-7246)
Meningitis	11 257 (0.1)	1629 (14.5)	8977 (79.7)	651 (5.8)	0	3199 (1878-5236)
Moderate to severe burns and corrosions	181 531 (1.1)	157 515 (86.8)	14 473 (8.0)	9485 (5.2)	58 (<0.1)	1403 (842-2317)
Myocardial infarction	595 744 (3.7)	34 578 (5.8)	469 469 (78.8)	87 557 (14.7)	4140 (0.7)	2629 (1800-4013)
Neck injuries	103 233 (0.6)	54 161 (52.5)	36 232 (35.1)	12 584 (12.2)	256 (0.2)	3780 (2089-7611)
Other cardiac arrhythmia	106 673 (0.7)	25 340 (23.8)	75 306 (70.6)	5432 (5.1)	595 (0.6)	2536 (1704-3991)
Other diseases of intestine	15 267 (0.1)	2602 (17.0)	10 045 (65.8)	2519 (16.5)	101 (0.7)	3189 (1893-6270)
Other tachyarrhythmias	726 569 (4.5)	369 745 (50.9)	326 473 (44.9)	30 055 (4.1)	297 (<0.1)	3249 (2021-5596)
Overdose or poisoning	593 963 (3.7)	385 895 (65.0)	190 777 (32.1)	16 584 (2.8)	706 (0.1)	2653 (1517-4519)
Paralytic ileus and intestinal obstruction without hernia	386 231 (2.4)	82 264 (21.3)	278 174 (72.0)	25 658 (6.6)	135 (<0.1)	2596 (1635-4531)
Pericardial disease, endocarditis, and myocarditis	54 066 (0.3)	17 850 (33.0)	33 650 (62.2)	2478 (4.6)	88 (0.2)	2941 (1838-5348)
Peritonitis	25 382 (0.2)	6311 (24.9)	16 993 (66.9)	2048 (8.1)	29 (0.1)	2875 (1688-5438)
Pneumonia	1 263 971 (7.9)	606 401 (48.0)	599 440 (47.4)	57 211 (4.5)	920 (0.1)	2628 (1617-4412)
Pneumothorax	33 914 (0.2)	8533 (25.2)	22 082 (65.1)	3268 (9.6)	31 (0.1)	2989 (1827-5078)
Postpartum hemorrhage	18 072 (0.1)	14 983 (82.9)	2661 (14.7)	428 (2.4)	0	3125 (1732-5949)
Preeclampsia or eclampsia	23 090 (0.1)	4204 (18.2)	18 010 (78.0)	872 (3.8)	5 (<0.1)	1731 (1303-3198)
Pulmonary embolism	192 954 (1.2)	31 547 (16.3)	150 568 (78.0)	10 427 (5.4)	412 (0.2)	2542 (1675-4344)
Respiratory failure	409 514 (2.6)	27 214 (6.6)	360 744 (88.1)	17 113 (4.2)	4443 (1.1)	2582 (1704-4154)
Sepsis and SIRS	1 716 004 (10.7)	46 237 (2.7)	1 626 911 (94.8)	39 123 (2.3)	3732 (0.2)	2531 (1679-3775)
Septic arthritis	19 523 (0.1)	2910 (14.9)	15 497 (79.4)	1116 (5.7)	0	2349 (1476-3573)
Shock	5455 (<0.1)	482 (8.8)	3607 (66.1)	920 (16.9)	446 (8.2)	3690 (2122-6653)
Thoracic injuries	348 238 (2.2)	214 656 (61.6)	111 727 (32.1)	19 433 (5.6)	2423 (0.7)	3223 (1744-7488)
Volume depletion	509 780 (3.2)	422 928 (83.0)	76 617 (15.0)	10 109 (2.0)	125 (<0.1)	3418 (2081-5876)

Abbreviations: ECSC, emergency care–sensitive condition; ED, emergency department; IQR, interquartile range; SIRS, systemic inflammatory response syndrome.

^a^ Based on the Nationwide Emergency Department Sample. Numbers of visits and percentages are based on weighted population estimates. Percentages may not add to 100% because of rounding.

^b^ Percentages based on the total number of ECSC ED visits (n = 16 033 359).

^c^ Percentages based on number of ECSC ED visits for condition group.

**Table 4.** Prevalence of the Most Common Conditions for ECSC Emergency Department Visits by Age Group

Rank	Age 18-44 y (n = 3 809 536)		Age 45-64 y (n = 5 124 995)		Age ≥65 y (n = 7 098 827)	
	ECSC Condition Group	No. (%)	ECSC Condition Group	No. (%)	ECSC Condition Group	No. (%)
1	Asthma	585 071 (15.4)	Chronic obstructive pulmonary disease	562 577 (11.0)	Sepsis and SIRS	983 423 (13.9)
2	Overdose or poisonings	356 632 (9.4)	Sepsis and SIRS	504 463 (9.8)	Chronic obstructive pulmonary disease	659 355 (9.3)
3	Acute diabetes	293 807 (7.7)	Pneumonia	386 492 (7.5)	Heart failure	638 304 (9.0)
4	Pneumonia	282 830 (7.4)	Acute diabetes	360 606 (7.0)	Pneumonia	594 649 (8.4)
5	Intracranial injury	238 679 (6.3)	Asthma	295 256 (5.8)	Other tachyarrhythmias	438 407 (6.2)
6	Sepsis and SIRS	228 117 (6.0)	Heart failure	230 586 (4.5)	Cerebral infarction	350 115 (4.9)
7	Volume depletion	164 854 (4.3)	Myocardial infarction	227 607 (4.4)	Myocardial infarction	334 626 (4.7)
8	Acute appendicitis	148 243 (3.9)	Other tachyarrhythmias	224 313 (4.4)	Gastrointestinal tract bleeding and/or perforation	310 374 (4.4)
9	Acute pancreatitis	136 213 (3.6)	Overdose or poisonings	176 195 (3.4)	Femur fracture	308 712 (4.3)
10	Abdominal, lower back, pelvic, and external genitalia injuries	135 333 (3.6)	Gastrointestinal tract bleeding and/or perforation	169 032 (3.3)	Acute diabetes	250 637 (3.5)

Abbreviations: ECSC, emergency care–sensitive condition; SIRS, systemic inflammatory response syndrome.

### Length of Stay and Charges

Median (interquartile range) length of stay for ECSC ED admissions was longer compared with non-ECSC ED admissions (3.2 [1.7-5.8] days vs 2.7 [1.4-4.9] days; *P* < .001). In 2016, median (interquartile range) ED charges per visit for ECSC were $2736 ($1684-$4605) compared with $2179 ($1118-$4359) per visit for non-ECSC ED visits (*P* < .001). The ECSCs with the highest median (interquartile range) ED charges in 2016 were acute appendicitis ($14 453 [$2771-$28 084]), ectopic pregnancy ($5494 [$2524-$20 247]), and aortic aneurysm and dissection ($5056 [$2500-$10 309]).

## Discussion

Using a rigorous modified Delphi process and expert panel review, we identified 51 condition groups most sensitive to emergency care, conditions where timely, high-quality emergency care is expected to make an impact on mortality and morbidity. In 2016, we found nearly 16 million ECSC ED visits and more than 8 million ECSC hospitalizations using the largest representative national sample of US ED visits. Patients who went to EDs with ECSCs and were admitted to the hospital had a median length of stay of 3.2 days and a median ED charge of $2736, compared with 2.7 days and $2179 for patients with non-ECSCs. Developing an acute care quality measurement framework around these ECSCs may encourage population-level measurement and incentivize collaboration across systems and within communities as supported by the National Quality Forum.^26^

We found that most ED visits (86.0%) were not ECSC related. While these visits are more likely to represent conditions for which emergency care is less likely to affect patient outcomes, it is crucial to recognize that non-ECSC visits are not synonymous with unnecessary or inappropriate ED visits and include diagnoses such as lacerations and cellulitis. In fact, evidence suggests that most ED visits are medically necessary and that EDs serve as a critical source of care for high-risk patients, especially for those with comorbid mental health conditions, substance use disorders, and poor social determinants of health.^27^ Instead of penalizing patients, physicians, or hospitals when a condition turns out to be less urgent, emphasis should be placed on integrating care across sites; improving access to primary care, behavioral health, and community-based resources; ensuring availability of affordable medicines; providing clear and consistent information; and identifying the supports required to keep patients healthy and optimize the use of health care.^27,28^

In recent years, use of non-ED acute care venues (eg, urgent care centers, retail clinics, and telemedicine) is increasing rapidly for nonurgent conditions.^29^ Future research could assess the diagnostic and therapeutic resources required to care for patients with selected non-ECSC conditions and may help influence future organization and delivery of acute care. Even some of the 41% of ECSC visits that resulted in treatment and discharge from the ED might have potentially been served in alternate settings. Future analysis could examine variations in ECSC admit-to-discharge ratios to identify emerging best practices at high-performing emergency care systems.

While the proportion of ECSC visits was notably higher in older adults and those with Medicare coverage, variation among other socioeconomic, hospital, and regional factors was minimal. This finding suggests that indicators based on ECSCs may need less adjustment when developed but warrants further study. Understandably, hospital admission rates vary across ECSCs. Future analysis should study variations in condition-specific admission rates, resource use, and outcomes across hospitals using more nuanced geographies that reflect local patterns in health system utilization. Using new methods that can define emergency care service regions, policy makers and regulators could evaluate regional patterns and performance for ECSCs.^8^ Ultimately, benchmarking regional outcomes could encourage competition, or cooperative competition, between traditionally unaligned stakeholders to coordinate on improving population-based outcomes.

We anticipate this comprehensive list of ECSCs will be useful in developing emergency care quality measures. Next, we can use traditional quality indicator development methods to select and determine specifications for potential process and outcome measures. Potential process measures could assess prehospital treatment, airway intervention, imaging, early transfer to the operating room, specialist consultation, and medication administration. Example outcome measures include those associated with morbidity (eg, intensive care unit days, procedural complications, and length of stay), mortality (eg, ED mortality and inpatient mortality), and posthospital events (eg, 30-day mortality and readmission). This new generation of ECSC-based measures could help spur and evaluate organizational innovations in emergency care.

### Limitations

To our knowledge, our evidence-based, expert panel–derived list of ECSCs is the most comprehensive list created to date. It builds on previous Canadian work, expanding the scope of conditions considered and providing detailed diagnostic codes.^15,16^ Nonetheless, we acknowledge that this is early work and this list of ECSCs may need further validation and refinement. Our study has important limitations that must be considered. First, there may be limited concordance between presenting concerns and ED final diagnoses. For that reason, we plan to map ECSC diagnoses to presenting concerns, but there will likely be considerable overlap with non-ECSCs. This will appropriately limit the use of the ECSCs for post hoc reimbursement decisions. Second, we purposefully did not include any pediatric or mental health conditions for panelist consideration because they would require additional expertise. A future panel will consider these conditions. Third, although the modified Delphi method is well known to have predictive validity, the results depend on the panel composition.^18,30^ While it is conceivable that the same process with different panelists would have produced different results, it is likely that the ECSCs selected would be similar. Fourth, cost data reported in this study are limited to ED charges, a likely overestimate that results from limitations of the NEDS data set. Fifth, while this study emphasizes outcomes like morbidity and mortality, we recognize that other valuable ED outcomes (eg, addressing uncertainty and providing reassurance, developing broad differentials, and ruling out life-threatening conditions) exist and should be considered.

## Conclusions

This study produced a comprehensive and detailed list of expert panel–rated ECSCs that could be used to evaluate the quality of acute care systems. Additionally, it presented national estimates of ED presentation for ECSCs across the United States. Often life threatening, ECSCs require high-functioning and coordinated systems in which rapid diagnoses, timely interventions, and seamless transitions are paramount. While measuring care across this acute care continuum is undoubtedly complex, our hope is that such systems thinking will encourage collaboration and accountability between specialties, hospitals, and health care systems. Ideally, this would encourage delivery systems to bridge traditional barriers and share infrastructure to build integrated networks of coordinated emergency care that efficiently deliver time-critical care to the patients who need it most.

## Notes

Supplement. **eAppendix. **Included and Excluded *ICD*-*10*-*CM* Diagnosis Subcodes for Each of the 51 ECSC Groups **eTable 1. **Characteristics of the Panelists **eTable 2. **Most Common Conditions Among Non-ECSC Visits, 2016
