# Supplementary material for: Identification of Emergency Care–Sensitive Conditions and Characteristics of Emergency Department Utilization
Source: JAMA Netw Open. 2019 Aug 7;2(8):e198642. doi: 10.1001/jamanetworkopen.2019.8642 (PMC6686774; doi:10.1001/jamanetworkopen.2019.8642)
Supplement: Supplement. — eAppendix. Included and Excluded ICD-10-CM Diagnosis Subcodes for Each of the 51 ECSC Groups eTable 1. Characteristics of the Panelists eTable 2. Most Common Conditions Among Non-ECSC Visits, 2016 [file jamanetwopen-2-e198642-s001.pdf]

# Supplementary Online Content

Vashi AA, Urech T, Carr B, et al. Identification of emergency care–sensitive conditions and characteristics of emergency department utilization. *JAMA Netw Open*. 2019;2(8):e198642. doi:10.1001/jamanetworkopen.2019.8642

**eAppendix.** Included and Excluded *ICD-10-CM* Diagnosis Subcodes for Each of the 51 ECSC Groups

**eTable 1.** Characteristics of the Panelists

**eTable 2.** Most Common Conditions Among Non-ECSC Visits, 2016

This supplementary material has been provided by the authors to give readers additional information about their work.

© 2019 Vashi AA et al. *JAMA Network Open.*

**eAppendix.** Included and Excluded *ICD-10-CM* Diagnosis Subcodes for Each of the 51 ECSC Groups

Number of Included and Excluded *ICD-10-CM* Diagnosis Subcodes for the 51 ECSC Groups

| | No. of ICD-10-CM diagnosis subcodes | | |
|---|---|---|---|
| **Condition Group** | **Included** | **Excluded[a]** | **Excluded - Other[b]** |
| Abdominal, lower back, pelvic and external genitalia injuries | 763 | 3 | 1532 |
| Acute Angle Closure Glaucoma | 4 | 229 | 0 |
| Acute appendicitis | 4 | 0 | 0 |
| Acute pancreatitis | 6 | 0 | 0 |
| Acute respiratory distress syndrome | 1 | 0 | 0 |
| Alcohol withdrawal | 4 | 1 | 0 |
| Anaphylaxis | 11 | 6 | 34 |
| Angina and other acute ischemic heart diseases | 8 | 0 | 0 |
| Aortic aneurysm and dissection | 12 | 0 | 0 |
| Arterial embolism and thrombosis | 12 | 0 | 0 |
| Asthma | 15 | 3 | 0 |
| Cardiac arrest and severe arrhythmias | 6 | 0 | 0 |
| Cerebral infarction | 72 | 0 | 0 |
| Cholecystitis and perforation of the gallbladder | 4 | 7 | 0 |
| Chronic Obstructive Pulmonary Disease | 3 | 0 | 0 |
| Complications of cardiac and vascular prosthetic devices/grafts | 95 | 2 | 194 |
| Complications of procedures | 62 | 10 | 144 |
| Diabetes mellitus - Acute | 20 | 0 | 0 |
| Disorders of the brain | 14 | 5 | 0 |
| Early complications of trauma | 18 | 0 | 36 |
| Ectopic pregnancy | 5 | 0 | 0 |
| Encephalitis, myelitis and encephalomyelitis | 18 | 0 | 0 |
| Environmental exposures | 6 | 5 | 22 |
| Femur fracture | 462 | 0 | 2004 |
| Gastrointestinal tract bleeding and/or perforation | 39 | 27 | 0 |

© 2019 Vashi AA et al. *JAMA Network Open.*

| | | | |
|---|---|---|---|
| Heart failure | 14 | 0 | 0 |
| Infectious fasciitis | 2 | 5 | 0 |
| Intracranial hemorrhage | 34 | 1 | 0 |
| Intracranial injury | 190 | 0 | 380 |
| Meningitis | 19 | 2 | 0 |
| Moderate-severe burns and corrosions | 548 | 0 | 880 |
| Myocardial infarction | 9 | 0 | 0 |
| Neck injuries | 303 | 6 | 618 |
| Other cardiac arrhythmia | 7 | 11 | 0 |
| Other diseases of intestine | 3 | 6 | 0 |
| Other tachyarrhythmias | 8 | 2 | 0 |
| Overdose/Poisonings | 572 | 68 | 1280 |
| Paralytic ileus and intestinal obstruction without hernia | 10 | 0 | 0 |
| Pericardial disease, endocarditis, and myocarditis | 26 | 0 | 0 |
| Peritonitis | 8 | 0 | 0 |
| Pneumonia | 37 | 0 | 0 |
| Pneumothorax | 5 | 2 | 0 |
| Postpartum hemorrhage | 4 | 0 | 0 |
| Pre-eclampsia/Eclampsia | 18 | 0 | 0 |
| Pulmonary embolism | 6 | 0 | 0 |
| Respiratory failure | 12 | 0 | 0 |
| Sepsis and Systemic inflammatory response syndrome (SIRS) | 35 | 5 | 0 |
| Septic arthritis | 122 | 0 | 0 |
| Shock | 4 | 0 | 0 |
| Thoracic injuries | 391 | 9 | 800 |
| Volume depletion | 3 | 0 | 0 |

[a]ICD-10-CM diagnosis subcodes indicating pediatric, chronic, or subacute conditions were excluded.

[b]ICD-10-CM diagnosis subcodes describing subsequent encounters or sequelae were excluded. Because subsequent encounters and sequelae are identified by the character in the 7th position of the ICD-10 code (D=subsequent and S=sequela), we do not include these in the supplemental tables that list the specific inclusion and exclusion subcodes for each condition group.

© 2019 Vashi AA et al. *JAMA Network Open.*

## Included and Excluded ICD-10-CM Diagnosis Subcodes for Abdominal, Lower Back, Pelvic and External Genitalia Injuries Condition Group

| Included ICD-10-CM diagnosis subcodes (n=763) | | Excluded ICD-10-CM diagnosis subcodes (n=3) | |
|---|---|---|---|
| **Subcode** | **Subcode description** | **Subcode** | **Subcode description** |
| S31.000A | Unspecified open wound of lower back and pelvis without penetration into retroperitoneum, initial encounter | S39.011A | Strain of muscle, fascia and tendon of abdomen, initial encounter |
| S31.001A | Unspecified open wound of lower back and pelvis with penetration into retroperitoneum, initial encounter | S39.012A | Strain of muscle, fascia and tendon of lower back, initial encounter |
| S31.010A | Laceration without foreign body of lower back and pelvis without penetration into retroperitoneum, initial encounter | S39.013A | Strain of muscle, fascia and tendon of pelvis, initial encounter |
| S31.011A | Laceration without foreign body of lower back and pelvis with penetration into retroperitoneum, initial encounter | | |
| S31.020A | Laceration with foreign body of lower back and pelvis without penetration into retroperitoneum, initial encounter | | |
| S31.021A | Laceration with foreign body of lower back and pelvis with penetration into retroperitoneum, initial encounter | | |
| S31.030A | Puncture wound without foreign body of lower back and pelvis without penetration into retroperitoneum, initial encounter | | |
| S31.031A | Puncture wound without foreign body of lower back and pelvis with penetration into retroperitoneum, initial encounter | | |
| S31.040A | Puncture wound with foreign body of lower back and pelvis without penetration into retroperitoneum, initial encounter | | |
| S31.041A | Puncture wound with foreign body of lower back and pelvis with penetration into retroperitoneum, initial encounter | | |
| S31.050A | Open bite of lower back and pelvis without penetration into retroperitoneum, initial encounter | | |
| S31.051A | Open bite of lower back and pelvis with penetration into retroperitoneum, initial encounter | | |
| S31.100A | Unspecified open wound of abdominal wall, right upper quadrant without penetration into peritoneal cavity, initial encounter | | |

© 2019 Vashi AA et al. *JAMA Network Open.*

| | | | |
|---|---|---|---|
| S31.101A | Unspecified open wound of abdominal wall, left upper quadrant without penetration into peritoneal cavity, initial encounter | | |
| S31.102A | Unspecified open wound of abdominal wall, epigastric region without penetration into peritoneal cavity, initial encounter | | |
| S31.103A | Unspecified open wound of abdominal wall, right lower quadrant without penetration into peritoneal cavity, initial encounter | | |
| S31.104A | Unspecified open wound of abdominal wall, left lower quadrant without penetration into peritoneal cavity, initial encounter | | |
| S31.105A | Unspecified open wound of abdominal wall, periumbilic region without penetration into peritoneal cavity, initial encounter | | |
| S31.109A | Unspecified open wound of abdominal wall, unspecified quadrant without penetration into peritoneal cavity, initial encounter | | |
| S31.110A | Laceration without foreign body of abdominal wall, right upper quadrant without penetration into peritoneal cavity, initial encounter | | |
| S31.111A | Laceration without foreign body of abdominal wall, left upper quadrant without penetration into peritoneal cavity, initial encounter | | |
| S31.112A | Laceration without foreign body of abdominal wall, epigastric region without penetration into peritoneal cavity, initial encounter | | |
| S31.113A | Laceration without foreign body of abdominal wall, right lower quadrant without penetration into peritoneal cavity, initial encounter | | |
| S31.114A | Laceration without foreign body of abdominal wall, left lower quadrant without penetration into peritoneal cavity, initial encounter | | |
| S31.115A | Laceration without foreign body of abdominal wall, periumbilic region without penetration into peritoneal cavity, initial encounter | | |
| S31.119A | Laceration without foreign body of abdominal wall, unspecified quadrant without penetration into peritoneal cavity, initial encounter | | |

© 2019 Vashi AA et al. *JAMA Network Open.*

| | | | |
|---|---|---|---|
| S31.120A | Laceration of abdominal wall with foreign body, right upper quadrant without penetration into peritoneal cavity, initial encounter | | |
| S31.121A | Laceration of abdominal wall with foreign body, left upper quadrant without penetration into peritoneal cavity, initial encounter | | |
| S31.122A | Laceration of abdominal wall with foreign body, epigastric region without penetration into peritoneal cavity, initial encounter | | |
| S31.123A | Laceration of abdominal wall with foreign body, right lower quadrant without penetration into peritoneal cavity, initial encounter | | |
| S31.124A | Laceration of abdominal wall with foreign body, left lower quadrant without penetration into peritoneal cavity, initial encounter | | |
| S31.125A | Laceration of abdominal wall with foreign body, periumbilic region without penetration into peritoneal cavity, initial encounter | | |
| S31.129A | Laceration of abdominal wall with foreign body, unspecified quadrant without penetration into peritoneal cavity, initial encounter | | |
| S31.130A | Puncture wound of abdominal wall without foreign body, right upper quadrant without penetration into peritoneal cavity, initial encounter | | |
| S31.131A | Puncture wound of abdominal wall without foreign body, left upper quadrant without penetration into peritoneal cavity, initial encounter | | |
| S31.132A | Puncture wound of abdominal wall without foreign body, epigastric region without penetration into peritoneal cavity, initial encounter | | |
| S31.133A | Puncture wound of abdominal wall without foreign body, right lower quadrant without penetration into peritoneal cavity, initial encounter | | |
| S31.134A | Puncture wound of abdominal wall without foreign body, left lower quadrant without penetration into peritoneal cavity, initial encounter | | |
| S31.135A | Puncture wound of abdominal wall without foreign body, periumbilic region without penetration into peritoneal cavity, initial encounter | | |

© 2019 Vashi AA et al. *JAMA Network Open.*

| | | | |
|---|---|---|---|
| S31.139A | Puncture wound of abdominal wall without foreign body, unspecified quadrant without penetration into peritoneal cavity, initial encounter | | |
| S31.140A | Puncture wound of abdominal wall with foreign body, right upper quadrant without penetration into peritoneal cavity, initial encounter | | |
| S31.141A | Puncture wound of abdominal wall with foreign body, left upper quadrant without penetration into peritoneal cavity, initial encounter | | |
| S31.142A | Puncture wound of abdominal wall with foreign body, epigastric region without penetration into peritoneal cavity, initial encounter | | |
| S31.143A | Puncture wound of abdominal wall with foreign body, right lower quadrant without penetration into peritoneal cavity, initial encounter | | |
| S31.144A | Puncture wound of abdominal wall with foreign body, left lower quadrant without penetration into peritoneal cavity, initial encounter | | |
| S31.145A | Puncture wound of abdominal wall with foreign body, periumbilic region without penetration into peritoneal cavity, initial encounter | | |
| S31.149A | Puncture wound of abdominal wall with foreign body, unspecified quadrant without penetration into peritoneal cavity, initial encounter | | |
| S31.150A | Open bite of abdominal wall, right upper quadrant without penetration into peritoneal cavity, initial encounter | | |
| S31.151A | Open bite of abdominal wall, left upper quadrant without penetration into peritoneal cavity, initial encounter | | |
| S31.152A | Open bite of abdominal wall, epigastric region without penetration into peritoneal cavity, initial encounter | | |
| S31.153A | Open bite of abdominal wall, right lower quadrant without penetration into peritoneal cavity, initial encounter | | |
| S31.154A | Open bite of abdominal wall, left lower quadrant without penetration into peritoneal cavity, initial encounter | | |
| S31.155A | Open bite of abdominal wall, periumbilic region without penetration into peritoneal cavity, initial encounter | | |

© 2019 Vashi AA et al. *JAMA Network Open.*

| | | | |
|---|---|---|---|
| S31.159A | Open bite of abdominal wall, unspecified quadrant without penetration into peritoneal cavity, initial encounter | | |
| S31.20XA | Unspecified open wound of penis, initial encounter | | |
| S31.21XA | Laceration without foreign body of penis, initial encounter | | |
| S31.22XA | Laceration with foreign body of penis, initial encounter | | |
| S31.23XA | Puncture wound without foreign body of penis, initial encounter | | |
| S31.24XA | Puncture wound with foreign body of penis, initial encounter | | |
| S31.25XA | Open bite of penis, initial encounter | | |
| S31.30XA | Unspecified open wound of scrotum and testes, initial encounter | | |
| S31.31XA | Laceration without foreign body of scrotum and testes, initial encounter | | |
| S31.32XA | Laceration with foreign body of scrotum and testes, initial encounter | | |
| S31.33XA | Puncture wound without foreign body of scrotum and testes, initial encounter | | |
| S31.34XA | Puncture wound with foreign body of scrotum and testes, initial encounter | | |
| S31.35XA | Open bite of scrotum and testes, initial encounter | | |
| S31.40XA | Unspecified open wound of vagina and vulva, initial encounter | | |
| S31.41XA | Laceration without foreign body of vagina and vulva, initial encounter | | |
| S31.42XA | Laceration with foreign body of vagina and vulva, initial encounter | | |
| S31.43XA | Puncture wound without foreign body of vagina and vulva, initial encounter | | |
| S31.44XA | Puncture wound with foreign body of vagina and vulva, initial encounter | | |
| S31.45XA | Open bite of vagina and vulva, initial encounter | | |
| S31.501A | Unspecified open wound of unspecified external genital organs, male, initial encounter | | |
| S31.502A | Unspecified open wound of unspecified external genital organs, female, initial encounter | | |
| S31.511A | Laceration without foreign body of unspecified external genital organs, male, initial encounter | | |

© 2019 Vashi AA et al. *JAMA Network Open.*

| | | | |
|---|---|---|---|
| S31.512A | Laceration without foreign body of unspecified external genital organs, female, initial encounter | | |
| S31.521A | Laceration with foreign body of unspecified external genital organs, male, initial encounter | | |
| S31.522A | Laceration with foreign body of unspecified external genital organs, female, initial encounter | | |
| S31.531A | Puncture wound without foreign body of unspecified external genital organs, male, initial encounter | | |
| S31.532A | Puncture wound without foreign body of unspecified external genital organs, female, initial encounter | | |
| S31.541A | Puncture wound with foreign body of unspecified external genital organs, male, initial encounter | | |
| S31.542A | Puncture wound with foreign body of unspecified external genital organs, female, initial encounter | | |
| S31.551A | Open bite of unspecified external genital organs, male, initial encounter | | |
| S31.552A | Open bite of unspecified external genital organs, female, initial encounter | | |
| S31.600A | Unspecified open wound of abdominal wall, right upper quadrant with penetration into peritoneal cavity, initial encounter | | |
| S31.601A | Unspecified open wound of abdominal wall, left upper quadrant with penetration into peritoneal cavity, initial encounter | | |
| S31.602A | Unspecified open wound of abdominal wall, epigastric region with penetration into peritoneal cavity, initial encounter | | |
| S31.603A | Unspecified open wound of abdominal wall, right lower quadrant with penetration into peritoneal cavity, initial encounter | | |
| S31.604A | Unspecified open wound of abdominal wall, left lower quadrant with penetration into peritoneal cavity, initial encounter | | |
| S31.605A | Unspecified open wound of abdominal wall, periumbilic region with penetration into peritoneal cavity, initial encounter | | |
| S31.609A | Unspecified open wound of abdominal wall, unspecified quadrant with penetration into peritoneal cavity, initial encounter | | |

© 2019 Vashi AA et al. *JAMA Network Open.*

| | | | |
|---|---|---|---|
| S31.610A | Laceration without foreign body of abdominal wall, right upper quadrant with penetration into peritoneal cavity, initial encounter | | |
| S31.611A | Laceration without foreign body of abdominal wall, left upper quadrant with penetration into peritoneal cavity, initial encounter | | |
| S31.612A | Laceration without foreign body of abdominal wall, epigastric region with penetration into peritoneal cavity, initial encounter | | |
| S31.613A | Laceration without foreign body of abdominal wall, right lower quadrant with penetration into peritoneal cavity, initial encounter | | |
| S31.614A | Laceration without foreign body of abdominal wall, left lower quadrant with penetration into peritoneal cavity, initial encounter | | |
| S31.615A | Laceration without foreign body of abdominal wall, periumbilic region with penetration into peritoneal cavity, initial encounter | | |
| S31.619A | Laceration without foreign body of abdominal wall, unspecified quadrant with penetration into peritoneal cavity, initial encounter | | |
| S31.620A | Laceration with foreign body of abdominal wall, right upper quadrant with penetration into peritoneal cavity, initial encounter | | |
| S31.621A | Laceration with foreign body of abdominal wall, left upper quadrant with penetration into peritoneal cavity, initial encounter | | |
| S31.622A | Laceration with foreign body of abdominal wall, epigastric region with penetration into peritoneal cavity, initial encounter | | |
| S31.623A | Laceration with foreign body of abdominal wall, right lower quadrant with penetration into peritoneal cavity, initial encounter | | |
| S31.624A | Laceration with foreign body of abdominal wall, left lower quadrant with penetration into peritoneal cavity, initial encounter | | |
| S31.625A | Laceration with foreign body of abdominal wall, periumbilic region with penetration into peritoneal cavity, initial encounter | | |

© 2019 Vashi AA et al. *JAMA Network Open.*

| | | | |
|---|---|---|---|
| S31.629A | Laceration with foreign body of abdominal wall, unspecified quadrant with penetration into peritoneal cavity, initial encounter | | |
| S31.630A | Puncture wound without foreign body of abdominal wall, right upper quadrant with penetration into peritoneal cavity, initial encounter | | |
| S31.631A | Puncture wound without foreign body of abdominal wall, left upper quadrant with penetration into peritoneal cavity, initial encounter | | |
| S31.632A | Puncture wound without foreign body of abdominal wall, epigastric region with penetration into peritoneal cavity, initial encounter | | |
| S31.633A | Puncture wound without foreign body of abdominal wall, right lower quadrant with penetration into peritoneal cavity, initial encounter | | |
| S31.634A | Puncture wound without foreign body of abdominal wall, left lower quadrant with penetration into peritoneal cavity, initial encounter | | |
| S31.635A | Puncture wound without foreign body of abdominal wall, periumbilic region with penetration into peritoneal cavity, initial encounter | | |
| S31.639A | Puncture wound without foreign body of abdominal wall, unspecified quadrant with penetration into peritoneal cavity, initial encounter | | |
| S31.640A | Puncture wound with foreign body of abdominal wall, right upper quadrant with penetration into peritoneal cavity, initial encounter | | |
| S31.641A | Puncture wound with foreign body of abdominal wall, left upper quadrant with penetration into peritoneal cavity, initial encounter | | |
| S31.642A | Puncture wound with foreign body of abdominal wall, epigastric region with penetration into peritoneal cavity, initial encounter | | |
| S31.643A | Puncture wound with foreign body of abdominal wall, right lower quadrant with penetration into peritoneal cavity, initial encounter | | |
| S31.644A | Puncture wound with foreign body of abdominal wall, left lower quadrant with penetration into peritoneal cavity, initial encounter | | |

© 2019 Vashi AA et al. *JAMA Network Open.*

| | | | |
|---|---|---|---|
| S31.645A | Puncture wound with foreign body of abdominal wall, periumbilic region with penetration into peritoneal cavity, initial encounter | | |
| S31.649A | Puncture wound with foreign body of abdominal wall, unspecified quadrant with penetration into peritoneal cavity, initial encounter | | |
| S31.650A | Open bite of abdominal wall, right upper quadrant with penetration into peritoneal cavity, initial encounter | | |
| S31.651A | Open bite of abdominal wall, left upper quadrant with penetration into peritoneal cavity, initial encounter | | |
| S31.652A | Open bite of abdominal wall, epigastric region with penetration into peritoneal cavity, initial encounter | | |
| S31.653A | Open bite of abdominal wall, right lower quadrant with penetration into peritoneal cavity, initial encounter | | |
| S31.654A | Open bite of abdominal wall, left lower quadrant with penetration into peritoneal cavity, initial encounter | | |
| S31.655A | Open bite of abdominal wall, periumbilic region with penetration into peritoneal cavity, initial encounter | | |
| S31.659A | Open bite of abdominal wall, unspecified quadrant with penetration into peritoneal cavity, initial encounter | | |
| S31.801A | Laceration without foreign body of unspecified buttock, initial encounter | | |
| S31.802A | Laceration with foreign body of unspecified buttock, initial encounter | | |
| S31.803A | Puncture wound without foreign body of unspecified buttock, initial encounter | | |
| S31.804A | Puncture wound with foreign body of unspecified buttock, initial encounter | | |
| S31.805A | Open bite of unspecified buttock, initial encounter | | |
| S31.809A | Unspecified open wound of unspecified buttock, initial encounter | | |
| S31.811A | Laceration without foreign body of right buttock, initial encounter | | |
| S31.812A | Laceration with foreign body of right buttock, initial encounter | | |
| S31.813A | Puncture wound without foreign body of right buttock, initial encounter | | |

© 2019 Vashi AA et al. *JAMA Network Open.*

| | | | |
|---|---|---|---|
| S31.814A | Puncture wound with foreign body of right buttock, initial encounter | | |
| S31.815A | Open bite of right buttock, initial encounter | | |
| S31.819A | Unspecified open wound of right buttock, initial encounter | | |
| S31.821A | Laceration without foreign body of left buttock, initial encounter | | |
| S31.822A | Laceration with foreign body of left buttock, initial encounter | | |
| S31.823A | Puncture wound without foreign body of left buttock, initial encounter | | |
| S31.824A | Puncture wound with foreign body of left buttock, initial encounter | | |
| S31.825A | Open bite of left buttock, initial encounter | | |
| S31.829A | Unspecified open wound of left buttock, initial encounter | | |
| S31.831A | Laceration without foreign body of anus, initial encounter | | |
| S31.832A | Laceration with foreign body of anus, initial encounter | | |
| S31.833A | Puncture wound without foreign body of anus, initial encounter | | |
| S31.834A | Puncture wound with foreign body of anus, initial encounter | | |
| S31.835A | Open bite of anus, initial encounter | | |
| S31.839A | Unspecified open wound of anus, initial encounter | | |
| S32.000A | Wedge compression fracture of unspecified lumbar vertebra, initial encounter for closed fracture | | |
| S32.000B | Wedge compression fracture of unspecified lumbar vertebra, initial encounter for open fracture | | |
| S32.001A | Stable burst fracture of unspecified lumbar vertebra, initial encounter for closed fracture | | |
| S32.001B | Stable burst fracture of unspecified lumbar vertebra, initial encounter for open fracture | | |
| S32.002A | Unstable burst fracture of unspecified lumbar vertebra, initial encounter for closed fracture | | |
| S32.002B | Unstable burst fracture of unspecified lumbar vertebra, initial encounter for open fracture | | |
| S32.008A | Other fracture of unspecified lumbar vertebra, initial encounter for closed fracture | | |
| S32.008B | Other fracture of unspecified lumbar vertebra, initial encounter for open fracture | | |

© 2019 Vashi AA et al. *JAMA Network Open.*

| | | | |
|---|---|---|---|
| S32.009A | Unspecified fracture of unspecified lumbar vertebra, initial encounter for closed fracture | | |
| S32.009B | Unspecified fracture of unspecified lumbar vertebra, initial encounter for open fracture | | |
| S32.010A | Wedge compression fracture of first lumbar vertebra, initial encounter for closed fracture | | |
| S32.010B | Wedge compression fracture of first lumbar vertebra, initial encounter for open fracture | | |
| S32.011A | Stable burst fracture of first lumbar vertebra, initial encounter for closed fracture | | |
| S32.011B | Stable burst fracture of first lumbar vertebra, initial encounter for open fracture | | |
| S32.012A | Unstable burst fracture of first lumbar vertebra, initial encounter for closed fracture | | |
| S32.012B | Unstable burst fracture of first lumbar vertebra, initial encounter for open fracture | | |
| S32.018A | Other fracture of first lumbar vertebra, initial encounter for closed fracture | | |
| S32.018B | Other fracture of first lumbar vertebra, initial encounter for open fracture | | |
| S32.019A | Unspecified fracture of first lumbar vertebra, initial encounter for closed fracture | | |
| S32.019B | Unspecified fracture of first lumbar vertebra, initial encounter for open fracture | | |
| S32.020A | Wedge compression fracture of second lumbar vertebra, initial encounter for closed fracture | | |
| S32.020B | Wedge compression fracture of second lumbar vertebra, initial encounter for open fracture | | |
| S32.021A | Stable burst fracture of second lumbar vertebra, initial encounter for closed fracture | | |
| S32.021B | Stable burst fracture of second lumbar vertebra, initial encounter for open fracture | | |
| S32.022A | Unstable burst fracture of second lumbar vertebra, initial encounter for closed fracture | | |
| S32.022B | Unstable burst fracture of second lumbar vertebra, initial encounter for open fracture | | |
| S32.028A | Other fracture of second lumbar vertebra, initial encounter for closed fracture | | |

© 2019 Vashi AA et al. *JAMA Network Open.*

| | | | |
|---|---|---|---|
| S32.028B | Other fracture of second lumbar vertebra, initial encounter for open fracture | | |
| S32.029A | Unspecified fracture of second lumbar vertebra, initial encounter for closed fracture | | |
| S32.029B | Unspecified fracture of second lumbar vertebra, initial encounter for open fracture | | |
| S32.030A | Wedge compression fracture of third lumbar vertebra, initial encounter for closed fracture | | |
| S32.030B | Wedge compression fracture of third lumbar vertebra, initial encounter for open fracture | | |
| S32.031A | Stable burst fracture of third lumbar vertebra, initial encounter for closed fracture | | |
| S32.031B | Stable burst fracture of third lumbar vertebra, initial encounter for open fracture | | |
| S32.032A | Unstable burst fracture of third lumbar vertebra, initial encounter for closed fracture | | |
| S32.032B | Unstable burst fracture of third lumbar vertebra, initial encounter for open fracture | | |
| S32.038A | Other fracture of third lumbar vertebra, initial encounter for closed fracture | | |
| S32.038B | Other fracture of third lumbar vertebra, initial encounter for open fracture | | |
| S32.039A | Unspecified fracture of third lumbar vertebra, initial encounter for closed fracture | | |
| S32.039B | Unspecified fracture of third lumbar vertebra, initial encounter for open fracture | | |
| S32.040A | Wedge compression fracture of fourth lumbar vertebra, initial encounter for closed fracture | | |
| S32.040B | Wedge compression fracture of fourth lumbar vertebra, initial encounter for open fracture | | |
| S32.041A | Stable burst fracture of fourth lumbar vertebra, initial encounter for closed fracture | | |
| S32.041B | Stable burst fracture of fourth lumbar vertebra, initial encounter for open fracture | | |
| S32.042A | Unstable burst fracture of fourth lumbar vertebra, initial encounter for closed fracture | | |
| S32.042B | Unstable burst fracture of fourth lumbar vertebra, initial encounter for open fracture | | |

© 2019 Vashi AA et al. *JAMA Network Open.*

| | | | |
|---|---|---|---|
| S32.048A | Other fracture of fourth lumbar vertebra, initial encounter for closed fracture | | |
| S32.048B | Other fracture of fourth lumbar vertebra, initial encounter for open fracture | | |
| S32.049A | Unspecified fracture of fourth lumbar vertebra, initial encounter for closed fracture | | |
| S32.049B | Unspecified fracture of fourth lumbar vertebra, initial encounter for open fracture | | |
| S32.050A | Wedge compression fracture of fifth lumbar vertebra, initial encounter for closed fracture | | |
| S32.050B | Wedge compression fracture of fifth lumbar vertebra, initial encounter for open fracture | | |
| S32.051A | Stable burst fracture of fifth lumbar vertebra, initial encounter for closed fracture | | |
| S32.051B | Stable burst fracture of fifth lumbar vertebra, initial encounter for open fracture | | |
| S32.052A | Unstable burst fracture of fifth lumbar vertebra, initial encounter for closed fracture | | |
| S32.052B | Unstable burst fracture of fifth lumbar vertebra, initial encounter for open fracture | | |
| S32.058A | Other fracture of fifth lumbar vertebra, initial encounter for closed fracture | | |
| S32.058B | Other fracture of fifth lumbar vertebra, initial encounter for open fracture | | |
| S32.059A | Unspecified fracture of fifth lumbar vertebra, initial encounter for closed fracture | | |
| S32.059B | Unspecified fracture of fifth lumbar vertebra, initial encounter for open fracture | | |
| S32.10XA | Unspecified fracture of sacrum, initial encounter for closed fracture | | |
| S32.10XB | Unspecified fracture of sacrum, initial encounter for open fracture | | |
| S32.110A | Nondisplaced Zone I fracture of sacrum, initial encounter for closed fracture | | |
| S32.110B | Nondisplaced Zone I fracture of sacrum, initial encounter for open fracture | | |
| S32.111A | Minimally displaced Zone I fracture of sacrum, initial encounter for closed fracture | | |

© 2019 Vashi AA et al. *JAMA Network Open.*

| | | | |
|---|---|---|---|
| S32.111B | Minimally displaced Zone I fracture of sacrum, initial encounter for open fracture | | |
| S32.112A | Severely displaced Zone I fracture of sacrum, initial encounter for closed fracture | | |
| S32.112B | Severely displaced Zone I fracture of sacrum, initial encounter for open fracture | | |
| S32.119A | Unspecified Zone I fracture of sacrum, initial encounter for closed fracture | | |
| S32.119B | Unspecified Zone I fracture of sacrum, initial encounter for open fracture | | |
| S32.120A | Nondisplaced Zone II fracture of sacrum, initial encounter for closed fracture | | |
| S32.120B | Nondisplaced Zone II fracture of sacrum, initial encounter for open fracture | | |
| S32.121A | Minimally displaced Zone II fracture of sacrum, initial encounter for closed fracture | | |
| S32.121B | Minimally displaced Zone II fracture of sacrum, initial encounter for open fracture | | |
| S32.122A | Severely displaced Zone II fracture of sacrum, initial encounter for closed fracture | | |
| S32.122B | Severely displaced Zone II fracture of sacrum, initial encounter for open fracture | | |
| S32.129A | Unspecified Zone II fracture of sacrum, initial encounter for closed fracture | | |
| S32.129B | Unspecified Zone II fracture of sacrum, initial encounter for open fracture | | |
| S32.130A | Nondisplaced Zone III fracture of sacrum, initial encounter for closed fracture | | |
| S32.130B | Nondisplaced Zone III fracture of sacrum, initial encounter for open fracture | | |
| S32.131A | Minimally displaced Zone III fracture of sacrum, initial encounter for closed fracture | | |
| S32.131B | Minimally displaced Zone III fracture of sacrum, initial encounter for open fracture | | |
| S32.132A | Severely displaced Zone III fracture of sacrum, initial encounter for closed fracture | | |
| S32.132B | Severely displaced Zone III fracture of sacrum, initial encounter for open fracture | | |

© 2019 Vashi AA et al. *JAMA Network Open.*

| | | | |
|---|---|---|---|
| S32.139A | Unspecified Zone III fracture of sacrum, initial encounter for closed fracture | | |
| S32.139B | Unspecified Zone III fracture of sacrum, initial encounter for open fracture | | |
| S32.14XA | Type 1 fracture of sacrum, initial encounter for closed fracture | | |
| S32.14XB | Type 1 fracture of sacrum, initial encounter for open fracture | | |
| S32.15XA | Type 2 fracture of sacrum, initial encounter for closed fracture | | |
| S32.15XB | Type 2 fracture of sacrum, initial encounter for open fracture | | |
| S32.16XA | Type 3 fracture of sacrum, initial encounter for closed fracture | | |
| S32.16XB | Type 3 fracture of sacrum, initial encounter for open fracture | | |
| S32.17XA | Type 4 fracture of sacrum, initial encounter for closed fracture | | |
| S32.17XB | Type 4 fracture of sacrum, initial encounter for open fracture | | |
| S32.19XA | Other fracture of sacrum, initial encounter for closed fracture | | |
| S32.19XB | Other fracture of sacrum, initial encounter for open fracture | | |
| S32.2XXA | Fracture of coccyx, initial encounter for closed fracture | | |
| S32.2XXB | Fracture of coccyx, initial encounter for open fracture | | |
| S32.301A | Unspecified fracture of right ilium, initial encounter for closed fracture | | |
| S32.301B | Unspecified fracture of right ilium, initial encounter for open fracture | | |
| S32.302A | Unspecified fracture of left ilium, initial encounter for closed fracture | | |
| S32.302B | Unspecified fracture of left ilium, initial encounter for open fracture | | |
| S32.309A | Unspecified fracture of unspecified ilium, initial encounter for closed fracture | | |
| S32.309B | Unspecified fracture of unspecified ilium, initial encounter for open fracture | | |

© 2019 Vashi AA et al. *JAMA Network Open.*

| | | | |
|---|---|---|---|
| S32.311A | Displaced avulsion fracture of right ilium, initial encounter for closed fracture | | |
| S32.311B | Displaced avulsion fracture of right ilium, initial encounter for open fracture | | |
| S32.312A | Displaced avulsion fracture of left ilium, initial encounter for closed fracture | | |
| S32.312B | Displaced avulsion fracture of left ilium, initial encounter for open fracture | | |
| S32.313A | Displaced avulsion fracture of unspecified ilium, initial encounter for closed fracture | | |
| S32.313B | Displaced avulsion fracture of unspecified ilium, initial encounter for open fracture | | |
| S32.314A | Nondisplaced avulsion fracture of right ilium, initial encounter for closed fracture | | |
| S32.314B | Nondisplaced avulsion fracture of right ilium, initial encounter for open fracture | | |
| S32.315A | Nondisplaced avulsion fracture of left ilium, initial encounter for closed fracture | | |
| S32.315B | Nondisplaced avulsion fracture of left ilium, initial encounter for open fracture | | |
| S32.316A | Nondisplaced avulsion fracture of unspecified ilium, initial encounter for closed fracture | | |
| S32.316B | Nondisplaced avulsion fracture of unspecified ilium, initial encounter for open fracture | | |
| S32.391A | Other fracture of right ilium, initial encounter for closed fracture | | |
| S32.391B | Other fracture of right ilium, initial encounter for open fracture | | |
| S32.392A | Other fracture of left ilium, initial encounter for closed fracture | | |
| S32.392B | Other fracture of left ilium, initial encounter for open fracture | | |
| S32.399A | Other fracture of unspecified ilium, initial encounter for closed fracture | | |
| S32.399B | Other fracture of unspecified ilium, initial encounter for open fracture | | |
| S32.401A | Unspecified fracture of right acetabulum, initial encounter for closed fracture | | |

© 2019 Vashi AA et al. *JAMA Network Open.*

| | | | |
|---|---|---|---|
| S32.401B | Unspecified fracture of right acetabulum, initial encounter for open fracture | | |
| S32.402A | Unspecified fracture of left acetabulum, initial encounter for closed fracture | | |
| S32.402B | Unspecified fracture of left acetabulum, initial encounter for open fracture | | |
| S32.409A | Unspecified fracture of unspecified acetabulum, initial encounter for closed fracture | | |
| S32.409B | Unspecified fracture of unspecified acetabulum, initial encounter for open fracture | | |
| S32.411A | Displaced fracture of anterior wall of right acetabulum, initial encounter for closed fracture | | |
| S32.411B | Displaced fracture of anterior wall of right acetabulum, initial encounter for open fracture | | |
| S32.412A | Displaced fracture of anterior wall of left acetabulum, initial encounter for closed fracture | | |
| S32.412B | Displaced fracture of anterior wall of left acetabulum, initial encounter for open fracture | | |
| S32.413A | Displaced fracture of anterior wall of unspecified acetabulum, initial encounter for closed fracture | | |
| S32.413B | Displaced fracture of anterior wall of unspecified acetabulum, initial encounter for open fracture | | |
| S32.414A | Nondisplaced fracture of anterior wall of right acetabulum, initial encounter for closed fracture | | |
| S32.414B | Nondisplaced fracture of anterior wall of right acetabulum, initial encounter for open fracture | | |
| S32.415A | Nondisplaced fracture of anterior wall of left acetabulum, initial encounter for closed fracture | | |
| S32.415B | Nondisplaced fracture of anterior wall of left acetabulum, initial encounter for open fracture | | |
| S32.416A | Nondisplaced fracture of anterior wall of unspecified acetabulum, initial encounter for closed fracture | | |
| S32.416B | Nondisplaced fracture of anterior wall of unspecified acetabulum, initial encounter for open fracture | | |
| S32.421A | Displaced fracture of posterior wall of right acetabulum, initial encounter for closed fracture | | |
| S32.421B | Displaced fracture of posterior wall of right acetabulum, initial encounter for open fracture | | |

© 2019 Vashi AA et al. *JAMA Network Open.*

| | | | |
|---|---|---|---|
| S32.422A | Displaced fracture of posterior wall of left acetabulum, initial encounter for closed fracture | | |
| S32.422B | Displaced fracture of posterior wall of left acetabulum, initial encounter for open fracture | | |
| S32.423A | Displaced fracture of posterior wall of unspecified acetabulum, initial encounter for closed fracture | | |
| S32.423B | Displaced fracture of posterior wall of unspecified acetabulum, initial encounter for open fracture | | |
| S32.424A | Nondisplaced fracture of posterior wall of right acetabulum, initial encounter for closed fracture | | |
| S32.424B | Nondisplaced fracture of posterior wall of right acetabulum, initial encounter for open fracture | | |
| S32.425A | Nondisplaced fracture of posterior wall of left acetabulum, initial encounter for closed fracture | | |
| S32.425B | Nondisplaced fracture of posterior wall of left acetabulum, initial encounter for open fracture | | |
| S32.426A | Nondisplaced fracture of posterior wall of unspecified acetabulum, initial encounter for closed fracture | | |
| S32.426B | Nondisplaced fracture of posterior wall of unspecified acetabulum, initial encounter for open fracture | | |
| S32.431A | Displaced fracture of anterior column [iliopubic] of right acetabulum, initial encounter for closed fracture | | |
| S32.431B | Displaced fracture of anterior column [iliopubic] of right acetabulum, initial encounter for open fracture | | |
| S32.432A | Displaced fracture of anterior column [iliopubic] of left acetabulum, initial encounter for closed fracture | | |
| S32.432B | Displaced fracture of anterior column [iliopubic] of left acetabulum, initial encounter for open fracture | | |
| S32.433A | Displaced fracture of anterior column [iliopubic] of unspecified acetabulum, initial encounter for closed fracture | | |
| S32.433B | Displaced fracture of anterior column [iliopubic] of unspecified acetabulum, initial encounter for open fracture | | |
| S32.434A | Nondisplaced fracture of anterior column [iliopubic] of right acetabulum, initial encounter for closed fracture | | |
| S32.434B | Nondisplaced fracture of anterior column [iliopubic] of right acetabulum, initial encounter for open fracture | | |

© 2019 Vashi AA et al. *JAMA Network Open.*

| | | | |
|---|---|---|---|
| S32.435A | Nondisplaced fracture of anterior column [iliopubic] of left acetabulum, initial encounter for closed fracture | | |
| S32.435B | Nondisplaced fracture of anterior column [iliopubic] of left acetabulum, initial encounter for open fracture | | |
| S32.436A | Nondisplaced fracture of anterior column [iliopubic] of unspecified acetabulum, initial encounter for closed fracture | | |
| S32.436B | Nondisplaced fracture of anterior column [iliopubic] of unspecified acetabulum, initial encounter for open fracture | | |
| S32.441A | Displaced fracture of posterior column [ilioischial] of right acetabulum, initial encounter for closed fracture | | |
| S32.441B | Displaced fracture of posterior column [ilioischial] of right acetabulum, initial encounter for open fracture | | |
| S32.442A | Displaced fracture of posterior column [ilioischial] of left acetabulum, initial encounter for closed fracture | | |
| S32.442B | Displaced fracture of posterior column [ilioischial] of left acetabulum, initial encounter for open fracture | | |
| S32.443A | Displaced fracture of posterior column [ilioischial] of unspecified acetabulum, initial encounter for closed fracture | | |
| S32.443B | Displaced fracture of posterior column [ilioischial] of unspecified acetabulum, initial encounter for open fracture | | |
| S32.444A | Nondisplaced fracture of posterior column [ilioischial] of right acetabulum, initial encounter for closed fracture | | |
| S32.444B | Nondisplaced fracture of posterior column [ilioischial] of right acetabulum, initial encounter for open fracture | | |
| S32.445A | Nondisplaced fracture of posterior column [ilioischial] of left acetabulum, initial encounter for closed fracture | | |
| S32.445B | Nondisplaced fracture of posterior column [ilioischial] of left acetabulum, initial encounter for open fracture | | |
| S32.446A | Nondisplaced fracture of posterior column [ilioischial] of unspecified acetabulum, initial encounter for closed fracture | | |

© 2019 Vashi AA et al. *JAMA Network Open.*

| | | | |
|---|---|---|---|
| S32.446B | Nondisplaced fracture of posterior column [ilioischial] of unspecified acetabulum, initial encounter for open fracture | | |
| S32.451A | Displaced transverse fracture of right acetabulum, initial encounter for closed fracture | | |
| S32.451B | Displaced transverse fracture of right acetabulum, initial encounter for open fracture | | |
| S32.452A | Displaced transverse fracture of left acetabulum, initial encounter for closed fracture | | |
| S32.452B | Displaced transverse fracture of left acetabulum, initial encounter for open fracture | | |
| S32.453A | Displaced transverse fracture of unspecified acetabulum, initial encounter for closed fracture | | |
| S32.453B | Displaced transverse fracture of unspecified acetabulum, initial encounter for open fracture | | |
| S32.454A | Nondisplaced transverse fracture of right acetabulum, initial encounter for closed fracture | | |
| S32.454B | Nondisplaced transverse fracture of right acetabulum, initial encounter for open fracture | | |
| S32.455A | Nondisplaced transverse fracture of left acetabulum, initial encounter for closed fracture | | |
| S32.455B | Nondisplaced transverse fracture of left acetabulum, initial encounter for open fracture | | |
| S32.456A | Nondisplaced transverse fracture of unspecified acetabulum, initial encounter for closed fracture | | |
| S32.456B | Nondisplaced transverse fracture of unspecified acetabulum, initial encounter for open fracture | | |
| S32.461A | Displaced associated transverse-posterior fracture of right acetabulum, initial encounter for closed fracture | | |
| S32.461B | Displaced associated transverse-posterior fracture of right acetabulum, initial encounter for open fracture | | |
| S32.462A | Displaced associated transverse-posterior fracture of left acetabulum, initial encounter for closed fracture | | |
| S32.462B | Displaced associated transverse-posterior fracture of left acetabulum, initial encounter for open fracture | | |
| S32.463A | Displaced associated transverse-posterior fracture of unspecified acetabulum, initial encounter for closed fracture | | |

© 2019 Vashi AA et al. *JAMA Network Open.*

| | | | |
|---|---|---|---|
| S32.463B | Displaced associated transverse-posterior fracture of unspecified acetabulum, initial encounter for open fracture | | |
| S32.464A | Nondisplaced associated transverse-posterior fracture of right acetabulum, initial encounter for closed fracture | | |
| S32.464B | Nondisplaced associated transverse-posterior fracture of right acetabulum, initial encounter for open fracture | | |
| S32.465A | Nondisplaced associated transverse-posterior fracture of left acetabulum, initial encounter for closed fracture | | |
| S32.465B | Nondisplaced associated transverse-posterior fracture of left acetabulum, initial encounter for open fracture | | |
| S32.466A | Nondisplaced associated transverse-posterior fracture of unspecified acetabulum, initial encounter for closed fracture | | |
| S32.466B | Nondisplaced associated transverse-posterior fracture of unspecified acetabulum, initial encounter for open fracture | | |
| S32.471A | Displaced fracture of medial wall of right acetabulum, initial encounter for closed fracture | | |
| S32.471B | Displaced fracture of medial wall of right acetabulum, initial encounter for open fracture | | |
| S32.472A | Displaced fracture of medial wall of left acetabulum, initial encounter for closed fracture | | |
| S32.472B | Displaced fracture of medial wall of left acetabulum, initial encounter for open fracture | | |
| S32.473A | Displaced fracture of medial wall of unspecified acetabulum, initial encounter for closed fracture | | |
| S32.473B | Displaced fracture of medial wall of unspecified acetabulum, initial encounter for open fracture | | |
| S32.474A | Nondisplaced fracture of medial wall of right acetabulum, initial encounter for closed fracture | | |
| S32.474B | Nondisplaced fracture of medial wall of right acetabulum, initial encounter for open fracture | | |
| S32.475A | Nondisplaced fracture of medial wall of left acetabulum, initial encounter for closed fracture | | |
| S32.475B | Nondisplaced fracture of medial wall of left acetabulum, initial encounter for open fracture | | |
| S32.476A | Nondisplaced fracture of medial wall of unspecified acetabulum, initial encounter for closed fracture | | |

© 2019 Vashi AA et al. *JAMA Network Open.*

| | | | |
|---|---|---|---|
| S32.476B | Nondisplaced fracture of medial wall of unspecified acetabulum, initial encounter for open fracture | | |
| S32.481A | Displaced dome fracture of right acetabulum, initial encounter for closed fracture | | |
| S32.481B | Displaced dome fracture of right acetabulum, initial encounter for open fracture | | |
| S32.482A | Displaced dome fracture of left acetabulum, initial encounter for closed fracture | | |
| S32.482B | Displaced dome fracture of left acetabulum, initial encounter for open fracture | | |
| S32.483A | Displaced dome fracture of unspecified acetabulum, initial encounter for closed fracture | | |
| S32.483B | Displaced dome fracture of unspecified acetabulum, initial encounter for open fracture | | |
| S32.484A | Nondisplaced dome fracture of right acetabulum, initial encounter for closed fracture | | |
| S32.484B | Nondisplaced dome fracture of right acetabulum, initial encounter for open fracture | | |
| S32.485A | Nondisplaced dome fracture of left acetabulum, initial encounter for closed fracture | | |
| S32.485B | Nondisplaced dome fracture of left acetabulum, initial encounter for open fracture | | |
| S32.486A | Nondisplaced dome fracture of unspecified acetabulum, initial encounter for closed fracture | | |
| S32.486B | Nondisplaced dome fracture of unspecified acetabulum, initial encounter for open fracture | | |
| S32.491A | Other specified fracture of right acetabulum, initial encounter for closed fracture | | |
| S32.491B | Other specified fracture of right acetabulum, initial encounter for open fracture | | |
| S32.492A | Other specified fracture of left acetabulum, initial encounter for closed fracture | | |
| S32.492B | Other specified fracture of left acetabulum, initial encounter for open fracture | | |
| S32.499A | Other specified fracture of unspecified acetabulum, initial encounter for closed fracture | | |
| S32.499B | Other specified fracture of unspecified acetabulum, initial encounter for open fracture | | |

© 2019 Vashi AA et al. *JAMA Network Open.*

| | | | |
|---|---|---|---|
| S32.501A | Unspecified fracture of right pubis, initial encounter for closed fracture | | |
| S32.501B | Unspecified fracture of right pubis, initial encounter for open fracture | | |
| S32.502A | Unspecified fracture of left pubis, initial encounter for closed fracture | | |
| S32.502B | Unspecified fracture of left pubis, initial encounter for open fracture | | |
| S32.509A | Unspecified fracture of unspecified pubis, initial encounter for closed fracture | | |
| S32.509B | Unspecified fracture of unspecified pubis, initial encounter for open fracture | | |
| S32.511A | Fracture of superior rim of right pubis, initial encounter for closed fracture | | |
| S32.511B | Fracture of superior rim of right pubis, initial encounter for open fracture | | |
| S32.512A | Fracture of superior rim of left pubis, initial encounter for closed fracture | | |
| S32.512B | Fracture of superior rim of left pubis, initial encounter for open fracture | | |
| S32.519A | Fracture of superior rim of unspecified pubis, initial encounter for closed fracture | | |
| S32.519B | Fracture of superior rim of unspecified pubis, initial encounter for open fracture | | |
| S32.591A | Other specified fracture of right pubis, initial encounter for closed fracture | | |
| S32.591B | Other specified fracture of right pubis, initial encounter for open fracture | | |
| S32.592A | Other specified fracture of left pubis, initial encounter for closed fracture | | |
| S32.592B | Other specified fracture of left pubis, initial encounter for open fracture | | |
| S32.599A | Other specified fracture of unspecified pubis, initial encounter for closed fracture | | |
| S32.599B | Other specified fracture of unspecified pubis, initial encounter for open fracture | | |
| S32.601A | Unspecified fracture of right ischium, initial encounter for closed fracture | | |

© 2019 Vashi AA et al. *JAMA Network Open.*

| | | | |
|---|---|---|---|
| S32.601B | Unspecified fracture of right ischium, initial encounter for open fracture | | |
| S32.602A | Unspecified fracture of left ischium, initial encounter for closed fracture | | |
| S32.602B | Unspecified fracture of left ischium, initial encounter for open fracture | | |
| S32.609A | Unspecified fracture of unspecified ischium, initial encounter for closed fracture | | |
| S32.609B | Unspecified fracture of unspecified ischium, initial encounter for open fracture | | |
| S32.611A | Displaced avulsion fracture of right ischium, initial encounter for closed fracture | | |
| S32.611B | Displaced avulsion fracture of right ischium, initial encounter for open fracture | | |
| S32.612A | Displaced avulsion fracture of left ischium, initial encounter for closed fracture | | |
| S32.612B | Displaced avulsion fracture of left ischium, initial encounter for open fracture | | |
| S32.613A | Displaced avulsion fracture of unspecified ischium, initial encounter for closed fracture | | |
| S32.613B | Displaced avulsion fracture of unspecified ischium, initial encounter for open fracture | | |
| S32.614A | Nondisplaced avulsion fracture of right ischium, initial encounter for closed fracture | | |
| S32.614B | Nondisplaced avulsion fracture of right ischium, initial encounter for open fracture | | |
| S32.615A | Nondisplaced avulsion fracture of left ischium, initial encounter for closed fracture | | |
| S32.615B | Nondisplaced avulsion fracture of left ischium, initial encounter for open fracture | | |
| S32.616A | Nondisplaced avulsion fracture of unspecified ischium, initial encounter for closed fracture | | |
| S32.616B | Nondisplaced avulsion fracture of unspecified ischium, initial encounter for open fracture | | |
| S32.691A | Other specified fracture of right ischium, initial encounter for closed fracture | | |
| S32.691B | Other specified fracture of right ischium, initial encounter for open fracture | | |

© 2019 Vashi AA et al. *JAMA Network Open.*

| | | | |
|---|---|---|---|
| S32.692A | Other specified fracture of left ischium, initial encounter for closed fracture | | |
| S32.692B | Other specified fracture of left ischium, initial encounter for open fracture | | |
| S32.699A | Other specified fracture of unspecified ischium, initial encounter for closed fracture | | |
| S32.699B | Other specified fracture of unspecified ischium, initial encounter for open fracture | | |
| S32.810A | Multiple fractures of pelvis with stable disruption of pelvic ring, initial encounter for closed fracture | | |
| S32.810B | Multiple fractures of pelvis with stable disruption of pelvic ring, initial encounter for open fracture | | |
| S32.811A | Multiple fractures of pelvis with unstable disruption of pelvic ring, initial encounter for closed fracture | | |
| S32.811B | Multiple fractures of pelvis with unstable disruption of pelvic ring, initial encounter for open fracture | | |
| S32.82XA | Multiple fractures of pelvis without disruption of pelvic ring, initial encounter for closed fracture | | |
| S32.82XB | Multiple fractures of pelvis without disruption of pelvic ring, initial encounter for open fracture | | |
| S32.89XA | Fracture of other parts of pelvis, initial encounter for closed fracture | | |
| S32.89XB | Fracture of other parts of pelvis, initial encounter for open fracture | | |
| S32.9XXA | Fracture of unspecified parts of lumbosacral spine and pelvis, initial encounter for closed fracture | | |
| S32.9XXB | Fracture of unspecified parts of lumbosacral spine and pelvis, initial encounter for open fracture | | |
| S34.01XA | Concussion and edema of lumbar spinal cord, initial encounter | | |
| S34.02XA | Concussion and edema of sacral spinal cord, initial encounter | | |
| S34.101A | Unspecified injury to L1 level of lumbar spinal cord, initial encounter | | |
| S34.102A | Unspecified injury to L2 level of lumbar spinal cord, initial encounter | | |
| S34.103A | Unspecified injury to L3 level of lumbar spinal cord, initial encounter | | |

© 2019 Vashi AA et al. *JAMA Network Open.*

| | | | |
|---|---|---|---|
| S34.104A | Unspecified injury to L4 level of lumbar spinal cord, initial encounter | | |
| S34.105A | Unspecified injury to L5 level of lumbar spinal cord, initial encounter | | |
| S34.109A | Unspecified injury to unspecified level of lumbar spinal cord, initial encounter | | |
| S34.111A | Complete lesion of L1 level of lumbar spinal cord, initial encounter | | |
| S34.112A | Complete lesion of L2 level of lumbar spinal cord, initial encounter | | |
| S34.113A | Complete lesion of L3 level of lumbar spinal cord, initial encounter | | |
| S34.114A | Complete lesion of L4 level of lumbar spinal cord, initial encounter | | |
| S34.115A | Complete lesion of L5 level of lumbar spinal cord, initial encounter | | |
| S34.119A | Complete lesion of unspecified level of lumbar spinal cord, initial encounter | | |
| S34.121A | Incomplete lesion of L1 level of lumbar spinal cord, initial encounter | | |
| S34.122A | Incomplete lesion of L2 level of lumbar spinal cord, initial encounter | | |
| S34.123A | Incomplete lesion of L3 level of lumbar spinal cord, initial encounter | | |
| S34.124A | Incomplete lesion of L4 level of lumbar spinal cord, initial encounter | | |
| S34.125A | Incomplete lesion of L5 level of lumbar spinal cord, initial encounter | | |
| S34.129A | Incomplete lesion of unspecified level of lumbar spinal cord, initial encounter | | |
| S34.131A | Complete lesion of sacral spinal cord, initial encounter | | |
| S34.132A | Incomplete lesion of sacral spinal cord, initial encounter | | |
| S34.139A | Unspecified injury to sacral spinal cord, initial encounter | | |
| S34.21XA | Injury of nerve root of lumbar spine, initial encounter | | |
| S34.22XA | Injury of nerve root of sacral spine, initial encounter | | |
| S34.3XXA | Injury of cauda equina, initial encounter | | |
| S34.4XXA | Injury of lumbosacral plexus, initial encounter | | |

© 2019 Vashi AA et al. *JAMA Network Open.*

| | | | |
|---|---|---|---|
| S34.5XXA | Injury of lumbar, sacral and pelvic sympathetic nerves, initial encounter | | |
| S34.6XXA | Injury of peripheral nerve(s) at abdomen, lower back and pelvis level, initial encounter | | |
| S34.8XXA | Injury of other nerves at abdomen, lower back and pelvis level, initial encounter | | |
| S34.9XXA | Injury of unspecified nerves at abdomen, lower back and pelvis level, initial encounter | | |
| S35.00XA | Unspecified injury of abdominal aorta, initial encounter | | |
| S35.01XA | Minor laceration of abdominal aorta, initial encounter | | |
| S35.02XA | Major laceration of abdominal aorta, initial encounter | | |
| S35.09XA | Other injury of abdominal aorta, initial encounter | | |
| S35.10XA | Unspecified injury of inferior vena cava, initial encounter | | |
| S35.11XA | Minor laceration of inferior vena cava, initial encounter | | |
| S35.12XA | Major laceration of inferior vena cava, initial encounter | | |
| S35.19XA | Other injury of inferior vena cava, initial encounter | | |
| S35.211A | Minor laceration of celiac artery, initial encounter | | |
| S35.212A | Major laceration of celiac artery, initial encounter | | |
| S35.218A | Other injury of celiac artery, initial encounter | | |
| S35.219A | Unspecified injury of celiac artery, initial encounter | | |
| S35.221A | Minor laceration of superior mesenteric artery, initial encounter | | |
| S35.222A | Major laceration of superior mesenteric artery, initial encounter | | |
| S35.228A | Other injury of superior mesenteric artery, initial encounter | | |
| S35.229A | Unspecified injury of superior mesenteric artery, initial encounter | | |
| S35.231A | Minor laceration of inferior mesenteric artery, initial encounter | | |
| S35.232A | Major laceration of inferior mesenteric artery, initial encounter | | |
| S35.238A | Other injury of inferior mesenteric artery, initial encounter | | |
| S35.239A | Unspecified injury of inferior mesenteric artery, initial encounter | | |
| S35.291A | Minor laceration of branches of celiac and mesenteric artery, initial encounter | | |

© 2019 Vashi AA et al. *JAMA Network Open.*

| | | | |
|---|---|---|---|
| S35.292A | Major laceration of branches of celiac and mesenteric artery, initial encounter | | |
| S35.298A | Other injury of branches of celiac and mesenteric artery, initial encounter | | |
| S35.299A | Unspecified injury of branches of celiac and mesenteric artery, initial encounter | | |
| S35.311A | Laceration of portal vein, initial encounter | | |
| S35.318A | Other specified injury of portal vein, initial encounter | | |
| S35.319A | Unspecified injury of portal vein, initial encounter | | |
| S35.321A | Laceration of splenic vein, initial encounter | | |
| S35.328A | Other specified injury of splenic vein, initial encounter | | |
| S35.329A | Unspecified injury of splenic vein, initial encounter | | |
| S35.331A | Laceration of superior mesenteric vein, initial encounter | | |
| S35.338A | Other specified injury of superior mesenteric vein, initial encounter | | |
| S35.339A | Unspecified injury of superior mesenteric vein, initial encounter | | |
| S35.341A | Laceration of inferior mesenteric vein, initial encounter | | |
| S35.348A | Other specified injury of inferior mesenteric vein, initial encounter | | |
| S35.349A | Unspecified injury of inferior mesenteric vein, initial encounter | | |
| S35.401A | Unspecified injury of right renal artery, initial encounter | | |
| S35.402A | Unspecified injury of left renal artery, initial encounter | | |
| S35.403A | Unspecified injury of unspecified renal artery, initial encounter | | |
| S35.404A | Unspecified injury of right renal vein, initial encounter | | |
| S35.405A | Unspecified injury of left renal vein, initial encounter | | |
| S35.406A | Unspecified injury of unspecified renal vein, initial encounter | | |
| S35.411A | Laceration of right renal artery, initial encounter | | |
| S35.412A | Laceration of left renal artery, initial encounter | | |
| S35.413A | Laceration of unspecified renal artery, initial encounter | | |
| S35.414A | Laceration of right renal vein, initial encounter | | |
| S35.415A | Laceration of left renal vein, initial encounter | | |
| S35.416A | Laceration of unspecified renal vein, initial encounter | | |

© 2019 Vashi AA et al. *JAMA Network Open.*

| | | | |
|---|---|---|---|
| S35.491A | Other specified injury of right renal artery, initial encounter | | |
| S35.492A | Other specified injury of left renal artery, initial encounter | | |
| S35.493A | Other specified injury of unspecified renal artery, initial encounter | | |
| S35.494A | Other specified injury of right renal vein, initial encounter | | |
| S35.495A | Other specified injury of left renal vein, initial encounter | | |
| S35.496A | Other specified injury of unspecified renal vein, initial encounter | | |
| S35.50XA | Injury of unspecified iliac blood vessel(s), initial encounter | | |
| S35.511A | Injury of right iliac artery, initial encounter | | |
| S35.512A | Injury of left iliac artery, initial encounter | | |
| S35.513A | Injury of unspecified iliac artery, initial encounter | | |
| S35.514A | Injury of right iliac vein, initial encounter | | |
| S35.515A | Injury of left iliac vein, initial encounter | | |
| S35.516A | Injury of unspecified iliac vein, initial encounter | | |
| S35.531A | Injury of right uterine artery, initial encounter | | |
| S35.532A | Injury of left uterine artery, initial encounter | | |
| S35.533A | Injury of unspecified uterine artery, initial encounter | | |
| S35.534A | Injury of right uterine vein, initial encounter | | |
| S35.535A | Injury of left uterine vein, initial encounter | | |
| S35.536A | Injury of unspecified uterine vein, initial encounter | | |
| S35.59XA | Injury of other iliac blood vessels, initial encounter | | |
| S35.8X1A | Laceration of other blood vessels at abdomen, lower back and pelvis level, initial encounter | | |
| S35.8X8A | Other specified injury of other blood vessels at abdomen, lower back and pelvis level, initial encounter | | |
| S35.8X9A | Unspecified injury of other blood vessels at abdomen, lower back and pelvis level, initial encounter | | |
| S35.90XA | Unspecified injury of unspecified blood vessel at abdomen, lower back and pelvis level, initial encounter | | |
| S35.91XA | Laceration of unspecified blood vessel at abdomen, lower back and pelvis level, initial encounter | | |
| S35.99XA | Other specified injury of unspecified blood vessel at abdomen, lower back and pelvis level, initial encounter | | |

© 2019 Vashi AA et al. *JAMA Network Open.*

| | | | |
|---|---|---|---|
| S36.00XA | Unspecified injury of spleen, initial encounter | | |
| S36.020A | Minor contusion of spleen, initial encounter | | |
| S36.021A | Major contusion of spleen, initial encounter | | |
| S36.029A | Unspecified contusion of spleen, initial encounter | | |
| S36.030A | Superficial (capsular) laceration of spleen, initial encounter | | |
| S36.031A | Moderate laceration of spleen, initial encounter | | |
| S36.032A | Major laceration of spleen, initial encounter | | |
| S36.039A | Unspecified laceration of spleen, initial encounter | | |
| S36.09XA | Other injury of spleen, initial encounter | | |
| S36.112A | Contusion of liver, initial encounter | | |
| S36.113A | Laceration of liver, unspecified degree, initial encounter | | |
| S36.114A | Minor laceration of liver, initial encounter | | |
| S36.115A | Moderate laceration of liver, initial encounter | | |
| S36.116A | Major laceration of liver, initial encounter | | |
| S36.118A | Other injury of liver, initial encounter | | |
| S36.119A | Unspecified injury of liver, initial encounter | | |
| S36.122A | Contusion of gallbladder, initial encounter | | |
| S36.123A | Laceration of gallbladder, initial encounter | | |
| S36.128A | Other injury of gallbladder, initial encounter | | |
| S36.129A | Unspecified injury of gallbladder, initial encounter | | |
| S36.13XA | Injury of bile duct, initial encounter | | |
| S36.200A | Unspecified injury of head of pancreas, initial encounter | | |
| S36.201A | Unspecified injury of body of pancreas, initial encounter | | |
| S36.202A | Unspecified injury of tail of pancreas, initial encounter | | |
| S36.209A | Unspecified injury of unspecified part of pancreas, initial encounter | | |
| S36.220A | Contusion of head of pancreas, initial encounter | | |
| S36.221A | Contusion of body of pancreas, initial encounter | | |
| S36.222A | Contusion of tail of pancreas, initial encounter | | |
| S36.229A | Contusion of unspecified part of pancreas, initial encounter | | |
| S36.230A | Laceration of head of pancreas, unspecified degree, initial encounter | | |
| S36.231A | Laceration of body of pancreas, unspecified degree, initial encounter | | |

© 2019 Vashi AA et al. *JAMA Network Open.*

| | | | |
|---|---|---|---|
| S36.232A | Laceration of tail of pancreas, unspecified degree, initial encounter | | |
| S36.239A | Laceration of unspecified part of pancreas, unspecified degree, initial encounter | | |
| S36.240A | Minor laceration of head of pancreas, initial encounter | | |
| S36.241A | Minor laceration of body of pancreas, initial encounter | | |
| S36.242A | Minor laceration of tail of pancreas, initial encounter | | |
| S36.249A | Minor laceration of unspecified part of pancreas, initial encounter | | |
| S36.250A | Moderate laceration of head of pancreas, initial encounter | | |
| S36.251A | Moderate laceration of body of pancreas, initial encounter | | |
| S36.252A | Moderate laceration of tail of pancreas, initial encounter | | |
| S36.259A | Moderate laceration of unspecified part of pancreas, initial encounter | | |
| S36.260A | Major laceration of head of pancreas, initial encounter | | |
| S36.261A | Major laceration of body of pancreas, initial encounter | | |
| S36.262A | Major laceration of tail of pancreas, initial encounter | | |
| S36.269A | Major laceration of unspecified part of pancreas, initial encounter | | |
| S36.290A | Other injury of head of pancreas, initial encounter | | |
| S36.291A | Other injury of body of pancreas, initial encounter | | |
| S36.292A | Other injury of tail of pancreas, initial encounter | | |
| S36.299A | Other injury of unspecified part of pancreas, initial encounter | | |
| S36.30XA | Unspecified injury of stomach, initial encounter | | |
| S36.32XA | Contusion of stomach, initial encounter | | |
| S36.33XA | Laceration of stomach, initial encounter | | |
| S36.39XA | Other injury of stomach, initial encounter | | |
| S36.400A | Unspecified injury of duodenum, initial encounter | | |
| S36.408A | Unspecified injury of other part of small intestine, initial encounter | | |
| S36.409A | Unspecified injury of unspecified part of small intestine, initial encounter | | |
| S36.410A | Primary blast injury of duodenum, initial encounter | | |

© 2019 Vashi AA et al. *JAMA Network Open.*

| | | | |
|---|---|---|---|
| S36.418A | Primary blast injury of other part of small intestine, initial encounter | | |
| S36.419A | Primary blast injury of unspecified part of small intestine, initial encounter | | |
| S36.420A | Contusion of duodenum, initial encounter | | |
| S36.428A | Contusion of other part of small intestine, initial encounter | | |
| S36.429A | Contusion of unspecified part of small intestine, initial encounter | | |
| S36.430A | Laceration of duodenum, initial encounter | | |
| S36.438A | Laceration of other part of small intestine, initial encounter | | |
| S36.439A | Laceration of unspecified part of small intestine, initial encounter | | |
| S36.490A | Other injury of duodenum, initial encounter | | |
| S36.498A | Other injury of other part of small intestine, initial encounter | | |
| S36.499A | Other injury of unspecified part of small intestine, initial encounter | | |
| S36.500A | Unspecified injury of ascending [right] colon, initial encounter | | |
| S36.501A | Unspecified injury of transverse colon, initial encounter | | |
| S36.502A | Unspecified injury of descending [left] colon, initial encounter | | |
| S36.503A | Unspecified injury of sigmoid colon, initial encounter | | |
| S36.508A | Unspecified injury of other part of colon, initial encounter | | |
| S36.509A | Unspecified injury of unspecified part of colon, initial encounter | | |
| S36.510A | Primary blast injury of ascending [right] colon, initial encounter | | |
| S36.511A | Primary blast injury of transverse colon, initial encounter | | |
| S36.512A | Primary blast injury of descending [left] colon, initial encounter | | |
| S36.513A | Primary blast injury of sigmoid colon, initial encounter | | |
| S36.518A | Primary blast injury of other part of colon, initial encounter | | |
| S36.519A | Primary blast injury of unspecified part of colon, initial encounter | | |

© 2019 Vashi AA et al. *JAMA Network Open.*

| | | | |
|---|---|---|---|
| S36.520A | Contusion of ascending [right] colon, initial encounter | | |
| S36.521A | Contusion of transverse colon, initial encounter | | |
| S36.522A | Contusion of descending [left] colon, initial encounter | | |
| S36.523A | Contusion of sigmoid colon, initial encounter | | |
| S36.528A | Contusion of other part of colon, initial encounter | | |
| S36.529A | Contusion of unspecified part of colon, initial encounter | | |
| S36.530A | Laceration of ascending [right] colon, initial encounter | | |
| S36.531A | Laceration of transverse colon, initial encounter | | |
| S36.532A | Laceration of descending [left] colon, initial encounter | | |
| S36.533A | Laceration of sigmoid colon, initial encounter | | |
| S36.538A | Laceration of other part of colon, initial encounter | | |
| S36.539A | Laceration of unspecified part of colon, initial encounter | | |
| S36.590A | Other injury of ascending [right] colon, initial encounter | | |
| S36.591A | Other injury of transverse colon, initial encounter | | |
| S36.592A | Other injury of descending [left] colon, initial encounter | | |
| S36.593A | Other injury of sigmoid colon, initial encounter | | |
| S36.598A | Other injury of other part of colon, initial encounter | | |
| S36.599A | Other injury of unspecified part of colon, initial encounter | | |
| S36.60XA | Unspecified injury of rectum, initial encounter | | |
| S36.61XA | Primary blast injury of rectum, initial encounter | | |
| S36.62XA | Contusion of rectum, initial encounter | | |
| S36.63XA | Laceration of rectum, initial encounter | | |
| S36.69XA | Other injury of rectum, initial encounter | | |
| S36.81XA | Injury of peritoneum, initial encounter | | |
| S36.892A | Contusion of other intra-abdominal organs, initial encounter | | |
| S36.893A | Laceration of other intra-abdominal organs, initial encounter | | |
| S36.898A | Other injury of other intra-abdominal organs, initial encounter | | |
| S36.899A | Unspecified injury of other intra-abdominal organs, initial encounter | | |
| S36.90XA | Unspecified injury of unspecified intra-abdominal organ, initial encounter | | |
| S36.92XA | Contusion of unspecified intra-abdominal organ, initial encounter | | |

© 2019 Vashi AA et al. *JAMA Network Open.*

| | | | |
|---|---|---|---|
| S36.93XA | Laceration of unspecified intra-abdominal organ, initial encounter | | |
| S36.99XA | Other injury of unspecified intra-abdominal organ, initial encounter | | |
| S37.001A | Unspecified injury of right kidney, initial encounter | | |
| S37.002A | Unspecified injury of left kidney, initial encounter | | |
| S37.009A | Unspecified injury of unspecified kidney, initial encounter | | |
| S37.011A | Minor contusion of right kidney, initial encounter | | |
| S37.012A | Minor contusion of left kidney, initial encounter | | |
| S37.019A | Minor contusion of unspecified kidney, initial encounter | | |
| S37.021A | Major contusion of right kidney, initial encounter | | |
| S37.022A | Major contusion of left kidney, initial encounter | | |
| S37.029A | Major contusion of unspecified kidney, initial encounter | | |
| S37.031A | Laceration of right kidney, unspecified degree, initial encounter | | |
| S37.032A | Laceration of left kidney, unspecified degree, initial encounter | | |
| S37.039A | Laceration of unspecified kidney, unspecified degree, initial encounter | | |
| S37.041A | Minor laceration of right kidney, initial encounter | | |
| S37.042A | Minor laceration of left kidney, initial encounter | | |
| S37.049A | Minor laceration of unspecified kidney, initial encounter | | |
| S37.051A | Moderate laceration of right kidney, initial encounter | | |
| S37.052A | Moderate laceration of left kidney, initial encounter | | |
| S37.059A | Moderate laceration of unspecified kidney, initial encounter | | |
| S37.061A | Major laceration of right kidney, initial encounter | | |
| S37.062A | Major laceration of left kidney, initial encounter | | |
| S37.069A | Major laceration of unspecified kidney, initial encounter | | |
| S37.091A | Other injury of right kidney, initial encounter | | |
| S37.092A | Other injury of left kidney, initial encounter | | |
| S37.099A | Other injury of unspecified kidney, initial encounter | | |
| S37.10XA | Unspecified injury of ureter, initial encounter | | |
| S37.12XA | Contusion of ureter, initial encounter | | |
| S37.13XA | Laceration of ureter, initial encounter | | |
| S37.19XA | Other injury of ureter, initial encounter | | |

© 2019 Vashi AA et al. *JAMA Network Open.*

| | | | |
|---|---|---|---|
| S37.20XA | Unspecified injury of bladder, initial encounter | | |
| S37.22XA | Contusion of bladder, initial encounter | | |
| S37.23XA | Laceration of bladder, initial encounter | | |
| S37.29XA | Other injury of bladder, initial encounter | | |
| S37.30XA | Unspecified injury of urethra, initial encounter | | |
| S37.32XA | Contusion of urethra, initial encounter | | |
| S37.33XA | Laceration of urethra, initial encounter | | |
| S37.39XA | Other injury of urethra, initial encounter | | |
| S37.401A | Unspecified injury of ovary, unilateral, initial encounter | | |
| S37.402A | Unspecified injury of ovary, bilateral, initial encounter | | |
| S37.409A | Unspecified injury of ovary, unspecified, initial encounter | | |
| S37.421A | Contusion of ovary, unilateral, initial encounter | | |
| S37.422A | Contusion of ovary, bilateral, initial encounter | | |
| S37.429A | Contusion of ovary, unspecified, initial encounter | | |
| S37.431A | Laceration of ovary, unilateral, initial encounter | | |
| S37.432A | Laceration of ovary, bilateral, initial encounter | | |
| S37.439A | Laceration of ovary, unspecified, initial encounter | | |
| S37.491A | Other injury of ovary, unilateral, initial encounter | | |
| S37.492A | Other injury of ovary, bilateral, initial encounter | | |
| S37.499A | Other injury of ovary, unspecified, initial encounter | | |
| S37.501A | Unspecified injury of fallopian tube, unilateral, initial encounter | | |
| S37.502A | Unspecified injury of fallopian tube, bilateral, initial encounter | | |
| S37.509A | Unspecified injury of fallopian tube, unspecified, initial encounter | | |
| S37.511A | Primary blast injury of fallopian tube, unilateral, initial encounter | | |
| S37.512A | Primary blast injury of fallopian tube, bilateral, initial encounter | | |
| S37.519A | Primary blast injury of fallopian tube, unspecified, initial encounter | | |
| S37.521A | Contusion of fallopian tube, unilateral, initial encounter | | |
| S37.522A | Contusion of fallopian tube, bilateral, initial encounter | | |
| S37.529A | Contusion of fallopian tube, unspecified, initial encounter | | |
| S37.531A | Laceration of fallopian tube, unilateral, initial encounter | | |

© 2019 Vashi AA et al. *JAMA Network Open.*

| | | | |
|---|---|---|---|
| S37.532A | Laceration of fallopian tube, bilateral, initial encounter | | |
| S37.539A | Laceration of fallopian tube, unspecified, initial encounter | | |
| S37.591A | Other injury of fallopian tube, unilateral, initial encounter | | |
| S37.592A | Other injury of fallopian tube, bilateral, initial encounter | | |
| S37.599A | Other injury of fallopian tube, unspecified, initial encounter | | |
| S37.60XA | Unspecified injury of uterus, initial encounter | | |
| S37.62XA | Contusion of uterus, initial encounter | | |
| S37.63XA | Laceration of uterus, initial encounter | | |
| S37.69XA | Other injury of uterus, initial encounter | | |
| S37.812A | Contusion of adrenal gland, initial encounter | | |
| S37.813A | Laceration of adrenal gland, initial encounter | | |
| S37.818A | Other injury of adrenal gland, initial encounter | | |
| S37.819A | Unspecified injury of adrenal gland, initial encounter | | |
| S37.822A | Contusion of prostate, initial encounter | | |
| S37.823A | Laceration of prostate, initial encounter | | |
| S37.828A | Other injury of prostate, initial encounter | | |
| S37.829A | Unspecified injury of prostate, initial encounter | | |
| S37.892A | Contusion of other urinary and pelvic organ, initial encounter | | |
| S37.893A | Laceration of other urinary and pelvic organ, initial encounter | | |
| S37.898A | Other injury of other urinary and pelvic organ, initial encounter | | |
| S37.899A | Unspecified injury of other urinary and pelvic organ, initial encounter | | |
| S37.90XA | Unspecified injury of unspecified urinary and pelvic organ, initial encounter | | |
| S37.92XA | Contusion of unspecified urinary and pelvic organ, initial encounter | | |
| S37.93XA | Laceration of unspecified urinary and pelvic organ, initial encounter | | |
| S37.99XA | Other injury of unspecified urinary and pelvic organ, initial encounter | | |
| S38.001A | Crushing injury of unspecified external genital organs, male, initial encounter | | |

© 2019 Vashi AA et al. *JAMA Network Open.*

| | | | |
|---|---|---|---|
| S38.002A | Crushing injury of unspecified external genital organs, female, initial encounter | | |
| S38.01XA | Crushing injury of penis, initial encounter | | |
| S38.02XA | Crushing injury of scrotum and testis, initial encounter | | |
| S38.03XA | Crushing injury of vulva, initial encounter | | |
| S38.1XXA | Crushing injury of abdomen, lower back, and pelvis, initial encounter | | |
| S38.211A | Complete traumatic amputation of female external genital organs, initial encounter | | |
| S38.212A | Partial traumatic amputation of female external genital organs, initial encounter | | |
| S38.221A | Complete traumatic amputation of penis, initial encounter | | |
| S38.222A | Partial traumatic amputation of penis, initial encounter | | |
| S38.231A | Complete traumatic amputation of scrotum and testis, initial encounter | | |
| S38.232A | Partial traumatic amputation of scrotum and testis, initial encounter | | |
| S38.3XXA | Transection (partial) of abdomen, initial encounter | | |
| S39.001A | Unspecified injury of muscle, fascia and tendon of abdomen, initial encounter | | |
| S39.002A | Unspecified injury of muscle, fascia and tendon of lower back, initial encounter | | |
| S39.003A | Unspecified injury of muscle, fascia and tendon of pelvis, initial encounter | | |
| S39.021A | Laceration of muscle, fascia and tendon of abdomen, initial encounter | | |
| S39.022A | Laceration of muscle, fascia and tendon of lower back, initial encounter | | |
| S39.023A | Laceration of muscle, fascia and tendon of pelvis, initial encounter | | |
| S39.091A | Other injury of muscle, fascia and tendon of abdomen, initial encounter | | |
| S39.092A | Other injury of muscle, fascia and tendon of lower back, initial encounter | | |
| S39.093A | Other injury of muscle, fascia and tendon of pelvis, initial encounter | | |
| S39.81XA | Other specified injuries of abdomen, initial encounter | | |
| S39.82XA | Other specified injuries of lower back, initial encounter | | |

© 2019 Vashi AA et al. *JAMA Network Open.*

| | | | |
|---|---|---|---|
| S39.83XA | Other specified injuries of pelvis, initial encounter | | |
| S39.840A | Fracture of corpus cavernosum penis, initial encounter | | |
| S39.848A | Other specified injuries of external genitals, initial encounter | | |
| S39.91XA | Unspecified injury of abdomen, initial encounter | | |
| S39.92XA | Unspecified injury of lower back, initial encounter | | |
| S39.93XA | Unspecified injury of pelvis, initial encounter | | |
| S39.94XA | Unspecified injury of external genitals, initial encounter | | |

© 2019 Vashi AA et al. *JAMA Network Open.*

## Included and Excluded ICD-10-CM Diagnosis Subcodes for Acute Angle Closure Glaucoma Condition Group

| **Included ICD10-CM diagnosis subcodes (n=4)** | | **Excluded ICD-10-CM diagnosis subcodes (n=229)** | |
|---|---|---|---|
| **Subcode** | **Subcode description** | **Subcode** | **Subcode description** |
| H40.211 | Acute angle-closure glaucoma, right eye | H40.001 | Preglaucoma, unspecified, right eye |
| H40.212 | Acute angle-closure glaucoma, left eye | H40.002 | Preglaucoma, unspecified, left eye |
| H40.213 | Acute angle-closure glaucoma, bilateral | H40.003 | Preglaucoma, unspecified, bilateral |
| H40.219 | Acute angle-closure glaucoma, unspecified eye | H40.009 | Preglaucoma, unspecified, unspecified eye |
| | | H40.011 | Open angle with borderline findings, low risk, right eye |
| | | H40.012 | Open angle with borderline findings, low risk, left eye |
| | | H40.013 | Open angle with borderline findings, low risk, bilateral |
| | | H40.019 | Open angle with borderline findings, low risk, unspecified eye |
| | | H40.021 | Open angle with borderline findings, high risk, right eye |
| | | H40.022 | Open angle with borderline findings, high risk, left eye |
| | | H40.023 | Open angle with borderline findings, high risk, bilateral |
| | | H40.029 | Open angle with borderline findings, high risk, unspecified eye |
| | | H40.031 | Anatomical narrow angle, right eye |
| | | H40.032 | Anatomical narrow angle, left eye |
| | | H40.033 | Anatomical narrow angle, bilateral |
| | | H40.039 | Anatomical narrow angle, unspecified eye |
| | | H40.041 | Steroid responder, right eye |
| | | H40.042 | Steroid responder, left eye |
| | | H40.043 | Steroid responder, bilateral |
| | | H40.049 | Steroid responder, unspecified eye |
| | | H40.051 | Ocular hypertension, right eye |
| | | H40.052 | Ocular hypertension, left eye |
| | | H40.053 | Ocular hypertension, bilateral |
| | | H40.059 | Ocular hypertension, unspecified eye |
| | | H40.061 | Primary angle closure without glaucoma damage, right eye |
| | | H40.062 | Primary angle closure without glaucoma damage, left eye |
| | | H40.063 | Primary angle closure without glaucoma damage, bilateral |

© 2019 Vashi AA et al. *JAMA Network Open.*

| | | | |
|---|---|---|---|
| | | H40.069 | Primary angle closure without glaucoma damage, unspecified eye |
| | | H40.10X0 | Unspecified open-angle glaucoma, stage unspecified |
| | | H40.10X1 | Unspecified open-angle glaucoma, mild stage |
| | | H40.10X2 | Unspecified open-angle glaucoma, moderate stage |
| | | H40.10X3 | Unspecified open-angle glaucoma, severe stage |
| | | H40.10X4 | Unspecified open-angle glaucoma, indeterminate stage |
| | | H40.11X0 | Primary open-angle glaucoma, stage unspecified |
| | | H40.11X1 | Primary open-angle glaucoma, mild stage |
| | | H40.11X2 | Primary open-angle glaucoma, moderate stage |
| | | H40.11X3 | Primary open-angle glaucoma, severe stage |
| | | H40.11X4 | Primary open-angle glaucoma, indeterminate stage |
| | | H40.1210 | Low-tension glaucoma, right eye, stage unspecified |
| | | H40.1211 | Low-tension glaucoma, right eye, mild stage |
| | | H40.1212 | Low-tension glaucoma, right eye, moderate stage |
| | | H40.1213 | Low-tension glaucoma, right eye, severe stage |
| | | H40.1214 | Low-tension glaucoma, right eye, indeterminate stage |
| | | H40.1220 | Low-tension glaucoma, left eye, stage unspecified |
| | | H40.1221 | Low-tension glaucoma, left eye, mild stage |
| | | H40.1222 | Low-tension glaucoma, left eye, moderate stage |
| | | H40.1223 | Low-tension glaucoma, left eye, severe stage |
| | | H40.1224 | Low-tension glaucoma, left eye, indeterminate stage |
| | | H40.1230 | Low-tension glaucoma, bilateral, stage unspecified |
| | | H40.1231 | Low-tension glaucoma, bilateral, mild stage |
| | | H40.1232 | Low-tension glaucoma, bilateral, moderate stage |
| | | H40.1233 | Low-tension glaucoma, bilateral, severe stage |
| | | H40.1234 | Low-tension glaucoma, bilateral, indeterminate stage |
| | | H40.1290 | Low-tension glaucoma, unspecified eye, stage unspecified |
| | | H40.1291 | Low-tension glaucoma, unspecified eye, mild stage |
| | | H40.1292 | Low-tension glaucoma, unspecified eye, moderate stage |
| | | H40.1293 | Low-tension glaucoma, unspecified eye, severe stage |
| | | H40.1294 | Low-tension glaucoma, unspecified eye, indeterminate stage |

© 2019 Vashi AA et al. *JAMA Network Open.*

| | | | |
|---|---|---|---|
| | | H40.1310 | Pigmentary glaucoma, right eye, stage unspecified |
| | | H40.1311 | Pigmentary glaucoma, right eye, mild stage |
| | | H40.1312 | Pigmentary glaucoma, right eye, moderate stage |
| | | H40.1313 | Pigmentary glaucoma, right eye, severe stage |
| | | H40.1314 | Pigmentary glaucoma, right eye, indeterminate stage |
| | | H40.1320 | Pigmentary glaucoma, left eye, stage unspecified |
| | | H40.1321 | Pigmentary glaucoma, left eye, mild stage |
| | | H40.1322 | Pigmentary glaucoma, left eye, moderate stage |
| | | H40.1323 | Pigmentary glaucoma, left eye, severe stage |
| | | H40.1324 | Pigmentary glaucoma, left eye, indeterminate stage |
| | | H40.1330 | Pigmentary glaucoma, bilateral, stage unspecified |
| | | H40.1331 | Pigmentary glaucoma, bilateral, mild stage |
| | | H40.1332 | Pigmentary glaucoma, bilateral, moderate stage |
| | | H40.1333 | Pigmentary glaucoma, bilateral, severe stage |
| | | H40.1334 | Pigmentary glaucoma, bilateral, indeterminate stage |
| | | H40.1390 | Pigmentary glaucoma, unspecified eye, stage unspecified |
| | | H40.1391 | Pigmentary glaucoma, unspecified eye, mild stage |
| | | H40.1392 | Pigmentary glaucoma, unspecified eye, moderate stage |
| | | H40.1393 | Pigmentary glaucoma, unspecified eye, severe stage |
| | | H40.1394 | Pigmentary glaucoma, unspecified eye, indeterminate stage |
| | | H40.1410 | Capsular glaucoma with pseudoexfoliation of lens, right eye, stage unspecified |
| | | H40.1411 | Capsular glaucoma with pseudoexfoliation of lens, right eye, mild stage |
| | | H40.1412 | Capsular glaucoma with pseudoexfoliation of lens, right eye, moderate stage |
| | | H40.1413 | Capsular glaucoma with pseudoexfoliation of lens, right eye, severe stage |
| | | H40.1414 | Capsular glaucoma with pseudoexfoliation of lens, right eye, indeterminate stage |
| | | H40.1420 | Capsular glaucoma with pseudoexfoliation of lens, left eye, stage unspecified |
| | | H40.1421 | Capsular glaucoma with pseudoexfoliation of lens, left eye, mild stage |

© 2019 Vashi AA et al. *JAMA Network Open.*

| | | | |
|---|---|---|---|
| | | H40.1422 | Capsular glaucoma with pseudoexfoliation of lens, left eye, moderate stage |
| | | H40.1423 | Capsular glaucoma with pseudoexfoliation of lens, left eye, severe stage |
| | | H40.1424 | Capsular glaucoma with pseudoexfoliation of lens, left eye, indeterminate stage |
| | | H40.1430 | Capsular glaucoma with pseudoexfoliation of lens, bilateral, stage unspecified |
| | | H40.1431 | Capsular glaucoma with pseudoexfoliation of lens, bilateral, mild stage |
| | | H40.1432 | Capsular glaucoma with pseudoexfoliation of lens, bilateral, moderate stage |
| | | H40.1433 | Capsular glaucoma with pseudoexfoliation of lens, bilateral, severe stage |
| | | H40.1434 | Capsular glaucoma with pseudoexfoliation of lens, bilateral, indeterminate stage |
| | | H40.1490 | Capsular glaucoma with pseudoexfoliation of lens, unspecified eye, stage unspecified |
| | | H40.1491 | Capsular glaucoma with pseudoexfoliation of lens, unspecified eye, mild stage |
| | | H40.1492 | Capsular glaucoma with pseudoexfoliation of lens, unspecified eye, moderate stage |
| | | H40.1493 | Capsular glaucoma with pseudoexfoliation of lens, unspecified eye, severe stage |
| | | H40.1494 | Capsular glaucoma with pseudoexfoliation of lens, unspecified eye, indeterminate stage |
| | | H40.151 | Residual stage of open-angle glaucoma, right eye |
| | | H40.152 | Residual stage of open-angle glaucoma, left eye |
| | | H40.153 | Residual stage of open-angle glaucoma, bilateral |
| | | H40.159 | Residual stage of open-angle glaucoma, unspecified eye |
| | | H40.20X0 | Unspecified primary angle-closure glaucoma, stage unspecified |
| | | H40.20X1 | Unspecified primary angle-closure glaucoma, mild stage |
| | | H40.20X2 | Unspecified primary angle-closure glaucoma, moderate stage |
| | | H40.20X3 | Unspecified primary angle-closure glaucoma, severe stage |

© 2019 Vashi AA et al. *JAMA Network Open.*

| | | | |
|---|---|---|---|
| | | H40.20X4 | Unspecified primary angle-closure glaucoma, indeterminate stage |
| | | H40.2210 | Chronic angle-closure glaucoma, right eye, stage unspecified |
| | | H40.2211 | Chronic angle-closure glaucoma, right eye, mild stage |
| | | H40.2212 | Chronic angle-closure glaucoma, right eye, moderate stage |
| | | H40.2213 | Chronic angle-closure glaucoma, right eye, severe stage |
| | | H40.2214 | Chronic angle-closure glaucoma, right eye, indeterminate stage |
| | | H40.2220 | Chronic angle-closure glaucoma, left eye, stage unspecified |
| | | H40.2221 | Chronic angle-closure glaucoma, left eye, mild stage |
| | | H40.2222 | Chronic angle-closure glaucoma, left eye, moderate stage |
| | | H40.2223 | Chronic angle-closure glaucoma, left eye, severe stage |
| | | H40.2224 | Chronic angle-closure glaucoma, left eye, indeterminate stage |
| | | H40.2230 | Chronic angle-closure glaucoma, bilateral, stage unspecified |
| | | H40.2231 | Chronic angle-closure glaucoma, bilateral, mild stage |
| | | H40.2232 | Chronic angle-closure glaucoma, bilateral, moderate stage |
| | | H40.2233 | Chronic angle-closure glaucoma, bilateral, severe stage |
| | | H40.2234 | Chronic angle-closure glaucoma, bilateral, indeterminate stage |
| | | H40.2290 | Chronic angle-closure glaucoma, unspecified eye, stage unspecified |
| | | H40.2291 | Chronic angle-closure glaucoma, unspecified eye, mild stage |
| | | H40.2292 | Chronic angle-closure glaucoma, unspecified eye, moderate stage |
| | | H40.2293 | Chronic angle-closure glaucoma, unspecified eye, severe stage |
| | | H40.2294 | Chronic angle-closure glaucoma, unspecified eye, indeterminate stage |
| | | H40.231 | Intermittent angle-closure glaucoma, right eye |
| | | H40.232 | Intermittent angle-closure glaucoma, left eye |

© 2019 Vashi AA et al. *JAMA Network Open.*

| | | | |
|---|---|---|---|
| | | H40.233 | Intermittent angle-closure glaucoma, bilateral |
| | | H40.239 | Intermittent angle-closure glaucoma, unspecified eye |
| | | H40.241 | Residual stage of angle-closure glaucoma, right eye |
| | | H40.242 | Residual stage of angle-closure glaucoma, left eye |
| | | H40.243 | Residual stage of angle-closure glaucoma, bilateral |
| | | H40.249 | Residual stage of angle-closure glaucoma, unspecified eye |
| | | H40.30X0 | Glaucoma secondary to eye trauma, unspecified eye, stage unspecified |
| | | H40.30X1 | Glaucoma secondary to eye trauma, unspecified eye, mild stage |
| | | H40.30X2 | Glaucoma secondary to eye trauma, unspecified eye, moderate stage |
| | | H40.30X3 | Glaucoma secondary to eye trauma, unspecified eye, severe stage |
| | | H40.30X4 | Glaucoma secondary to eye trauma, unspecified eye, indeterminate stage |
| | | H40.31X0 | Glaucoma secondary to eye trauma, right eye, stage unspecified |
| | | H40.31X1 | Glaucoma secondary to eye trauma, right eye, mild stage |
| | | H40.31X2 | Glaucoma secondary to eye trauma, right eye, moderate stage |
| | | H40.31X3 | Glaucoma secondary to eye trauma, right eye, severe stage |
| | | H40.31X4 | Glaucoma secondary to eye trauma, right eye, indeterminate stage |
| | | H40.32X0 | Glaucoma secondary to eye trauma, left eye, stage unspecified |
| | | H40.32X1 | Glaucoma secondary to eye trauma, left eye, mild stage |
| | | H40.32X2 | Glaucoma secondary to eye trauma, left eye, moderate stage |
| | | H40.32X3 | Glaucoma secondary to eye trauma, left eye, severe stage |
| | | H40.32X4 | Glaucoma secondary to eye trauma, left eye, indeterminate stage |
| | | H40.33X0 | Glaucoma secondary to eye trauma, bilateral, stage unspecified |
| | | H40.33X1 | Glaucoma secondary to eye trauma, bilateral, mild stage |

© 2019 Vashi AA et al. *JAMA Network Open.*

| | | | |
|---|---|---|---|
| | | H40.33X2 | Glaucoma secondary to eye trauma, bilateral, moderate stage |
| | | H40.33X3 | Glaucoma secondary to eye trauma, bilateral, severe stage |
| | | H40.33X4 | Glaucoma secondary to eye trauma, bilateral, indeterminate stage |
| | | H40.40X0 | Glaucoma secondary to eye inflammation, unspecified eye, stage unspecified |
| | | H40.40X1 | Glaucoma secondary to eye inflammation, unspecified eye, mild stage |
| | | H40.40X2 | Glaucoma secondary to eye inflammation, unspecified eye, moderate stage |
| | | H40.40X3 | Glaucoma secondary to eye inflammation, unspecified eye, severe stage |
| | | H40.40X4 | Glaucoma secondary to eye inflammation, unspecified eye, indeterminate stage |
| | | H40.41X0 | Glaucoma secondary to eye inflammation, right eye, stage unspecified |
| | | H40.41X1 | Glaucoma secondary to eye inflammation, right eye, mild stage |
| | | H40.41X2 | Glaucoma secondary to eye inflammation, right eye, moderate stage |
| | | H40.41X3 | Glaucoma secondary to eye inflammation, right eye, severe stage |
| | | H40.41X4 | Glaucoma secondary to eye inflammation, right eye, indeterminate stage |
| | | H40.42X0 | Glaucoma secondary to eye inflammation, left eye, stage unspecified |
| | | H40.42X1 | Glaucoma secondary to eye inflammation, left eye, mild stage |
| | | H40.42X2 | Glaucoma secondary to eye inflammation, left eye, moderate stage |
| | | H40.42X3 | Glaucoma secondary to eye inflammation, left eye, severe stage |
| | | H40.42X4 | Glaucoma secondary to eye inflammation, left eye, indeterminate stage |
| | | H40.43X0 | Glaucoma secondary to eye inflammation, bilateral, stage unspecified |

© 2019 Vashi AA et al. *JAMA Network Open.*

| | | | |
|---|---|---|---|
| | | H40.43X1 | Glaucoma secondary to eye inflammation, bilateral, mild stage |
| | | H40.43X2 | Glaucoma secondary to eye inflammation, bilateral, moderate stage |
| | | H40.43X3 | Glaucoma secondary to eye inflammation, bilateral, severe stage |
| | | H40.43X4 | Glaucoma secondary to eye inflammation, bilateral, indeterminate stage |
| | | H40.50X0 | Glaucoma secondary to other eye disorders, unspecified eye, stage unspecified |
| | | H40.50X1 | Glaucoma secondary to other eye disorders, unspecified eye, mild stage |
| | | H40.50X2 | Glaucoma secondary to other eye disorders, unspecified eye, moderate stage |
| | | H40.50X3 | Glaucoma secondary to other eye disorders, unspecified eye, severe stage |
| | | H40.50X4 | Glaucoma secondary to other eye disorders, unspecified eye, indeterminate stage |
| | | H40.51X0 | Glaucoma secondary to other eye disorders, right eye, stage unspecified |
| | | H40.51X1 | Glaucoma secondary to other eye disorders, right eye, mild stage |
| | | H40.51X2 | Glaucoma secondary to other eye disorders, right eye, moderate stage |
| | | H40.51X3 | Glaucoma secondary to other eye disorders, right eye, severe stage |
| | | H40.51X4 | Glaucoma secondary to other eye disorders, right eye, indeterminate stage |
| | | H40.52X0 | Glaucoma secondary to other eye disorders, left eye, stage unspecified |
| | | H40.52X1 | Glaucoma secondary to other eye disorders, left eye, mild stage |
| | | H40.52X2 | Glaucoma secondary to other eye disorders, left eye, moderate stage |
| | | H40.52X3 | Glaucoma secondary to other eye disorders, left eye, severe stage |
| | | H40.52X4 | Glaucoma secondary to other eye disorders, left eye, indeterminate stage |

© 2019 Vashi AA et al. *JAMA Network Open.*

| | | | |
|---|---|---|---|
| | | H40.53X0 | Glaucoma secondary to other eye disorders, bilateral, stage unspecified |
| | | H40.53X1 | Glaucoma secondary to other eye disorders, bilateral, mild stage |
| | | H40.53X2 | Glaucoma secondary to other eye disorders, bilateral, moderate stage |
| | | H40.53X3 | Glaucoma secondary to other eye disorders, bilateral, severe stage |
| | | H40.53X4 | Glaucoma secondary to other eye disorders, bilateral, indeterminate stage |
| | | H40.60X0 | Glaucoma secondary to drugs, unspecified eye, stage unspecified |
| | | H40.60X1 | Glaucoma secondary to drugs, unspecified eye, mild stage |
| | | H40.60X2 | Glaucoma secondary to drugs, unspecified eye, moderate stage |
| | | H40.60X3 | Glaucoma secondary to drugs, unspecified eye, severe stage |
| | | H40.60X4 | Glaucoma secondary to drugs, unspecified eye, indeterminate stage |
| | | H40.61X0 | Glaucoma secondary to drugs, right eye, stage unspecified |
| | | H40.61X1 | Glaucoma secondary to drugs, right eye, mild stage |
| | | H40.61X2 | Glaucoma secondary to drugs, right eye, moderate stage |
| | | H40.61X3 | Glaucoma secondary to drugs, right eye, severe stage |
| | | H40.61X4 | Glaucoma secondary to drugs, right eye, indeterminate stage |
| | | H40.62X0 | Glaucoma secondary to drugs, left eye, stage unspecified |
| | | H40.62X1 | Glaucoma secondary to drugs, left eye, mild stage |
| | | H40.62X2 | Glaucoma secondary to drugs, left eye, moderate stage |
| | | H40.62X3 | Glaucoma secondary to drugs, left eye, severe stage |
| | | H40.62X4 | Glaucoma secondary to drugs, left eye, indeterminate stage |
| | | H40.63X0 | Glaucoma secondary to drugs, bilateral, stage unspecified |
| | | H40.63X1 | Glaucoma secondary to drugs, bilateral, mild stage |
| | | H40.63X2 | Glaucoma secondary to drugs, bilateral, moderate stage |
| | | H40.63X3 | Glaucoma secondary to drugs, bilateral, severe stage |

© 2019 Vashi AA et al. *JAMA Network Open.*

| | | | |
|---|---|---|---|
| | | H40.63X4 | Glaucoma secondary to drugs, bilateral, indeterminate stage |
| | | H40.811 | Glaucoma with increased episcleral venous pressure, right eye |
| | | H40.812 | Glaucoma with increased episcleral venous pressure, left eye |
| | | H40.813 | Glaucoma with increased episcleral venous pressure, bilateral |
| | | H40.819 | Glaucoma with increased episcleral venous pressure, unspecified eye |
| | | H40.821 | Hypersecretion glaucoma, right eye |
| | | H40.822 | Hypersecretion glaucoma, left eye |
| | | H40.823 | Hypersecretion glaucoma, bilateral |
| | | H40.829 | Hypersecretion glaucoma, unspecified eye |
| | | H40.831 | Aqueous misdirection, right eye |
| | | H40.832 | Aqueous misdirection, left eye |
| | | H40.833 | Aqueous misdirection, bilateral |
| | | H40.839 | Aqueous misdirection, unspecified eye |
| | | H40.89 | Other specified glaucoma |
| | | H40.9 | Unspecified glaucoma |

© 2019 Vashi AA et al. *JAMA Network Open.*

## Included and Excluded ICD-10-CM Diagnosis Subcodes for Acute Appendicitis Condition Group

| Included ICD-10-CM diagnosis subcodes (n=4) | | Excluded ICD-10-CM diagnosis subcodes (n=0) | |
|---|---|---|---|
| **Subcode** | **Subcode description** | **Subcode** | **Subcode description** |
| K35.2 | Acute appendicitis with generalized peritonitis | | |
| K35.3 | Acute appendicitis with localized peritonitis | | |
| K35.80 | Unspecified acute appendicitis | | |
| K35.89 | Other acute appendicitis | | |

## Included and Excluded ICD-10- CM Diagnosis Subcodes for Acute Pancreatitis Condition Group

| Included ICD-10-CM diagnosis subcodes (n=6) | | Excluded ICD-10-CM diagnosis subcodes (n=0) | |
|---|---|---|---|
| **Subcode** | **Subcode description** | **Subcode** | **Subcode description** |
| K85.0 | Idiopathic acute pancreatitis | | |
| K85.1 | Biliary acute pancreatitis | | |
| K85.2 | Alcohol induced acute pancreatitis | | |
| K85.3 | Drug induced acute pancreatitis | | |
| K85.8 | Other acute pancreatitis | | |
| K85.9 | Acute pancreatitis, unspecified | | |

## Included and Excluded ICD-10- CM Diagnosis Subcodes for Acute Respiratory Distress Syndrome Condition Group

| Included ICD-10-CM diagnosis subcodes (n=1) | | Excluded ICD-10-CM diagnosis subcodes (n=0) | |
|---|---|---|---|
| **Subcode** | **Subcode description** | **Subcode** | **Subcode description** |
| J80 | Acute respiratory distress syndrome | | |

© 2019 Vashi AA et al. *JAMA Network Open.*

## Included and Excluded ICD-10-CM Diagnosis Subcodes for Alcohol Withdrawal Condition Group

| Included ICD-10-CM diagnosis subcodes (n=4) | | Excluded ICD-10-CM diagnosis subcodes (n=1) | |
|---|---|---|---|
| **Subcode** | **Subcode description** | **Subcode** | **Subcode description** |
| F10.230 | Alcohol dependence with withdrawal, uncomplicated | F10.24 | Alcohol dependence with alcohol-induced mood disorder |
| F10.231 | Alcohol dependence with withdrawal delirium | | |
| F10.232 | Alcohol dependence with withdrawal with perceptual disturbance | | |
| F10.239 | Alcohol dependence with withdrawal, unspecified | | |

## Included and Excluded ICD-10-CM Diagnosis Subcodes for Anaphylaxis Condition Group

| Included ICD-10-CM diagnosis subcodes (n=11) | | Excluded ICD-10-CM diagnosis subcodes (n=6) | |
|---|---|---|---|
| **Subcode** | **Subcode description** | **Subcode** | **Subcode description** |
| T78.00XA | Anaphylactic reaction due to unspecified food, initial encounter | T78.1XXA | Other adverse food reactions, not elsewhere classified, initial encounter |
| T78.01XA | Anaphylactic reaction due to peanuts, initial encounter | T78.3XXA | Angioneurotic edema, initial encounter |
| T78.02XA | Anaphylactic reaction due to shellfish (crustaceans), initial encounter | T78.40XA | Allergy, unspecified, initial encounter |
| T78.03XA | Anaphylactic reaction due to other fish, initial encounter | T78.41XA | Arthus phenomenon, initial encounter |
| T78.04XA | Anaphylactic reaction due to fruits and vegetables, initial encounter | T78.49XA | Other allergy, initial encounter |
| T78.05XA | Anaphylactic reaction due to tree nuts and seeds, initial encounter | T78.8XXA | Other adverse effects, not elsewhere classified, initial encounter |
| T78.06XA | Anaphylactic reaction due to food additives, initial encounter | | |
| T78.07XA | Anaphylactic reaction due to milk and dairy products, initial encounter | | |
| T78.08XA | Anaphylactic reaction due to eggs, initial encounter | | |
| T78.09XA | Anaphylactic reaction due to other food products, initial encounter | | |
| T78.2XXA | Anaphylactic shock, unspecified, initial encounter | | |

© 2019 Vashi AA et al. *JAMA Network Open.*

## Included and Excluded ICD-10-CM Diagnosis Subcodes for Angina and Other Acute Ischemic Heart Disease Condition Group

| Included ICD-10-CM diagnosis subcodes (n=8) | | Excluded ICD-10-CM diagnosis subcodes (n=0) | |
|---|---|---|---|
| **Subcode** | **Subcode description** | **Subcode** | **Subcode description** |
| I20.0 | Unstable angina | | |
| I20.1 | Angina pectoris with documented spasm | | |
| I20.8 | Other forms of angina pectoris | | |
| I20.9 | Angina pectoris, unspecified | | |
| I24.0 | Acute coronary thrombosis not resulting in myocardial infarction | | |
| I24.1 | Dressler's syndrome | | |
| I24.8 | Other forms of acute ischemic heart disease | | |
| I24.9 | Acute ischemic heart disease, unspecified | | |

© 2019 Vashi AA et al. *JAMA Network Open.*

## Included and Excluded ICD-10-CM Diagnosis Subcodes for Aortic Aneurysm and Dissection Condition Group

| Included ICD-10-CM diagnosis subcodes (n=12) | | Excluded ICD-10-CM diagnosis subcodes (n=0) | |
|---|---|---|---|
| **Subcode** | **Subcode description** | **Subcode** | **Subcode description** |
| I71.00 | Dissection of unspecified site of aorta | | |
| I71.01 | Dissection of thoracic aorta | | |
| I71.02 | Dissection of abdominal aorta | | |
| I71.03 | Dissection of thoracoabdominal aorta | | |
| I71.1 | Thoracic aortic aneurysm, ruptured | | |
| I71.2 | Thoracic aortic aneurysm, without rupture | | |
| I71.3 | Abdominal aortic aneurysm, ruptured | | |
| I71.4 | Abdominal aortic aneurysm, without rupture | | |
| I71.5 | Thoracoabdominal aortic aneurysm, ruptured | | |
| I71.6 | Thoracoabdominal aortic aneurysm, without rupture | | |
| I71.8 | Aortic aneurysm of unspecified site, ruptured | | |
| I71.9 | Aortic aneurysm of unspecified site, without rupture | | |

© 2019 Vashi AA et al. *JAMA Network Open.*

## Included and Excluded ICD-10-CM Diagnosis Subcodes for Arterial Embolism and Thrombosis Condition Group

| Included ICD-10-CM diagnosis subcodes (n=12) | | Excluded ICD-10-CM diagnosis subcodes (n=0) | |
|---|---|---|---|
| **Subcode** | **Subcode description** | **Subcode** | **Subcode description** |
| I74.01 | Saddle embolus of abdominal aorta | | |
| I74.09 | Other arterial embolism and thrombosis of abdominal aorta | | |
| I74.10 | Embolism and thrombosis of unspecified parts of aorta | | |
| I74.11 | Embolism and thrombosis of thoracic aorta | | |
| I74.19 | Embolism and thrombosis of other parts of aorta | | |
| I74.2 | Embolism and thrombosis of arteries of the upper extremities | | |
| I74.3 | Embolism and thrombosis of arteries of the lower extremities | | |
| I74.4 | Embolism and thrombosis of arteries of extremities, unspecified | | |
| I74.5 | Embolism and thrombosis of iliac artery | | |
| I74.8 | Embolism and thrombosis of other arteries | | |
| I74.9 | Embolism and thrombosis of unspecified artery | | |
| I76 | Septic arterial embolism | | |

© 2019 Vashi AA et al. *JAMA Network Open.*

## Included and Excluded ICD-10-CM Diagnosis Subcodes for Asthma Condition Group

| Included ICD-10-CM diagnosis subcodes (n=15) | | Excluded ICD-10-CM diagnosis subcodes (n=3) | |
|---|---|---|---|
| **Subcode** | **Subcode description** | **Subcode** | **Subcode description** |
| J45.21 | Mild intermittent asthma with (acute) exacerbation | J45.20 | Mild intermittent asthma, uncomplicated |
| J45.22 | Mild intermittent asthma with status asthmaticus | J45.30 | Mild persistent asthma, uncomplicated |
| J45.31 | Mild persistent asthma with (acute) exacerbation | J45.991 | Cough variant asthma |
| J45.32 | Mild persistent asthma with status asthmaticus | | |
| J45.40 | Moderate persistent asthma, uncomplicated | | |
| J45.41 | Moderate persistent asthma with (acute) exacerbation | | |
| J45.42 | Moderate persistent asthma with status asthmaticus | | |
| J45.50 | Severe persistent asthma, uncomplicated | | |
| J45.51 | Severe persistent asthma with (acute) exacerbation | | |
| J45.52 | Severe persistent asthma with status asthmaticus | | |
| J45.901 | Unspecified asthma with (acute) exacerbation | | |
| J45.902 | Unspecified asthma with status asthmaticus | | |
| J45.909 | Unspecified asthma, uncomplicated | | |
| J45.990 | Exercise induced bronchospasm | | |
| J45.998 | Other asthma | | |

© 2019 Vashi AA et al. *JAMA Network Open.*

## Included and Excluded ICD-10-CM Diagnosis Subcodes for Cardiac Arrest and Severe Arrhythmias Condition Group

| Included ICD-10-CM diagnosis subcodes (n=6) | | Excluded ICD-10-CM diagnosis subcodes (n=0) | |
|---|---|---|---|
| **Subcode** | **Subcode description** | **Subcode** | **Subcode description** |
| I46.2 | Cardiac arrest due to underlying cardiac condition | | |
| I46.8 | Cardiac arrest due to other underlying condition | | |
| I46.9 | Cardiac arrest, cause unspecified | | |
| I47.2 | Ventricular tachycardia | | |
| I49.01 | Ventricular fibrillation | | |
| I49.02 | Ventricular flutter | | |

© 2019 Vashi AA et al. *JAMA Network Open.*

## Included and Excluded ICD-10-CM Diagnosis Subcodes for Cerebral Infarction Condition Group

| Included ICD-10-CM diagnosis subcodes (n=72) | | Excluded ICD-10-CM diagnosis subcodes (n=0) | |
|---|---|---|---|
| **Subcode** | **Subcode description** | **Subcode** | **Subcode description** |
| I63.00 | Cerebral infarction due to thrombosis of unspecified precerebral artery | | |
| I63.011 | Cerebral infarction due to thrombosis of right vertebral artery | | |
| I63.012 | Cerebral infarction due to thrombosis of left vertebral artery | | |
| I63.019 | Cerebral infarction due to thrombosis of unspecified vertebral artery | | |
| I63.02 | Cerebral infarction due to thrombosis of basilar artery | | |
| I63.031 | Cerebral infarction due to thrombosis of right carotid artery | | |
| I63.032 | Cerebral infarction due to thrombosis of left carotid artery | | |
| I63.039 | Cerebral infarction due to thrombosis of unspecified carotid artery | | |
| I63.09 | Cerebral infarction due to thrombosis of other precerebral artery | | |
| I63.10 | Cerebral infarction due to embolism of unspecified precerebral artery | | |
| I63.111 | Cerebral infarction due to embolism of right vertebral artery | | |
| I63.112 | Cerebral infarction due to embolism of left vertebral artery | | |
| I63.119 | Cerebral infarction due to embolism of unspecified vertebral artery | | |
| I63.12 | Cerebral infarction due to embolism of basilar artery | | |
| I63.131 | Cerebral infarction due to embolism of right carotid artery | | |
| I63.132 | Cerebral infarction due to embolism of left carotid artery | | |
| I63.139 | Cerebral infarction due to embolism of unspecified carotid artery | | |
| I63.19 | Cerebral infarction due to embolism of other precerebral artery | | |
| I63.20 | Cerebral infarction due to unspecified occlusion or stenosis of unspecified precerebral arteries | | |
| I63.211 | Cerebral infarction due to unspecified occlusion or stenosis of right vertebral arteries | | |

© 2019 Vashi AA et al. *JAMA Network Open.*

| | | | |
|---|---|---|---|
| I63.212 | Cerebral infarction due to unspecified occlusion or stenosis of left vertebral arteries | | |
| I63.219 | Cerebral infarction due to unspecified occlusion or stenosis of unspecified vertebral arteries | | |
| I63.22 | Cerebral infarction due to unspecified occlusion or stenosis of basilar arteries | | |
| I63.231 | Cerebral infarction due to unspecified occlusion or stenosis of right carotid arteries | | |
| I63.232 | Cerebral infarction due to unspecified occlusion or stenosis of left carotid arteries | | |
| I63.239 | Cerebral infarction due to unspecified occlusion or stenosis of unspecified carotid arteries | | |
| I63.29 | Cerebral infarction due to unspecified occlusion or stenosis of other precerebral arteries | | |
| I63.30 | Cerebral infarction due to thrombosis of unspecified cerebral artery | | |
| I63.311 | Cerebral infarction due to thrombosis of right middle cerebral artery | | |
| I63.312 | Cerebral infarction due to thrombosis of left middle cerebral artery | | |
| I63.319 | Cerebral infarction due to thrombosis of unspecified middle cerebral artery | | |
| I63.321 | Cerebral infarction due to thrombosis of right anterior cerebral artery | | |
| I63.322 | Cerebral infarction due to thrombosis of left anterior cerebral artery | | |
| I63.329 | Cerebral infarction due to thrombosis of unspecified anterior cerebral artery | | |
| I63.331 | Cerebral infarction due to thrombosis of right posterior cerebral artery | | |
| I63.332 | Cerebral infarction due to thrombosis of left posterior cerebral artery | | |
| I63.339 | Cerebral infarction due to thrombosis of unspecified posterior cerebral artery | | |
| I63.341 | Cerebral infarction due to thrombosis of right cerebellar artery | | |
| I63.342 | Cerebral infarction due to thrombosis of left cerebellar artery | | |

© 2019 Vashi AA et al. *JAMA Network Open.*

| | | | |
|---|---|---|---|
| I63.349 | Cerebral infarction due to thrombosis of unspecified cerebellar artery | | |
| I63.39 | Cerebral infarction due to thrombosis of other cerebral artery | | |
| I63.40 | Cerebral infarction due to embolism of unspecified cerebral artery | | |
| I63.411 | Cerebral infarction due to embolism of right middle cerebral artery | | |
| I63.412 | Cerebral infarction due to embolism of left middle cerebral artery | | |
| I63.419 | Cerebral infarction due to embolism of unspecified middle cerebral artery | | |
| I63.421 | Cerebral infarction due to embolism of right anterior cerebral artery | | |
| I63.422 | Cerebral infarction due to embolism of left anterior cerebral artery | | |
| I63.429 | Cerebral infarction due to embolism of unspecified anterior cerebral artery | | |
| I63.431 | Cerebral infarction due to embolism of right posterior cerebral artery | | |
| I63.432 | Cerebral infarction due to embolism of left posterior cerebral artery | | |
| I63.439 | Cerebral infarction due to embolism of unspecified posterior cerebral artery | | |
| I63.441 | Cerebral infarction due to embolism of right cerebellar artery | | |
| I63.442 | Cerebral infarction due to embolism of left cerebellar artery | | |
| I63.449 | Cerebral infarction due to embolism of unspecified cerebellar artery | | |
| I63.49 | Cerebral infarction due to embolism of other cerebral artery | | |
| I63.50 | Cerebral infarction due to unspecified occlusion or stenosis of unspecified cerebral artery | | |
| I63.511 | Cerebral infarction due to unspecified occlusion or stenosis of right middle cerebral artery | | |
| I63.512 | Cerebral infarction due to unspecified occlusion or stenosis of left middle cerebral artery | | |

© 2019 Vashi AA et al. *JAMA Network Open.*

| | | | |
|---|---|---|---|
| I63.519 | Cerebral infarction due to unspecified occlusion or stenosis of unspecified middle cerebral artery | | |
| I63.521 | Cerebral infarction due to unspecified occlusion or stenosis of right anterior cerebral artery | | |
| I63.522 | Cerebral infarction due to unspecified occlusion or stenosis of left anterior cerebral artery | | |
| I63.529 | Cerebral infarction due to unspecified occlusion or stenosis of unspecified anterior cerebral artery | | |
| I63.531 | Cerebral infarction due to unspecified occlusion or stenosis of right posterior cerebral artery | | |
| I63.532 | Cerebral infarction due to unspecified occlusion or stenosis of left posterior cerebral artery | | |
| I63.539 | Cerebral infarction due to unspecified occlusion or stenosis of unspecified posterior cerebral artery | | |
| I63.541 | Cerebral infarction due to unspecified occlusion or stenosis of right cerebellar artery | | |
| I63.542 | Cerebral infarction due to unspecified occlusion or stenosis of left cerebellar artery | | |
| I63.549 | Cerebral infarction due to unspecified occlusion or stenosis of unspecified cerebellar artery | | |
| I63.59 | Cerebral infarction due to unspecified occlusion or stenosis of other cerebral artery | | |
| I63.6 | Cerebral infarction due to cerebral venous thrombosis, nonpyogenic | | |
| I63.8 | Other cerebral infarction | | |
| I63.9 | Cerebral infarction, unspecified | | |

© 2019 Vashi AA et al. *JAMA Network Open.*

## Included and Excluded ICD-10-CM Diagnosis Subcodes for Cholecystitis and Perforation of the Gallbladder Condition Group

| Included ICD-10-CM diagnosis subcodes (n=4) | | Excluded ICD-10-CM diagnosis subcodes (n=7) | |
|---|---|---|---|
| **Subcode** | **Subcode description** | **Subcode** | **Subcode description** |
| K81.0 | Acute cholecystitis | K81.1 | Chronic cholecystitis |
| K81.2 | Acute cholecystitis with chronic cholecystitis | K82.0 | Obstruction of gallbladder |
| K81.9 | Cholecystitis, unspecified | K82.1 | Hydrops of gallbladder |
| K82.2 | Perforation of gallbladder | K82.3 | Fistula of gallbladder |
| | | K82.4 | Cholesterolosis of gallbladder |
| | | K82.8 | Other specified diseases of gallbladder |
| | | K82.9 | Disease of gallbladder, unspecified |

## Included and Excluded ICD-10-CM Diagnosis Subcodes for Chronic Obstructive Pulmonary Disease Condition Group

| Included ICD-10-CM diagnosis subcodes (n=3) | | Excluded ICD-10-CM diagnosis subcodes (n=0) | |
|---|---|---|---|
| **Subcode** | **Subcode description** | **Subcode** | **Subcode description** |
| J44.0 | Chronic obstructive pulmonary disease with acute lower respiratory infection | | |
| J44.1 | Chronic obstructive pulmonary disease with (acute) exacerbation | | |
| J44.9 | Chronic obstructive pulmonary disease, unspecified | | |

© 2019 Vashi AA et al. *JAMA Network Open.*

## Included and Excluded ICD-10-CM Diagnosis Subcodes for Complications of Cardiac and Vascular Prosthetic Devices/Grafts Condition Group

| Included ICD-10-CM diagnosis subcodes (n=95) | | Excluded ICD-10-CM diagnosis subcodes (n=2) | |
|---|---|---|---|
| **Subcode** | **Subcode description** | **Subcode** | **Subcode description** |
| T82.01XA | Breakdown (mechanical) of heart valve prosthesis, initial encounter | T82.847A | Pain from cardiac prosthetic devices, implants and grafts, initial encounter |
| T82.02XA | Displacement of heart valve prosthesis, initial encounter | T82.848A | Pain from vascular prosthetic devices, implants and grafts, initial encounter |
| T82.03XA | Leakage of heart valve prosthesis, initial encounter | | |
| T82.09XA | Other mechanical complication of heart valve prosthesis, initial encounter | | |
| T82.110A | Breakdown (mechanical) of cardiac electrode, initial encounter | | |
| T82.111A | Breakdown (mechanical) of cardiac pulse generator (battery), initial encounter | | |
| T82.118A | Breakdown (mechanical) of other cardiac electronic device, initial encounter | | |
| T82.119A | Breakdown (mechanical) of unspecified cardiac electronic device, initial encounter | | |
| T82.120A | Displacement of cardiac electrode, initial encounter | | |
| T82.121A | Displacement of cardiac pulse generator (battery), initial encounter | | |
| T82.128A | Displacement of other cardiac electronic device, initial encounter | | |
| T82.129A | Displacement of unspecified cardiac electronic device, initial encounter | | |
| T82.190A | Other mechanical complication of cardiac electrode, initial encounter | | |
| T82.191A | Other mechanical complication of cardiac pulse generator (battery), initial encounter | | |
| T82.198A | Other mechanical complication of other cardiac electronic device, initial encounter | | |
| T82.199A | Other mechanical complication of unspecified cardiac device, initial encounter | | |
| T82.211A | Breakdown (mechanical) of coronary artery bypass graft, initial encounter | | |

© 2019 Vashi AA et al. *JAMA Network Open.*

| | | | |
|---|---|---|---|
| T82.212A | Displacement of coronary artery bypass graft, initial encounter | | |
| T82.213A | Leakage of coronary artery bypass graft, initial encounter | | |
| T82.218A | Other mechanical complication of coronary artery bypass graft, initial encounter | | |
| T82.221A | Breakdown (mechanical) of biological heart valve graft, initial encounter | | |
| T82.222A | Displacement of biological heart valve graft, initial encounter | | |
| T82.223A | Leakage of biological heart valve graft, initial encounter | | |
| T82.228A | Other mechanical complication of biological heart valve graft, initial encounter | | |
| T82.310A | Breakdown (mechanical) of aortic (bifurcation) graft (replacement), initial encounter | | |
| T82.311A | Breakdown (mechanical) of carotid arterial graft (bypass), initial encounter | | |
| T82.312A | Breakdown (mechanical) of femoral arterial graft (bypass), initial encounter | | |
| T82.318A | Breakdown (mechanical) of other vascular grafts, initial encounter | | |
| T82.319A | Breakdown (mechanical) of unspecified vascular grafts, initial encounter | | |
| T82.320A | Displacement of aortic (bifurcation) graft (replacement), initial encounter | | |
| T82.321A | Displacement of carotid arterial graft (bypass), initial encounter | | |
| T82.322A | Displacement of femoral arterial graft (bypass), initial encounter | | |
| T82.328A | Displacement of other vascular grafts, initial encounter | | |
| T82.329A | Displacement of unspecified vascular grafts, initial encounter | | |
| T82.330A | Leakage of aortic (bifurcation) graft (replacement), initial encounter | | |
| T82.331A | Leakage of carotid arterial graft (bypass), initial encounter | | |
| T82.332A | Leakage of femoral arterial graft (bypass), initial encounter | | |
| T82.338A | Leakage of other vascular grafts, initial encounter | | |
| T82.339A | Leakage of unspecified vascular graft, initial encounter | | |

© 2019 Vashi AA et al. *JAMA Network Open.*

| | | | |
|---|---|---|---|
| T82.390A | Other mechanical complication of aortic (bifurcation) graft (replacement), initial encounter | | |
| T82.391A | Other mechanical complication of carotid arterial graft (bypass), initial encounter | | |
| T82.392A | Other mechanical complication of femoral arterial graft (bypass), initial encounter | | |
| T82.398A | Other mechanical complication of other vascular grafts, initial encounter | | |
| T82.399A | Other mechanical complication of unspecified vascular grafts, initial encounter | | |
| T82.41XA | Breakdown (mechanical) of vascular dialysis catheter, initial encounter | | |
| T82.42XA | Displacement of vascular dialysis catheter, initial encounter | | |
| T82.43XA | Leakage of vascular dialysis catheter, initial encounter | | |
| T82.49XA | Other complication of vascular dialysis catheter, initial encounter | | |
| T82.510A | Breakdown (mechanical) of surgically created arteriovenous fistula, initial encounter | | |
| T82.511A | Breakdown (mechanical) of surgically created arteriovenous shunt, initial encounter | | |
| T82.512A | Breakdown (mechanical) of artificial heart, initial encounter | | |
| T82.513A | Breakdown (mechanical) of balloon (counterpulsation) device, initial encounter | | |
| T82.514A | Breakdown (mechanical) of infusion catheter, initial encounter | | |
| T82.515A | Breakdown (mechanical) of umbrella device, initial encounter | | |
| T82.518A | Breakdown (mechanical) of other cardiac and vascular devices and implants, initial encounter | | |
| T82.519A | Breakdown (mechanical) of unspecified cardiac and vascular devices and implants, initial encounter | | |
| T82.520A | Displacement of surgically created arteriovenous fistula, initial encounter | | |
| T82.521A | Displacement of surgically created arteriovenous shunt, initial encounter | | |
| T82.522A | Displacement of artificial heart, initial encounter | | |

© 2019 Vashi AA et al. *JAMA Network Open.*

| | | | |
|---|---|---|---|
| T82.523A | Displacement of balloon (counterpulsation) device, initial encounter | | |
| T82.524A | Displacement of infusion catheter, initial encounter | | |
| T82.525A | Displacement of umbrella device, initial encounter | | |
| T82.528A | Displacement of other cardiac and vascular devices and implants, initial encounter | | |
| T82.529A | Displacement of unspecified cardiac and vascular devices and implants, initial encounter | | |
| T82.530A | Leakage of surgically created arteriovenous fistula, initial encounter | | |
| T82.531A | Leakage of surgically created arteriovenous shunt, initial encounter | | |
| T82.532A | Leakage of artificial heart, initial encounter | | |
| T82.533A | Leakage of balloon (counterpulsation) device, initial encounter | | |
| T82.534A | Leakage of infusion catheter, initial encounter | | |
| T82.535A | Leakage of umbrella device, initial encounter | | |
| T82.538A | Leakage of other cardiac and vascular devices and implants, initial encounter | | |
| T82.539A | Leakage of unspecified cardiac and vascular devices and implants, initial encounter | | |
| T82.590A | Other mechanical complication of surgically created arteriovenous fistula, initial encounter | | |
| T82.591A | Other mechanical complication of surgically created arteriovenous shunt, initial encounter | | |
| T82.592A | Other mechanical complication of artificial heart, initial encounter | | |
| T82.593A | Other mechanical complication of balloon (counterpulsation) device, initial encounter | | |
| T82.594A | Other mechanical complication of infusion catheter, initial encounter | | |
| T82.595A | Other mechanical complication of umbrella device, initial encounter | | |
| T82.598A | Other mechanical complication of other cardiac and vascular devices and implants, initial encounter | | |
| T82.599A | Other mechanical complication of unspecified cardiac and vascular devices and implants, initial encounter | | |
| T82.6XXA | Infection and inflammatory reaction due to cardiac valve prosthesis, initial encounter | | |

© 2019 Vashi AA et al. *JAMA Network Open.*

| | | | |
|---|---|---|---|
| T82.7XXA | Infection and inflammatory reaction due to other cardiac and vascular devices, implants and grafts, initial encounter | | |
| T82.817A | Embolism of cardiac prosthetic devices, implants and grafts, initial encounter | | |
| T82.818A | Embolism of vascular prosthetic devices, implants and grafts, initial encounter | | |
| T82.827A | Fibrosis of cardiac prosthetic devices, implants and grafts, initial encounter | | |
| T82.828A | Fibrosis of vascular prosthetic devices, implants and grafts, initial encounter | | |
| T82.837A | Hemorrhage of cardiac prosthetic devices, implants and grafts, initial encounter | | |
| T82.838A | Hemorrhage of vascular prosthetic devices, implants and grafts, initial encounter | | |
| T82.857A | Stenosis of cardiac prosthetic devices, implants and grafts, initial encounter | | |
| T82.858A | Stenosis of vascular prosthetic devices, implants and grafts, initial encounter | | |
| T82.867A | Thrombosis of cardiac prosthetic devices, implants and grafts, initial encounter | | |
| T82.868A | Thrombosis of vascular prosthetic devices, implants and grafts, initial encounter | | |
| T82.897A | Other specified complication of cardiac prosthetic devices, implants and grafts, initial encounter | | |
| T82.898A | Other specified complication of vascular prosthetic devices, implants and grafts, initial encounter | | |
| T82.9XXA | Unspecified complication of cardiac and vascular prosthetic device, implant and graft, initial encounter | | |

© 2019 Vashi AA et al. *JAMA Network Open.*

## Included and Excluded ICD-10-CM Diagnosis Subcodes for Complications of Procedures Condition Group

| Included ICD-10-CM diagnosis subcodes (n=62) | | Excluded ICD-10-CM diagnosis subcodes (n=10) | |
|---|---|---|---|
| **Subcode** | **Subcode description** | **Subcode** | **Subcode description** |
| T81.10XA | Postprocedural shock unspecified, initial encounter | T81.510A | Adhesions due to foreign body accidentally left in body following surgical operation, initial encounter |
| T81.11XA | Postprocedural cardiogenic shock, initial encounter | T81.511A | Adhesions due to foreign body accidentally left in body following infusion or transfusion, initial encounter |
| T81.12XA | Postprocedural septic shock, initial encounter | T81.512A | Adhesions due to foreign body accidentally left in body following kidney dialysis, initial encounter |
| T81.19XA | Other postprocedural shock, initial encounter | T81.513A | Adhesions due to foreign body accidentally left in body following injection or immunization, initial encounter |
| T81.30XA | Disruption of wound, unspecified, initial encounter | T81.514A | Adhesions due to foreign body accidentally left in body following endoscopic examination, initial encounter |
| T81.31XA | Disruption of external operation (surgical) wound, not elsewhere classified, initial encounter | T81.515A | Adhesions due to foreign body accidentally left in body following heart catheterization, initial encounter |
| T81.32XA | Disruption of internal operation (surgical) wound, not elsewhere classified, initial encounter | T81.516A | Adhesions due to foreign body accidentally left in body following aspiration, puncture or other catheterization, initial encounter |
| T81.33XA | Disruption of traumatic injury wound repair, initial encounter | T81.517A | Adhesions due to foreign body accidentally left in body following removal of catheter or packing, initial encounter |
| T81.4XXA | Infection following a procedure, initial encounter | T81.518A | Adhesions due to foreign body accidentally left in body following other procedure, initial encounter |
| T81.500A | Unspecified complication of foreign body accidentally left in body following surgical operation, initial encounter | T81.519A | Adhesions due to foreign body accidentally left in body following unspecified procedure, initial encounter |
| T81.501A | Unspecified complication of foreign body accidentally left in body following infusion or transfusion, initial encounter | | |
| T81.502A | Unspecified complication of foreign body accidentally left in body following kidney dialysis, initial encounter | | |
| T81.503A | Unspecified complication of foreign body accidentally left in body following injection or immunization, initial encounter | | |
| T81.504A | Unspecified complication of foreign body accidentally left in body following endoscopic examination, initial encounter | | |
| T81.505A | Unspecified complication of foreign body accidentally left in body following heart catheterization, initial encounter | | |

© 2019 Vashi AA et al. *JAMA Network Open.*

| | | | |
|---|---|---|---|
| T81.506A | Unspecified complication of foreign body accidentally left in body following aspiration, puncture or other catheterization, initial encounter | | |
| T81.507A | Unspecified complication of foreign body accidentally left in body following removal of catheter or packing, initial encounter | | |
| T81.508A | Unspecified complication of foreign body accidentally left in body following other procedure, initial encounter | | |
| T81.509A | Unspecified complication of foreign body accidentally left in body following unspecified procedure, initial encounter | | |
| T81.520A | Obstruction due to foreign body accidentally left in body following surgical operation, initial encounter | | |
| T81.521A | Obstruction due to foreign body accidentally left in body following infusion or transfusion, initial encounter | | |
| T81.522A | Obstruction due to foreign body accidentally left in body following kidney dialysis, initial encounter | | |
| T81.523A | Obstruction due to foreign body accidentally left in body following injection or immunization, initial encounter | | |
| T81.524A | Obstruction due to foreign body accidentally left in body following endoscopic examination, initial encounter | | |
| T81.525A | Obstruction due to foreign body accidentally left in body following heart catheterization, initial encounter | | |
| T81.526A | Obstruction due to foreign body accidentally left in body following aspiration, puncture or other catheterization, initial encounter | | |
| T81.527A | Obstruction due to foreign body accidentally left in body following removal of catheter or packing, initial encounter | | |
| T81.528A | Obstruction due to foreign body accidentally left in body following other procedure, initial encounter | | |
| T81.529A | Obstruction due to foreign body accidentally left in body following unspecified procedure, initial encounter | | |
| T81.530A | Perforation due to foreign body accidentally left in body following surgical operation, initial encounter | | |
| T81.531A | Perforation due to foreign body accidentally left in body following infusion or transfusion, initial encounter | | |
| T81.532A | Perforation due to foreign body accidentally left in body following kidney dialysis, initial encounter | | |
| T81.533A | Perforation due to foreign body accidentally left in body following injection or immunization, initial encounter | | |

© 2019 Vashi AA et al. *JAMA Network Open.*

| | | | |
|---|---|---|---|
| T81.534A | Perforation due to foreign body accidentally left in body following endoscopic examination, initial encounter | | |
| T81.535A | Perforation due to foreign body accidentally left in body following heart catheterization, initial encounter | | |
| T81.536A | Perforation due to foreign body accidentally left in body following aspiration, puncture or other catheterization, initial encounter | | |
| T81.537A | Perforation due to foreign body accidentally left in body following removal of catheter or packing, initial encounter | | |
| T81.538A | Perforation due to foreign body accidentally left in body following other procedure, initial encounter | | |
| T81.539A | Perforation due to foreign body accidentally left in body following unspecified procedure, initial encounter | | |
| T81.590A | Other complications of foreign body accidentally left in body following surgical operation, initial encounter | | |
| T81.591A | Other complications of foreign body accidentally left in body following infusion or transfusion, initial encounter | | |
| T81.592A | Other complications of foreign body accidentally left in body following kidney dialysis, initial encounter | | |
| T81.593A | Other complications of foreign body accidentally left in body following injection or immunization, initial encounter | | |
| T81.594A | Other complications of foreign body accidentally left in body following endoscopic examination, initial encounter | | |
| T81.595A | Other complications of foreign body accidentally left in body following heart catheterization, initial encounter | | |
| T81.596A | Other complications of foreign body accidentally left in body following aspiration, puncture or other catheterization, initial encounter | | |
| T81.597A | Other complications of foreign body accidentally left in body following removal of catheter or packing, initial encounter | | |
| T81.598A | Other complications of foreign body accidentally left in body following other procedure, initial encounter | | |
| T81.599A | Other complications of foreign body accidentally left in body following unspecified procedure, initial encounter | | |
| T81.60XA | Unspecified acute reaction to foreign substance accidentally left during a procedure, initial encounter | | |
| T81.61XA | Aseptic peritonitis due to foreign substance accidentally left during a procedure, initial encounter | | |

© 2019 Vashi AA et al. *JAMA Network Open.*

| | | | |
|---|---|---|---|
| T81.69XA | Other acute reaction to foreign substance accidentally left during a procedure, initial encounter | | |
| T81.710A | Complication of mesenteric artery following a procedure, not elsewhere classified, initial encounter | | |
| T81.711A | Complication of renal artery following a procedure, not elsewhere classified, initial encounter | | |
| T81.718A | Complication of other artery following a procedure, not elsewhere classified, initial encounter | | |
| T81.719A | Complication of unspecified artery following a procedure, not elsewhere classified, initial encounter | | |
| T81.72XA | Complication of vein following a procedure, not elsewhere classified, initial encounter | | |
| T81.81XA | Complication of inhalation therapy, initial encounter | | |
| T81.82XA | Emphysema (subcutaneous) resulting from a procedure, initial encounter | | |
| T81.83XA | Persistent postprocedural fistula, initial encounter | | |
| T81.89XA | Other complications of procedures, not elsewhere classified, initial encounter | | |
| T81.9XXA | Unspecified complication of procedure, initial encounter | | |

© 2019 Vashi AA et al. *JAMA Network Open.*

## Included and Excluded ICD-10-CM Diagnosis Subcodes for Diabetes Mellitus-Acute Condition Group

| | Included ICD-10-CM diagnosis subcodes (n=20) | | Excluded ICD-10-CM diagnosis subcodes (n=0) | |
|---|---|---|---|---|
| | **Subcode** | **Subcode description** | **Subcode** | **Subcode description** |
| | E10.10 | Type 1 diabetes mellitus with ketoacidosis without coma | | |
| | E10.11 | Type 1 diabetes mellitus with ketoacidosis with coma | | |
| | E10.49 | Type 1 diabetes mellitus with other diabetic neurological complication | | |
| | E10.52 | Type 1 diabetes mellitus with diabetic peripheral angiopathy with gangrene | | |
| | E10.59 | Type 1 diabetes mellitus with other circulatory complications | | |
| | E10.621 | Type 1 diabetes mellitus with foot ulcer | | |
| | E10.641 | Type 1 diabetes mellitus with hypoglycemia with coma | | |
| | E10.649 | Type 1 diabetes mellitus with hypoglycemia without coma | | |
| | E10.65 | Type 1 diabetes mellitus with hyperglycemia | | |
| | E10.69 | Type 1 diabetes mellitus with other specified complication | | |
| | E11.00 | Type 2 diabetes mellitus with hyperosmolarity without nonketotic hyperglycemic-hyperosmolar coma (NKHHC) | | |
| | E11.01 | Type 2 diabetes mellitus with hyperosmolarity with coma | | |
| | E11.49 | Type 2 diabetes mellitus with other diabetic neurological complication | | |
| | E11.52 | Type 2 diabetes mellitus with diabetic peripheral angiopathy with gangrene | | |
| | E11.59 | Type 2 diabetes mellitus with other circulatory complications | | |
| | E11.621 | Type 2 diabetes mellitus with foot ulcer | | |
| | E11.641 | Type 2 diabetes mellitus with hypoglycemia with coma | | |
| | E11.649 | Type 2 diabetes mellitus with hypoglycemia without coma | | |
| | E11.65 | Type 2 diabetes mellitus with hyperglycemia | | |
| | E11.69 | Type 2 diabetes mellitus with other specified complication | | |

© 2019 Vashi AA et al. *JAMA Network Open.*

## Included and Excluded ICD-10-CM Diagnosis Subcodes for Disorders of the Brain Condition Group

| Included ICD-10-CM diagnosis subcodes (n=14) | | Excluded ICD-10-CM diagnosis subcodes (n=5) | |
|---|---|---|---|
| **Subcode** | **Subcode description** | **Subcode** | **Subcode description** |
| G06.0 | Intracranial abscess and granuloma | G93.0 | Cerebral cysts |
| G06.1 | Intraspinal abscess and granuloma | G93.3 | Postviral fatigue syndrome |
| G06.2 | Extradural and subdural abscess, unspecified | G93.7 | Reye's syndrome |
| G07. | Intracranial and intraspinal abscess and granuloma in diseases classified elsewhere | G93.81 | Temporal sclerosis |
| G92. | Toxic encephalopathy | G93.82 | Brain death |
| G93.1 | Anoxic brain damage, not elsewhere classified | | |
| G93.2 | Benign intracranial hypertension | | |
| G93.40 | Encephalopathy, unspecified | | |
| G93.41 | Metabolic encephalopathy | | |
| G93.49 | Other encephalopathy | | |
| G93.5 | Compression of brain | | |
| G93.6 | Cerebral edema | | |
| G93.89 | Other specified disorders of brain | | |
| G93.9 | Disorder of brain, unspecified | | |

© 2019 Vashi AA et al. *JAMA Network Open.*

## Included and Excluded ICD-10-CM Diagnosis Subcodes for Early Complications of Trauma Condition Group

| Included ICD-10-CM diagnosis subcodes (n=18) | | Excluded ICD-10-CM diagnosis subcodes (n=0) | |
|---|---|---|---|
| **Subcode** | **Subcode description** | **Subcode** | **Subcode description** |
| T79.0XXA | Air embolism (traumatic), initial encounter | | |
| T79.1XXA | Fat embolism (traumatic), initial encounter | | |
| T79.2XXA | Traumatic secondary and recurrent hemorrhage and seroma, initial encounter | | |
| T79.4XXA | Traumatic shock, initial encounter | | |
| T79.5XXA | Traumatic anuria, initial encounter | | |
| T79.6XXA | Traumatic ischemia of muscle, initial encounter | | |
| T79.7XXA | Traumatic subcutaneous emphysema, initial encounter | | |
| T79.8XXA | Other early complications of trauma, initial encounter | | |
| T79.9XXA | Unspecified early complication of trauma, initial encounter | | |
| T79.A0XA | Compartment syndrome, unspecified, initial encounter | | |
| T79.A11A | Traumatic compartment syndrome of right upper extremity, initial encounter | | |
| T79.A12A | Traumatic compartment syndrome of left upper extremity, initial encounter | | |
| T79.A19A | Traumatic compartment syndrome of unspecified upper extremity, initial encounter | | |
| T79.A21A | Traumatic compartment syndrome of right lower extremity, initial encounter | | |
| T79.A22A | Traumatic compartment syndrome of left lower extremity, initial encounter | | |
| T79.A29A | Traumatic compartment syndrome of unspecified lower extremity, initial encounter | | |
| T79.A3XA | Traumatic compartment syndrome of abdomen, initial encounter | | |
| T79.A9XA | Traumatic compartment syndrome of other sites, initial encounter | | |

© 2019 Vashi AA et al. *JAMA Network Open.*

## Included and Excluded ICD-10-CM Diagnosis Subcodes for Ectopic Pregnancy Condition Group

| Included ICD-10-CM diagnosis subcodes (n=5) | | Excluded ICD-10-CM diagnosis subcodes (n=0) | |
|---|---|---|---|
| **Subcode** | **Subcode description** | **Subcode** | **Subcode description** |
| O00.0 | Abdominal pregnancy | | |
| O00.1 | Tubal pregnancy | | |
| O00.2 | Ovarian pregnancy | | |
| O00.8 | Other ectopic pregnancy | | |
| O00.9 | Ectopic pregnancy, unspecified | | |

© 2019 Vashi AA et al. *JAMA Network Open.*

## Included and Excluded ICD-10-CM Diagnosis Subcodes for Encephalitis, Myelitis and Encephalomyelitis Condition Group

| Included ICD-10-CM diagnosis subcodes (n=18) | | Excluded ICD-10-CM diagnosis subcodes (n=0) | |
|---|---|---|---|
| | | | |
| **Subcode** | **Subcode description** | **Subcode** | **Subcode description** |
| A32.12 | Listerial meningoencephalitis | | |
| A39.81 | Meningococcal encephalitis | | |
| A42.82 | Actinomycotic encephalitis | | |
| G04.00 | Acute disseminated encephalitis and encephalomyelitis, unspecified | | |
| G04.01 | Postinfectious acute disseminated encephalitis and encephalomyelitis (postinfectious ADEM) | | |
| G04.02 | Postimmunization acute disseminated encephalitis, myelitis and encephalomyelitis | | |
| G04.1 | Tropical spastic paraplegia | | |
| G04.2 | Bacterial meningoencephalitis and meningomyelitis, not elsewhere classified | | |
| G04.30 | Acute necrotizing hemorrhagic encephalopathy, unspecified | | |
| G04.31 | Postinfectious acute necrotizing hemorrhagic encephalopathy | | |
| G04.32 | Postimmunization acute necrotizing hemorrhagic encephalopathy | | |
| G04.39 | Other acute necrotizing hemorrhagic encephalopathy | | |
| G04.81 | Other encephalitis and encephalomyelitis | | |
| G04.89 | Other myelitis | | |
| G04.90 | Encephalitis and encephalomyelitis, unspecified | | |
| G04.91 | Myelitis, unspecified | | |
| G05.3 | Encephalitis and encephalomyelitis in diseases classified elsewhere | | |
| G05.4 | Myelitis in diseases classified elsewhere | | |

© 2019 Vashi AA et al. *JAMA Network Open.*

## Included and Excluded ICD-10-CM Diagnosis Subcodes for Environmental Exposures Condition Group

| Included ICD-10-CM diagnosis subcodes (n=6) | | Excluded ICD-10-CM diagnosis subcodes (n=5) | |
|---|---|---|---|
| | | | |
| **Subcode** | **Subcode description** | **Subcode** | **Subcode description** |
| T67.0XXA | Heatstroke and sunstroke, initial encounter | T67.2XXA | Heat cramp, initial encounter |
| T67.1XXA | Heat syncope, initial encounter | T67.6XXA | Heat fatigue, transient, initial encounter |
| T67.3XXA | Heat exhaustion, anhydrotic, initial encounter | T67.7XXA | Heat edema, initial encounter |
| T67.4XXA | Heat exhaustion due to salt depletion, initial encounter | T67.8XXA | Other effects of heat and light, initial encounter |
| T67.5XXA | Heat exhaustion, unspecified, initial encounter | T67.9XXA | Effect of heat and light, unspecified, initial encounter |
| T68.XXXA | Hypothermia, initial encounter | | |

© 2019 Vashi AA et al. *JAMA Network Open.*

## Included and Excluded ICD-10-CM Diagnosis Subcodes for Femur Fracture Condition Group

| Included ICD-10-CM diagnosis subcodes (n=462) | | Excluded ICD-10-CM diagnosis subcodes (n=0) | |
|---|---|---|---|
| **Subcode** | **Subcode description** | **Subcode** | **Subcode description** |
| S72.001A | Fracture of unspecified part of neck of right femur, initial encounter for closed fracture | | |
| S72.001B | Fracture of unspecified part of neck of right femur, initial encounter for open fracture type I or II | | |
| S72.001C | Fracture of unspecified part of neck of right femur, initial encounter for open fracture type IIIA, IIIB, or IIIC | | |
| S72.002A | Fracture of unspecified part of neck of left femur, initial encounter for closed fracture | | |
| S72.002B | Fracture of unspecified part of neck of left femur, initial encounter for open fracture type I or II | | |
| S72.002C | Fracture of unspecified part of neck of left femur, initial encounter for open fracture type IIIA, IIIB, or IIIC | | |
| S72.009A | Fracture of unspecified part of neck of unspecified femur, initial encounter for closed fracture | | |
| S72.009B | Fracture of unspecified part of neck of unspecified femur, initial encounter for open fracture type I or II | | |
| S72.009C | Fracture of unspecified part of neck of unspecified femur, initial encounter for open fracture type IIIA, IIIB, or IIIC | | |
| S72.011A | Unspecified intracapsular fracture of right femur, initial encounter for closed fracture | | |
| S72.011B | Unspecified intracapsular fracture of right femur, initial encounter for open fracture type I or II | | |
| S72.011C | Unspecified intracapsular fracture of right femur, initial encounter for open fracture type IIIA, IIIB, or IIIC | | |
| S72.012A | Unspecified intracapsular fracture of left femur, initial encounter for closed fracture | | |
| S72.012B | Unspecified intracapsular fracture of left femur, initial encounter for open fracture type I or II | | |
| S72.012C | Unspecified intracapsular fracture of left femur, initial encounter for open fracture type IIIA, IIIB, or IIIC | | |
| S72.019A | Unspecified intracapsular fracture of unspecified femur, initial encounter for closed fracture | | |
| S72.019B | Unspecified intracapsular fracture of unspecified femur, initial encounter for open fracture type I or II | | |

© 2019 Vashi AA et al. *JAMA Network Open.*

| | | | |
|---|---|---|---|
| S72.019C | Unspecified intracapsular fracture of unspecified femur, initial encounter for open fracture type IIIA, IIIB, or IIIC | | |
| S72.021A | Displaced fracture of epiphysis (separation) (upper) of right femur, initial encounter for closed fracture | | |
| S72.021B | Displaced fracture of epiphysis (separation) (upper) of right femur, initial encounter for open fracture type I or II | | |
| S72.021C | Displaced fracture of epiphysis (separation) (upper) of right femur, initial encounter for open fracture type IIIA, IIIB, or IIIC | | |
| S72.022A | Displaced fracture of epiphysis (separation) (upper) of left femur, initial encounter for closed fracture | | |
| S72.022B | Displaced fracture of epiphysis (separation) (upper) of left femur, initial encounter for open fracture type I or II | | |
| S72.022C | Displaced fracture of epiphysis (separation) (upper) of left femur, initial encounter for open fracture type IIIA, IIIB, or IIIC | | |
| S72.023A | Displaced fracture of epiphysis (separation) (upper) of unspecified femur, initial encounter for closed fracture | | |
| S72.023B | Displaced fracture of epiphysis (separation) (upper) of unspecified femur, initial encounter for open fracture type I or II | | |
| S72.023C | Displaced fracture of epiphysis (separation) (upper) of unspecified femur, initial encounter for open fracture type IIIA, IIIB, or IIIC | | |
| S72.024A | Nondisplaced fracture of epiphysis (separation) (upper) of right femur, initial encounter for closed fracture | | |
| S72.024B | Nondisplaced fracture of epiphysis (separation) (upper) of right femur, initial encounter for open fracture type I or II | | |
| S72.024C | Nondisplaced fracture of epiphysis (separation) (upper) of right femur, initial encounter for open fracture type IIIA, IIIB, or IIIC | | |
| S72.025A | Nondisplaced fracture of epiphysis (separation) (upper) of left femur, initial encounter for closed fracture | | |
| S72.025B | Nondisplaced fracture of epiphysis (separation) (upper) of left femur, initial encounter for open fracture type I or II | | |
| S72.025C | Nondisplaced fracture of epiphysis (separation) (upper) of left femur, initial encounter for open fracture type IIIA, IIIB, or IIIC | | |

© 2019 Vashi AA et al. *JAMA Network Open.*

| | | | |
|---|---|---|---|
| S72.026A | Nondisplaced fracture of epiphysis (separation) (upper) of unspecified femur, initial encounter for closed fracture | | |
| S72.026B | Nondisplaced fracture of epiphysis (separation) (upper) of unspecified femur, initial encounter for open fracture type I or II | | |
| S72.026C | Nondisplaced fracture of epiphysis (separation) (upper) of unspecified femur, initial encounter for open fracture type IIIA, IIIB, or IIIC | | |
| S72.031A | Displaced midcervical fracture of right femur, initial encounter for closed fracture | | |
| S72.031B | Displaced midcervical fracture of right femur, initial encounter for open fracture type I or II | | |
| S72.031C | Displaced midcervical fracture of right femur, initial encounter for open fracture type IIIA, IIIB, or IIIC | | |
| S72.032A | Displaced midcervical fracture of left femur, initial encounter for closed fracture | | |
| S72.032B | Displaced midcervical fracture of left femur, initial encounter for open fracture type I or II | | |
| S72.032C | Displaced midcervical fracture of left femur, initial encounter for open fracture type IIIA, IIIB, or IIIC | | |
| S72.033A | Displaced midcervical fracture of unspecified femur, initial encounter for closed fracture | | |
| S72.033B | Displaced midcervical fracture of unspecified femur, initial encounter for open fracture type I or II | | |
| S72.033C | Displaced midcervical fracture of unspecified femur, initial encounter for open fracture type IIIA, IIIB, or IIIC | | |
| S72.034A | Nondisplaced midcervical fracture of right femur, initial encounter for closed fracture | | |
| S72.034B | Nondisplaced midcervical fracture of right femur, initial encounter for open fracture type I or II | | |
| S72.034C | Nondisplaced midcervical fracture of right femur, initial encounter for open fracture type IIIA, IIIB, or IIIC | | |
| S72.035A | Nondisplaced midcervical fracture of left femur, initial encounter for closed fracture | | |
| S72.035B | Nondisplaced midcervical fracture of left femur, initial encounter for open fracture type I or II | | |
| S72.035C | Nondisplaced midcervical fracture of left femur, initial encounter for open fracture type IIIA, IIIB, or IIIC | | |

© 2019 Vashi AA et al. *JAMA Network Open.*

| | | | |
|---|---|---|---|
| S72.036A | Nondisplaced midcervical fracture of unspecified femur, initial encounter for closed fracture | | |
| S72.036B | Nondisplaced midcervical fracture of unspecified femur, initial encounter for open fracture type I or II | | |
| S72.036C | Nondisplaced midcervical fracture of unspecified femur, initial encounter for open fracture type IIIA, IIIB, or IIIC | | |
| S72.041A | Displaced fracture of base of neck of right femur, initial encounter for closed fracture | | |
| S72.041B | Displaced fracture of base of neck of right femur, initial encounter for open fracture type I or II | | |
| S72.041C | Displaced fracture of base of neck of right femur, initial encounter for open fracture type IIIA, IIIB, or IIIC | | |
| S72.042A | Displaced fracture of base of neck of left femur, initial encounter for closed fracture | | |
| S72.042B | Displaced fracture of base of neck of left femur, initial encounter for open fracture type I or II | | |
| S72.042C | Displaced fracture of base of neck of left femur, initial encounter for open fracture type IIIA, IIIB, or IIIC | | |
| S72.043A | Displaced fracture of base of neck of unspecified femur, initial encounter for closed fracture | | |
| S72.043B | Displaced fracture of base of neck of unspecified femur, initial encounter for open fracture type I or II | | |
| S72.043C | Displaced fracture of base of neck of unspecified femur, initial encounter for open fracture type IIIA, IIIB, or IIIC | | |
| S72.044A | Nondisplaced fracture of base of neck of right femur, initial encounter for closed fracture | | |
| S72.044B | Nondisplaced fracture of base of neck of right femur, initial encounter for open fracture type I or II | | |
| S72.044C | Nondisplaced fracture of base of neck of right femur, initial encounter for open fracture type IIIA, IIIB, or IIIC | | |
| S72.045A | Nondisplaced fracture of base of neck of left femur, initial encounter for closed fracture | | |
| S72.045B | Nondisplaced fracture of base of neck of left femur, initial encounter for open fracture type I or II | | |
| S72.045C | Nondisplaced fracture of base of neck of left femur, initial encounter for open fracture type IIIA, IIIB, or IIIC | | |
| S72.046A | Nondisplaced fracture of base of neck of unspecified femur, initial encounter for closed fracture | | |

© 2019 Vashi AA et al. *JAMA Network Open.*

| | | | |
|---|---|---|---|
| S72.046B | Nondisplaced fracture of base of neck of unspecified femur, initial encounter for open fracture type I or II | | |
| S72.046C | Nondisplaced fracture of base of neck of unspecified femur, initial encounter for open fracture type IIIA, IIIB, or IIIC | | |
| S72.051A | Unspecified fracture of head of right femur, initial encounter for closed fracture | | |
| S72.051B | Unspecified fracture of head of right femur, initial encounter for open fracture type I or II | | |
| S72.051C | Unspecified fracture of head of right femur, initial encounter for open fracture type IIIA, IIIB, or IIIC | | |
| S72.052A | Unspecified fracture of head of left femur, initial encounter for closed fracture | | |
| S72.052B | Unspecified fracture of head of left femur, initial encounter for open fracture type I or II | | |
| S72.052C | Unspecified fracture of head of left femur, initial encounter for open fracture type IIIA, IIIB, or IIIC | | |
| S72.059A | Unspecified fracture of head of unspecified femur, initial encounter for closed fracture | | |
| S72.059B | Unspecified fracture of head of unspecified femur, initial encounter for open fracture type I or II | | |
| S72.059C | Unspecified fracture of head of unspecified femur, initial encounter for open fracture type IIIA, IIIB, or IIIC | | |
| S72.061A | Displaced articular fracture of head of right femur, initial encounter for closed fracture | | |
| S72.061B | Displaced articular fracture of head of right femur, initial encounter for open fracture type I or II | | |
| S72.061C | Displaced articular fracture of head of right femur, initial encounter for open fracture type IIIA, IIIB, or IIIC | | |
| S72.062A | Displaced articular fracture of head of left femur, initial encounter for closed fracture | | |
| S72.062B | Displaced articular fracture of head of left femur, initial encounter for open fracture type I or II | | |
| S72.062C | Displaced articular fracture of head of left femur, initial encounter for open fracture type IIIA, IIIB, or IIIC | | |
| S72.063A | Displaced articular fracture of head of unspecified femur, initial encounter for closed fracture | | |
| S72.063B | Displaced articular fracture of head of unspecified femur, initial encounter for open fracture type I or II | | |

© 2019 Vashi AA et al. *JAMA Network Open.*

| | | | |
|---|---|---|---|
| S72.063C | Displaced articular fracture of head of unspecified femur, initial encounter for open fracture type IIIA, IIIB, or IIIC | | |
| S72.064A | Nondisplaced articular fracture of head of right femur, initial encounter for closed fracture | | |
| S72.064B | Nondisplaced articular fracture of head of right femur, initial encounter for open fracture type I or II | | |
| S72.064C | Nondisplaced articular fracture of head of right femur, initial encounter for open fracture type IIIA, IIIB, or IIIC | | |
| S72.065A | Nondisplaced articular fracture of head of left femur, initial encounter for closed fracture | | |
| S72.065B | Nondisplaced articular fracture of head of left femur, initial encounter for open fracture type I or II | | |
| S72.065C | Nondisplaced articular fracture of head of left femur, initial encounter for open fracture type IIIA, IIIB, or IIIC | | |
| S72.066A | Nondisplaced articular fracture of head of unspecified femur, initial encounter for closed fracture | | |
| S72.066B | Nondisplaced articular fracture of head of unspecified femur, initial encounter for open fracture type I or II | | |
| S72.066C | Nondisplaced articular fracture of head of unspecified femur, initial encounter for open fracture type IIIA, IIIB, or IIIC | | |
| S72.091A | Other fracture of head and neck of right femur, initial encounter for closed fracture | | |
| S72.091B | Other fracture of head and neck of right femur, initial encounter for open fracture type I or II | | |
| S72.091C | Other fracture of head and neck of right femur, initial encounter for open fracture type IIIA, IIIB, or IIIC | | |
| S72.092A | Other fracture of head and neck of left femur, initial encounter for closed fracture | | |
| S72.092B | Other fracture of head and neck of left femur, initial encounter for open fracture type I or II | | |
| S72.092C | Other fracture of head and neck of left femur, initial encounter for open fracture type IIIA, IIIB, or IIIC | | |
| S72.099A | Other fracture of head and neck of unspecified femur, initial encounter for closed fracture | | |
| S72.099B | Other fracture of head and neck of unspecified femur, initial encounter for open fracture type I or II | | |
| S72.099C | Other fracture of head and neck of unspecified femur, initial encounter for open fracture type IIIA, IIIB, or IIIC | | |

© 2019 Vashi AA et al. *JAMA Network Open.*

| | | | |
|---|---|---|---|
| S72.101A | Unspecified trochanteric fracture of right femur, initial encounter for closed fracture | | |
| S72.101B | Unspecified trochanteric fracture of right femur, initial encounter for open fracture type I or II | | |
| S72.101C | Unspecified trochanteric fracture of right femur, initial encounter for open fracture type IIIA, IIIB, or IIIC | | |
| S72.102A | Unspecified trochanteric fracture of left femur, initial encounter for closed fracture | | |
| S72.102B | Unspecified trochanteric fracture of left femur, initial encounter for open fracture type I or II | | |
| S72.102C | Unspecified trochanteric fracture of left femur, initial encounter for open fracture type IIIA, IIIB, or IIIC | | |
| S72.109A | Unspecified trochanteric fracture of unspecified femur, initial encounter for closed fracture | | |
| S72.109B | Unspecified trochanteric fracture of unspecified femur, initial encounter for open fracture type I or II | | |
| S72.109C | Unspecified trochanteric fracture of unspecified femur, initial encounter for open fracture type IIIA, IIIB, or IIIC | | |
| S72.111A | Displaced fracture of greater trochanter of right femur, initial encounter for closed fracture | | |
| S72.111B | Displaced fracture of greater trochanter of right femur, initial encounter for open fracture type I or II | | |
| S72.111C | Displaced fracture of greater trochanter of right femur, initial encounter for open fracture type IIIA, IIIB, or IIIC | | |
| S72.112A | Displaced fracture of greater trochanter of left femur, initial encounter for closed fracture | | |
| S72.112B | Displaced fracture of greater trochanter of left femur, initial encounter for open fracture type I or II | | |
| S72.112C | Displaced fracture of greater trochanter of left femur, initial encounter for open fracture type IIIA, IIIB, or IIIC | | |
| S72.113A | Displaced fracture of greater trochanter of unspecified femur, initial encounter for closed fracture | | |
| S72.113B | Displaced fracture of greater trochanter of unspecified femur, initial encounter for open fracture type I or II | | |
| S72.113C | Displaced fracture of greater trochanter of unspecified femur, initial encounter for open fracture type IIIA, IIIB, or IIIC | | |
| S72.114A | Nondisplaced fracture of greater trochanter of right femur, initial encounter for closed fracture | | |

© 2019 Vashi AA et al. *JAMA Network Open.*

| | | | |
|---|---|---|---|
| S72.114B | Nondisplaced fracture of greater trochanter of right femur, initial encounter for open fracture type I or II | | |
| S72.114C | Nondisplaced fracture of greater trochanter of right femur, initial encounter for open fracture type IIIA, IIIB, or IIIC | | |
| S72.115A | Nondisplaced fracture of greater trochanter of left femur, initial encounter for closed fracture | | |
| S72.115B | Nondisplaced fracture of greater trochanter of left femur, initial encounter for open fracture type I or II | | |
| S72.115C | Nondisplaced fracture of greater trochanter of left femur, initial encounter for open fracture type IIIA, IIIB, or IIIC | | |
| S72.116A | Nondisplaced fracture of greater trochanter of unspecified femur, initial encounter for closed fracture | | |
| S72.116B | Nondisplaced fracture of greater trochanter of unspecified femur, initial encounter for open fracture type I or II | | |
| S72.116C | Nondisplaced fracture of greater trochanter of unspecified femur, initial encounter for open fracture type IIIA, IIIB, or IIIC | | |
| S72.121A | Displaced fracture of lesser trochanter of right femur, initial encounter for closed fracture | | |
| S72.121B | Displaced fracture of lesser trochanter of right femur, initial encounter for open fracture type I or II | | |
| S72.121C | Displaced fracture of lesser trochanter of right femur, initial encounter for open fracture type IIIA, IIIB, or IIIC | | |
| S72.122A | Displaced fracture of lesser trochanter of left femur, initial encounter for closed fracture | | |
| S72.122B | Displaced fracture of lesser trochanter of left femur, initial encounter for open fracture type I or II | | |
| S72.122C | Displaced fracture of lesser trochanter of left femur, initial encounter for open fracture type IIIA, IIIB, or IIIC | | |
| S72.123A | Displaced fracture of lesser trochanter of unspecified femur, initial encounter for closed fracture | | |
| S72.123B | Displaced fracture of lesser trochanter of unspecified femur, initial encounter for open fracture type I or II | | |
| S72.123C | Displaced fracture of lesser trochanter of unspecified femur, initial encounter for open fracture type IIIA, IIIB, or IIIC | | |

© 2019 Vashi AA et al. *JAMA Network Open.*

| | | | |
|---|---|---|---|
| S72.124A | Nondisplaced fracture of lesser trochanter of right femur, initial encounter for closed fracture | | |
| S72.124B | Nondisplaced fracture of lesser trochanter of right femur, initial encounter for open fracture type I or II | | |
| S72.124C | Nondisplaced fracture of lesser trochanter of right femur, initial encounter for open fracture type IIIA, IIIB, or IIIC | | |
| S72.125A | Nondisplaced fracture of lesser trochanter of left femur, initial encounter for closed fracture | | |
| S72.125B | Nondisplaced fracture of lesser trochanter of left femur, initial encounter for open fracture type I or II | | |
| S72.125C | Nondisplaced fracture of lesser trochanter of left femur, initial encounter for open fracture type IIIA, IIIB, or IIIC | | |
| S72.126A | Nondisplaced fracture of lesser trochanter of unspecified femur, initial encounter for closed fracture | | |
| S72.126B | Nondisplaced fracture of lesser trochanter of unspecified femur, initial encounter for open fracture type I or II | | |
| S72.126C | Nondisplaced fracture of lesser trochanter of unspecified femur, initial encounter for open fracture type IIIA, IIIB, or IIIC | | |
| S72.131A | Displaced apophyseal fracture of right femur, initial encounter for closed fracture | | |
| S72.131B | Displaced apophyseal fracture of right femur, initial encounter for open fracture type I or II | | |
| S72.131C | Displaced apophyseal fracture of right femur, initial encounter for open fracture type IIIA, IIIB, or IIIC | | |
| S72.132A | Displaced apophyseal fracture of left femur, initial encounter for closed fracture | | |
| S72.132B | Displaced apophyseal fracture of left femur, initial encounter for open fracture type I or II | | |
| S72.132C | Displaced apophyseal fracture of left femur, initial encounter for open fracture type IIIA, IIIB, or IIIC | | |
| S72.133A | Displaced apophyseal fracture of unspecified femur, initial encounter for closed fracture | | |
| S72.133B | Displaced apophyseal fracture of unspecified femur, initial encounter for open fracture type I or II | | |
| S72.133C | Displaced apophyseal fracture of unspecified femur, initial encounter for open fracture type IIIA, IIIB, or IIIC | | |
| S72.134A | Nondisplaced apophyseal fracture of right femur, initial encounter for closed fracture | | |

© 2019 Vashi AA et al. *JAMA Network Open.*

| | | | |
|---|---|---|---|
| S72.134B | Nondisplaced apophyseal fracture of right femur, initial encounter for open fracture type I or II | | |
| S72.134C | Nondisplaced apophyseal fracture of right femur, initial encounter for open fracture type IIIA, IIIB, or IIIC | | |
| S72.135A | Nondisplaced apophyseal fracture of left femur, initial encounter for closed fracture | | |
| S72.135B | Nondisplaced apophyseal fracture of left femur, initial encounter for open fracture type I or II | | |
| S72.135C | Nondisplaced apophyseal fracture of left femur, initial encounter for open fracture type IIIA, IIIB, or IIIC | | |
| S72.136A | Nondisplaced apophyseal fracture of unspecified femur, initial encounter for closed fracture | | |
| S72.136B | Nondisplaced apophyseal fracture of unspecified femur, initial encounter for open fracture type I or II | | |
| S72.136C | Nondisplaced apophyseal fracture of unspecified femur, initial encounter for open fracture type IIIA, IIIB, or IIIC | | |
| S72.141A | Displaced intertrochanteric fracture of right femur, initial encounter for closed fracture | | |
| S72.141B | Displaced intertrochanteric fracture of right femur, initial encounter for open fracture type I or II | | |
| S72.141C | Displaced intertrochanteric fracture of right femur, initial encounter for open fracture type IIIA, IIIB, or IIIC | | |
| S72.142A | Displaced intertrochanteric fracture of left femur, initial encounter for closed fracture | | |
| S72.142B | Displaced intertrochanteric fracture of left femur, initial encounter for open fracture type I or II | | |
| S72.142C | Displaced intertrochanteric fracture of left femur, initial encounter for open fracture type IIIA, IIIB, or IIIC | | |
| S72.143A | Displaced intertrochanteric fracture of unspecified femur, initial encounter for closed fracture | | |
| S72.143B | Displaced intertrochanteric fracture of unspecified femur, initial encounter for open fracture type I or II | | |
| S72.143C | Displaced intertrochanteric fracture of unspecified femur, initial encounter for open fracture type IIIA, IIIB, or IIIC | | |
| S72.144A | Nondisplaced intertrochanteric fracture of right femur, initial encounter for closed fracture | | |
| S72.144B | Nondisplaced intertrochanteric fracture of right femur, initial encounter for open fracture type I or II | | |

© 2019 Vashi AA et al. *JAMA Network Open.*

| | | | |
|---|---|---|---|
| S72.144C | Nondisplaced intertrochanteric fracture of right femur, initial encounter for open fracture type IIIA, IIIB, or IIIC | | |
| S72.145A | Nondisplaced intertrochanteric fracture of left femur, initial encounter for closed fracture | | |
| S72.145B | Nondisplaced intertrochanteric fracture of left femur, initial encounter for open fracture type I or II | | |
| S72.145C | Nondisplaced intertrochanteric fracture of left femur, initial encounter for open fracture type IIIA, IIIB, or IIIC | | |
| S72.146A | Nondisplaced intertrochanteric fracture of unspecified femur, initial encounter for closed fracture | | |
| S72.146B | Nondisplaced intertrochanteric fracture of unspecified femur, initial encounter for open fracture type I or II | | |
| S72.146C | Nondisplaced intertrochanteric fracture of unspecified femur, initial encounter for open fracture type IIIA, IIIB, or IIIC | | |
| S72.21XA | Displaced subtrochanteric fracture of right femur, initial encounter for closed fracture | | |
| S72.21XB | Displaced subtrochanteric fracture of right femur, initial encounter for open fracture type I or II | | |
| S72.21XC | Displaced subtrochanteric fracture of right femur, initial encounter for open fracture type IIIA, IIIB, or IIIC | | |
| S72.22XA | Displaced subtrochanteric fracture of left femur, initial encounter for closed fracture | | |
| S72.22XB | Displaced subtrochanteric fracture of left femur, initial encounter for open fracture type I or II | | |
| S72.22XC | Displaced subtrochanteric fracture of left femur, initial encounter for open fracture type IIIA, IIIB, or IIIC | | |
| S72.23XA | Displaced subtrochanteric fracture of unspecified femur, initial encounter for closed fracture | | |
| S72.23XB | Displaced subtrochanteric fracture of unspecified femur, initial encounter for open fracture type I or II | | |
| S72.23XC | Displaced subtrochanteric fracture of unspecified femur, initial encounter for open fracture type IIIA, IIIB, or IIIC | | |
| S72.24XA | Nondisplaced subtrochanteric fracture of right femur, initial encounter for closed fracture | | |
| S72.24XB | Nondisplaced subtrochanteric fracture of right femur, initial encounter for open fracture type I or II | | |
| S72.24XC | Nondisplaced subtrochanteric fracture of right femur, initial encounter for open fracture type IIIA, IIIB, or IIIC | | |

© 2019 Vashi AA et al. *JAMA Network Open.*

| | | | |
|---|---|---|---|
| S72.25XA | Nondisplaced subtrochanteric fracture of left femur, initial encounter for closed fracture | | |
| S72.25XB | Nondisplaced subtrochanteric fracture of left femur, initial encounter for open fracture type I or II | | |
| S72.25XC | Nondisplaced subtrochanteric fracture of left femur, initial encounter for open fracture type IIIA, IIIB, or IIIC | | |
| S72.26XA | Nondisplaced subtrochanteric fracture of unspecified femur, initial encounter for closed fracture | | |
| S72.26XB | Nondisplaced subtrochanteric fracture of unspecified femur, initial encounter for open fracture type I or II | | |
| S72.26XC | Nondisplaced subtrochanteric fracture of unspecified femur, initial encounter for open fracture type IIIA, IIIB, or IIIC | | |
| S72.301A | Unspecified fracture of shaft of right femur, initial encounter for closed fracture | | |
| S72.301B | Unspecified fracture of shaft of right femur, initial encounter for open fracture type I or II | | |
| S72.301C | Unspecified fracture of shaft of right femur, initial encounter for open fracture type IIIA, IIIB, or IIIC | | |
| S72.302A | Unspecified fracture of shaft of left femur, initial encounter for closed fracture | | |
| S72.302B | Unspecified fracture of shaft of left femur, initial encounter for open fracture type I or II | | |
| S72.302C | Unspecified fracture of shaft of left femur, initial encounter for open fracture type IIIA, IIIB, or IIIC | | |
| S72.309A | Unspecified fracture of shaft of unspecified femur, initial encounter for closed fracture | | |
| S72.309B | Unspecified fracture of shaft of unspecified femur, initial encounter for open fracture type I or II | | |
| S72.309C | Unspecified fracture of shaft of unspecified femur, initial encounter for open fracture type IIIA, IIIB, or IIIC | | |
| S72.321A | Displaced transverse fracture of shaft of right femur, initial encounter for closed fracture | | |
| S72.321B | Displaced transverse fracture of shaft of right femur, initial encounter for open fracture type I or II | | |
| S72.321C | Displaced transverse fracture of shaft of right femur, initial encounter for open fracture type IIIA, IIIB, or IIIC | | |
| S72.322A | Displaced transverse fracture of shaft of left femur, initial encounter for closed fracture | | |

© 2019 Vashi AA et al. *JAMA Network Open.*

| | | | |
|---|---|---|---|
| S72.322B | Displaced transverse fracture of shaft of left femur, initial encounter for open fracture type I or II | | |
| S72.322C | Displaced transverse fracture of shaft of left femur, initial encounter for open fracture type IIIA, IIIB, or IIIC | | |
| S72.323A | Displaced transverse fracture of shaft of unspecified femur, initial encounter for closed fracture | | |
| S72.323B | Displaced transverse fracture of shaft of unspecified femur, initial encounter for open fracture type I or II | | |
| S72.323C | Displaced transverse fracture of shaft of unspecified femur, initial encounter for open fracture type IIIA, IIIB, or IIIC | | |
| S72.324A | Nondisplaced transverse fracture of shaft of right femur, initial encounter for closed fracture | | |
| S72.324B | Nondisplaced transverse fracture of shaft of right femur, initial encounter for open fracture type I or II | | |
| S72.324C | Nondisplaced transverse fracture of shaft of right femur, initial encounter for open fracture type IIIA, IIIB, or IIIC | | |
| S72.325A | Nondisplaced transverse fracture of shaft of left femur, initial encounter for closed fracture | | |
| S72.325B | Nondisplaced transverse fracture of shaft of left femur, initial encounter for open fracture type I or II | | |
| S72.325C | Nondisplaced transverse fracture of shaft of left femur, initial encounter for open fracture type IIIA, IIIB, or IIIC | | |
| S72.326A | Nondisplaced transverse fracture of shaft of unspecified femur, initial encounter for closed fracture | | |
| S72.326B | Nondisplaced transverse fracture of shaft of unspecified femur, initial encounter for open fracture type I or II | | |
| S72.326C | Nondisplaced transverse fracture of shaft of unspecified femur, initial encounter for open fracture type IIIA, IIIB, or IIIC | | |
| S72.331A | Displaced oblique fracture of shaft of right femur, initial encounter for closed fracture | | |
| S72.331B | Displaced oblique fracture of shaft of right femur, initial encounter for open fracture type I or II | | |
| S72.331C | Displaced oblique fracture of shaft of right femur, initial encounter for open fracture type IIIA, IIIB, or IIIC | | |
| S72.332A | Displaced oblique fracture of shaft of left femur, initial encounter for closed fracture | | |

© 2019 Vashi AA et al. *JAMA Network Open.*

| | | | |
|---|---|---|---|
| S72.332B | Displaced oblique fracture of shaft of left femur, initial encounter for open fracture type I or II | | |
| S72.332C | Displaced oblique fracture of shaft of left femur, initial encounter for open fracture type IIIA, IIIB, or IIIC | | |
| S72.333A | Displaced oblique fracture of shaft of unspecified femur, initial encounter for closed fracture | | |
| S72.333B | Displaced oblique fracture of shaft of unspecified femur, initial encounter for open fracture type I or II | | |
| S72.333C | Displaced oblique fracture of shaft of unspecified femur, initial encounter for open fracture type IIIA, IIIB, or IIIC | | |
| S72.334A | Nondisplaced oblique fracture of shaft of right femur, initial encounter for closed fracture | | |
| S72.334B | Nondisplaced oblique fracture of shaft of right femur, initial encounter for open fracture type I or II | | |
| S72.334C | Nondisplaced oblique fracture of shaft of right femur, initial encounter for open fracture type IIIA, IIIB, or IIIC | | |
| S72.335A | Nondisplaced oblique fracture of shaft of left femur, initial encounter for closed fracture | | |
| S72.335B | Nondisplaced oblique fracture of shaft of left femur, initial encounter for open fracture type I or II | | |
| S72.335C | Nondisplaced oblique fracture of shaft of left femur, initial encounter for open fracture type IIIA, IIIB, or IIIC | | |
| S72.336A | Nondisplaced oblique fracture of shaft of unspecified femur, initial encounter for closed fracture | | |
| S72.336B | Nondisplaced oblique fracture of shaft of unspecified femur, initial encounter for open fracture type I or II | | |
| S72.336C | Nondisplaced oblique fracture of shaft of unspecified femur, initial encounter for open fracture type IIIA, IIIB, or IIIC | | |
| S72.341A | Displaced spiral fracture of shaft of right femur, initial encounter for closed fracture | | |
| S72.341B | Displaced spiral fracture of shaft of right femur, initial encounter for open fracture type I or II | | |
| S72.341C | Displaced spiral fracture of shaft of right femur, initial encounter for open fracture type IIIA, IIIB, or IIIC | | |
| S72.342A | Displaced spiral fracture of shaft of left femur, initial encounter for closed fracture | | |
| S72.342B | Displaced spiral fracture of shaft of left femur, initial encounter for open fracture type I or II | | |

© 2019 Vashi AA et al. *JAMA Network Open.*

| | | | |
|---|---|---|---|
| S72.342C | Displaced spiral fracture of shaft of left femur, initial encounter for open fracture type IIIA, IIIB, or IIIC | | |
| S72.343A | Displaced spiral fracture of shaft of unspecified femur, initial encounter for closed fracture | | |
| S72.343B | Displaced spiral fracture of shaft of unspecified femur, initial encounter for open fracture type I or II | | |
| S72.343C | Displaced spiral fracture of shaft of unspecified femur, initial encounter for open fracture type IIIA, IIIB, or IIIC | | |
| S72.344A | Nondisplaced spiral fracture of shaft of right femur, initial encounter for closed fracture | | |
| S72.344B | Nondisplaced spiral fracture of shaft of right femur, initial encounter for open fracture type I or II | | |
| S72.344C | Nondisplaced spiral fracture of shaft of right femur, initial encounter for open fracture type IIIA, IIIB, or IIIC | | |
| S72.345A | Nondisplaced spiral fracture of shaft of left femur, initial encounter for closed fracture | | |
| S72.345B | Nondisplaced spiral fracture of shaft of left femur, initial encounter for open fracture type I or II | | |
| S72.345C | Nondisplaced spiral fracture of shaft of left femur, initial encounter for open fracture type IIIA, IIIB, or IIIC | | |
| S72.346A | Nondisplaced spiral fracture of shaft of unspecified femur, initial encounter for closed fracture | | |
| S72.346B | Nondisplaced spiral fracture of shaft of unspecified femur, initial encounter for open fracture type I or II | | |
| S72.346C | Nondisplaced spiral fracture of shaft of unspecified femur, initial encounter for open fracture type IIIA, IIIB, or IIIC | | |
| S72.351A | Displaced comminuted fracture of shaft of right femur, initial encounter for closed fracture | | |
| S72.351B | Displaced comminuted fracture of shaft of right femur, initial encounter for open fracture type I or II | | |
| S72.351C | Displaced comminuted fracture of shaft of right femur, initial encounter for open fracture type IIIA, IIIB, or IIIC | | |
| S72.352A | Displaced comminuted fracture of shaft of left femur, initial encounter for closed fracture | | |
| S72.352B | Displaced comminuted fracture of shaft of left femur, initial encounter for open fracture type I or II | | |
| S72.352C | Displaced comminuted fracture of shaft of left femur, initial encounter for open fracture type IIIA, IIIB, or IIIC | | |

© 2019 Vashi AA et al. *JAMA Network Open.*

| | | | |
|---|---|---|---|
| S72.353A | Displaced comminuted fracture of shaft of unspecified femur, initial encounter for closed fracture | | |
| S72.353B | Displaced comminuted fracture of shaft of unspecified femur, initial encounter for open fracture type I or II | | |
| S72.353C | Displaced comminuted fracture of shaft of unspecified femur, initial encounter for open fracture type IIIA, IIIB, or IIIC | | |
| S72.354A | Nondisplaced comminuted fracture of shaft of right femur, initial encounter for closed fracture | | |
| S72.354B | Nondisplaced comminuted fracture of shaft of right femur, initial encounter for open fracture type I or II | | |
| S72.354C | Nondisplaced comminuted fracture of shaft of right femur, initial encounter for open fracture type IIIA, IIIB, or IIIC | | |
| S72.355A | Nondisplaced comminuted fracture of shaft of left femur, initial encounter for closed fracture | | |
| S72.355B | Nondisplaced comminuted fracture of shaft of left femur, initial encounter for open fracture type I or II | | |
| S72.355C | Nondisplaced comminuted fracture of shaft of left femur, initial encounter for open fracture type IIIA, IIIB, or IIIC | | |
| S72.356A | Nondisplaced comminuted fracture of shaft of unspecified femur, initial encounter for closed fracture | | |
| S72.356B | Nondisplaced comminuted fracture of shaft of unspecified femur, initial encounter for open fracture type I or II | | |
| S72.356C | Nondisplaced comminuted fracture of shaft of unspecified femur, initial encounter for open fracture type IIIA, IIIB, or IIIC | | |
| S72.361A | Displaced segmental fracture of shaft of right femur, initial encounter for closed fracture | | |
| S72.361B | Displaced segmental fracture of shaft of right femur, initial encounter for open fracture type I or II | | |
| S72.361C | Displaced segmental fracture of shaft of right femur, initial encounter for open fracture type IIIA, IIIB, or IIIC | | |
| S72.362A | Displaced segmental fracture of shaft of left femur, initial encounter for closed fracture | | |
| S72.362B | Displaced segmental fracture of shaft of left femur, initial encounter for open fracture type I or II | | |

© 2019 Vashi AA et al. *JAMA Network Open.*

| | | | |
|---|---|---|---|
| S72.362C | Displaced segmental fracture of shaft of left femur, initial encounter for open fracture type IIIA, IIIB, or IIIC | | |
| S72.363A | Displaced segmental fracture of shaft of unspecified femur, initial encounter for closed fracture | | |
| S72.363B | Displaced segmental fracture of shaft of unspecified femur, initial encounter for open fracture type I or II | | |
| S72.363C | Displaced segmental fracture of shaft of unspecified femur, initial encounter for open fracture type IIIA, IIIB, or IIIC | | |
| S72.364A | Nondisplaced segmental fracture of shaft of right femur, initial encounter for closed fracture | | |
| S72.364B | Nondisplaced segmental fracture of shaft of right femur, initial encounter for open fracture type I or II | | |
| S72.364C | Nondisplaced segmental fracture of shaft of right femur, initial encounter for open fracture type IIIA, IIIB, or IIIC | | |
| S72.365A | Nondisplaced segmental fracture of shaft of left femur, initial encounter for closed fracture | | |
| S72.365B | Nondisplaced segmental fracture of shaft of left femur, initial encounter for open fracture type I or II | | |
| S72.365C | Nondisplaced segmental fracture of shaft of left femur, initial encounter for open fracture type IIIA, IIIB, or IIIC | | |
| S72.366A | Nondisplaced segmental fracture of shaft of unspecified femur, initial encounter for closed fracture | | |
| S72.366B | Nondisplaced segmental fracture of shaft of unspecified femur, initial encounter for open fracture type I or II | | |
| S72.366C | Nondisplaced segmental fracture of shaft of unspecified femur, initial encounter for open fracture type IIIA, IIIB, or IIIC | | |
| S72.391A | Other fracture of shaft of right femur, initial encounter for closed fracture | | |
| S72.391B | Other fracture of shaft of right femur, initial encounter for open fracture type I or II | | |
| S72.391C | Other fracture of shaft of right femur, initial encounter for open fracture type IIIA, IIIB, or IIIC | | |
| S72.392A | Other fracture of shaft of left femur, initial encounter for closed fracture | | |
| S72.392B | Other fracture of shaft of left femur, initial encounter for open fracture type I or II | | |

© 2019 Vashi AA et al. *JAMA Network Open.*

| | | | |
|---|---|---|---|
| S72.392C | Other fracture of shaft of left femur, initial encounter for open fracture type IIIA, IIIB, or IIIC | | |
| S72.399A | Other fracture of shaft of unspecified femur, initial encounter for closed fracture | | |
| S72.399B | Other fracture of shaft of unspecified femur, initial encounter for open fracture type I or II | | |
| S72.399C | Other fracture of shaft of unspecified femur, initial encounter for open fracture type IIIA, IIIB, or IIIC | | |
| S72.401A | Unspecified fracture of lower end of right femur, initial encounter for closed fracture | | |
| S72.401B | Unspecified fracture of lower end of right femur, initial encounter for open fracture type I or II | | |
| S72.401C | Unspecified fracture of lower end of right femur, initial encounter for open fracture type IIIA, IIIB, or IIIC | | |
| S72.402A | Unspecified fracture of lower end of left femur, initial encounter for closed fracture | | |
| S72.402B | Unspecified fracture of lower end of left femur, initial encounter for open fracture type I or II | | |
| S72.402C | Unspecified fracture of lower end of left femur, initial encounter for open fracture type IIIA, IIIB, or IIIC | | |
| S72.409A | Unspecified fracture of lower end of unspecified femur, initial encounter for closed fracture | | |
| S72.409B | Unspecified fracture of lower end of unspecified femur, initial encounter for open fracture type I or II | | |
| S72.409C | Unspecified fracture of lower end of unspecified femur, initial encounter for open fracture type IIIA, IIIB, or IIIC | | |
| S72.411A | Displaced unspecified condyle fracture of lower end of right femur, initial encounter for closed fracture | | |
| S72.411B | Displaced unspecified condyle fracture of lower end of right femur, initial encounter for open fracture type I or II | | |
| S72.411C | Displaced unspecified condyle fracture of lower end of right femur, initial encounter for open fracture type IIIA, IIIB, or IIIC | | |
| S72.412A | Displaced unspecified condyle fracture of lower end of left femur, initial encounter for closed fracture | | |
| S72.412B | Displaced unspecified condyle fracture of lower end of left femur, initial encounter for open fracture type I or II | | |

© 2019 Vashi AA et al. *JAMA Network Open.*

| | | | |
|---|---|---|---|
| S72.412C | Displaced unspecified condyle fracture of lower end of left femur, initial encounter for open fracture type IIIA, IIIB, or IIIC | | |
| S72.413A | Displaced unspecified condyle fracture of lower end of unspecified femur, initial encounter for closed fracture | | |
| S72.413B | Displaced unspecified condyle fracture of lower end of unspecified femur, initial encounter for open fracture type I or II | | |
| S72.413C | Displaced unspecified condyle fracture of lower end of unspecified femur, initial encounter for open fracture type IIIA, IIIB, or IIIC | | |
| S72.414A | Nondisplaced unspecified condyle fracture of lower end of right femur, initial encounter for closed fracture | | |
| S72.414B | Nondisplaced unspecified condyle fracture of lower end of right femur, initial encounter for open fracture type I or II | | |
| S72.414C | Nondisplaced unspecified condyle fracture of lower end of right femur, initial encounter for open fracture type IIIA, IIIB, or IIIC | | |
| S72.415A | Nondisplaced unspecified condyle fracture of lower end of left femur, initial encounter for closed fracture | | |
| S72.415B | Nondisplaced unspecified condyle fracture of lower end of left femur, initial encounter for open fracture type I or II | | |
| S72.415C | Nondisplaced unspecified condyle fracture of lower end of left femur, initial encounter for open fracture type IIIA, IIIB, or IIIC | | |
| S72.416A | Nondisplaced unspecified condyle fracture of lower end of unspecified femur, initial encounter for closed fracture | | |
| S72.416B | Nondisplaced unspecified condyle fracture of lower end of unspecified femur, initial encounter for open fracture type I or II | | |
| S72.416C | Nondisplaced unspecified condyle fracture of lower end of unspecified femur, initial encounter for open fracture type IIIA, IIIB, or IIIC | | |
| S72.421A | Displaced fracture of lateral condyle of right femur, initial encounter for closed fracture | | |
| S72.421B | Displaced fracture of lateral condyle of right femur, initial encounter for open fracture type I or II | | |

© 2019 Vashi AA et al. *JAMA Network Open.*

| | | | |
|---|---|---|---|
| S72.421C | Displaced fracture of lateral condyle of right femur, initial encounter for open fracture type IIIA, IIIB, or IIIC | | |
| S72.422A | Displaced fracture of lateral condyle of left femur, initial encounter for closed fracture | | |
| S72.422B | Displaced fracture of lateral condyle of left femur, initial encounter for open fracture type I or II | | |
| S72.422C | Displaced fracture of lateral condyle of left femur, initial encounter for open fracture type IIIA, IIIB, or IIIC | | |
| S72.423A | Displaced fracture of lateral condyle of unspecified femur, initial encounter for closed fracture | | |
| S72.423B | Displaced fracture of lateral condyle of unspecified femur, initial encounter for open fracture type I or II | | |
| S72.423C | Displaced fracture of lateral condyle of unspecified femur, initial encounter for open fracture type IIIA, IIIB, or IIIC | | |
| S72.424A | Nondisplaced fracture of lateral condyle of right femur, initial encounter for closed fracture | | |
| S72.424B | Nondisplaced fracture of lateral condyle of right femur, initial encounter for open fracture type I or II | | |
| S72.424C | Nondisplaced fracture of lateral condyle of right femur, initial encounter for open fracture type IIIA, IIIB, or IIIC | | |
| S72.425A | Nondisplaced fracture of lateral condyle of left femur, initial encounter for closed fracture | | |
| S72.425B | Nondisplaced fracture of lateral condyle of left femur, initial encounter for open fracture type I or II | | |
| S72.425C | Nondisplaced fracture of lateral condyle of left femur, initial encounter for open fracture type IIIA, IIIB, or IIIC | | |
| S72.426A | Nondisplaced fracture of lateral condyle of unspecified femur, initial encounter for closed fracture | | |
| S72.426B | Nondisplaced fracture of lateral condyle of unspecified femur, initial encounter for open fracture type I or II | | |
| S72.426C | Nondisplaced fracture of lateral condyle of unspecified femur, initial encounter for open fracture type IIIA, IIIB, or IIIC | | |
| S72.431A | Displaced fracture of medial condyle of right femur, initial encounter for closed fracture | | |
| S72.431B | Displaced fracture of medial condyle of right femur, initial encounter for open fracture type I or II | | |

© 2019 Vashi AA et al. *JAMA Network Open.*

| | | | |
|---|---|---|---|
| S72.431C | Displaced fracture of medial condyle of right femur, initial encounter for open fracture type IIIA, IIIB, or IIIC | | |
| S72.432A | Displaced fracture of medial condyle of left femur, initial encounter for closed fracture | | |
| S72.432B | Displaced fracture of medial condyle of left femur, initial encounter for open fracture type I or II | | |
| S72.432C | Displaced fracture of medial condyle of left femur, initial encounter for open fracture type IIIA, IIIB, or IIIC | | |
| S72.433A | Displaced fracture of medial condyle of unspecified femur, initial encounter for closed fracture | | |
| S72.433B | Displaced fracture of medial condyle of unspecified femur, initial encounter for open fracture type I or II | | |
| S72.433C | Displaced fracture of medial condyle of unspecified femur, initial encounter for open fracture type IIIA, IIIB, or IIIC | | |
| S72.434A | Nondisplaced fracture of medial condyle of right femur, initial encounter for closed fracture | | |
| S72.434B | Nondisplaced fracture of medial condyle of right femur, initial encounter for open fracture type I or II | | |
| S72.434C | Nondisplaced fracture of medial condyle of right femur, initial encounter for open fracture type IIIA, IIIB, or IIIC | | |
| S72.435A | Nondisplaced fracture of medial condyle of left femur, initial encounter for closed fracture | | |
| S72.435B | Nondisplaced fracture of medial condyle of left femur, initial encounter for open fracture type I or II | | |
| S72.435C | Nondisplaced fracture of medial condyle of left femur, initial encounter for open fracture type IIIA, IIIB, or IIIC | | |
| S72.436A | Nondisplaced fracture of medial condyle of unspecified femur, initial encounter for closed fracture | | |
| S72.436B | Nondisplaced fracture of medial condyle of unspecified femur, initial encounter for open fracture type I or II | | |
| S72.436C | Nondisplaced fracture of medial condyle of unspecified femur, initial encounter for open fracture type IIIA, IIIB, or IIIC | | |
| S72.441A | Displaced fracture of lower epiphysis (separation) of right femur, initial encounter for closed fracture | | |
| S72.441B | Displaced fracture of lower epiphysis (separation) of right femur, initial encounter for open fracture type I or II | | |

© 2019 Vashi AA et al. *JAMA Network Open.*

| | | | |
|---|---|---|---|
| S72.441C | Displaced fracture of lower epiphysis (separation) of right femur, initial encounter for open fracture type IIIA, IIIB, or IIIC | | |
| S72.442A | Displaced fracture of lower epiphysis (separation) of left femur, initial encounter for closed fracture | | |
| S72.442B | Displaced fracture of lower epiphysis (separation) of left femur, initial encounter for open fracture type I or II | | |
| S72.442C | Displaced fracture of lower epiphysis (separation) of left femur, initial encounter for open fracture type IIIA, IIIB, or IIIC | | |
| S72.443A | Displaced fracture of lower epiphysis (separation) of unspecified femur, initial encounter for closed fracture | | |
| S72.443B | Displaced fracture of lower epiphysis (separation) of unspecified femur, initial encounter for open fracture type I or II | | |
| S72.443C | Displaced fracture of lower epiphysis (separation) of unspecified femur, initial encounter for open fracture type IIIA, IIIB, or IIIC | | |
| S72.444A | Nondisplaced fracture of lower epiphysis (separation) of right femur, initial encounter for closed fracture | | |
| S72.444B | Nondisplaced fracture of lower epiphysis (separation) of right femur, initial encounter for open fracture type I or II | | |
| S72.444C | Nondisplaced fracture of lower epiphysis (separation) of right femur, initial encounter for open fracture type IIIA, IIIB, or IIIC | | |
| S72.445A | Nondisplaced fracture of lower epiphysis (separation) of left femur, initial encounter for closed fracture | | |
| S72.445B | Nondisplaced fracture of lower epiphysis (separation) of left femur, initial encounter for open fracture type I or II | | |
| S72.445C | Nondisplaced fracture of lower epiphysis (separation) of left femur, initial encounter for open fracture type IIIA, IIIB, or IIIC | | |
| S72.446A | Nondisplaced fracture of lower epiphysis (separation) of unspecified femur, initial encounter for closed fracture | | |
| S72.446B | Nondisplaced fracture of lower epiphysis (separation) of unspecified femur, initial encounter for open fracture type I or II | | |

© 2019 Vashi AA et al. *JAMA Network Open.*

| | | | |
|---|---|---|---|
| S72.446C | Nondisplaced fracture of lower epiphysis (separation) of unspecified femur, initial encounter for open fracture type IIIA, IIIB, or IIIC | | |
| S72.451A | Displaced supracondylar fracture without intracondylar extension of lower end of right femur, initial encounter for closed fracture | | |
| S72.451B | Displaced supracondylar fracture without intracondylar extension of lower end of right femur, initial encounter for open fracture type I or II | | |
| S72.451C | Displaced supracondylar fracture without intracondylar extension of lower end of right femur, initial encounter for open fracture type IIIA, IIIB, or IIIC | | |
| S72.452A | Displaced supracondylar fracture without intracondylar extension of lower end of left femur, initial encounter for closed fracture | | |
| S72.452B | Displaced supracondylar fracture without intracondylar extension of lower end of left femur, initial encounter for open fracture type I or II | | |
| S72.452C | Displaced supracondylar fracture without intracondylar extension of lower end of left femur, initial encounter for open fracture type IIIA, IIIB, or IIIC | | |
| S72.453A | Displaced supracondylar fracture without intracondylar extension of lower end of unspecified femur, initial encounter for closed fracture | | |
| S72.453B | Displaced supracondylar fracture without intracondylar extension of lower end of unspecified femur, initial encounter for open fracture type I or II | | |
| S72.453C | Displaced supracondylar fracture without intracondylar extension of lower end of unspecified femur, initial encounter for open fracture type IIIA, IIIB, or IIIC | | |
| S72.454A | Nondisplaced supracondylar fracture without intracondylar extension of lower end of right femur, initial encounter for closed fracture | | |
| S72.454B | Nondisplaced supracondylar fracture without intracondylar extension of lower end of right femur, initial encounter for open fracture type I or II | | |
| S72.454C | Nondisplaced supracondylar fracture without intracondylar extension of lower end of right femur, initial encounter for open fracture type IIIA, IIIB, or IIIC | | |

© 2019 Vashi AA et al. *JAMA Network Open.*

| | | | |
|---|---|---|---|
| S72.455A | Nondisplaced supracondylar fracture without intracondylar extension of lower end of left femur, initial encounter for closed fracture | | |
| S72.455B | Nondisplaced supracondylar fracture without intracondylar extension of lower end of left femur, initial encounter for open fracture type I or II | | |
| S72.455C | Nondisplaced supracondylar fracture without intracondylar extension of lower end of left femur, initial encounter for open fracture type IIIA, IIIB, or IIIC | | |
| S72.456A | Nondisplaced supracondylar fracture without intracondylar extension of lower end of unspecified femur, initial encounter for closed fracture | | |
| S72.456B | Nondisplaced supracondylar fracture without intracondylar extension of lower end of unspecified femur, initial encounter for open fracture type I or II | | |
| S72.456C | Nondisplaced supracondylar fracture without intracondylar extension of lower end of unspecified femur, initial encounter for open fracture type IIIA, IIIB, or IIIC | | |
| S72.461A | Displaced supracondylar fracture with intracondylar extension of lower end of right femur, initial encounter for closed fracture | | |
| S72.461B | Displaced supracondylar fracture with intracondylar extension of lower end of right femur, initial encounter for open fracture type I or II | | |
| S72.461C | Displaced supracondylar fracture with intracondylar extension of lower end of right femur, initial encounter for open fracture type IIIA, IIIB, or IIIC | | |
| S72.462A | Displaced supracondylar fracture with intracondylar extension of lower end of left femur, initial encounter for closed fracture | | |
| S72.462B | Displaced supracondylar fracture with intracondylar extension of lower end of left femur, initial encounter for open fracture type I or II | | |
| S72.462C | Displaced supracondylar fracture with intracondylar extension of lower end of left femur, initial encounter for open fracture type IIIA, IIIB, or IIIC | | |
| S72.463A | Displaced supracondylar fracture with intracondylar extension of lower end of unspecified femur, initial encounter for closed fracture | | |

© 2019 Vashi AA et al. *JAMA Network Open.*

| | | | |
|---|---|---|---|
| S72.463B | Displaced supracondylar fracture with intracondylar extension of lower end of unspecified femur, initial encounter for open fracture type I or II | | |
| S72.463C | Displaced supracondylar fracture with intracondylar extension of lower end of unspecified femur, initial encounter for open fracture type IIIA, IIIB, or IIIC | | |
| S72.464A | Nondisplaced supracondylar fracture with intracondylar extension of lower end of right femur, initial encounter for closed fracture | | |
| S72.464B | Nondisplaced supracondylar fracture with intracondylar extension of lower end of right femur, initial encounter for open fracture type I or II | | |
| S72.464C | Nondisplaced supracondylar fracture with intracondylar extension of lower end of right femur, initial encounter for open fracture type IIIA, IIIB, or IIIC | | |
| S72.465A | Nondisplaced supracondylar fracture with intracondylar extension of lower end of left femur, initial encounter for closed fracture | | |
| S72.465B | Nondisplaced supracondylar fracture with intracondylar extension of lower end of left femur, initial encounter for open fracture type I or II | | |
| S72.465C | Nondisplaced supracondylar fracture with intracondylar extension of lower end of left femur, initial encounter for open fracture type IIIA, IIIB, or IIIC | | |
| S72.466A | Nondisplaced supracondylar fracture with intracondylar extension of lower end of unspecified femur, initial encounter for closed fracture | | |
| S72.466B | Nondisplaced supracondylar fracture with intracondylar extension of lower end of unspecified femur, initial encounter for open fracture type I or II | | |
| S72.466C | Nondisplaced supracondylar fracture with intracondylar extension of lower end of unspecified femur, initial encounter for open fracture type IIIA, IIIB, or IIIC | | |
| S72.471A | Torus fracture of lower end of right femur, initial encounter for closed fracture | | |
| S72.472A | Torus fracture of lower end of left femur, initial encounter for closed fracture | | |
| S72.479A | Torus fracture of lower end of unspecified femur, initial encounter for closed fracture | | |

© 2019 Vashi AA et al. *JAMA Network Open.*

| | | | |
|---|---|---|---|
| S72.491A | Other fracture of lower end of right femur, initial encounter for closed fracture | | |
| S72.491B | Other fracture of lower end of right femur, initial encounter for open fracture type I or II | | |
| S72.491C | Other fracture of lower end of right femur, initial encounter for open fracture type IIIA, IIIB, or IIIC | | |
| S72.492A | Other fracture of lower end of left femur, initial encounter for closed fracture | | |
| S72.492B | Other fracture of lower end of left femur, initial encounter for open fracture type I or II | | |
| S72.492C | Other fracture of lower end of left femur, initial encounter for open fracture type IIIA, IIIB, or IIIC | | |
| S72.499A | Other fracture of lower end of unspecified femur, initial encounter for closed fracture | | |
| S72.499B | Other fracture of lower end of unspecified femur, initial encounter for open fracture type I or II | | |
| S72.499C | Other fracture of lower end of unspecified femur, initial encounter for open fracture type IIIA, IIIB, or IIIC | | |
| S72.8X1A | Other fracture of right femur, initial encounter for closed fracture | | |
| S72.8X1B | Other fracture of right femur, initial encounter for open fracture type I or II | | |
| S72.8X1C | Other fracture of right femur, initial encounter for open fracture type IIIA, IIIB, or IIIC | | |
| S72.8X2A | Other fracture of left femur, initial encounter for closed fracture | | |
| S72.8X2B | Other fracture of left femur, initial encounter for open fracture type I or II | | |
| S72.8X2C | Other fracture of left femur, initial encounter for open fracture type IIIA, IIIB, or IIIC | | |
| S72.8X9A | Other fracture of unspecified femur, initial encounter for closed fracture | | |
| S72.8X9B | Other fracture of unspecified femur, initial encounter for open fracture type I or II | | |
| S72.8X9C | Other fracture of unspecified femur, initial encounter for open fracture type IIIA, IIIB, or IIIC | | |
| S72.90XA | Unspecified fracture of unspecified femur, initial encounter for closed fracture | | |

© 2019 Vashi AA et al. *JAMA Network Open.*

| | | | |
|---|---|---|---|
| S72.90XB | Unspecified fracture of unspecified femur, initial encounter for open fracture type I or II | | |
| S72.90XC | Unspecified fracture of unspecified femur, initial encounter for open fracture type IIIA, IIIB, or IIIC | | |
| S72.91XA | Unspecified fracture of right femur, initial encounter for closed fracture | | |
| S72.91XB | Unspecified fracture of right femur, initial encounter for open fracture type I or II | | |
| S72.91XC | Unspecified fracture of right femur, initial encounter for open fracture type IIIA, IIIB, or IIIC | | |
| S72.92XA | Unspecified fracture of left femur, initial encounter for closed fracture | | |
| S72.92XB | Unspecified fracture of left femur, initial encounter for open fracture type I or II | | |
| S72.92XC | Unspecified fracture of left femur, initial encounter for open fracture type IIIA, IIIB, or IIIC | | |

© 2019 Vashi AA et al. *JAMA Network Open.*

## Included and Excluded ICD-10-CM Diagnosis Subcodes for Gastrointestinal Tract Bleeding and/or Perforation Condition Group

| Included ICD-10-CM diagnosis subcodes (n=39) | | Excluded ICD-10-CM diagnosis subcodes (n=27) | |
|---|---|---|---|
| **Subcode** | **Subcode description** | **Subcode** | **Subcode description** |
| K25.0 | Acute gastric ulcer with hemorrhage | K25.3 | Acute gastric ulcer without hemorrhage or perforation |
| K25.1 | Acute gastric ulcer with perforation | K25.7 | Chronic gastric ulcer without hemorrhage or perforation |
| K25.2 | Acute gastric ulcer with both hemorrhage and perforation | K25.9 | Gastric ulcer, unspecified as acute or chronic, without hemorrhage or perforation |
| K25.4 | Chronic or unspecified gastric ulcer with hemorrhage | K26.3 | Acute duodenal ulcer without hemorrhage or perforation |
| K25.5 | Chronic or unspecified gastric ulcer with perforation | K26.7 | Chronic duodenal ulcer without hemorrhage or perforation |
| K25.6 | Chronic or unspecified gastric ulcer with both hemorrhage and perforation | K26.9 | Duodenal ulcer, unspecified as acute or chronic, without hemorrhage or perforation |
| K26.0 | Acute duodenal ulcer with hemorrhage | K27.3 | Acute peptic ulcer, site unspecified, without hemorrhage or perforation |
| K26.1 | Acute duodenal ulcer with perforation | K27.7 | Chronic peptic ulcer, site unspecified, without hemorrhage or perforation |
| K26.2 | Acute duodenal ulcer with both hemorrhage and perforation | K27.9 | Peptic ulcer, site unspecified, unspecified as acute or chronic, without hemorrhage or perforation |
| K26.4 | Chronic or unspecified duodenal ulcer with hemorrhage | K28.3 | Acute gastrojejunal ulcer without hemorrhage or perforation |
| K26.5 | Chronic or unspecified duodenal ulcer with perforation | K28.7 | Chronic gastrojejunal ulcer without hemorrhage or perforation |
| K26.6 | Chronic or unspecified duodenal ulcer with both hemorrhage and perforation | K28.9 | Gastrojejunal ulcer, unspecified as acute or chronic, without hemorrhage or perforation |
| K27.0 | Acute peptic ulcer, site unspecified, with hemorrhage | K29.00 | Acute gastritis without bleeding |
| K27.1 | Acute peptic ulcer, site unspecified, with perforation | K29.20 | Alcoholic gastritis without bleeding |
| K27.2 | Acute peptic ulcer, site unspecified, with both hemorrhage and perforation | K29.30 | Chronic superficial gastritis without bleeding |
| K27.4 | Chronic or unspecified peptic ulcer, site unspecified, with hemorrhage | K29.40 | Chronic atrophic gastritis without bleeding |
| K27.5 | Chronic or unspecified peptic ulcer, site unspecified, with perforation | K29.50 | Unspecified chronic gastritis without bleeding |
| K27.6 | Chronic or unspecified peptic ulcer, site unspecified, with both hemorrhage and perforation | K29.60 | Other gastritis without bleeding |
| K28.0 | Acute gastrojejunal ulcer with hemorrhage | K29.70 | Gastritis, unspecified, without bleeding |
| K28.1 | Acute gastrojejunal ulcer with perforation | K29.80 | Duodenitis without bleeding |

© 2019 Vashi AA et al. *JAMA Network Open.*

| | | | |
|---|---|---|---|
| K28.2 | Acute gastrojejunal ulcer with both hemorrhage and perforation | K29.90 | Gastroduodenitis, unspecified, without bleeding |
| K28.4 | Chronic or unspecified gastrojejunal ulcer with hemorrhage | K55.1 | Chronic vascular disorders of intestine |
| K28.5 | Chronic or unspecified gastrojejunal ulcer with perforation | K55.20 | Angiodysplasia of colon without hemorrhage |
| K28.6 | Chronic or unspecified gastrojejunal ulcer with both hemorrhage and perforation | K55.8 | Other vascular disorders of intestine |
| K29.01 | Acute gastritis with bleeding | K92.81 | Gastrointestinal mucositis (ulcerative) |
| K29.21 | Alcoholic gastritis with bleeding | K92.89 | Other specified diseases of the digestive system |
| K29.31 | Chronic superficial gastritis with bleeding | K92.9 | Disease of digestive system, unspecified |
| K29.41 | Chronic atrophic gastritis with bleeding | | |
| K29.51 | Unspecified chronic gastritis with bleeding | | |
| K29.61 | Other gastritis with bleeding | | |
| K29.71 | Gastritis, unspecified, with bleeding | | |
| K29.81 | Duodenitis with bleeding | | |
| K29.91 | Gastroduodenitis, unspecified, with bleeding | | |
| K55.0 | Acute vascular disorders of intestine | | |
| K55.21 | Angiodysplasia of colon with hemorrhage | | |
| K55.9 | Vascular disorder of intestine, unspecified | | |
| K92.0 | Hematemesis | | |
| K92.1 | Melena | | |
| K92.2 | Gastrointestinal hemorrhage, unspecified | | |

© 2019 Vashi AA et al. *JAMA Network Open.*

## Included and Excluded ICD-10-CM Diagnosis Subcodes for Heart Failure Condition Group

| Included ICD-10-CM diagnosis subcodes (n=14) | | Excluded ICD-10-CM diagnosis subcodes (n=0) | |
|---|---|---|---|
| **Subcode** | **Subcode description** | **Subcode** | **Subcode description** |
| I50.1 | Left ventricular failure | | |
| I50.20 | Unspecified systolic (congestive) heart failure | | |
| I50.21 | Acute systolic (congestive) heart failure | | |
| I50.22 | Chronic systolic (congestive) heart failure | | |
| I50.23 | Acute on chronic systolic (congestive) heart failure | | |
| I50.30 | Unspecified diastolic (congestive) heart failure | | |
| I50.31 | Acute diastolic (congestive) heart failure | | |
| I50.32 | Chronic diastolic (congestive) heart failure | | |
| I50.33 | Acute on chronic diastolic (congestive) heart failure | | |
| I50.40 | Unspecified combined systolic (congestive) and diastolic (congestive) heart failure | | |
| I50.41 | Acute combined systolic (congestive) and diastolic (congestive) heart failure | | |
| I50.42 | Chronic combined systolic (congestive) and diastolic (congestive) heart failure | | |
| I50.43 | Acute on chronic combined systolic (congestive) and diastolic (congestive) heart failure | | |
| I50.9 | Heart failure, unspecified | | |

© 2019 Vashi AA et al. *JAMA Network Open.*

## Included and Excluded ICD-10-CM Diagnosis Subcodes for Infectious Fasciitis Condition Group

| Included ICD-10-CM diagnosis subcodes (n=2) | | Excluded ICD-10-CM diagnosis subcodes (n=5) | |
|---|---|---|---|
| **Subcode** | **Subcode description** | **Subcode** | **Subcode description** |
| M72.6 | Necrotizing fasciitis | M72.0 | Palmar fascial fibromatosis [Dupuytren] |
| M72.8 | Other fibroblastic disorders | M72.1 | Knuckle pads |
| | | M72.2 | Plantar fascial fibromatosis |
| | | M72.4 | Pseudosarcomatous fibromatosis |
| | | M72.9 | Fibroblastic disorder, unspecified |

© 2019 Vashi AA et al. *JAMA Network Open.*

## Included and Excluded ICD-10-CM Diagnosis Subcodes for Intracranial Hemorrhage Condition Group

| Included ICD-10-CM diagnosis subcodes (n=34) | | Excluded ICD-10-CM diagnosis subcodes (n=1) | |
|---|---|---|---|
| **Subcode** | **Subcode description** | **Subcode** | **Subcode description** |
| I60.01 | Nontraumatic subarachnoid hemorrhage from right carotid siphon and bifurcation | I62.03 | Nontraumatic chronic subdural hemorrhage |
| I60.02 | Nontraumatic subarachnoid hemorrhage from left carotid siphon and bifurcation | | |
| I60.10 | Nontraumatic subarachnoid hemorrhage from unspecified middle cerebral artery | | |
| I60.11 | Nontraumatic subarachnoid hemorrhage from right middle cerebral artery | | |
| I60.12 | Nontraumatic subarachnoid hemorrhage from left middle cerebral artery | | |
| I60.20 | Nontraumatic subarachnoid hemorrhage from unspecified anterior communicating artery | | |
| I60.21 | Nontraumatic subarachnoid hemorrhage from right anterior communicating artery | | |
| I60.22 | Nontraumatic subarachnoid hemorrhage from left anterior communicating artery | | |
| I60.30 | Nontraumatic subarachnoid hemorrhage from unspecified posterior communicating artery | | |
| I60.31 | Nontraumatic subarachnoid hemorrhage from right posterior communicating artery | | |
| I60.32 | Nontraumatic subarachnoid hemorrhage from left posterior communicating artery | | |
| I60.4 | Nontraumatic subarachnoid hemorrhage from basilar artery | | |
| I60.50 | Nontraumatic subarachnoid hemorrhage from unspecified vertebral artery | | |
| I60.51 | Nontraumatic subarachnoid hemorrhage from right vertebral artery | | |
| I60.52 | Nontraumatic subarachnoid hemorrhage from left vertebral artery | | |
| I60.6 | Nontraumatic subarachnoid hemorrhage from other intracranial arteries | | |
| I60.7 | Nontraumatic subarachnoid hemorrhage from unspecified intracranial artery | | |
| I60.8 | Other nontraumatic subarachnoid hemorrhage | | |

© 2019 Vashi AA et al. *JAMA Network Open.*

| | | | |
|---|---|---|---|
| I60.9 | Nontraumatic subarachnoid hemorrhage, unspecified | | |
| I61.0 | Nontraumatic intracerebral hemorrhage in hemisphere, subcortical | | |
| I61.1 | Nontraumatic intracerebral hemorrhage in hemisphere, cortical | | |
| I61.2 | Nontraumatic intracerebral hemorrhage in hemisphere, unspecified | | |
| I61.3 | Nontraumatic intracerebral hemorrhage in brain stem | | |
| I61.4 | Nontraumatic intracerebral hemorrhage in cerebellum | | |
| I61.5 | Nontraumatic intracerebral hemorrhage, intraventricular | | |
| I61.6 | Nontraumatic intracerebral hemorrhage, multiple localized | | |
| I61.8 | Other nontraumatic intracerebral hemorrhage | | |
| I61.9 | Nontraumatic intracerebral hemorrhage, unspecified | | |
| I62.00 | Nontraumatic subdural hemorrhage, unspecified | | |
| I62.01 | Nontraumatic acute subdural hemorrhage | | |
| I62.02 | Nontraumatic subacute subdural hemorrhage | | |
| I62.1 | Nontraumatic extradural hemorrhage | | |
| I62.9 | Nontraumatic intracranial hemorrhage, unspecified | | |

© 2019 Vashi AA et al. *JAMA Network Open.*

## Included and Excluded ICD-10-CM Diagnosis Subcodes for Intracranial Injury Condition Group

| Included ICD-10-CM diagnosis subcodes (n=190) | | Excluded ICD-10-CM diagnosis subcodes (n=0) | |
|---|---|---|---|
| **Subcode** | **Subcode description** | **Subcode** | **Subcode description** |
| S06.0X0A | Concussion without loss of consciousness, initial encounter | | |
| S06.0X1A | Concussion with loss of consciousness of 30 minutes or less, initial encounter | | |
| S06.0X2A | Concussion with loss of consciousness of 31 minutes to 59 minutes, initial encounter | | |
| S06.0X3A | Concussion with loss of consciousness of 1 hour to 5 hours 59 minutes, initial encounter | | |
| S06.0X4A | Concussion with loss of consciousness of 6 hours to 24 hours, initial encounter | | |
| S06.0X5A | Concussion with loss of consciousness greater than 24 hours with return to pre-existing conscious level, initial encounter | | |
| S06.0X6A | Concussion with loss of consciousness greater than 24 hours without return to pre-existing conscious level with patient surviving, initial encounter | | |
| S06.0X7A | Concussion with loss of consciousness of any duration with death due to brain injury prior to regaining consciousness, initial encounter | | |
| S06.0X8A | Concussion with loss of consciousness of any duration with death due to other cause prior to regaining consciousness, initial encounter | | |
| S06.0X9A | Concussion with loss of consciousness of unspecified duration, initial encounter | | |
| S06.1X0A | Traumatic cerebral edema without loss of consciousness, initial encounter | | |
| S06.1X1A | Traumatic cerebral edema with loss of consciousness of 30 minutes or less, initial encounter | | |
| S06.1X2A | Traumatic cerebral edema with loss of consciousness of 31 minutes to 59 minutes, initial encounter | | |
| S06.1X3A | Traumatic cerebral edema with loss of consciousness of 1 hour to 5 hours 59 minutes, initial encounter | | |
| S06.1X4A | Traumatic cerebral edema with loss of consciousness of 6 hours to 24 hours, initial encounter | | |

© 2019 Vashi AA et al. *JAMA Network Open.*

| | | | |
|---|---|---|---|
| S06.1X5A | Traumatic cerebral edema with loss of consciousness greater than 24 hours with return to pre-existing conscious level, initial encounter | | |
| S06.1X6A | Traumatic cerebral edema with loss of consciousness greater than 24 hours without return to pre-existing conscious level with patient surviving, initial encounter | | |
| S06.1X7A | Traumatic cerebral edema with loss of consciousness of any duration with death due to brain injury prior to regaining consciousness, initial encounter | | |
| S06.1X8A | Traumatic cerebral edema with loss of consciousness of any duration with death due to other cause prior to regaining consciousness, initial encounter | | |
| S06.1X9A | Traumatic cerebral edema with loss of consciousness of unspecified duration, initial encounter | | |
| S06.2X0A | Diffuse traumatic brain injury without loss of consciousness, initial encounter | | |
| S06.2X1A | Diffuse traumatic brain injury with loss of consciousness of 30 minutes or less, initial encounter | | |
| S06.2X2A | Diffuse traumatic brain injury with loss of consciousness of 31 minutes to 59 minutes, initial encounter | | |
| S06.2X3A | Diffuse traumatic brain injury with loss of consciousness of 1 hour to 5 hours 59 minutes, initial encounter | | |
| S06.2X4A | Diffuse traumatic brain injury with loss of consciousness of 6 hours to 24 hours, initial encounter | | |
| S06.2X5A | Diffuse traumatic brain injury with loss of consciousness greater than 24 hours with return to pre-existing conscious levels, initial encounter | | |
| S06.2X6A | Diffuse traumatic brain injury with loss of consciousness greater than 24 hours without return to pre-existing conscious level with patient surviving, initial encounter | | |
| S06.2X7A | Diffuse traumatic brain injury with loss of consciousness of any duration with death due to brain injury prior to regaining consciousness, initial encounter | | |
| S06.2X8A | Diffuse traumatic brain injury with loss of consciousness of any duration with death due to other cause prior to regaining consciousness, initial encounter | | |
| S06.2X9A | Diffuse traumatic brain injury with loss of consciousness of unspecified duration, initial encounter | | |

© 2019 Vashi AA et al. *JAMA Network Open.*

| | | | |
|---|---|---|---|
| S06.300A | Unspecified focal traumatic brain injury without loss of consciousness, initial encounter | | |
| S06.301A | Unspecified focal traumatic brain injury with loss of consciousness of 30 minutes or less, initial encounter | | |
| S06.302A | Unspecified focal traumatic brain injury with loss of consciousness of 31 minutes to 59 minutes, initial encounter | | |
| S06.303A | Unspecified focal traumatic brain injury with loss of consciousness of 1 hour to 5 hours 59 minutes, initial encounter | | |
| S06.304A | Unspecified focal traumatic brain injury with loss of consciousness of 6 hours to 24 hours, initial encounter | | |
| S06.305A | Unspecified focal traumatic brain injury with loss of consciousness greater than 24 hours with return to pre-existing conscious level, initial encounter | | |
| S06.306A | Unspecified focal traumatic brain injury with loss of consciousness greater than 24 hours without return to pre-existing conscious level with patient surviving, initial encounter | | |
| S06.307A | Unspecified focal traumatic brain injury with loss of consciousness of any duration with death due to brain injury prior to regaining consciousness, initial encounter | | |
| S06.308A | Unspecified focal traumatic brain injury with loss of consciousness of any duration with death due to other cause prior to regaining consciousness, initial encounter | | |
| S06.309A | Unspecified focal traumatic brain injury with loss of consciousness of unspecified duration, initial encounter | | |
| S06.310A | Contusion and laceration of right cerebrum without loss of consciousness, initial encounter | | |
| S06.311A | Contusion and laceration of right cerebrum with loss of consciousness of 30 minutes or less, initial encounter | | |
| S06.312A | Contusion and laceration of right cerebrum with loss of consciousness of 31 minutes to 59 minutes, initial encounter | | |
| S06.313A | Contusion and laceration of right cerebrum with loss of consciousness of 1 hour to 5 hours 59 minutes, initial encounter | | |
| S06.314A | Contusion and laceration of right cerebrum with loss of consciousness of 6 hours to 24 hours, initial encounter | | |

© 2019 Vashi AA et al. *JAMA Network Open.*

| | | | |
|---|---|---|---|
| S06.315A | Contusion and laceration of right cerebrum with loss of consciousness greater than 24 hours with return to pre-existing conscious level, initial encounter | | |
| S06.316A | Contusion and laceration of right cerebrum with loss of consciousness greater than 24 hours without return to pre-existing conscious level with patient surviving, initial encounter | | |
| S06.317A | Contusion and laceration of right cerebrum with loss of consciousness of any duration with death due to brain injury prior to regaining consciousness, initial encounter | | |
| S06.318A | Contusion and laceration of right cerebrum with loss of consciousness of any duration with death due to other cause prior to regaining consciousness, initial encounter | | |
| S06.319A | Contusion and laceration of right cerebrum with loss of consciousness of unspecified duration, initial encounter | | |
| S06.320A | Contusion and laceration of left cerebrum without loss of consciousness, initial encounter | | |
| S06.321A | Contusion and laceration of left cerebrum with loss of consciousness of 30 minutes or less, initial encounter | | |
| S06.322A | Contusion and laceration of left cerebrum with loss of consciousness of 31 minutes to 59 minutes, initial encounter | | |
| S06.323A | Contusion and laceration of left cerebrum with loss of consciousness of 1 hour to 5 hours 59 minutes, initial encounter | | |
| S06.324A | Contusion and laceration of left cerebrum with loss of consciousness of 6 hours to 24 hours, initial encounter | | |
| S06.325A | Contusion and laceration of left cerebrum with loss of consciousness greater than 24 hours with return to pre-existing conscious level, initial encounter | | |
| S06.326A | Contusion and laceration of left cerebrum with loss of consciousness greater than 24 hours without return to pre-existing conscious level with patient surviving, initial encounter | | |
| S06.327A | Contusion and laceration of left cerebrum with loss of consciousness of any duration with death due to brain injury prior to regaining consciousness, initial encounter | | |
| S06.328A | Contusion and laceration of left cerebrum with loss of consciousness of any duration with death due to other cause prior to regaining consciousness, initial encounter | | |

© 2019 Vashi AA et al. *JAMA Network Open.*

| | | | |
|---|---|---|---|
| S06.329A | Contusion and laceration of left cerebrum with loss of consciousness of unspecified duration, initial encounter | | |
| S06.330A | Contusion and laceration of cerebrum, unspecified, without loss of consciousness, initial encounter | | |
| S06.331A | Contusion and laceration of cerebrum, unspecified, with loss of consciousness of 30 minutes or less, initial encounter | | |
| S06.332A | Contusion and laceration of cerebrum, unspecified, with loss of consciousness of 31 minutes to 59 minutes, initial encounter | | |
| S06.333A | Contusion and laceration of cerebrum, unspecified, with loss of consciousness of 1 hour to 5 hours 59 minutes, initial encounter | | |
| S06.334A | Contusion and laceration of cerebrum, unspecified, with loss of consciousness of 6 hours to 24 hours, initial encounter | | |
| S06.335A | Contusion and laceration of cerebrum, unspecified, with loss of consciousness greater than 24 hours with return to pre-existing conscious level, initial encounter | | |
| S06.336A | Contusion and laceration of cerebrum, unspecified, with loss of consciousness greater than 24 hours without return to pre-existing conscious level with patient surviving, initial encounter | | |
| S06.337A | Contusion and laceration of cerebrum, unspecified, with loss of consciousness of any duration with death due to brain injury prior to regaining consciousness, initial encounter | | |
| S06.338A | Contusion and laceration of cerebrum, unspecified, with loss of consciousness of any duration with death due to other cause prior to regaining consciousness, initial encounter | | |
| S06.339A | Contusion and laceration of cerebrum, unspecified, with loss of consciousness of unspecified duration, initial encounter | | |
| S06.340A | Traumatic hemorrhage of right cerebrum without loss of consciousness, initial encounter | | |
| S06.341A | Traumatic hemorrhage of right cerebrum with loss of consciousness of 30 minutes or less, initial encounter | | |

© 2019 Vashi AA et al. *JAMA Network Open.*

| | | | |
|---|---|---|---|
| S06.342A | Traumatic hemorrhage of right cerebrum with loss of consciousness of 31 minutes to 59 minutes, initial encounter | | |
| S06.343A | Traumatic hemorrhage of right cerebrum with loss of consciousness of 1 hours to 5 hours 59 minutes, initial encounter | | |
| S06.344A | Traumatic hemorrhage of right cerebrum with loss of consciousness of 6 hours to 24 hours, initial encounter | | |
| S06.345A | Traumatic hemorrhage of right cerebrum with loss of consciousness greater than 24 hours with return to pre-existing conscious level, initial encounter | | |
| S06.346A | Traumatic hemorrhage of right cerebrum with loss of consciousness greater than 24 hours without return to pre-existing conscious level with patient surviving, initial encounter | | |
| S06.347A | Traumatic hemorrhage of right cerebrum with loss of consciousness of any duration with death due to brain injury prior to regaining consciousness, initial encounter | | |
| S06.348A | Traumatic hemorrhage of right cerebrum with loss of consciousness of any duration with death due to other cause prior to regaining consciousness, initial encounter | | |
| S06.349A | Traumatic hemorrhage of right cerebrum with loss of consciousness of unspecified duration, initial encounter | | |
| S06.350A | Traumatic hemorrhage of left cerebrum without loss of consciousness, initial encounter | | |
| S06.351A | Traumatic hemorrhage of left cerebrum with loss of consciousness of 30 minutes or less, initial encounter | | |
| S06.352A | Traumatic hemorrhage of left cerebrum with loss of consciousness of 31 minutes to 59 minutes, initial encounter | | |
| S06.353A | Traumatic hemorrhage of left cerebrum with loss of consciousness of 1 hours to 5 hours 59 minutes, initial encounter | | |
| S06.354A | Traumatic hemorrhage of left cerebrum with loss of consciousness of 6 hours to 24 hours, initial encounter | | |
| S06.355A | Traumatic hemorrhage of left cerebrum with loss of consciousness greater than 24 hours with return to pre-existing conscious level, initial encounter | | |
| S06.356A | Traumatic hemorrhage of left cerebrum with loss of consciousness greater than 24 hours without return to | | |

© 2019 Vashi AA et al. *JAMA Network Open.*

| | | | |
|---|---|---|---|
| | pre-existing conscious level with patient surviving, initial encounter | | |
| S06.357A | Traumatic hemorrhage of left cerebrum with loss of consciousness of any duration with death due to brain injury prior to regaining consciousness, initial encounter | | |
| S06.358A | Traumatic hemorrhage of left cerebrum with loss of consciousness of any duration with death due to other cause prior to regaining consciousness, initial encounter | | |
| S06.359A | Traumatic hemorrhage of left cerebrum with loss of consciousness of unspecified duration, initial encounter | | |
| S06.360A | Traumatic hemorrhage of cerebrum, unspecified, without loss of consciousness, initial encounter | | |
| S06.361A | Traumatic hemorrhage of cerebrum, unspecified, with loss of consciousness of 30 minutes or less, initial encounter | | |
| S06.362A | Traumatic hemorrhage of cerebrum, unspecified, with loss of consciousness of 31 minutes to 59 minutes, initial encounter | | |
| S06.363A | Traumatic hemorrhage of cerebrum, unspecified, with loss of consciousness of 1 hours to 5 hours 59 minutes, initial encounter | | |
| S06.364A | Traumatic hemorrhage of cerebrum, unspecified, with loss of consciousness of 6 hours to 24 hours, initial encounter | | |
| S06.365A | Traumatic hemorrhage of cerebrum, unspecified, with loss of consciousness greater than 24 hours with return to pre-existing conscious level, initial encounter | | |
| S06.366A | Traumatic hemorrhage of cerebrum, unspecified, with loss of consciousness greater than 24 hours without return to pre-existing conscious level with patient surviving, initial encounter | | |
| S06.367A | Traumatic hemorrhage of cerebrum, unspecified, with loss of consciousness of any duration with death due to brain injury prior to regaining consciousness, initial encounter | | |
| S06.368A | Traumatic hemorrhage of cerebrum, unspecified, with loss of consciousness of any duration with death due to other cause prior to regaining consciousness, initial encounter | | |

© 2019 Vashi AA et al. *JAMA Network Open.*

| | | | |
|---|---|---|---|
| S06.369A | Traumatic hemorrhage of cerebrum, unspecified, with loss of consciousness of unspecified duration, initial encounter | | |
| S06.370A | Contusion, laceration, and hemorrhage of cerebellum without loss of consciousness, initial encounter | | |
| S06.371A | Contusion, laceration, and hemorrhage of cerebellum with loss of consciousness of 30 minutes or less, initial encounter | | |
| S06.372A | Contusion, laceration, and hemorrhage of cerebellum with loss of consciousness of 31 minutes to 59 minutes, initial encounter | | |
| S06.373A | Contusion, laceration, and hemorrhage of cerebellum with loss of consciousness of 1 hour to 5 hours 59 minutes, initial encounter | | |
| S06.374A | Contusion, laceration, and hemorrhage of cerebellum with loss of consciousness of 6 hours to 24 hours, initial encounter | | |
| S06.375A | Contusion, laceration, and hemorrhage of cerebellum with loss of consciousness greater than 24 hours with return to pre-existing conscious level, initial encounter | | |
| S06.376A | Contusion, laceration, and hemorrhage of cerebellum with loss of consciousness greater than 24 hours without return to pre-existing conscious level with patient surviving, initial encounter | | |
| S06.377A | Contusion, laceration, and hemorrhage of cerebellum with loss of consciousness of any duration with death due to brain injury prior to regaining consciousness, initial encounter | | |
| S06.378A | Contusion, laceration, and hemorrhage of cerebellum with loss of consciousness of any duration with death due to other cause prior to regaining consciousness, initial encounter | | |
| S06.379A | Contusion, laceration, and hemorrhage of cerebellum with loss of consciousness of unspecified duration, initial encounter | | |
| S06.380A | Contusion, laceration, and hemorrhage of brainstem without loss of consciousness, initial encounter | | |
| S06.381A | Contusion, laceration, and hemorrhage of brainstem with loss of consciousness of 30 minutes or less, initial encounter | | |

© 2019 Vashi AA et al. *JAMA Network Open.*

| | | | |
|---|---|---|---|
| S06.382A | Contusion, laceration, and hemorrhage of brainstem with loss of consciousness of 31 minutes to 59 minutes, initial encounter | | |
| S06.383A | Contusion, laceration, and hemorrhage of brainstem with loss of consciousness of 1 hour to 5 hours 59 minutes, initial encounter | | |
| S06.384A | Contusion, laceration, and hemorrhage of brainstem with loss of consciousness of 6 hours to 24 hours, initial encounter | | |
| S06.385A | Contusion, laceration, and hemorrhage of brainstem with loss of consciousness greater than 24 hours with return to pre-existing conscious level, initial encounter | | |
| S06.386A | Contusion, laceration, and hemorrhage of brainstem with loss of consciousness greater than 24 hours without return to pre-existing conscious level with patient surviving, initial encounter | | |
| S06.387A | Contusion, laceration, and hemorrhage of brainstem with loss of consciousness of any duration with death due to brain injury prior to regaining consciousness, initial encounter | | |
| S06.388A | Contusion, laceration, and hemorrhage of brainstem with loss of consciousness of any duration with death due to other cause prior to regaining consciousness, initial encounter | | |
| S06.389A | Contusion, laceration, and hemorrhage of brainstem with loss of consciousness of unspecified duration, initial encounter | | |
| S06.4X0A | Epidural hemorrhage without loss of consciousness, initial encounter | | |
| S06.4X1A | Epidural hemorrhage with loss of consciousness of 30 minutes or less, initial encounter | | |
| S06.4X2A | Epidural hemorrhage with loss of consciousness of 31 minutes to 59 minutes, initial encounter | | |
| S06.4X3A | Epidural hemorrhage with loss of consciousness of 1 hour to 5 hours 59 minutes, initial encounter | | |
| S06.4X4A | Epidural hemorrhage with loss of consciousness of 6 hours to 24 hours, initial encounter | | |
| S06.4X5A | Epidural hemorrhage with loss of consciousness greater than 24 hours with return to pre-existing conscious level, initial encounter | | |

© 2019 Vashi AA et al. *JAMA Network Open.*

| | | | |
|---|---|---|---|
| S06.4X6A | Epidural hemorrhage with loss of consciousness greater than 24 hours without return to pre-existing conscious level with patient surviving, initial encounter | | |
| S06.4X7A | Epidural hemorrhage with loss of consciousness of any duration with death due to brain injury prior to regaining consciousness, initial encounter | | |
| S06.4X8A | Epidural hemorrhage with loss of consciousness of any duration with death due to other causes prior to regaining consciousness, initial encounter | | |
| S06.4X9A | Epidural hemorrhage with loss of consciousness of unspecified duration, initial encounter | | |
| S06.5X0A | Traumatic subdural hemorrhage without loss of consciousness, initial encounter | | |
| S06.5X1A | Traumatic subdural hemorrhage with loss of consciousness of 30 minutes or less, initial encounter | | |
| S06.5X2A | Traumatic subdural hemorrhage with loss of consciousness of 31 minutes to 59 minutes, initial encounter | | |
| S06.5X3A | Traumatic subdural hemorrhage with loss of consciousness of 1 hour to 5 hours 59 minutes, initial encounter | | |
| S06.5X4A | Traumatic subdural hemorrhage with loss of consciousness of 6 hours to 24 hours, initial encounter | | |
| S06.5X5A | Traumatic subdural hemorrhage with loss of consciousness greater than 24 hours with return to pre-existing conscious level, initial encounter | | |
| S06.5X6A | Traumatic subdural hemorrhage with loss of consciousness greater than 24 hours without return to pre-existing conscious level with patient surviving, initial encounter | | |
| S06.5X7A | Traumatic subdural hemorrhage with loss of consciousness of any duration with death due to brain injury before regaining consciousness, initial encounter | | |
| S06.5X8A | Traumatic subdural hemorrhage with loss of consciousness of any duration with death due to other cause before regaining consciousness, initial encounter | | |
| S06.5X9A | Traumatic subdural hemorrhage with loss of consciousness of unspecified duration, initial encounter | | |
| S06.6X0A | Traumatic subarachnoid hemorrhage without loss of consciousness, initial encounter | | |

© 2019 Vashi AA et al. *JAMA Network Open.*

| | | | |
|---|---|---|---|
| S06.6X1A | Traumatic subarachnoid hemorrhage with loss of consciousness of 30 minutes or less, initial encounter | | |
| S06.6X2A | Traumatic subarachnoid hemorrhage with loss of consciousness of 31 minutes to 59 minutes, initial encounter | | |
| S06.6X3A | Traumatic subarachnoid hemorrhage with loss of consciousness of 1 hour to 5 hours 59 minutes, initial encounter | | |
| S06.6X4A | Traumatic subarachnoid hemorrhage with loss of consciousness of 6 hours to 24 hours, initial encounter | | |
| S06.6X5A | Traumatic subarachnoid hemorrhage with loss of consciousness greater than 24 hours with return to pre-existing conscious level, initial encounter | | |
| S06.6X6A | Traumatic subarachnoid hemorrhage with loss of consciousness greater than 24 hours without return to pre-existing conscious level with patient surviving, initial encounter | | |
| S06.6X7A | Traumatic subarachnoid hemorrhage with loss of consciousness of any duration with death due to brain injury prior to regaining consciousness, initial encounter | | |
| S06.6X8A | Traumatic subarachnoid hemorrhage with loss of consciousness of any duration with death due to other cause prior to regaining consciousness, initial encounter | | |
| S06.6X9A | Traumatic subarachnoid hemorrhage with loss of consciousness of unspecified duration, initial encounter | | |
| S06.810A | Injury of right internal carotid artery, intracranial portion, not elsewhere classified without loss of consciousness, initial encounter | | |
| S06.811A | Injury of right internal carotid artery, intracranial portion, not elsewhere classified with loss of consciousness of 30 minutes or less, initial encounter | | |
| S06.812A | Injury of right internal carotid artery, intracranial portion, not elsewhere classified with loss of consciousness of 31 minutes to 59 minutes, initial encounter | | |
| S06.813A | Injury of right internal carotid artery, intracranial portion, not elsewhere classified with loss of consciousness of 1 hour to 5 hours 59 minutes, initial encounter | | |
| S06.814A | Injury of right internal carotid artery, intracranial portion, not elsewhere classified with loss of consciousness of 6 hours to 24 hours, initial encounter | | |

© 2019 Vashi AA et al. *JAMA Network Open.*

| | | | |
|---|---|---|---|
| S06.815A | Injury of right internal carotid artery, intracranial portion, not elsewhere classified with loss of consciousness greater than 24 hours with return to pre-existing conscious level, initial encounter | | |
| S06.816A | Injury of right internal carotid artery, intracranial portion, not elsewhere classified with loss of consciousness greater than 24 hours without return to pre-existing conscious level with patient surviving, initial encounter | | |
| S06.817A | Injury of right internal carotid artery, intracranial portion, not elsewhere classified with loss of consciousness of any duration with death due to brain injury prior to regaining consciousness, initial encounter | | |
| S06.818A | Injury of right internal carotid artery, intracranial portion, not elsewhere classified with loss of consciousness of any duration with death due to other cause prior to regaining consciousness, initial encounter | | |
| S06.819A | Injury of right internal carotid artery, intracranial portion, not elsewhere classified with loss of consciousness of unspecified duration, initial encounter | | |
| S06.820A | Injury of left internal carotid artery, intracranial portion, not elsewhere classified without loss of consciousness, initial encounter | | |
| S06.821A | Injury of left internal carotid artery, intracranial portion, not elsewhere classified with loss of consciousness of 30 minutes or less, initial encounter | | |
| S06.822A | Injury of left internal carotid artery, intracranial portion, not elsewhere classified with loss of consciousness of 31 minutes to 59 minutes, initial encounter | | |
| S06.823A | Injury of left internal carotid artery, intracranial portion, not elsewhere classified with loss of consciousness of 1 hour to 5 hours 59 minutes, initial encounter | | |
| S06.824A | Injury of left internal carotid artery, intracranial portion, not elsewhere classified with loss of consciousness of 6 hours to 24 hours, initial encounter | | |
| S06.825A | Injury of left internal carotid artery, intracranial portion, not elsewhere classified with loss of consciousness greater than 24 hours with return to pre-existing conscious level, initial encounter | | |
| S06.826A | Injury of left internal carotid artery, intracranial portion, not elsewhere classified with loss of consciousness | | |

© 2019 Vashi AA et al. *JAMA Network Open.*

| | | | |
|---|---|---|---|
| | greater than 24 hours without return to pre-existing conscious level with patient surviving, initial encounter | | |
| S06.827A | Injury of left internal carotid artery, intracranial portion, not elsewhere classified with loss of consciousness of any duration with death due to brain injury prior to regaining consciousness, initial encounter | | |
| S06.828A | Injury of left internal carotid artery, intracranial portion, not elsewhere classified with loss of consciousness of any duration with death due to other cause prior to regaining consciousness, initial encounter | | |
| S06.829A | Injury of left internal carotid artery, intracranial portion, not elsewhere classified with loss of consciousness of unspecified duration, initial encounter | | |
| S06.890A | Other specified intracranial injury without loss of consciousness, initial encounter | | |
| S06.891A | Other specified intracranial injury with loss of consciousness of 30 minutes or less, initial encounter | | |
| S06.892A | Other specified intracranial injury with loss of consciousness of 31 minutes to 59 minutes, initial encounter | | |
| S06.893A | Other specified intracranial injury with loss of consciousness of 1 hour to 5 hours 59 minutes, initial encounter | | |
| S06.894A | Other specified intracranial injury with loss of consciousness of 6 hours to 24 hours, initial encounter | | |
| S06.895A | Other specified intracranial injury with loss of consciousness greater than 24 hours with return to pre-existing conscious level, initial encounter | | |
| S06.896A | Other specified intracranial injury with loss of consciousness greater than 24 hours without return to pre-existing conscious level with patient surviving, initial encounter | | |
| S06.897A | Other specified intracranial injury with loss of consciousness of any duration with death due to brain injury prior to regaining consciousness, initial encounter | | |
| S06.898A | Other specified intracranial injury with loss of consciousness of any duration with death due to other cause prior to regaining consciousness, initial encounter | | |
| S06.899A | Other specified intracranial injury with loss of consciousness of unspecified duration, initial encounter | | |

© 2019 Vashi AA et al. *JAMA Network Open.*

| | | | |
|---|---|---|---|
| S06.9X0A | Unspecified intracranial injury without loss of consciousness, initial encounter | | |
| S06.9X1A | Unspecified intracranial injury with loss of consciousness of 30 minutes or less, initial encounter | | |
| S06.9X2A | Unspecified intracranial injury with loss of consciousness of 31 minutes to 59 minutes, initial encounter | | |
| S06.9X3A | Unspecified intracranial injury with loss of consciousness of 1 hour to 5 hours 59 minutes, initial encounter | | |
| S06.9X4A | Unspecified intracranial injury with loss of consciousness of 6 hours to 24 hours, initial encounter | | |
| S06.9X5A | Unspecified intracranial injury with loss of consciousness greater than 24 hours with return to pre-existing conscious level, initial encounter | | |
| S06.9X6A | Unspecified intracranial injury with loss of consciousness greater than 24 hours without return to pre-existing conscious level with patient surviving, initial encounter | | |
| S06.9X7A | Unspecified intracranial injury with loss of consciousness of any duration with death due to brain injury prior to regaining consciousness, initial encounter | | |
| S06.9X8A | Unspecified intracranial injury with loss of consciousness of any duration with death due to other cause prior to regaining consciousness, initial encounter | | |
| S06.9X9A | Unspecified intracranial injury with loss of consciousness of unspecified duration, initial encounter | | |

© 2019 Vashi AA et al. *JAMA Network Open.*

## Included and Excluded ICD-10-CM Diagnosis Subcodes for Meningitis Condition Group

| Included ICD-10-CM diagnosis subcodes (n=19) | | Excluded ICD-10-CM diagnosis subcodes (n=2) | |
|---|---|---|---|
| **Subcode** | **Subcode description** | **Subcode** | **Subcode description** |
| A01.01 | Typhoid meningitis | G03.1 | Chronic meningitis |
| A02.21 | Salmonella meningitis | G03.2 | Benign recurrent meningitis [Mollaret] |
| A20.3 | Plague meningitis | | |
| A32.11 | Listerial meningitis | | |
| A39.0 | Meningococcal meningitis | | |
| A42.81 | Actinomycotic meningitis | | |
| A54.81 | Gonococcal meningitis | | |
| B37.5 | Candidal meningitis | | |
| G00.0 | Hemophilus meningitis | | |
| G00.1 | Pneumococcal meningitis | | |
| G00.2 | Streptococcal meningitis | | |
| G00.3 | Staphylococcal meningitis | | |
| G00.8 | Other bacterial meningitis | | |
| G00.9 | Bacterial meningitis, unspecified | | |
| G01. | Meningitis in bacterial diseases classified elsewhere | | |
| G02. | Meningitis in other infectious and parasitic diseases classified elsewhere | | |
| G03.0 | Nonpyogenic meningitis | | |
| G03.8 | Meningitis due to other specified causes | | |
| G03.9 | Meningitis, unspecified | | |

© 2019 Vashi AA et al. *JAMA Network Open.*

## Included and Excluded ICD-10-CM Diagnosis Subcodes for Moderate-Severe Burns and Corrosions Condition Group

| Included ICD-10-CM diagnosis subcodes (n=548) | | Excluded ICD-10-CM diagnosis subcodes (n=0) | |
|---|---|---|---|
| **Subcode** | **Subcode description** | **Subcode** | **Subcode description** |
| T20.20XA | Burn of second degree of head, face, and neck, unspecified site, initial encounter | | |
| T20.211A | Burn of second degree of right ear [any part, except ear drum], initial encounter | | |
| T20.212A | Burn of second degree of left ear [any part, except ear drum], initial encounter | | |
| T20.219A | Burn of second degree of unspecified ear [any part, except ear drum], initial encounter | | |
| T20.22XA | Burn of second degree of lip(s), initial encounter | | |
| T20.23XA | Burn of second degree of chin, initial encounter | | |
| T20.24XA | Burn of second degree of nose (septum), initial encounter | | |
| T20.25XA | Burn of second degree of scalp [any part], initial encounter | | |
| T20.26XA | Burn of second degree of forehead and cheek, initial encounter | | |
| T20.27XA | Burn of second degree of neck, initial encounter | | |
| T20.29XA | Burn of second degree of multiple sites of head, face, and neck, initial encounter | | |
| T20.30XA | Burn of third degree of head, face, and neck, unspecified site, initial encounter | | |
| T20.311A | Burn of third degree of right ear [any part, except ear drum], initial encounter | | |
| T20.312A | Burn of third degree of left ear [any part, except ear drum], initial encounter | | |
| T20.319A | Burn of third degree of unspecified ear [any part, except ear drum], initial encounter | | |
| T20.32XA | Burn of third degree of lip(s), initial encounter | | |
| T20.33XA | Burn of third degree of chin, initial encounter | | |
| T20.34XA | Burn of third degree of nose (septum), initial encounter | | |
| T20.35XA | Burn of third degree of scalp [any part], initial encounter | | |

© 2019 Vashi AA et al. *JAMA Network Open.*

| | | | |
|---|---|---|---|
| T20.36XA | Burn of third degree of forehead and cheek, initial encounter | | |
| T20.37XA | Burn of third degree of neck, initial encounter | | |
| T20.39XA | Burn of third degree of multiple sites of head, face, and neck, initial encounter | | |
| T20.60XA | Corrosion of second degree of head, face, and neck, unspecified site, initial encounter | | |
| T20.611A | Corrosion of second degree of right ear [any part, except ear drum], initial encounter | | |
| T20.612A | Corrosion of second degree of left ear [any part, except ear drum], initial encounter | | |
| T20.619A | Corrosion of second degree of unspecified ear [any part, except ear drum], initial encounter | | |
| T20.62XA | Corrosion of second degree of lip(s), initial encounter | | |
| T20.63XA | Corrosion of second degree of chin, initial encounter | | |
| T20.64XA | Corrosion of second degree of nose (septum), initial encounter | | |
| T20.65XA | Corrosion of second degree of scalp [any part], initial encounter | | |
| T20.66XA | Corrosion of second degree of forehead and cheek, initial encounter | | |
| T20.67XA | Corrosion of second degree of neck, initial encounter | | |
| T20.69XA | Corrosion of second degree of multiple sites of head, face, and neck, initial encounter | | |
| T20.70XA | Corrosion of third degree of head, face, and neck, unspecified site, initial encounter | | |
| T20.711A | Corrosion of third degree of right ear [any part, except ear drum], initial encounter | | |
| T20.712A | Corrosion of third degree of left ear [any part, except ear drum], initial encounter | | |
| T20.719A | Corrosion of third degree of unspecified ear [any part, except ear drum], initial encounter | | |
| T20.72XA | Corrosion of third degree of lip(s), initial encounter | | |
| T20.73XA | Corrosion of third degree of chin, initial encounter | | |
| T20.74XA | Corrosion of third degree of nose (septum), initial encounter | | |
| T20.75XA | Corrosion of third degree of scalp [any part], initial encounter | | |

© 2019 Vashi AA et al. *JAMA Network Open.*

| | | | |
|---|---|---|---|
| T20.76XA | Corrosion of third degree of forehead and cheek, initial encounter | | |
| T20.77XA | Corrosion of third degree of neck, initial encounter | | |
| T20.79XA | Corrosion of third degree of multiple sites of head, face, and neck, initial encounter | | |
| T21.20XA | Burn of second degree of trunk, unspecified site, initial encounter | | |
| T21.21XA | Burn of second degree of chest wall, initial encounter | | |
| T21.22XA | Burn of second degree of abdominal wall, initial encounter | | |
| T21.23XA | Burn of second degree of upper back, initial encounter | | |
| T21.24XA | Burn of second degree of lower back, initial encounter | | |
| T21.25XA | Burn of second degree of buttock, initial encounter | | |
| T21.26XA | Burn of second degree of male genital region, initial encounter | | |
| T21.27XA | Burn of second degree of female genital region, initial encounter | | |
| T21.29XA | Burn of second degree of other site of trunk, initial encounter | | |
| T21.30XA | Burn of third degree of trunk, unspecified site, initial encounter | | |
| T21.31XA | Burn of third degree of chest wall, initial encounter | | |
| T21.32XA | Burn of third degree of abdominal wall, initial encounter | | |
| T21.33XA | Burn of third degree of upper back, initial encounter | | |
| T21.34XA | Burn of third degree of lower back, initial encounter | | |
| T21.35XA | Burn of third degree of buttock, initial encounter | | |
| T21.36XA | Burn of third degree of male genital region, initial encounter | | |
| T21.37XA | Burn of third degree of female genital region, initial encounter | | |
| T21.39XA | Burn of third degree of other site of trunk, initial encounter | | |
| T21.60XA | Corrosion of second degree of trunk, unspecified site, initial encounter | | |
| T21.61XA | Corrosion of second degree of chest wall, initial encounter | | |
| T21.62XA | Corrosion of second degree of abdominal wall, initial encounter | | |

© 2019 Vashi AA et al. *JAMA Network Open.*

| | | | |
|---|---|---|---|
| T21.63XA | Corrosion of second degree of upper back, initial encounter | | |
| T21.64XA | Corrosion of second degree of lower back, initial encounter | | |
| T21.65XA | Corrosion of second degree of buttock, initial encounter | | |
| T21.66XA | Corrosion of second degree of male genital region, initial encounter | | |
| T21.67XA | Corrosion of second degree of female genital region, initial encounter | | |
| T21.69XA | Corrosion of second degree of other site of trunk, initial encounter | | |
| T21.70XA | Corrosion of third degree of trunk, unspecified site, initial encounter | | |
| T21.71XA | Corrosion of third degree of chest wall, initial encounter | | |
| T21.72XA | Corrosion of third degree of abdominal wall, initial encounter | | |
| T21.73XA | Corrosion of third degree of upper back, initial encounter | | |
| T21.74XA | Corrosion of third degree of lower back, initial encounter | | |
| T21.75XA | Corrosion of third degree of buttock, initial encounter | | |
| T21.76XA | Corrosion of third degree of male genital region, initial encounter | | |
| T21.77XA | Corrosion of third degree of female genital region, initial encounter | | |
| T21.79XA | Corrosion of third degree of other site of trunk, initial encounter | | |
| T22.20XA | Burn of second degree of shoulder and upper limb, except wrist and hand, unspecified site, initial encounter | | |
| T22.211A | Burn of second degree of right forearm, initial encounter | | |
| T22.212A | Burn of second degree of left forearm, initial encounter | | |
| T22.219A | Burn of second degree of unspecified forearm, initial encounter | | |
| T22.221A | Burn of second degree of right elbow, initial encounter | | |
| T22.222A | Burn of second degree of left elbow, initial encounter | | |
| T22.229A | Burn of second degree of unspecified elbow, initial encounter | | |
| T22.231A | Burn of second degree of right upper arm, initial encounter | | |
| T22.232A | Burn of second degree of left upper arm, initial encounter | | |

© 2019 Vashi AA et al. *JAMA Network Open.*

| | | | |
|---|---|---|---|
| T22.239A | Burn of second degree of unspecified upper arm, initial encounter | | |
| T22.241A | Burn of second degree of right axilla, initial encounter | | |
| T22.242A | Burn of second degree of left axilla, initial encounter | | |
| T22.249A | Burn of second degree of unspecified axilla, initial encounter | | |
| T22.251A | Burn of second degree of right shoulder, initial encounter | | |
| T22.252A | Burn of second degree of left shoulder, initial encounter | | |
| T22.259A | Burn of second degree of unspecified shoulder, initial encounter | | |
| T22.261A | Burn of second degree of right scapular region, initial encounter | | |
| T22.262A | Burn of second degree of left scapular region, initial encounter | | |
| T22.269A | Burn of second degree of unspecified scapular region, initial encounter | | |
| T22.291A | Burn of second degree of multiple sites of right shoulder and upper limb, except wrist and hand, initial encounter | | |
| T22.292A | Burn of second degree of multiple sites of left shoulder and upper limb, except wrist and hand, initial encounter | | |
| T22.299A | Burn of second degree of multiple sites of unspecified shoulder and upper limb, except wrist and hand, initial encounter | | |
| T22.30XA | Burn of third degree of shoulder and upper limb, except wrist and hand, unspecified site, initial encounter | | |
| T22.311A | Burn of third degree of right forearm, initial encounter | | |
| T22.312A | Burn of third degree of left forearm, initial encounter | | |
| T22.319A | Burn of third degree of unspecified forearm, initial encounter | | |
| T22.321A | Burn of third degree of right elbow, initial encounter | | |
| T22.322A | Burn of third degree of left elbow, initial encounter | | |
| T22.329A | Burn of third degree of unspecified elbow, initial encounter | | |
| T22.331A | Burn of third degree of right upper arm, initial encounter | | |
| T22.332A | Burn of third degree of left upper arm, initial encounter | | |
| T22.339A | Burn of third degree of unspecified upper arm, initial encounter | | |
| T22.341A | Burn of third degree of right axilla, initial encounter | | |

© 2019 Vashi AA et al. *JAMA Network Open.*

| | | | |
|---|---|---|---|
| T22.342A | Burn of third degree of left axilla, initial encounter | | |
| T22.349A | Burn of third degree of unspecified axilla, initial encounter | | |
| T22.351A | Burn of third degree of right shoulder, initial encounter | | |
| T22.352A | Burn of third degree of left shoulder, initial encounter | | |
| T22.359A | Burn of third degree of unspecified shoulder, initial encounter | | |
| T22.361A | Burn of third degree of right scapular region, initial encounter | | |
| T22.362A | Burn of third degree of left scapular region, initial encounter | | |
| T22.369A | Burn of third degree of unspecified scapular region, initial encounter | | |
| T22.391A | Burn of third degree of multiple sites of right shoulder and upper limb, except wrist and hand, initial encounter | | |
| T22.392A | Burn of third degree of multiple sites of left shoulder and upper limb, except wrist and hand, initial encounter | | |
| T22.399A | Burn of third degree of multiple sites of unspecified shoulder and upper limb, except wrist and hand, initial encounter | | |
| T22.60XA | Corrosion of second degree of shoulder and upper limb, except wrist and hand, unspecified site, initial encounter | | |
| T22.611A | Corrosion of second degree of right forearm, initial encounter | | |
| T22.612A | Corrosion of second degree of left forearm, initial encounter | | |
| T22.619A | Corrosion of second degree of unspecified forearm, initial encounter | | |
| T22.621A | Corrosion of second degree of right elbow, initial encounter | | |
| T22.622A | Corrosion of second degree of left elbow, initial encounter | | |
| T22.629A | Corrosion of second degree of unspecified elbow, initial encounter | | |
| T22.631A | Corrosion of second degree of right upper arm, initial encounter | | |
| T22.632A | Corrosion of second degree of left upper arm, initial encounter | | |

© 2019 Vashi AA et al. *JAMA Network Open.*

| | | | |
|---|---|---|---|
| T22.639A | Corrosion of second degree of unspecified upper arm, initial encounter | | |
| T22.641A | Corrosion of second degree of right axilla, initial encounter | | |
| T22.642A | Corrosion of second degree of left axilla, initial encounter | | |
| T22.649A | Corrosion of second degree of unspecified axilla, initial encounter | | |
| T22.651A | Corrosion of second degree of right shoulder, initial encounter | | |
| T22.652A | Corrosion of second degree of left shoulder, initial encounter | | |
| T22.659A | Corrosion of second degree of unspecified shoulder, initial encounter | | |
| T22.661A | Corrosion of second degree of right scapular region, initial encounter | | |
| T22.662A | Corrosion of second degree of left scapular region, initial encounter | | |
| T22.669A | Corrosion of second degree of unspecified scapular region, initial encounter | | |
| T22.691A | Corrosion of second degree of multiple sites of right shoulder and upper limb, except wrist and hand, initial encounter | | |
| T22.692A | Corrosion of second degree of multiple sites of left shoulder and upper limb, except wrist and hand, initial encounter | | |
| T22.699A | Corrosion of second degree of multiple sites of unspecified shoulder and upper limb, except wrist and hand, initial encounter | | |
| T22.70XA | Corrosion of third degree of shoulder and upper limb, except wrist and hand, unspecified site, initial encounter | | |
| T22.711A | Corrosion of third degree of right forearm, initial encounter | | |
| T22.712A | Corrosion of third degree of left forearm, initial encounter | | |
| T22.719A | Corrosion of third degree of unspecified forearm, initial encounter | | |
| T22.721A | Corrosion of third degree of right elbow, initial encounter | | |
| T22.722A | Corrosion of third degree of left elbow, initial encounter | | |
| T22.729A | Corrosion of third degree of unspecified elbow, initial encounter | | |

© 2019 Vashi AA et al. *JAMA Network Open.*

| | | | |
|---|---|---|---|
| T22.731A | Corrosion of third degree of right upper arm, initial encounter | | |
| T22.732A | Corrosion of third degree of left upper arm, initial encounter | | |
| T22.739A | Corrosion of third degree of unspecified upper arm, initial encounter | | |
| T22.741A | Corrosion of third degree of right axilla, initial encounter | | |
| T22.742A | Corrosion of third degree of left axilla, initial encounter | | |
| T22.749A | Corrosion of third degree of unspecified axilla, initial encounter | | |
| T22.751A | Corrosion of third degree of right shoulder, initial encounter | | |
| T22.752A | Corrosion of third degree of left shoulder, initial encounter | | |
| T22.759A | Corrosion of third degree of unspecified shoulder, initial encounter | | |
| T22.761A | Corrosion of third degree of right scapular region, initial encounter | | |
| T22.762A | Corrosion of third degree of left scapular region, initial encounter | | |
| T22.769A | Corrosion of third degree of unspecified scapular region, initial encounter | | |
| T22.791A | Corrosion of third degree of multiple sites of right shoulder and upper limb, except wrist and hand, initial encounter | | |
| T22.792A | Corrosion of third degree of multiple sites of left shoulder and upper limb, except wrist and hand, initial encounter | | |
| T22.799A | Corrosion of third degree of multiple sites of unspecified shoulder and upper limb, except wrist and hand, initial encounter | | |
| T23.201A | Burn of second degree of right hand, unspecified site, initial encounter | | |
| T23.202A | Burn of second degree of left hand, unspecified site, initial encounter | | |
| T23.209A | Burn of second degree of unspecified hand, unspecified site, initial encounter | | |
| T23.211A | Burn of second degree of right thumb (nail), initial encounter | | |

© 2019 Vashi AA et al. *JAMA Network Open.*

| | | | |
|---|---|---|---|
| T23.212A | Burn of second degree of left thumb (nail), initial encounter | | |
| T23.219A | Burn of second degree of unspecified thumb (nail), initial encounter | | |
| T23.221A | Burn of second degree of single right finger (nail) except thumb, initial encounter | | |
| T23.222A | Burn of second degree of single left finger (nail) except thumb, initial encounter | | |
| T23.229A | Burn of second degree of unspecified single finger (nail) except thumb, initial encounter | | |
| T23.231A | Burn of second degree of multiple right fingers (nail), not including thumb, initial encounter | | |
| T23.232A | Burn of second degree of multiple left fingers (nail), not including thumb, initial encounter | | |
| T23.239A | Burn of second degree of unspecified multiple fingers (nail), not including thumb, initial encounter | | |
| T23.241A | Burn of second degree of multiple right fingers (nail), including thumb, initial encounter | | |
| T23.242A | Burn of second degree of multiple left fingers (nail), including thumb, initial encounter | | |
| T23.249A | Burn of second degree of unspecified multiple fingers (nail), including thumb, initial encounter | | |
| T23.251A | Burn of second degree of right palm, initial encounter | | |
| T23.252A | Burn of second degree of left palm, initial encounter | | |
| T23.259A | Burn of second degree of unspecified palm, initial encounter | | |
| T23.261A | Burn of second degree of back of right hand, initial encounter | | |
| T23.262A | Burn of second degree of back of left hand, initial encounter | | |
| T23.269A | Burn of second degree of back of unspecified hand, initial encounter | | |
| T23.271A | Burn of second degree of right wrist, initial encounter | | |
| T23.272A | Burn of second degree of left wrist, initial encounter | | |
| T23.279A | Burn of second degree of unspecified wrist, initial encounter | | |
| T23.291A | Burn of second degree of multiple sites of right wrist and hand, initial encounter | | |

© 2019 Vashi AA et al. *JAMA Network Open.*

| | | | |
|---|---|---|---|
| T23.292A | Burn of second degree of multiple sites of left wrist and hand, initial encounter | | |
| T23.299A | Burn of second degree of multiple sites of unspecified wrist and hand, initial encounter | | |
| T23.301A | Burn of third degree of right hand, unspecified site, initial encounter | | |
| T23.302A | Burn of third degree of left hand, unspecified site, initial encounter | | |
| T23.309A | Burn of third degree of unspecified hand, unspecified site, initial encounter | | |
| T23.311A | Burn of third degree of right thumb (nail), initial encounter | | |
| T23.312A | Burn of third degree of left thumb (nail), initial encounter | | |
| T23.319A | Burn of third degree of unspecified thumb (nail), initial encounter | | |
| T23.321A | Burn of third degree of single right finger (nail) except thumb, initial encounter | | |
| T23.322A | Burn of third degree of single left finger (nail) except thumb, initial encounter | | |
| T23.329A | Burn of third degree of unspecified single finger (nail) except thumb, initial encounter | | |
| T23.331A | Burn of third degree of multiple right fingers (nail), not including thumb, initial encounter | | |
| T23.332A | Burn of third degree of multiple left fingers (nail), not including thumb, initial encounter | | |
| T23.339A | Burn of third degree of unspecified multiple fingers (nail), not including thumb, initial encounter | | |
| T23.341A | Burn of third degree of multiple right fingers (nail), including thumb, initial encounter | | |
| T23.342A | Burn of third degree of multiple left fingers (nail), including thumb, initial encounter | | |
| T23.349A | Burn of third degree of unspecified multiple fingers (nail), including thumb, initial encounter | | |
| T23.351A | Burn of third degree of right palm, initial encounter | | |
| T23.352A | Burn of third degree of left palm, initial encounter | | |
| T23.359A | Burn of third degree of unspecified palm, initial encounter | | |
| T23.361A | Burn of third degree of back of right hand, initial encounter | | |
| T23.362A | Burn of third degree of back of left hand, initial encounter | | |

© 2019 Vashi AA et al. *JAMA Network Open.*

| | | | |
|---|---|---|---|
| T23.369A | Burn of third degree of back of unspecified hand, initial encounter | | |
| T23.371A | Burn of third degree of right wrist, initial encounter | | |
| T23.372A | Burn of third degree of left wrist, initial encounter | | |
| T23.379A | Burn of third degree of unspecified wrist, initial encounter | | |
| T23.391A | Burn of third degree of multiple sites of right wrist and hand, initial encounter | | |
| T23.392A | Burn of third degree of multiple sites of left wrist and hand, initial encounter | | |
| T23.399A | Burn of third degree of multiple sites of unspecified wrist and hand, initial encounter | | |
| T23.601A | Corrosion of second degree of right hand, unspecified site, initial encounter | | |
| T23.602A | Corrosion of second degree of left hand, unspecified site, initial encounter | | |
| T23.609A | Corrosion of second degree of unspecified hand, unspecified site, initial encounter | | |
| T23.611A | Corrosion of second degree of right thumb (nail), initial encounter | | |
| T23.612A | Corrosion of second degree of left thumb (nail), initial encounter | | |
| T23.619A | Corrosion of second degree of unspecified thumb (nail), initial encounter | | |
| T23.621A | Corrosion of second degree of single right finger (nail) except thumb, initial encounter | | |
| T23.622A | Corrosion of second degree of single left finger (nail) except thumb, initial encounter | | |
| T23.629A | Corrosion of second degree of unspecified single finger (nail) except thumb, initial encounter | | |
| T23.631A | Corrosion of second degree of multiple right fingers (nail), not including thumb, initial encounter | | |
| T23.632A | Corrosion of second degree of multiple left fingers (nail), not including thumb, initial encounter | | |
| T23.639A | Corrosion of second degree of unspecified multiple fingers (nail), not including thumb, initial encounter | | |
| T23.641A | Corrosion of second degree of multiple right fingers (nail), including thumb, initial encounter | | |
| T23.642A | Corrosion of second degree of multiple left fingers (nail), including thumb, initial encounter | | |

© 2019 Vashi AA et al. *JAMA Network Open.*

| | | | |
|---|---|---|---|
| T23.649A | Corrosion of second degree of unspecified multiple fingers (nail), including thumb, initial encounter | | |
| T23.651A | Corrosion of second degree of right palm, initial encounter | | |
| T23.652A | Corrosion of second degree of left palm, initial encounter | | |
| T23.659A | Corrosion of second degree of unspecified palm, initial encounter | | |
| T23.661A | Corrosion of second degree back of right hand, initial encounter | | |
| T23.662A | Corrosion of second degree back of left hand, initial encounter | | |
| T23.669A | Corrosion of second degree back of unspecified hand, initial encounter | | |
| T23.671A | Corrosion of second degree of right wrist, initial encounter | | |
| T23.672A | Corrosion of second degree of left wrist, initial encounter | | |
| T23.679A | Corrosion of second degree of unspecified wrist, initial encounter | | |
| T23.691A | Corrosion of second degree of multiple sites of right wrist and hand, initial encounter | | |
| T23.692A | Corrosion of second degree of multiple sites of left wrist and hand, initial encounter | | |
| T23.699A | Corrosion of second degree of multiple sites of unspecified wrist and hand, initial encounter | | |
| T23.701A | Corrosion of third degree of right hand, unspecified site, initial encounter | | |
| T23.702A | Corrosion of third degree of left hand, unspecified site, initial encounter | | |
| T23.709A | Corrosion of third degree of unspecified hand, unspecified site, initial encounter | | |
| T23.711A | Corrosion of third degree of right thumb (nail), initial encounter | | |
| T23.712A | Corrosion of third degree of left thumb (nail), initial encounter | | |
| T23.719A | Corrosion of third degree of unspecified thumb (nail), initial encounter | | |
| T23.721A | Corrosion of third degree of single right finger (nail) except thumb, initial encounter | | |

© 2019 Vashi AA et al. *JAMA Network Open.*

| | | | |
|---|---|---|---|
| T23.722A | Corrosion of third degree of single left finger (nail) except thumb, initial encounter | | |
| T23.729A | Corrosion of third degree of unspecified single finger (nail) except thumb, initial encounter | | |
| T23.731A | Corrosion of third degree of multiple right fingers (nail), not including thumb, initial encounter | | |
| T23.732A | Corrosion of third degree of multiple left fingers (nail), not including thumb, initial encounter | | |
| T23.739A | Corrosion of third degree of unspecified multiple fingers (nail), not including thumb, initial encounter | | |
| T23.741A | Corrosion of third degree of multiple right fingers (nail), including thumb, initial encounter | | |
| T23.742A | Corrosion of third degree of multiple left fingers (nail), including thumb, initial encounter | | |
| T23.749A | Corrosion of third degree of unspecified multiple fingers (nail), including thumb, initial encounter | | |
| T23.751A | Corrosion of third degree of right palm, initial encounter | | |
| T23.752A | Corrosion of third degree of left palm, initial encounter | | |
| T23.759A | Corrosion of third degree of unspecified palm, initial encounter | | |
| T23.761A | Corrosion of third degree of back of right hand, initial encounter | | |
| T23.762A | Corrosion of third degree of back of left hand, initial encounter | | |
| T23.769A | Corrosion of third degree back of unspecified hand, initial encounter | | |
| T23.771A | Corrosion of third degree of right wrist, initial encounter | | |
| T23.772A | Corrosion of third degree of left wrist, initial encounter | | |
| T23.779A | Corrosion of third degree of unspecified wrist, initial encounter | | |
| T23.791A | Corrosion of third degree of multiple sites of right wrist and hand, initial encounter | | |
| T23.792A | Corrosion of third degree of multiple sites of left wrist and hand, initial encounter | | |
| T23.799A | Corrosion of third degree of multiple sites of unspecified wrist and hand, initial encounter | | |
| T24.201A | Burn of second degree of unspecified site of right lower limb, except ankle and foot, initial encounter | | |

© 2019 Vashi AA et al. *JAMA Network Open.*

| | | | |
|---|---|---|---|
| T24.202A | Burn of second degree of unspecified site of left lower limb, except ankle and foot, initial encounter | | |
| T24.209A | Burn of second degree of unspecified site of unspecified lower limb, except ankle and foot, initial encounter | | |
| T24.211A | Burn of second degree of right thigh, initial encounter | | |
| T24.212A | Burn of second degree of left thigh, initial encounter | | |
| T24.219A | Burn of second degree of unspecified thigh, initial encounter | | |
| T24.221A | Burn of second degree of right knee, initial encounter | | |
| T24.222A | Burn of second degree of left knee, initial encounter | | |
| T24.229A | Burn of second degree of unspecified knee, initial encounter | | |
| T24.231A | Burn of second degree of right lower leg, initial encounter | | |
| T24.232A | Burn of second degree of left lower leg, initial encounter | | |
| T24.239A | Burn of second degree of unspecified lower leg, initial encounter | | |
| T24.291A | Burn of second degree of multiple sites of right lower limb, except ankle and foot, initial encounter | | |
| T24.292A | Burn of second degree of multiple sites of left lower limb, except ankle and foot, initial encounter | | |
| T24.299A | Burn of second degree of multiple sites of unspecified lower limb, except ankle and foot, initial encounter | | |
| T24.301A | Burn of third degree of unspecified site of right lower limb, except ankle and foot, initial encounter | | |
| T24.302A | Burn of third degree of unspecified site of left lower limb, except ankle and foot, initial encounter | | |
| T24.309A | Burn of third degree of unspecified site of unspecified lower limb, except ankle and foot, initial encounter | | |
| T24.311A | Burn of third degree of right thigh, initial encounter | | |
| T24.312A | Burn of third degree of left thigh, initial encounter | | |
| T24.319A | Burn of third degree of unspecified thigh, initial encounter | | |
| T24.321A | Burn of third degree of right knee, initial encounter | | |
| T24.322A | Burn of third degree of left knee, initial encounter | | |
| T24.329A | Burn of third degree of unspecified knee, initial encounter | | |
| T24.331A | Burn of third degree of right lower leg, initial encounter | | |
| T24.332A | Burn of third degree of left lower leg, initial encounter | | |

© 2019 Vashi AA et al. *JAMA Network Open.*

| | | | |
|---|---|---|---|
| T24.339A | Burn of third degree of unspecified lower leg, initial encounter | | |
| T24.391A | Burn of third degree of multiple sites of right lower limb, except ankle and foot, initial encounter | | |
| T24.392A | Burn of third degree of multiple sites of left lower limb, except ankle and foot, initial encounter | | |
| T24.399A | Burn of third degree of multiple sites of unspecified lower limb, except ankle and foot, initial encounter | | |
| T24.601A | Corrosion of second degree of unspecified site of right lower limb, except ankle and foot, initial encounter | | |
| T24.602A | Corrosion of second degree of unspecified site of left lower limb, except ankle and foot, initial encounter | | |
| T24.609A | Corrosion of second degree of unspecified site of unspecified lower limb, except ankle and foot, initial encounter | | |
| T24.611A | Corrosion of second degree of right thigh, initial encounter | | |
| T24.612A | Corrosion of second degree of left thigh, initial encounter | | |
| T24.619A | Corrosion of second degree of unspecified thigh, initial encounter | | |
| T24.621A | Corrosion of second degree of right knee, initial encounter | | |
| T24.622A | Corrosion of second degree of left knee, initial encounter | | |
| T24.629A | Corrosion of second degree of unspecified knee, initial encounter | | |
| T24.631A | Corrosion of second degree of right lower leg, initial encounter | | |
| T24.632A | Corrosion of second degree of left lower leg, initial encounter | | |
| T24.639A | Corrosion of second degree of unspecified lower leg, initial encounter | | |
| T24.691A | Corrosion of second degree of multiple sites of right lower limb, except ankle and foot, initial encounter | | |
| T24.692A | Corrosion of second degree of multiple sites of left lower limb, except ankle and foot, initial encounter | | |
| T24.699A | Corrosion of second degree of multiple sites of unspecified lower limb, except ankle and foot, initial encounter | | |

© 2019 Vashi AA et al. *JAMA Network Open.*

| | | | |
|---|---|---|---|
| T24.701A | Corrosion of third degree of unspecified site of right lower limb, except ankle and foot, initial encounter | | |
| T24.702A | Corrosion of third degree of unspecified site of left lower limb, except ankle and foot, initial encounter | | |
| T24.709A | Corrosion of third degree of unspecified site of unspecified lower limb, except ankle and foot, initial encounter | | |
| T24.711A | Corrosion of third degree of right thigh, initial encounter | | |
| T24.712A | Corrosion of third degree of left thigh, initial encounter | | |
| T24.719A | Corrosion of third degree of unspecified thigh, initial encounter | | |
| T24.721A | Corrosion of third degree of right knee, initial encounter | | |
| T24.722A | Corrosion of third degree of left knee, initial encounter | | |
| T24.729A | Corrosion of third degree of unspecified knee, initial encounter | | |
| T24.731A | Corrosion of third degree of right lower leg, initial encounter | | |
| T24.732A | Corrosion of third degree of left lower leg, initial encounter | | |
| T24.739A | Corrosion of third degree of unspecified lower leg, initial encounter | | |
| T24.791A | Corrosion of third degree of multiple sites of right lower limb, except ankle and foot, initial encounter | | |
| T24.792A | Corrosion of third degree of multiple sites of left lower limb, except ankle and foot, initial encounter | | |
| T24.799A | Corrosion of third degree of multiple sites of unspecified lower limb, except ankle and foot, initial encounter | | |
| T25.211A | Burn of second degree of right ankle, initial encounter | | |
| T25.212A | Burn of second degree of left ankle, initial encounter | | |
| T25.219A | Burn of second degree of unspecified ankle, initial encounter | | |
| T25.221A | Burn of second degree of right foot, initial encounter | | |
| T25.222A | Burn of second degree of left foot, initial encounter | | |
| T25.229A | Burn of second degree of unspecified foot, initial encounter | | |
| T25.231A | Burn of second degree of right toe(s) (nail), initial encounter | | |

© 2019 Vashi AA et al. *JAMA Network Open.*

| | | | |
|---|---|---|---|
| T25.232A | Burn of second degree of left toe(s) (nail), initial encounter | | |
| T25.239A | Burn of second degree of unspecified toe(s) (nail), initial encounter | | |
| T25.291A | Burn of second degree of multiple sites of right ankle and foot, initial encounter | | |
| T25.292A | Burn of second degree of multiple sites of left ankle and foot, initial encounter | | |
| T25.299A | Burn of second degree of multiple sites of unspecified ankle and foot, initial encounter | | |
| T25.311A | Burn of third degree of right ankle, initial encounter | | |
| T25.312A | Burn of third degree of left ankle, initial encounter | | |
| T25.319A | Burn of third degree of unspecified ankle, initial encounter | | |
| T25.321A | Burn of third degree of right foot, initial encounter | | |
| T25.322A | Burn of third degree of left foot, initial encounter | | |
| T25.329A | Burn of third degree of unspecified foot, initial encounter | | |
| T25.331A | Burn of third degree of right toe(s) (nail), initial encounter | | |
| T25.332A | Burn of third degree of left toe(s) (nail), initial encounter | | |
| T25.339A | Burn of third degree of unspecified toe(s) (nail), initial encounter | | |
| T25.391A | Burn of third degree of multiple sites of right ankle and foot, initial encounter | | |
| T25.392A | Burn of third degree of multiple sites of left ankle and foot, initial encounter | | |
| T25.399A | Burn of third degree of multiple sites of unspecified ankle and foot, initial encounter | | |
| T25.611A | Corrosion of second degree of right ankle, initial encounter | | |
| T25.612A | Corrosion of second degree of left ankle, initial encounter | | |
| T25.619A | Corrosion of second degree of unspecified ankle, initial encounter | | |
| T25.621A | Corrosion of second degree of right foot, initial encounter | | |
| T25.622A | Corrosion of second degree of left foot, initial encounter | | |
| T25.629A | Corrosion of second degree of unspecified foot, initial encounter | | |
| T25.631A | Corrosion of second degree of right toe(s) (nail), initial encounter | | |

© 2019 Vashi AA et al. *JAMA Network Open.*

| | | | |
|---|---|---|---|
| T25.632A | Corrosion of second degree of left toe(s) (nail), initial encounter | | |
| T25.639A | Corrosion of second degree of unspecified toe(s) (nail), initial encounter | | |
| T25.691A | Corrosion of second degree of right ankle and foot, initial encounter | | |
| T25.692A | Corrosion of second degree of left ankle and foot, initial encounter | | |
| T25.699A | Corrosion of second degree of unspecified ankle and foot, initial encounter | | |
| T25.711A | Corrosion of third degree of right ankle, initial encounter | | |
| T25.712A | Corrosion of third degree of left ankle, initial encounter | | |
| T25.719A | Corrosion of third degree of unspecified ankle, initial encounter | | |
| T25.721A | Corrosion of third degree of right foot, initial encounter | | |
| T25.722A | Corrosion of third degree of left foot, initial encounter | | |
| T25.729A | Corrosion of third degree of unspecified foot, initial encounter | | |
| T25.731A | Corrosion of third degree of right toe(s) (nail), initial encounter | | |
| T25.732A | Corrosion of third degree of left toe(s) (nail), initial encounter | | |
| T25.739A | Corrosion of third degree of unspecified toe(s) (nail), initial encounter | | |
| T25.791A | Corrosion of third degree of multiple sites of right ankle and foot, initial encounter | | |
| T25.792A | Corrosion of third degree of multiple sites of left ankle and foot, initial encounter | | |
| T25.799A | Corrosion of third degree of multiple sites of unspecified ankle and foot, initial encounter | | |
| T26.00XA | Burn of unspecified eyelid and periocular area, initial encounter | | |
| T26.01XA | Burn of right eyelid and periocular area, initial encounter | | |
| T26.02XA | Burn of left eyelid and periocular area, initial encounter | | |
| T26.10XA | Burn of cornea and conjunctival sac, unspecified eye, initial encounter | | |
| T26.11XA | Burn of cornea and conjunctival sac, right eye, initial encounter | | |

© 2019 Vashi AA et al. *JAMA Network Open.*

| | | | |
|---|---|---|---|
| T26.12XA | Burn of cornea and conjunctival sac, left eye, initial encounter | | |
| T26.20XA | Burn with resulting rupture and destruction of unspecified eyeball, initial encounter | | |
| T26.21XA | Burn with resulting rupture and destruction of right eyeball, initial encounter | | |
| T26.22XA | Burn with resulting rupture and destruction of left eyeball, initial encounter | | |
| T26.30XA | Burns of other specified parts of unspecified eye and adnexa, initial encounter | | |
| T26.31XA | Burns of other specified parts of right eye and adnexa, initial encounter | | |
| T26.32XA | Burns of other specified parts of left eye and adnexa, initial encounter | | |
| T26.40XA | Burn of unspecified eye and adnexa, part unspecified, initial encounter | | |
| T26.41XA | Burn of right eye and adnexa, part unspecified, initial encounter | | |
| T26.42XA | Burn of left eye and adnexa, part unspecified, initial encounter | | |
| T26.50XA | Corrosion of unspecified eyelid and periocular area, initial encounter | | |
| T26.51XA | Corrosion of right eyelid and periocular area, initial encounter | | |
| T26.52XA | Corrosion of left eyelid and periocular area, initial encounter | | |
| T26.60XA | Corrosion of cornea and conjunctival sac, unspecified eye, initial encounter | | |
| T26.61XA | Corrosion of cornea and conjunctival sac, right eye, initial encounter | | |
| T26.62XA | Corrosion of cornea and conjunctival sac, left eye, initial encounter | | |
| T26.70XA | Corrosion with resulting rupture and destruction of unspecified eyeball, initial encounter | | |
| T26.71XA | Corrosion with resulting rupture and destruction of right eyeball, initial encounter | | |
| T26.72XA | Corrosion with resulting rupture and destruction of left eyeball, initial encounter | | |

© 2019 Vashi AA et al. *JAMA Network Open.*

| | | | |
|---|---|---|---|
| T26.80XA | Corrosions of other specified parts of unspecified eye and adnexa, initial encounter | | |
| T26.81XA | Corrosions of other specified parts of right eye and adnexa, initial encounter | | |
| T26.82XA | Corrosions of other specified parts of left eye and adnexa, initial encounter | | |
| T26.90XA | Corrosion of unspecified eye and adnexa, part unspecified, initial encounter | | |
| T26.91XA | Corrosion of right eye and adnexa, part unspecified, initial encounter | | |
| T26.92XA | Corrosion of left eye and adnexa, part unspecified, initial encounter | | |
| T27.0XXA | Burn of larynx and trachea, initial encounter | | |
| T27.1XXA | Burn involving larynx and trachea with lung, initial encounter | | |
| T27.2XXA | Burn of other parts of respiratory tract, initial encounter | | |
| T27.3XXA | Burn of respiratory tract, part unspecified, initial encounter | | |
| T27.4XXA | Corrosion of larynx and trachea, initial encounter | | |
| T27.5XXA | Corrosion involving larynx and trachea with lung, initial encounter | | |
| T27.6XXA | Corrosion of other parts of respiratory tract, initial encounter | | |
| T27.7XXA | Corrosion of respiratory tract, part unspecified, initial encounter | | |
| T28.0XXA | Burn of mouth and pharynx, initial encounter | | |
| T28.1XXA | Burn of esophagus, initial encounter | | |
| T28.2XXA | Burn of other parts of alimentary tract, initial encounter | | |
| T28.3XXA | Burn of internal genitourinary organs, initial encounter | | |
| T28.40XA | Burn of unspecified internal organ, initial encounter | | |
| T28.411A | Burn of right ear drum, initial encounter | | |
| T28.412A | Burn of left ear drum, initial encounter | | |
| T28.419A | Burn of unspecified ear drum, initial encounter | | |
| T28.49XA | Burn of other internal organ, initial encounter | | |
| T28.5XXA | Corrosion of mouth and pharynx, initial encounter | | |
| T28.6XXA | Corrosion of esophagus, initial encounter | | |

© 2019 Vashi AA et al. *JAMA Network Open.*

| | | | |
|---|---|---|---|
| T28.7XXA | Corrosion of other parts of alimentary tract, initial encounter | | |
| T28.8XXA | Corrosion of internal genitourinary organs, initial encounter | | |
| T28.90XA | Corrosions of unspecified internal organs, initial encounter | | |
| T28.911A | Corrosions of right ear drum, initial encounter | | |
| T28.912A | Corrosions of left ear drum, initial encounter | | |
| T28.919A | Corrosions of unspecified ear drum, initial encounter | | |
| T28.99XA | Corrosions of other internal organs, initial encounter | | |
| T31.10 | Burns involving 10-19% of body surface with 0% to 9% third degree burns | | |
| T31.11 | Burns involving 10-19% of body surface with 10-19% third degree burns | | |
| T31.20 | Burns involving 20-29% of body surface with 0% to 9% third degree burns | | |
| T31.21 | Burns involving 20-29% of body surface with 10-19% third degree burns | | |
| T31.22 | Burns involving 20-29% of body surface with 20-29% third degree burns | | |
| T31.30 | Burns involving 30-39% of body surface with 0% to 9% third degree burns | | |
| T31.31 | Burns involving 30-39% of body surface with 10-19% third degree burns | | |
| T31.32 | Burns involving 30-39% of body surface with 20-29% third degree burns | | |
| T31.33 | Burns involving 30-39% of body surface with 30-39% third degree burns | | |
| T31.40 | Burns involving 40-49% of body surface with 0% to 9% third degree burns | | |
| T31.41 | Burns involving 40-49% of body surface with 10-19% third degree burns | | |
| T31.42 | Burns involving 40-49% of body surface with 20-29% third degree burns | | |
| T31.43 | Burns involving 40-49% of body surface with 30-39% third degree burns | | |
| T31.44 | Burns involving 40-49% of body surface with 40-49% third degree burns | | |

© 2019 Vashi AA et al. *JAMA Network Open.*

| | | | |
|---|---|---|---|
| T31.50 | Burns involving 50-59% of body surface with 0% to 9% third degree burns | | |
| T31.51 | Burns involving 50-59% of body surface with 10-19% third degree burns | | |
| T31.52 | Burns involving 50-59% of body surface with 20-29% third degree burns | | |
| T31.53 | Burns involving 50-59% of body surface with 30-39% third degree burns | | |
| T31.54 | Burns involving 50-59% of body surface with 40-49% third degree burns | | |
| T31.55 | Burns involving 50-59% of body surface with 50-59% third degree burns | | |
| T31.60 | Burns involving 60-69% of body surface with 0% to 9% third degree burns | | |
| T31.61 | Burns involving 60-69% of body surface with 10-19% third degree burns | | |
| T31.62 | Burns involving 60-69% of body surface with 20-29% third degree burns | | |
| T31.63 | Burns involving 60-69% of body surface with 30-39% third degree burns | | |
| T31.64 | Burns involving 60-69% of body surface with 40-49% third degree burns | | |
| T31.65 | Burns involving 60-69% of body surface with 50-59% third degree burns | | |
| T31.66 | Burns involving 60-69% of body surface with 60-69% third degree burns | | |
| T31.70 | Burns involving 70-79% of body surface with 0% to 9% third degree burns | | |
| T31.71 | Burns involving 70-79% of body surface with 10-19% third degree burns | | |
| T31.72 | Burns involving 70-79% of body surface with 20-29% third degree burns | | |
| T31.73 | Burns involving 70-79% of body surface with 30-39% third degree burns | | |
| T31.74 | Burns involving 70-79% of body surface with 40-49% third degree burns | | |
| T31.75 | Burns involving 70-79% of body surface with 50-59% third degree burns | | |

© 2019 Vashi AA et al. *JAMA Network Open.*

| | | | |
|---|---|---|---|
| T31.76 | Burns involving 70-79% of body surface with 60-69% third degree burns | | |
| T31.77 | Burns involving 70-79% of body surface with 70-79% third degree burns | | |
| T31.80 | Burns involving 80-89% of body surface with 0% to 9% third degree burns | | |
| T31.81 | Burns involving 80-89% of body surface with 10-19% third degree burns | | |
| T31.82 | Burns involving 80-89% of body surface with 20-29% third degree burns | | |
| T31.83 | Burns involving 80-89% of body surface with 30-39% third degree burns | | |
| T31.84 | Burns involving 80-89% of body surface with 40-49% third degree burns | | |
| T31.85 | Burns involving 80-89% of body surface with 50-59% third degree burns | | |
| T31.86 | Burns involving 80-89% of body surface with 60-69% third degree burns | | |
| T31.87 | Burns involving 80-89% of body surface with 70-79% third degree burns | | |
| T31.88 | Burns involving 80-89% of body surface with 80-89% third degree burns | | |
| T31.90 | Burns involving 90% or more of body surface with 0% to 9% third degree burns | | |
| T31.91 | Burns involving 90% or more of body surface with 10-19% third degree burns | | |
| T31.92 | Burns involving 90% or more of body surface with 20-29% third degree burns | | |
| T31.93 | Burns involving 90% or more of body surface with 30-39% third degree burns | | |
| T31.94 | Burns involving 90% or more of body surface with 40-49% third degree burns | | |
| T31.95 | Burns involving 90% or more of body surface with 50-59% third degree burns | | |
| T31.96 | Burns involving 90% or more of body surface with 60-69% third degree burns | | |
| T31.97 | Burns involving 90% or more of body surface with 70-79% third degree burns | | |

© 2019 Vashi AA et al. *JAMA Network Open.*

| | | | |
|---|---|---|---|
| T31.98 | Burns involving 90% or more of body surface with 80-89% third degree burns | | |
| T31.99 | Burns involving 90% or more of body surface with 90% or more third degree burns | | |
| T32.10 | Corrosions involving 10-19% of body surface with 0% to 9% third degree corrosion | | |
| T32.11 | Corrosions involving 10-19% of body surface with 10-19% third degree corrosion | | |
| T32.20 | Corrosions involving 20-29% of body surface with 0% to 9% third degree corrosion | | |
| T32.21 | Corrosions involving 20-29% of body surface with 10-19% third degree corrosion | | |
| T32.22 | Corrosions involving 20-29% of body surface with 20-29% third degree corrosion | | |
| T32.30 | Corrosions involving 30-39% of body surface with 0% to 9% third degree corrosion | | |
| T32.31 | Corrosions involving 30-39% of body surface with 10-19% third degree corrosion | | |
| T32.32 | Corrosions involving 30-39% of body surface with 20-29% third degree corrosion | | |
| T32.33 | Corrosions involving 30-39% of body surface with 30-39% third degree corrosion | | |
| T32.40 | Corrosions involving 40-49% of body surface with 0% to 9% third degree corrosion | | |
| T32.41 | Corrosions involving 40-49% of body surface with 10-19% third degree corrosion | | |
| T32.42 | Corrosions involving 40-49% of body surface with 20-29% third degree corrosion | | |
| T32.43 | Corrosions involving 40-49% of body surface with 30-39% third degree corrosion | | |
| T32.44 | Corrosions involving 40-49% of body surface with 40-49% third degree corrosion | | |
| T32.50 | Corrosions involving 50-59% of body surface with 0% to 9% third degree corrosion | | |
| T32.51 | Corrosions involving 50-59% of body surface with 10-19% third degree corrosion | | |
| T32.52 | Corrosions involving 50-59% of body surface with 20-29% third degree corrosion | | |

© 2019 Vashi AA et al. *JAMA Network Open.*

| | | | |
|---|---|---|---|
| T32.53 | Corrosions involving 50-59% of body surface with 30-39% third degree corrosion | | |
| T32.54 | Corrosions involving 50-59% of body surface with 40-49% third degree corrosion | | |
| T32.55 | Corrosions involving 50-59% of body surface with 50-59% third degree corrosion | | |
| T32.60 | Corrosions involving 60-69% of body surface with 0% to 9% third degree corrosion | | |
| T32.61 | Corrosions involving 60-69% of body surface with 10-19% third degree corrosion | | |
| T32.62 | Corrosions involving 60-69% of body surface with 20-29% third degree corrosion | | |
| T32.63 | Corrosions involving 60-69% of body surface with 30-39% third degree corrosion | | |
| T32.64 | Corrosions involving 60-69% of body surface with 40-49% third degree corrosion | | |
| T32.65 | Corrosions involving 60-69% of body surface with 50-59% third degree corrosion | | |
| T32.66 | Corrosions involving 60-69% of body surface with 60-69% third degree corrosion | | |
| T32.70 | Corrosions involving 70-79% of body surface with 0% to 9% third degree corrosion | | |
| T32.71 | Corrosions involving 70-79% of body surface with 10-19% third degree corrosion | | |
| T32.72 | Corrosions involving 70-79% of body surface with 20-29% third degree corrosion | | |
| T32.73 | Corrosions involving 70-79% of body surface with 30-39% third degree corrosion | | |
| T32.74 | Corrosions involving 70-79% of body surface with 40-49% third degree corrosion | | |
| T32.75 | Corrosions involving 70-79% of body surface with 50-59% third degree corrosion | | |
| T32.76 | Corrosions involving 70-79% of body surface with 60-69% third degree corrosion | | |
| T32.77 | Corrosions involving 70-79% of body surface with 70-79% third degree corrosion | | |
| T32.80 | Corrosions involving 80-89% of body surface with 0% to 9% third degree corrosion | | |

© 2019 Vashi AA et al. *JAMA Network Open.*

| | | | |
|---|---|---|---|
| T32.81 | Corrosions involving 80-89% of body surface with 10-19% third degree corrosion | | |
| T32.82 | Corrosions involving 80-89% of body surface with 20-29% third degree corrosion | | |
| T32.83 | Corrosions involving 80-89% of body surface with 30-39% third degree corrosion | | |
| T32.84 | Corrosions involving 80-89% of body surface with 40-49% third degree corrosion | | |
| T32.85 | Corrosions involving 80-89% of body surface with 50-59% third degree corrosion | | |
| T32.86 | Corrosions involving 80-89% of body surface with 60-69% third degree corrosion | | |
| T32.87 | Corrosions involving 80-89% of body surface with 70-79% third degree corrosion | | |
| T32.88 | Corrosions involving 80-89% of body surface with 80-89% third degree corrosion | | |
| T32.90 | Corrosions involving 90% or more of body surface with 0% to 9% third degree corrosion | | |
| T32.91 | Corrosions involving 90% or more of body surface with 10-19% third degree corrosion | | |
| T32.92 | Corrosions involving 90% or more of body surface with 20-29% third degree corrosion | | |
| T32.93 | Corrosions involving 90% or more of body surface with 30-39% third degree corrosion | | |
| T32.94 | Corrosions involving 90% or more of body surface with 40-49% third degree corrosion | | |
| T32.95 | Corrosions involving 90% or more of body surface with 50-59% third degree corrosion | | |
| T32.96 | Corrosions involving 90% or more of body surface with 60-69% third degree corrosion | | |
| T32.97 | Corrosions involving 90% or more of body surface with 70-79% third degree corrosion | | |
| T32.98 | Corrosions involving 90% or more of body surface with 80-89% third degree corrosion | | |
| T32.99 | Corrosions involving 90% or more of body surface with 90% or more third degree corrosion | | |

© 2019 Vashi AA et al. *JAMA Network Open.*

## Included and Excluded ICD-10-CM Diagnosis Subcodes for Myocardial Infarction Condition Group

| Included ICD-10-CM diagnosis subcodes (n=9) | | Excluded ICD-10-CM diagnosis subcodes (n=0) | |
|---|---|---|---|
| **Subcode** | **Subcode description** | **Subcode** | **Subcode description** |
| I21.01 | ST elevation (STEMI) myocardial infarction involving left main coronary artery | | |
| I21.02 | ST elevation (STEMI) myocardial infarction involving left anterior descending coronary artery | | |
| I21.09 | ST elevation (STEMI) myocardial infarction involving other coronary artery of anterior wall | | |
| I21.11 | ST elevation (STEMI) myocardial infarction involving right coronary artery | | |
| I21.19 | ST elevation (STEMI) myocardial infarction involving other coronary artery of inferior wall | | |
| I21.21 | ST elevation (STEMI) myocardial infarction involving left circumflex coronary artery | | |
| I21.29 | ST elevation (STEMI) myocardial infarction involving other sites | | |
| I21.3 | ST elevation (STEMI) myocardial infarction of unspecified site | | |
| I21.4 | Non-ST elevation (NSTEMI) myocardial infarction | | |
| | | | |
| | | | |
| | | | |
| | | | |
| | | | |

© 2019 Vashi AA et al. *JAMA Network Open.*

## Included and Excluded ICD-10-CM Diagnosis Subcodes for Neck Injuries Condition Group

| Included ICD-10-CM diagnosis subcodes (n=303) | | Excluded ICD-10-CM diagnosis subcodes (n=6) | |
|---|---|---|---|
| **Subcode** | **Subcode description** | **Subcode** | **Subcode description** |
| S11.011A | Laceration without foreign body of larynx, initial encounter | S14.2XXA | Injury of nerve root of cervical spine, initial encounter |
| S11.012A | Laceration with foreign body of larynx, initial encounter | S14.3XXA | Injury of brachial plexus, initial encounter |
| S11.013A | Puncture wound without foreign body of larynx, initial encounter | S14.4XXA | Injury of peripheral nerves of neck, initial encounter |
| S11.014A | Puncture wound with foreign body of larynx, initial encounter | S14.5XXA | Injury of cervical sympathetic nerves, initial encounter |
| S11.015A | Open bite of larynx, initial encounter | S14.8XXA | Injury of other specified nerves of neck, initial encounter |
| S11.019A | Unspecified open wound of larynx, initial encounter | S16.1XXA | Strain of muscle, fascia and tendon at neck level, initial encounter |
| S11.021A | Laceration without foreign body of trachea, initial encounter | | |
| S11.022A | Laceration with foreign body of trachea, initial encounter | | |
| S11.023A | Puncture wound without foreign body of trachea, initial encounter | | |
| S11.024A | Puncture wound with foreign body of trachea, initial encounter | | |
| S11.025A | Open bite of trachea, initial encounter | | |
| S11.029A | Unspecified open wound of trachea, initial encounter | | |
| S11.031A | Laceration without foreign body of vocal cord, initial encounter | | |
| S11.032A | Laceration with foreign body of vocal cord, initial encounter | | |
| S11.033A | Puncture wound without foreign body of vocal cord, initial encounter | | |
| S11.034A | Puncture wound with foreign body of vocal cord, initial encounter | | |
| S11.035A | Open bite of vocal cord, initial encounter | | |
| S11.039A | Unspecified open wound of vocal cord, initial encounter | | |
| S11.10XA | Unspecified open wound of thyroid gland, initial encounter | | |
| S11.11XA | Laceration without foreign body of thyroid gland, initial encounter | | |

© 2019 Vashi AA et al. *JAMA Network Open.*

| | | | |
|---|---|---|---|
| S11.12XA | Laceration with foreign body of thyroid gland, initial encounter | | |
| S11.13XA | Puncture wound without foreign body of thyroid gland, initial encounter | | |
| S11.14XA | Puncture wound with foreign body of thyroid gland, initial encounter | | |
| S11.15XA | Open bite of thyroid gland, initial encounter | | |
| S11.20XA | Unspecified open wound of pharynx and cervical esophagus, initial encounter | | |
| S11.21XA | Laceration without foreign body of pharynx and cervical esophagus, initial encounter | | |
| S11.22XA | Laceration with foreign body of pharynx and cervical esophagus, initial encounter | | |
| S11.23XA | Puncture wound without foreign body of pharynx and cervical esophagus, initial encounter | | |
| S11.24XA | Puncture wound with foreign body of pharynx and cervical esophagus, initial encounter | | |
| S11.25XA | Open bite of pharynx and cervical esophagus, initial encounter | | |
| S11.80XA | Unspecified open wound of other specified part of neck, initial encounter | | |
| S11.81XA | Laceration without foreign body of other specified part of neck, initial encounter | | |
| S11.82XA | Laceration with foreign body of other specified part of neck, initial encounter | | |
| S11.83XA | Puncture wound without foreign body of other specified part of neck, initial encounter | | |
| S11.84XA | Puncture wound with foreign body of other specified part of neck, initial encounter | | |
| S11.85XA | Open bite of other specified part of neck, initial encounter | | |
| S11.89XA | Other open wound of other specified part of neck, initial encounter | | |
| S11.90XA | Unspecified open wound of unspecified part of neck, initial encounter | | |
| S11.91XA | Laceration without foreign body of unspecified part of neck, initial encounter | | |
| S11.92XA | Laceration with foreign body of unspecified part of neck, initial encounter | | |

© 2019 Vashi AA et al. *JAMA Network Open.*

| | | | |
|---|---|---|---|
| S11.93XA | Puncture wound without foreign body of unspecified part of neck, initial encounter | | |
| S11.94XA | Puncture wound with foreign body of unspecified part of neck, initial encounter | | |
| S11.95XA | Open bite of unspecified part of neck, initial encounter | | |
| S12.000A | Unspecified displaced fracture of first cervical vertebra, initial encounter for closed fracture | | |
| S12.000B | Unspecified displaced fracture of first cervical vertebra, initial encounter for open fracture | | |
| S12.001A | Unspecified nondisplaced fracture of first cervical vertebra, initial encounter for closed fracture | | |
| S12.001B | Unspecified nondisplaced fracture of first cervical vertebra, initial encounter for open fracture | | |
| S12.01XA | Stable burst fracture of first cervical vertebra, initial encounter for closed fracture | | |
| S12.01XB | Stable burst fracture of first cervical vertebra, initial encounter for open fracture | | |
| S12.02XA | Unstable burst fracture of first cervical vertebra, initial encounter for closed fracture | | |
| S12.02XB | Unstable burst fracture of first cervical vertebra, initial encounter for open fracture | | |
| S12.030A | Displaced posterior arch fracture of first cervical vertebra, initial encounter for closed fracture | | |
| S12.030B | Displaced posterior arch fracture of first cervical vertebra, initial encounter for open fracture | | |
| S12.031A | Nondisplaced posterior arch fracture of first cervical vertebra, initial encounter for closed fracture | | |
| S12.031B | Nondisplaced posterior arch fracture of first cervical vertebra, initial encounter for open fracture | | |
| S12.040A | Displaced lateral mass fracture of first cervical vertebra, initial encounter for closed fracture | | |
| S12.040B | Displaced lateral mass fracture of first cervical vertebra, initial encounter for open fracture | | |
| S12.041A | Nondisplaced lateral mass fracture of first cervical vertebra, initial encounter for closed fracture | | |
| S12.041B | Nondisplaced lateral mass fracture of first cervical vertebra, initial encounter for open fracture | | |
| S12.090A | Other displaced fracture of first cervical vertebra, initial encounter for closed fracture | | |

© 2019 Vashi AA et al. *JAMA Network Open.*

| | | | |
|---|---|---|---|
| S12.090B | Other displaced fracture of first cervical vertebra, initial encounter for open fracture | | |
| S12.091A | Other nondisplaced fracture of first cervical vertebra, initial encounter for closed fracture | | |
| S12.091B | Other nondisplaced fracture of first cervical vertebra, initial encounter for open fracture | | |
| S12.100A | Unspecified displaced fracture of second cervical vertebra, initial encounter for closed fracture | | |
| S12.100B | Unspecified displaced fracture of second cervical vertebra, initial encounter for open fracture | | |
| S12.101A | Unspecified nondisplaced fracture of second cervical vertebra, initial encounter for closed fracture | | |
| S12.101B | Unspecified nondisplaced fracture of second cervical vertebra, initial encounter for open fracture | | |
| S12.110A | Anterior displaced Type II dens fracture, initial encounter for closed fracture | | |
| S12.110B | Anterior displaced Type II dens fracture, initial encounter for open fracture | | |
| S12.111A | Posterior displaced Type II dens fracture, initial encounter for closed fracture | | |
| S12.111B | Posterior displaced Type II dens fracture, initial encounter for open fracture | | |
| S12.112A | Nondisplaced Type II dens fracture, initial encounter for closed fracture | | |
| S12.112B | Nondisplaced Type II dens fracture, initial encounter for open fracture | | |
| S12.120A | Other displaced dens fracture, initial encounter for closed fracture | | |
| S12.120B | Other displaced dens fracture, initial encounter for open fracture | | |
| S12.121A | Other nondisplaced dens fracture, initial encounter for closed fracture | | |
| S12.121B | Other nondisplaced dens fracture, initial encounter for open fracture | | |
| S12.130A | Unspecified traumatic displaced spondylolisthesis of second cervical vertebra, initial encounter for closed fracture | | |

© 2019 Vashi AA et al. *JAMA Network Open.*

| | | | |
|---|---|---|---|
| S12.130B | Unspecified traumatic displaced spondylolisthesis of second cervical vertebra, initial encounter for open fracture | | |
| S12.131A | Unspecified traumatic nondisplaced spondylolisthesis of second cervical vertebra, initial encounter for closed fracture | | |
| S12.131B | Unspecified traumatic nondisplaced spondylolisthesis of second cervical vertebra, initial encounter for open fracture | | |
| S12.14XA | Type III traumatic spondylolisthesis of second cervical vertebra, initial encounter for closed fracture | | |
| S12.14XB | Type III traumatic spondylolisthesis of second cervical vertebra, initial encounter for open fracture | | |
| S12.150A | Other traumatic displaced spondylolisthesis of second cervical vertebra, initial encounter for closed fracture | | |
| S12.150B | Other traumatic displaced spondylolisthesis of second cervical vertebra, initial encounter for open fracture | | |
| S12.151A | Other traumatic nondisplaced spondylolisthesis of second cervical vertebra, initial encounter for closed fracture | | |
| S12.151B | Other traumatic nondisplaced spondylolisthesis of second cervical vertebra, initial encounter for open fracture | | |
| S12.190A | Other displaced fracture of second cervical vertebra, initial encounter for closed fracture | | |
| S12.190B | Other displaced fracture of second cervical vertebra, initial encounter for open fracture | | |
| S12.191A | Other nondisplaced fracture of second cervical vertebra, initial encounter for closed fracture | | |
| S12.191B | Other nondisplaced fracture of second cervical vertebra, initial encounter for open fracture | | |
| S12.200A | Unspecified displaced fracture of third cervical vertebra, initial encounter for closed fracture | | |
| S12.200B | Unspecified displaced fracture of third cervical vertebra, initial encounter for open fracture | | |
| S12.201A | Unspecified nondisplaced fracture of third cervical vertebra, initial encounter for closed fracture | | |
| S12.201B | Unspecified nondisplaced fracture of third cervical vertebra, initial encounter for open fracture | | |

© 2019 Vashi AA et al. *JAMA Network Open.*

| | | | |
|---|---|---|---|
| S12.230A | Unspecified traumatic displaced spondylolisthesis of third cervical vertebra, initial encounter for closed fracture | | |
| S12.230B | Unspecified traumatic displaced spondylolisthesis of third cervical vertebra, initial encounter for open fracture | | |
| S12.231A | Unspecified traumatic nondisplaced spondylolisthesis of third cervical vertebra, initial encounter for closed fracture | | |
| S12.231B | Unspecified traumatic nondisplaced spondylolisthesis of third cervical vertebra, initial encounter for open fracture | | |
| S12.24XA | Type III traumatic spondylolisthesis of third cervical vertebra, initial encounter for closed fracture | | |
| S12.24XB | Type III traumatic spondylolisthesis of third cervical vertebra, initial encounter for open fracture | | |
| S12.250A | Other traumatic displaced spondylolisthesis of third cervical vertebra, initial encounter for closed fracture | | |
| S12.250B | Other traumatic displaced spondylolisthesis of third cervical vertebra, initial encounter for open fracture | | |
| S12.251A | Other traumatic nondisplaced spondylolisthesis of third cervical vertebra, initial encounter for closed fracture | | |
| S12.251B | Other traumatic nondisplaced spondylolisthesis of third cervical vertebra, initial encounter for open fracture | | |
| S12.290A | Other displaced fracture of third cervical vertebra, initial encounter for closed fracture | | |
| S12.290B | Other displaced fracture of third cervical vertebra, initial encounter for open fracture | | |
| S12.291A | Other nondisplaced fracture of third cervical vertebra, initial encounter for closed fracture | | |
| S12.291B | Other nondisplaced fracture of third cervical vertebra, initial encounter for open fracture | | |
| S12.300A | Unspecified displaced fracture of fourth cervical vertebra, initial encounter for closed fracture | | |
| S12.300B | Unspecified displaced fracture of fourth cervical vertebra, initial encounter for open fracture | | |
| S12.301A | Unspecified nondisplaced fracture of fourth cervical vertebra, initial encounter for closed fracture | | |
| S12.301B | Unspecified nondisplaced fracture of fourth cervical vertebra, initial encounter for open fracture | | |

© 2019 Vashi AA et al. *JAMA Network Open.*

| | | | |
|---|---|---|---|
| S12.330A | Unspecified traumatic displaced spondylolisthesis of fourth cervical vertebra, initial encounter for closed fracture | | |
| S12.330B | Unspecified traumatic displaced spondylolisthesis of fourth cervical vertebra, initial encounter for open fracture | | |
| S12.331A | Unspecified traumatic nondisplaced spondylolisthesis of fourth cervical vertebra, initial encounter for closed fracture | | |
| S12.331B | Unspecified traumatic nondisplaced spondylolisthesis of fourth cervical vertebra, initial encounter for open fracture | | |
| S12.34XA | Type III traumatic spondylolisthesis of fourth cervical vertebra, initial encounter for closed fracture | | |
| S12.34XB | Type III traumatic spondylolisthesis of fourth cervical vertebra, initial encounter for open fracture | | |
| S12.350A | Other traumatic displaced spondylolisthesis of fourth cervical vertebra, initial encounter for closed fracture | | |
| S12.350B | Other traumatic displaced spondylolisthesis of fourth cervical vertebra, initial encounter for open fracture | | |
| S12.351A | Other traumatic nondisplaced spondylolisthesis of fourth cervical vertebra, initial encounter for closed fracture | | |
| S12.351B | Other traumatic nondisplaced spondylolisthesis of fourth cervical vertebra, initial encounter for open fracture | | |
| S12.390A | Other displaced fracture of fourth cervical vertebra, initial encounter for closed fracture | | |
| S12.390B | Other displaced fracture of fourth cervical vertebra, initial encounter for open fracture | | |
| S12.391A | Other nondisplaced fracture of fourth cervical vertebra, initial encounter for closed fracture | | |
| S12.391B | Other nondisplaced fracture of fourth cervical vertebra, initial encounter for open fracture | | |
| S12.400A | Unspecified displaced fracture of fifth cervical vertebra, initial encounter for closed fracture | | |
| S12.400B | Unspecified displaced fracture of fifth cervical vertebra, initial encounter for open fracture | | |
| S12.401A | Unspecified nondisplaced fracture of fifth cervical vertebra, initial encounter for closed fracture | | |
| S12.401B | Unspecified nondisplaced fracture of fifth cervical vertebra, initial encounter for open fracture | | |

© 2019 Vashi AA et al. *JAMA Network Open.*

| | | | |
|---|---|---|---|
| S12.430A | Unspecified traumatic displaced spondylolisthesis of fifth cervical vertebra, initial encounter for closed fracture | | |
| S12.430B | Unspecified traumatic displaced spondylolisthesis of fifth cervical vertebra, initial encounter for open fracture | | |
| S12.431A | Unspecified traumatic nondisplaced spondylolisthesis of fifth cervical vertebra, initial encounter for closed fracture | | |
| S12.431B | Unspecified traumatic nondisplaced spondylolisthesis of fifth cervical vertebra, initial encounter for open fracture | | |
| S12.44XA | Type III traumatic spondylolisthesis of fifth cervical vertebra, initial encounter for closed fracture | | |
| S12.44XB | Type III traumatic spondylolisthesis of fifth cervical vertebra, initial encounter for open fracture | | |
| S12.450A | Other traumatic displaced spondylolisthesis of fifth cervical vertebra, initial encounter for closed fracture | | |
| S12.450B | Other traumatic displaced spondylolisthesis of fifth cervical vertebra, initial encounter for open fracture | | |
| S12.451A | Other traumatic nondisplaced spondylolisthesis of fifth cervical vertebra, initial encounter for closed fracture | | |
| S12.451B | Other traumatic nondisplaced spondylolisthesis of fifth cervical vertebra, initial encounter for open fracture | | |
| S12.490A | Other displaced fracture of fifth cervical vertebra, initial encounter for closed fracture | | |
| S12.490B | Other displaced fracture of fifth cervical vertebra, initial encounter for open fracture | | |
| S12.491A | Other nondisplaced fracture of fifth cervical vertebra, initial encounter for closed fracture | | |
| S12.491B | Other nondisplaced fracture of fifth cervical vertebra, initial encounter for open fracture | | |
| S12.500A | Unspecified displaced fracture of sixth cervical vertebra, initial encounter for closed fracture | | |
| S12.500B | Unspecified displaced fracture of sixth cervical vertebra, initial encounter for open fracture | | |
| S12.501A | Unspecified nondisplaced fracture of sixth cervical vertebra, initial encounter for closed fracture | | |
| S12.501B | Unspecified nondisplaced fracture of sixth cervical vertebra, initial encounter for open fracture | | |
| S12.530A | Unspecified traumatic displaced spondylolisthesis of sixth cervical vertebra, initial encounter for closed fracture | | |

© 2019 Vashi AA et al. *JAMA Network Open.*

| | | | |
|---|---|---|---|
| S12.530B | Unspecified traumatic displaced spondylolisthesis of sixth cervical vertebra, initial encounter for open fracture | | |
| S12.531A | Unspecified traumatic nondisplaced spondylolisthesis of sixth cervical vertebra, initial encounter for closed fracture | | |
| S12.531B | Unspecified traumatic nondisplaced spondylolisthesis of sixth cervical vertebra, initial encounter for open fracture | | |
| S12.54XA | Type III traumatic spondylolisthesis of sixth cervical vertebra, initial encounter for closed fracture | | |
| S12.54XB | Type III traumatic spondylolisthesis of sixth cervical vertebra, initial encounter for open fracture | | |
| S12.550A | Other traumatic displaced spondylolisthesis of sixth cervical vertebra, initial encounter for closed fracture | | |
| S12.550B | Other traumatic displaced spondylolisthesis of sixth cervical vertebra, initial encounter for open fracture | | |
| S12.551A | Other traumatic nondisplaced spondylolisthesis of sixth cervical vertebra, initial encounter for closed fracture | | |
| S12.551B | Other traumatic nondisplaced spondylolisthesis of sixth cervical vertebra, initial encounter for open fracture | | |
| S12.590A | Other displaced fracture of sixth cervical vertebra, initial encounter for closed fracture | | |
| S12.590B | Other displaced fracture of sixth cervical vertebra, initial encounter for open fracture | | |
| S12.591A | Other nondisplaced fracture of sixth cervical vertebra, initial encounter for closed fracture | | |
| S12.591B | Other nondisplaced fracture of sixth cervical vertebra, initial encounter for open fracture | | |
| S12.600A | Unspecified displaced fracture of seventh cervical vertebra, initial encounter for closed fracture | | |
| S12.600B | Unspecified displaced fracture of seventh cervical vertebra, initial encounter for open fracture | | |
| S12.601A | Unspecified nondisplaced fracture of seventh cervical vertebra, initial encounter for closed fracture | | |
| S12.601B | Unspecified nondisplaced fracture of seventh cervical vertebra, initial encounter for open fracture | | |
| S12.630A | Unspecified traumatic displaced spondylolisthesis of seventh cervical vertebra, initial encounter for closed fracture | | |

© 2019 Vashi AA et al. *JAMA Network Open.*

| | | | |
|---|---|---|---|
| S12.630B | Unspecified traumatic displaced spondylolisthesis of seventh cervical vertebra, initial encounter for open fracture | | |
| S12.631A | Unspecified traumatic nondisplaced spondylolisthesis of seventh cervical vertebra, initial encounter for closed fracture | | |
| S12.631B | Unspecified traumatic nondisplaced spondylolisthesis of seventh cervical vertebra, initial encounter for open fracture | | |
| S12.64XA | Type III traumatic spondylolisthesis of seventh cervical vertebra, initial encounter for closed fracture | | |
| S12.64XB | Type III traumatic spondylolisthesis of seventh cervical vertebra, initial encounter for open fracture | | |
| S12.650A | Other traumatic displaced spondylolisthesis of seventh cervical vertebra, initial encounter for closed fracture | | |
| S12.650B | Other traumatic displaced spondylolisthesis of seventh cervical vertebra, initial encounter for open fracture | | |
| S12.651A | Other traumatic nondisplaced spondylolisthesis of seventh cervical vertebra, initial encounter for closed fracture | | |
| S12.651B | Other traumatic nondisplaced spondylolisthesis of seventh cervical vertebra, initial encounter for open fracture | | |
| S12.690A | Other displaced fracture of seventh cervical vertebra, initial encounter for closed fracture | | |
| S12.690B | Other displaced fracture of seventh cervical vertebra, initial encounter for open fracture | | |
| S12.691A | Other nondisplaced fracture of seventh cervical vertebra, initial encounter for closed fracture | | |
| S12.691B | Other nondisplaced fracture of seventh cervical vertebra, initial encounter for open fracture | | |
| S12.8XXA | Fracture of other parts of neck, initial encounter | | |
| S12.9XXA | Fracture of neck, unspecified, initial encounter | | |
| S14.0XXA | Concussion and edema of cervical spinal cord, initial encounter | | |
| S14.101A | Unspecified injury at C1 level of cervical spinal cord, initial encounter | | |
| S14.102A | Unspecified injury at C2 level of cervical spinal cord, initial encounter | | |

© 2019 Vashi AA et al. *JAMA Network Open.*

| | | | |
|---|---|---|---|
| S14.103A | Unspecified injury at C3 level of cervical spinal cord, initial encounter | | |
| S14.104A | Unspecified injury at C4 level of cervical spinal cord, initial encounter | | |
| S14.105A | Unspecified injury at C5 level of cervical spinal cord, initial encounter | | |
| S14.106A | Unspecified injury at C6 level of cervical spinal cord, initial encounter | | |
| S14.107A | Unspecified injury at C7 level of cervical spinal cord, initial encounter | | |
| S14.108A | Unspecified injury at C8 level of cervical spinal cord, initial encounter | | |
| S14.109A | Unspecified injury at unspecified level of cervical spinal cord, initial encounter | | |
| S14.111A | Complete lesion at C1 level of cervical spinal cord, initial encounter | | |
| S14.112A | Complete lesion at C2 level of cervical spinal cord, initial encounter | | |
| S14.113A | Complete lesion at C3 level of cervical spinal cord, initial encounter | | |
| S14.114A | Complete lesion at C4 level of cervical spinal cord, initial encounter | | |
| S14.115A | Complete lesion at C5 level of cervical spinal cord, initial encounter | | |
| S14.116A | Complete lesion at C6 level of cervical spinal cord, initial encounter | | |
| S14.117A | Complete lesion at C7 level of cervical spinal cord, initial encounter | | |
| S14.118A | Complete lesion at C8 level of cervical spinal cord, initial encounter | | |
| S14.119A | Complete lesion at unspecified level of cervical spinal cord, initial encounter | | |
| S14.121A | Central cord syndrome at C1 level of cervical spinal cord, initial encounter | | |
| S14.122A | Central cord syndrome at C2 level of cervical spinal cord, initial encounter | | |
| S14.123A | Central cord syndrome at C3 level of cervical spinal cord, initial encounter | | |

© 2019 Vashi AA et al. *JAMA Network Open.*

| | | | |
|---|---|---|---|
| S14.124A | Central cord syndrome at C4 level of cervical spinal cord, initial encounter | | |
| S14.125A | Central cord syndrome at C5 level of cervical spinal cord, initial encounter | | |
| S14.126A | Central cord syndrome at C6 level of cervical spinal cord, initial encounter | | |
| S14.127A | Central cord syndrome at C7 level of cervical spinal cord, initial encounter | | |
| S14.128A | Central cord syndrome at C8 level of cervical spinal cord, initial encounter | | |
| S14.129A | Central cord syndrome at unspecified level of cervical spinal cord, initial encounter | | |
| S14.131A | Anterior cord syndrome at C1 level of cervical spinal cord, initial encounter | | |
| S14.132A | Anterior cord syndrome at C2 level of cervical spinal cord, initial encounter | | |
| S14.133A | Anterior cord syndrome at C3 level of cervical spinal cord, initial encounter | | |
| S14.134A | Anterior cord syndrome at C4 level of cervical spinal cord, initial encounter | | |
| S14.135A | Anterior cord syndrome at C5 level of cervical spinal cord, initial encounter | | |
| S14.136A | Anterior cord syndrome at C6 level of cervical spinal cord, initial encounter | | |
| S14.137A | Anterior cord syndrome at C7 level of cervical spinal cord, initial encounter | | |
| S14.138A | Anterior cord syndrome at C8 level of cervical spinal cord, initial encounter | | |
| S14.139A | Anterior cord syndrome at unspecified level of cervical spinal cord, initial encounter | | |
| S14.141A | Brown-Sequard syndrome at C1 level of cervical spinal cord, initial encounter | | |
| S14.142A | Brown-Sequard syndrome at C2 level of cervical spinal cord, initial encounter | | |
| S14.143A | Brown-Sequard syndrome at C3 level of cervical spinal cord, initial encounter | | |
| S14.144A | Brown-Sequard syndrome at C4 level of cervical spinal cord, initial encounter | | |

© 2019 Vashi AA et al. *JAMA Network Open.*

| | | | |
|---|---|---|---|
| S14.145A | Brown-Sequard syndrome at C5 level of cervical spinal cord, initial encounter | | |
| S14.146A | Brown-Sequard syndrome at C6 level of cervical spinal cord, initial encounter | | |
| S14.147A | Brown-Sequard syndrome at C7 level of cervical spinal cord, initial encounter | | |
| S14.148A | Brown-Sequard syndrome at C8 level of cervical spinal cord, initial encounter | | |
| S14.149A | Brown-Sequard syndrome at unspecified level of cervical spinal cord, initial encounter | | |
| S14.151A | Other incomplete lesion at C1 level of cervical spinal cord, initial encounter | | |
| S14.152A | Other incomplete lesion at C2 level of cervical spinal cord, initial encounter | | |
| S14.153A | Other incomplete lesion at C3 level of cervical spinal cord, initial encounter | | |
| S14.154A | Other incomplete lesion at C4 level of cervical spinal cord, initial encounter | | |
| S14.155A | Other incomplete lesion at C5 level of cervical spinal cord, initial encounter | | |
| S14.156A | Other incomplete lesion at C6 level of cervical spinal cord, initial encounter | | |
| S14.157A | Other incomplete lesion at C7 level of cervical spinal cord, initial encounter | | |
| S14.158A | Other incomplete lesion at C8 level of cervical spinal cord, initial encounter | | |
| S14.159A | Other incomplete lesion at unspecified level of cervical spinal cord, initial encounter | | |
| S14.9XXA | Injury of unspecified nerves of neck, initial encounter | | |
| S15.001A | Unspecified injury of right carotid artery, initial encounter | | |
| S15.002A | Unspecified injury of left carotid artery, initial encounter | | |
| S15.009A | Unspecified injury of unspecified carotid artery, initial encounter | | |
| S15.011A | Minor laceration of right carotid artery, initial encounter | | |
| S15.012A | Minor laceration of left carotid artery, initial encounter | | |
| S15.019A | Minor laceration of unspecified carotid artery, initial encounter | | |
| S15.021A | Major laceration of right carotid artery, initial encounter | | |

© 2019 Vashi AA et al. *JAMA Network Open.*

| | | | |
|---|---|---|---|
| S15.022A | Major laceration of left carotid artery, initial encounter | | |
| S15.029A | Major laceration of unspecified carotid artery, initial encounter | | |
| S15.091A | Other specified injury of right carotid artery, initial encounter | | |
| S15.092A | Other specified injury of left carotid artery, initial encounter | | |
| S15.099A | Other specified injury of unspecified carotid artery, initial encounter | | |
| S15.101A | Unspecified injury of right vertebral artery, initial encounter | | |
| S15.102A | Unspecified injury of left vertebral artery, initial encounter | | |
| S15.109A | Unspecified injury of unspecified vertebral artery, initial encounter | | |
| S15.111A | Minor laceration of right vertebral artery, initial encounter | | |
| S15.112A | Minor laceration of left vertebral artery, initial encounter | | |
| S15.119A | Minor laceration of unspecified vertebral artery, initial encounter | | |
| S15.121A | Major laceration of right vertebral artery, initial encounter | | |
| S15.122A | Major laceration of left vertebral artery, initial encounter | | |
| S15.129A | Major laceration of unspecified vertebral artery, initial encounter | | |
| S15.191A | Other specified injury of right vertebral artery, initial encounter | | |
| S15.192A | Other specified injury of left vertebral artery, initial encounter | | |
| S15.199A | Other specified injury of unspecified vertebral artery, initial encounter | | |
| S15.201A | Unspecified injury of right external jugular vein, initial encounter | | |
| S15.202A | Unspecified injury of left external jugular vein, initial encounter | | |
| S15.209A | Unspecified injury of unspecified external jugular vein, initial encounter | | |
| S15.211A | Minor laceration of right external jugular vein, initial encounter | | |
| S15.212A | Minor laceration of left external jugular vein, initial encounter | | |

© 2019 Vashi AA et al. *JAMA Network Open.*

| | | | |
|---|---|---|---|
| S15.219A | Minor laceration of unspecified external jugular vein, initial encounter | | |
| S15.221A | Major laceration of right external jugular vein, initial encounter | | |
| S15.222A | Major laceration of left external jugular vein, initial encounter | | |
| S15.229A | Major laceration of unspecified external jugular vein, initial encounter | | |
| S15.291A | Other specified injury of right external jugular vein, initial encounter | | |
| S15.292A | Other specified injury of left external jugular vein, initial encounter | | |
| S15.299A | Other specified injury of unspecified external jugular vein, initial encounter | | |
| S15.301A | Unspecified injury of right internal jugular vein, initial encounter | | |
| S15.302A | Unspecified injury of left internal jugular vein, initial encounter | | |
| S15.309A | Unspecified injury of unspecified internal jugular vein, initial encounter | | |
| S15.311A | Minor laceration of right internal jugular vein, initial encounter | | |
| S15.312A | Minor laceration of left internal jugular vein, initial encounter | | |
| S15.319A | Minor laceration of unspecified internal jugular vein, initial encounter | | |
| S15.321A | Major laceration of right internal jugular vein, initial encounter | | |
| S15.322A | Major laceration of left internal jugular vein, initial encounter | | |
| S15.329A | Major laceration of unspecified internal jugular vein, initial encounter | | |
| S15.391A | Other specified injury of right internal jugular vein, initial encounter | | |
| S15.392A | Other specified injury of left internal jugular vein, initial encounter | | |
| S15.399A | Other specified injury of unspecified internal jugular vein, initial encounter | | |

© 2019 Vashi AA et al. *JAMA Network Open.*

| | | | |
|---|---|---|---|
| S15.8XXA | Injury of other specified blood vessels at neck level, initial encounter | | |
| S15.9XXA | Injury of unspecified blood vessel at neck level, initial encounter | | |
| S16.2XXA | Laceration of muscle, fascia and tendon at neck level, initial encounter | | |
| S16.8XXA | Other specified injury of muscle, fascia and tendon at neck level, initial encounter | | |
| S16.9XXA | Unspecified injury of muscle, fascia and tendon at neck level, initial encounter | | |
| S17.0XXA | Crushing injury of larynx and trachea, initial encounter | | |
| S17.8XXA | Crushing injury of other specified parts of neck, initial encounter | | |
| S17.9XXA | Crushing injury of neck, part unspecified, initial encounter | | |
| S19.80XA | Other specified injuries of unspecified part of neck, initial encounter | | |
| S19.81XA | Other specified injuries of larynx, initial encounter | | |
| S19.82XA | Other specified injuries of cervical trachea, initial encounter | | |
| S19.83XA | Other specified injuries of vocal cord, initial encounter | | |
| S19.84XA | Other specified injuries of thyroid gland, initial encounter | | |
| S19.85XA | Other specified injuries of pharynx and cervical esophagus, initial encounter | | |
| S19.89XA | Other specified injuries of other specified part of neck, initial encounter | | |
| S19.9XXA | Unspecified injury of neck, initial encounter | | |

© 2019 Vashi AA et al. *JAMA Network Open.*

## Included and Excluded ICD-10-CM Diagnosis Subcodes for Other Cardiac Arrhythmia Condition Group

| Included ICD-10-CM diagnosis subcodes (n=7) | | Excluded ICD-10-CM diagnosis subcodes (n=11) | |
|---|---|---|---|
| **Subcode** | **Subcode description** | **Subcode** | **Subcode description** |
| I44.1 | Atrioventricular block, second degree | I44.0 | Atrioventricular block, first degree |
| I44.2 | Atrioventricular block, complete | I44.4 | Left anterior fascicular block |
| I44.30 | Unspecified atrioventricular block | I44.5 | Left posterior fascicular block |
| I44.39 | Other atrioventricular block | I44.60 | Unspecified fascicular block |
| I49.5 | Sick sinus syndrome | I44.69 | Other fascicular block |
| I49.8 | Other specified cardiac arrhythmias | I44.7 | Left bundle-branch block, unspecified |
| I49.9 | Cardiac arrhythmia, unspecified | I49.1 | Atrial premature depolarization |
| | | I49.2 | Junctional premature depolarization |
| | | I49.3 | Ventricular premature depolarization |
| | | I49.40 | Unspecified premature depolarization |
| | | I49.49 | Other premature depolarization |

## Included and Excluded ICD-10-CM Diagnosis Subcodes for Other Diseases of Intestine Condition Group

| Included ICD-10-CM diagnosis subcodes (n=3) | | Excluded ICD-10-CM diagnosis subcodes (n=6) | |
|---|---|---|---|
| **Subcode** | **Subcode description** | **Subcode** | **Subcode description** |
| K63.0 | Abscess of intestine | K63.3 | Ulcer of intestine |
| K63.1 | Perforation of intestine (nontraumatic) | K63.4 | Enteroptosis |
| K63.2 | Fistula of intestine | K63.5 | Polyp of colon |
| | | K63.81 | Dieulafoy lesion of intestine |
| | | K63.89 | Other specified diseases of intestine |
| | | K63.9 | Disease of intestine, unspecified |

© 2019 Vashi AA et al. *JAMA Network Open.*

## Included and Excluded ICD-10-CM Diagnosis Subcodes for Other Tachyarrhythmias Condition Group

| Included ICD-10-CM diagnosis subcodes (n=8) | | Excluded ICD-10-CM diagnosis subcodes (n=2) | |
|---|---|---|---|
| **Subcode** | **Subcode description** | **Subcode** | **Subcode description** |
| I47.0 | Re-entry ventricular arrhythmia | I48.1 | Persistent atrial fibrillation |
| I47.1 | Supraventricular tachycardia | I48.2 | Chronic atrial fibrillation |
| I47.9 | Paroxysmal tachycardia, unspecified | | |
| I48.0 | Paroxysmal atrial fibrillation | | |
| I48.3 | Typical atrial flutter | | |
| I48.4 | Atypical atrial flutter | | |
| I48.91 | Unspecified atrial fibrillation | | |
| I48.92 | Unspecified atrial flutter | | |

© 2019 Vashi AA et al. *JAMA Network Open.*

## Included and Excluded ICD-10-CM Diagnosis Subcodes for Overdose/Poisonings Condition Group

| Included ICD-10-CM diagnosis subcodes (n=572) | | Excluded ICD-10-CM diagnosis subcodes (n=68) | |
|---|---|---|---|
| **Subcode** | **Subcode description** | **Subcode** | **Subcode description** |
| T39.011A | Poisoning by aspirin, accidental (unintentional), initial encounter | T39.016A | Underdosing of aspirin, initial encounter |
| T39.012A | Poisoning by aspirin, intentional self-harm, initial encounter | T39.096A | Underdosing of salicylates, initial encounter |
| T39.013A | Poisoning by aspirin, assault, initial encounter | T39.1X6A | Underdosing of 4-Aminophenol derivatives, initial encounter |
| T39.014A | Poisoning by aspirin, undetermined, initial encounter | T39.2X6A | Underdosing of pyrazolone derivatives, initial encounter |
| T39.015A | Adverse effect of aspirin, initial encounter | T39.316A | Underdosing of propionic acid derivatives, initial encounter |
| T39.091A | Poisoning by salicylates, accidental (unintentional), initial encounter | T39.396A | Underdosing of other nonsteroidal anti-inflammatory drugs [NSAID], initial encounter |
| T39.092A | Poisoning by salicylates, intentional self-harm, initial encounter | T39.4X6A | Underdosing of antirheumatics, not elsewhere classified, initial encounter |
| T39.093A | Poisoning by salicylates, assault, initial encounter | T39.8X6A | Underdosing of other nonopioid analgesics and antipyretics, not elsewhere classified, initial encounter |
| T39.094A | Poisoning by salicylates, undetermined, initial encounter | T39.96XA | Underdosing of unspecified nonopioid analgesic, antipyretic and antirheumatic, initial encounter |
| T39.095A | Adverse effect of salicylates, initial encounter | T40.0X6A | Underdosing of opium, initial encounter |
| T39.1X1A | Poisoning by 4-Aminophenol derivatives, accidental (unintentional), initial encounter | T40.2X6A | Underdosing of other opioids, initial encounter |
| T39.1X2A | Poisoning by 4-Aminophenol derivatives, intentional self-harm, initial encounter | T40.3X6A | Underdosing of methadone, initial encounter |
| T39.1X3A | Poisoning by 4-Aminophenol derivatives, assault, initial encounter | T40.4X6A | Underdosing of other synthetic narcotics, initial encounter |
| T39.1X4A | Poisoning by 4-Aminophenol derivatives, undetermined, initial encounter | T40.5X6A | Underdosing of cocaine, initial encounter |
| T39.1X5A | Adverse effect of 4-Aminophenol derivatives, initial encounter | T40.606A | Underdosing of unspecified narcotics, initial encounter |
| T39.2X1A | Poisoning by pyrazolone derivatives, accidental (unintentional), initial encounter | T40.696A | Underdosing of other narcotics, initial encounter |
| T39.2X2A | Poisoning by pyrazolone derivatives, intentional self-harm, initial encounter | T40.7X6A | Underdosing of cannabis (derivatives), initial encounter |
| T39.2X3A | Poisoning by pyrazolone derivatives, assault, initial encounter | T40.906A | Underdosing of unspecified psychodysleptics, initial encounter |

© 2019 Vashi AA et al. *JAMA Network Open.*

| | | | |
|---|---|---|---|
| T39.2X4A | Poisoning by pyrazolone derivatives, undetermined, initial encounter | T40.996A | Underdosing of other psychodysleptics, initial encounter |
| T39.2X5A | Adverse effect of pyrazolone derivatives, initial encounter | T42.0X6A | Underdosing of hydantoin derivatives, initial encounter |
| T39.311A | Poisoning by propionic acid derivatives, accidental (unintentional), initial encounter | T42.1X6A | Underdosing of iminostilbenes, initial encounter |
| T39.312A | Poisoning by propionic acid derivatives, intentional self-harm, initial encounter | T42.2X6A | Underdosing of succinimides and oxazolidinediones, initial encounter |
| T39.313A | Poisoning by propionic acid derivatives, assault, initial encounter | T42.3X6A | Underdosing of barbiturates, initial encounter |
| T39.314A | Poisoning by propionic acid derivatives, undetermined, initial encounter | T42.4X6A | Underdosing of benzodiazepines, initial encounter |
| T39.315A | Adverse effect of propionic acid derivatives, initial encounter | T42.5X6A | Underdosing of mixed antiepileptics, initial encounter |
| T39.391A | Poisoning by other nonsteroidal anti-inflammatory drugs [NSAID], accidental (unintentional), initial encounter | T42.6X6A | Underdosing of other antiepileptic and sedative-hypnotic drugs, initial encounter |
| T39.392A | Poisoning by other nonsteroidal anti-inflammatory drugs [NSAID], intentional self-harm, initial encounter | T42.76XA | Underdosing of unspecified antiepileptic and sedative-hypnotic drugs, initial encounter |
| T39.393A | Poisoning by other nonsteroidal anti-inflammatory drugs [NSAID], assault, initial encounter | T42.8X6A | Underdosing of antiparkinsonism drugs and other central muscle-tone depressants, initial encounter |
| T39.394A | Poisoning by other nonsteroidal anti-inflammatory drugs [NSAID], undetermined, initial encounter | T43.016A | Underdosing of tricyclic antidepressants, initial encounter |
| T39.395A | Adverse effect of other nonsteroidal anti-inflammatory drugs [NSAID], initial encounter | T43.026A | Underdosing of tetracyclic antidepressants, initial encounter |
| T39.4X1A | Poisoning by antirheumatics, not elsewhere classified, accidental (unintentional), initial encounter | T43.1X6A | Underdosing of monoamine-oxidase-inhibitor antidepressants, initial encounter |
| T39.4X2A | Poisoning by antirheumatics, not elsewhere classified, intentional self-harm, initial encounter | T43.206A | Underdosing of unspecified antidepressants, initial encounter |
| T39.4X3A | Poisoning by antirheumatics, not elsewhere classified, assault, initial encounter | T43.216A | Underdosing of selective serotonin and norepinephrine reuptake inhibitors, initial encounter |
| T39.4X4A | Poisoning by antirheumatics, not elsewhere classified, undetermined, initial encounter | T43.226A | Underdosing of selective serotonin reuptake inhibitors, initial encounter |
| T39.4X5A | Adverse effect of antirheumatics, not elsewhere classified, initial encounter | T43.296A | Underdosing of other antidepressants, initial encounter |
| T39.8X1A | Poisoning by other nonopioid analgesics and antipyretics, not elsewhere classified, accidental (unintentional), initial encounter | T43.3X6A | Underdosing of phenothiazine antipsychotics and neuroleptics, initial encounter |
| T39.8X2A | Poisoning by other nonopioid analgesics and antipyretics, not elsewhere classified, intentional self-harm, initial encounter | T43.4X6A | Underdosing of butyrophenone and thiothixene neuroleptics, initial encounter |

© 2019 Vashi AA et al. *JAMA Network Open.*

| | | | |
|---|---|---|---|
| T39.8X3A | Poisoning by other nonopioid analgesics and antipyretics, not elsewhere classified, assault, initial encounter | T43.506A | Underdosing of unspecified antipsychotics and neuroleptics, initial encounter |
| T39.8X4A | Poisoning by other nonopioid analgesics and antipyretics, not elsewhere classified, undetermined, initial encounter | T43.596A | Underdosing of other antipsychotics and neuroleptics, initial encounter |
| T39.8X5A | Adverse effect of other nonopioid analgesics and antipyretics, not elsewhere classified, initial encounter | T43.606A | Underdosing of unspecified psychostimulants, initial encounter |
| T39.91XA | Poisoning by unspecified nonopioid analgesic, antipyretic and antirheumatic, accidental (unintentional), initial encounter | T43.616A | Underdosing of caffeine, initial encounter |
| T39.92XA | Poisoning by unspecified nonopioid analgesic, antipyretic and antirheumatic, intentional self-harm, initial encounter | T43.626A | Underdosing of amphetamines, initial encounter |
| T39.93XA | Poisoning by unspecified nonopioid analgesic, antipyretic and antirheumatic, assault, initial encounter | T43.636A | Underdosing of methylphenidate, initial encounter |
| T39.94XA | Poisoning by unspecified nonopioid analgesic, antipyretic and antirheumatic, undetermined, initial encounter | T43.696A | Underdosing of other psychostimulants, initial encounter |
| T39.95XA | Adverse effect of unspecified nonopioid analgesic, antipyretic and antirheumatic, initial encounter | T43.8X6A | Underdosing of other psychotropic drugs, initial encounter |
| T40.0X1A | Poisoning by opium, accidental (unintentional), initial encounter | T43.96XA | Underdosing of unspecified psychotropic drug, initial encounter |
| T40.0X2A | Poisoning by opium, intentional self-harm, initial encounter | T44.0X6A | Underdosing of anticholinesterase agents, initial encounter |
| T40.0X3A | Poisoning by opium, assault, initial encounter | T44.1X6A | Underdosing of other parasympathomimetics, initial encounter |
| T40.0X4A | Poisoning by opium, undetermined, initial encounter | T44.2X6A | Underdosing of ganglionic blocking drugs, initial encounter |
| T40.0X5A | Adverse effect of opium, initial encounter | T44.3X6A | Underdosing of other parasympatholytics [anticholinergics and antimuscarinics] and spasmolytics, initial encounter |
| T40.1X1A | Poisoning by heroin, accidental (unintentional), initial encounter | T44.4X6A | Underdosing of predominantly alpha-adrenoreceptor agonists, initial encounter |
| T40.1X2A | Poisoning by heroin, intentional self-harm, initial encounter | T44.5X6A | Underdosing of predominantly beta-adrenoreceptor agonists, initial encounter |
| T40.1X3A | Poisoning by heroin, assault, initial encounter | T44.6X6A | Underdosing of alpha-adrenoreceptor antagonists, initial encounter |
| T40.1X4A | Poisoning by heroin, undetermined, initial encounter | T44.7X6A | Underdosing of beta-adrenoreceptor antagonists, initial encounter |
| T40.2X1A | Poisoning by other opioids, accidental (unintentional), initial encounter | T44.8X6A | Underdosing of centrally-acting and adrenergic-neuron-blocking agents, initial encounter |

© 2019 Vashi AA et al. *JAMA Network Open.*

| | | | |
|---|---|---|---|
| T40.2X2A | Poisoning by other opioids, intentional self-harm, initial encounter | T44.906A | Underdosing of unspecified drugs primarily affecting the autonomic nervous system, initial encounter |
| T40.2X3A | Poisoning by other opioids, assault, initial encounter | T44.996A | Underdosing of other drug primarily affecting the autonomic nervous system, initial encounter |
| T40.2X4A | Poisoning by other opioids, undetermined, initial encounter | T46.0X6A | Underdosing of cardiac-stimulant glycosides and drugs of similar action, initial encounter |
| T40.2X5A | Adverse effect of other opioids, initial encounter | T46.1X6A | Underdosing of calcium-channel blockers, initial encounter |
| T40.3X1A | Poisoning by methadone, accidental (unintentional), initial encounter | T46.2X6A | Underdosing of other antidysrhythmic drugs, initial encounter |
| T40.3X2A | Poisoning by methadone, intentional self-harm, initial encounter | T46.3X6A | Underdosing of coronary vasodilators, initial encounter |
| T40.3X3A | Poisoning by methadone, assault, initial encounter | T46.4X6A | Underdosing of angiotensin-converting-enzyme inhibitors, initial encounter |
| T40.3X4A | Poisoning by methadone, undetermined, initial encounter | T46.5X6A | Underdosing of other antihypertensive drugs, initial encounter |
| T40.3X5A | Adverse effect of methadone, initial encounter | T46.6X6A | Underdosing of antihyperlipidemic and antiarteriosclerotic drugs, initial encounter |
| T40.4X1A | Poisoning by other synthetic narcotics, accidental (unintentional), initial encounter | T46.7X6A | Underdosing of peripheral vasodilators, initial encounter |
| T40.4X2A | Poisoning by other synthetic narcotics, intentional self-harm, initial encounter | T46.8X6A | Underdosing of antivaricose drugs, including sclerosing agents, initial encounter |
| T40.4X3A | Poisoning by other synthetic narcotics, assault, initial encounter | T46.906A | Underdosing of unspecified agents primarily affecting the cardiovascular system, initial encounter |
| T40.4X4A | Poisoning by other synthetic narcotics, undetermined, initial encounter | T46.996A | Underdosing of other agents primarily affecting the cardiovascular system, initial encounter |
| T40.4X5A | Adverse effect of other synthetic narcotics, initial encounter | | |
| T40.5X1A | Poisoning by cocaine, accidental (unintentional), initial encounter | | |
| T40.5X2A | Poisoning by cocaine, intentional self-harm, initial encounter | | |
| T40.5X3A | Poisoning by cocaine, assault, initial encounter | | |
| T40.5X4A | Poisoning by cocaine, undetermined, initial encounter | | |
| T40.5X5A | Adverse effect of cocaine, initial encounter | | |
| T40.601A | Poisoning by unspecified narcotics, accidental (unintentional), initial encounter | | |
| T40.602A | Poisoning by unspecified narcotics, intentional self-harm, initial encounter | | |

© 2019 Vashi AA et al. *JAMA Network Open.*

| | | | |
|---|---|---|---|
| T40.603A | Poisoning by unspecified narcotics, assault, initial encounter | | |
| T40.604A | Poisoning by unspecified narcotics, undetermined, initial encounter | | |
| T40.605A | Adverse effect of unspecified narcotics, initial encounter | | |
| T40.691A | Poisoning by other narcotics, accidental (unintentional), initial encounter | | |
| T40.692A | Poisoning by other narcotics, intentional self-harm, initial encounter | | |
| T40.693A | Poisoning by other narcotics, assault, initial encounter | | |
| T40.694A | Poisoning by other narcotics, undetermined, initial encounter | | |
| T40.695A | Adverse effect of other narcotics, initial encounter | | |
| T40.7X1A | Poisoning by cannabis (derivatives), accidental (unintentional), initial encounter | | |
| T40.7X2A | Poisoning by cannabis (derivatives), intentional self-harm, initial encounter | | |
| T40.7X3A | Poisoning by cannabis (derivatives), assault, initial encounter | | |
| T40.7X4A | Poisoning by cannabis (derivatives), undetermined, initial encounter | | |
| T40.7X5A | Adverse effect of cannabis (derivatives), initial encounter | | |
| T40.8X1A | Poisoning by lysergide [LSD], accidental (unintentional), initial encounter | | |
| T40.8X2A | Poisoning by lysergide [LSD], intentional self-harm, initial encounter | | |
| T40.8X3A | Poisoning by lysergide [LSD], assault, initial encounter | | |
| T40.8X4A | Poisoning by lysergide [LSD], undetermined, initial encounter | | |
| T40.901A | Poisoning by unspecified psychodysleptics [hallucinogens], accidental (unintentional), initial encounter | | |
| T40.902A | Poisoning by unspecified psychodysleptics [hallucinogens], intentional self-harm, initial encounter | | |
| T40.903A | Poisoning by unspecified psychodysleptics [hallucinogens], assault, initial encounter | | |
| T40.904A | Poisoning by unspecified psychodysleptics [hallucinogens], undetermined, initial encounter | | |

© 2019 Vashi AA et al. *JAMA Network Open.*

| | | | |
|---|---|---|---|
| T40.905A | Adverse effect of unspecified psychodysleptics [hallucinogens], initial encounter | | |
| T40.991A | Poisoning by other psychodysleptics [hallucinogens], accidental (unintentional), initial encounter | | |
| T40.992A | Poisoning by other psychodysleptics [hallucinogens], intentional self-harm, initial encounter | | |
| T40.993A | Poisoning by other psychodysleptics [hallucinogens], assault, initial encounter | | |
| T40.994A | Poisoning by other psychodysleptics [hallucinogens], undetermined, initial encounter | | |
| T40.995A | Adverse effect of other psychodysleptics [hallucinogens], initial encounter | | |
| T42.0X1A | Poisoning by hydantoin derivatives, accidental (unintentional), initial encounter | | |
| T42.0X2A | Poisoning by hydantoin derivatives, intentional self-harm, initial encounter | | |
| T42.0X3A | Poisoning by hydantoin derivatives, assault, initial encounter | | |
| T42.0X4A | Poisoning by hydantoin derivatives, undetermined, initial encounter | | |
| T42.0X5A | Adverse effect of hydantoin derivatives, initial encounter | | |
| T42.1X1A | Poisoning by iminostilbenes, accidental (unintentional), initial encounter | | |
| T42.1X2A | Poisoning by iminostilbenes, intentional self-harm, initial encounter | | |
| T42.1X3A | Poisoning by iminostilbenes, assault, initial encounter | | |
| T42.1X4A | Poisoning by iminostilbenes, undetermined, initial encounter | | |
| T42.1X5A | Adverse effect of iminostilbenes, initial encounter | | |
| T42.2X1A | Poisoning by succinimides and oxazolidinediones, accidental (unintentional), initial encounter | | |
| T42.2X2A | Poisoning by succinimides and oxazolidinediones, intentional self-harm, initial encounter | | |
| T42.2X3A | Poisoning by succinimides and oxazolidinediones, assault, initial encounter | | |
| T42.2X4A | Poisoning by succinimides and oxazolidinediones, undetermined, initial encounter | | |
| T42.2X5A | Adverse effect of succinimides and oxazolidinediones, initial encounter | | |

© 2019 Vashi AA et al. *JAMA Network Open.*

| | | | |
|---|---|---|---|
| T42.3X1A | Poisoning by barbiturates, accidental (unintentional), initial encounter | | |
| T42.3X2A | Poisoning by barbiturates, intentional self-harm, initial encounter | | |
| T42.3X3A | Poisoning by barbiturates, assault, initial encounter | | |
| T42.3X4A | Poisoning by barbiturates, undetermined, initial encounter | | |
| T42.3X5A | Adverse effect of barbiturates, initial encounter | | |
| T42.4X1A | Poisoning by benzodiazepines, accidental (unintentional), initial encounter | | |
| T42.4X2A | Poisoning by benzodiazepines, intentional self-harm, initial encounter | | |
| T42.4X3A | Poisoning by benzodiazepines, assault, initial encounter | | |
| T42.4X4A | Poisoning by benzodiazepines, undetermined, initial encounter | | |
| T42.4X5A | Adverse effect of benzodiazepines, initial encounter | | |
| T42.5X1A | Poisoning by mixed antiepileptics, accidental (unintentional), initial encounter | | |
| T42.5X2A | Poisoning by mixed antiepileptics, intentional self-harm, initial encounter | | |
| T42.5X3A | Poisoning by mixed antiepileptics, assault, initial encounter | | |
| T42.5X4A | Poisoning by mixed antiepileptics, undetermined, initial encounter | | |
| T42.5X5A | Adverse effect of mixed antiepileptics, initial encounter | | |
| T42.6X1A | Poisoning by other antiepileptic and sedative-hypnotic drugs, accidental (unintentional), initial encounter | | |
| T42.6X2A | Poisoning by other antiepileptic and sedative-hypnotic drugs, intentional self-harm, initial encounter | | |
| T42.6X3A | Poisoning by other antiepileptic and sedative-hypnotic drugs, assault, initial encounter | | |
| T42.6X4A | Poisoning by other antiepileptic and sedative-hypnotic drugs, undetermined, initial encounter | | |
| T42.6X5A | Adverse effect of other antiepileptic and sedative-hypnotic drugs, initial encounter | | |
| T42.71XA | Poisoning by unspecified antiepileptic and sedative-hypnotic drugs, accidental (unintentional), initial encounter | | |

© 2019 Vashi AA et al. *JAMA Network Open.*

| | | | |
|---|---|---|---|
| T42.72XA | Poisoning by unspecified antiepileptic and sedative-hypnotic drugs, intentional self-harm, initial encounter | | |
| T42.73XA | Poisoning by unspecified antiepileptic and sedative-hypnotic drugs, assault, initial encounter | | |
| T42.74XA | Poisoning by unspecified antiepileptic and sedative-hypnotic drugs, undetermined, initial encounter | | |
| T42.75XA | Adverse effect of unspecified antiepileptic and sedative-hypnotic drugs, initial encounter | | |
| T42.8X1A | Poisoning by antiparkinsonism drugs and other central muscle-tone depressants, accidental (unintentional), initial encounter | | |
| T42.8X2A | Poisoning by antiparkinsonism drugs and other central muscle-tone depressants, intentional self-harm, initial encounter | | |
| T42.8X3A | Poisoning by antiparkinsonism drugs and other central muscle-tone depressants, assault, initial encounter | | |
| T42.8X4A | Poisoning by antiparkinsonism drugs and other central muscle-tone depressants, undetermined, initial encounter | | |
| T42.8X5A | Adverse effect of antiparkinsonism drugs and other central muscle-tone depressants, initial encounter | | |
| T43.011A | Poisoning by tricyclic antidepressants, accidental (unintentional), initial encounter | | |
| T43.012A | Poisoning by tricyclic antidepressants, intentional self-harm, initial encounter | | |
| T43.013A | Poisoning by tricyclic antidepressants, assault, initial encounter | | |
| T43.014A | Poisoning by tricyclic antidepressants, undetermined, initial encounter | | |
| T43.015A | Adverse effect of tricyclic antidepressants, initial encounter | | |
| T43.021A | Poisoning by tetracyclic antidepressants, accidental (unintentional), initial encounter | | |
| T43.022A | Poisoning by tetracyclic antidepressants, intentional self-harm, initial encounter | | |
| T43.023A | Poisoning by tetracyclic antidepressants, assault, initial encounter | | |
| T43.024A | Poisoning by tetracyclic antidepressants, undetermined, initial encounter | | |

© 2019 Vashi AA et al. *JAMA Network Open.*

| | | | |
|---|---|---|---|
| T43.025A | Adverse effect of tetracyclic antidepressants, initial encounter | | |
| T43.1X1A | Poisoning by monoamine-oxidase-inhibitor antidepressants, accidental (unintentional), initial encounter | | |
| T43.1X2A | Poisoning by monoamine-oxidase-inhibitor antidepressants, intentional self-harm, initial encounter | | |
| T43.1X3A | Poisoning by monoamine-oxidase-inhibitor antidepressants, assault, initial encounter | | |
| T43.1X4A | Poisoning by monoamine-oxidase-inhibitor antidepressants, undetermined, initial encounter | | |
| T43.1X5A | Adverse effect of monoamine-oxidase-inhibitor antidepressants, initial encounter | | |
| T43.201A | Poisoning by unspecified antidepressants, accidental (unintentional), initial encounter | | |
| T43.202A | Poisoning by unspecified antidepressants, intentional self-harm, initial encounter | | |
| T43.203A | Poisoning by unspecified antidepressants, assault, initial encounter | | |
| T43.204A | Poisoning by unspecified antidepressants, undetermined, initial encounter | | |
| T43.205A | Adverse effect of unspecified antidepressants, initial encounter | | |
| T43.211A | Poisoning by selective serotonin and norepinephrine reuptake inhibitors, accidental (unintentional), initial encounter | | |
| T43.212A | Poisoning by selective serotonin and norepinephrine reuptake inhibitors, intentional self-harm, initial encounter | | |
| T43.213A | Poisoning by selective serotonin and norepinephrine reuptake inhibitors, assault, initial encounter | | |
| T43.214A | Poisoning by selective serotonin and norepinephrine reuptake inhibitors, undetermined, initial encounter | | |
| T43.215A | Adverse effect of selective serotonin and norepinephrine reuptake inhibitors, initial encounter | | |
| T43.221A | Poisoning by selective serotonin reuptake inhibitors, accidental (unintentional), initial encounter | | |
| T43.222A | Poisoning by selective serotonin reuptake inhibitors, intentional self-harm, initial encounter | | |

© 2019 Vashi AA et al. *JAMA Network Open.*

| | | | |
|---|---|---|---|
| T43.223A | Poisoning by selective serotonin reuptake inhibitors, assault, initial encounter | | |
| T43.224A | Poisoning by selective serotonin reuptake inhibitors, undetermined, initial encounter | | |
| T43.225A | Adverse effect of selective serotonin reuptake inhibitors, initial encounter | | |
| T43.291A | Poisoning by other antidepressants, accidental (unintentional), initial encounter | | |
| T43.292A | Poisoning by other antidepressants, intentional self-harm, initial encounter | | |
| T43.293A | Poisoning by other antidepressants, assault, initial encounter | | |
| T43.294A | Poisoning by other antidepressants, undetermined, initial encounter | | |
| T43.295A | Adverse effect of other antidepressants, initial encounter | | |
| T43.3X1A | Poisoning by phenothiazine antipsychotics and neuroleptics, accidental (unintentional), initial encounter | | |
| T43.3X2A | Poisoning by phenothiazine antipsychotics and neuroleptics, intentional self-harm, initial encounter | | |
| T43.3X3A | Poisoning by phenothiazine antipsychotics and neuroleptics, assault, initial encounter | | |
| T43.3X4A | Poisoning by phenothiazine antipsychotics and neuroleptics, undetermined, initial encounter | | |
| T43.3X5A | Adverse effect of phenothiazine antipsychotics and neuroleptics, initial encounter | | |
| T43.4X1A | Poisoning by butyrophenone and thiothixene neuroleptics, accidental (unintentional), initial encounter | | |
| T43.4X2A | Poisoning by butyrophenone and thiothixene neuroleptics, intentional self-harm, initial encounter | | |
| T43.4X3A | Poisoning by butyrophenone and thiothixene neuroleptics, assault, initial encounter | | |
| T43.4X4A | Poisoning by butyrophenone and thiothixene neuroleptics, undetermined, initial encounter | | |
| T43.4X5A | Adverse effect of butyrophenone and thiothixene neuroleptics, initial encounter | | |
| T43.501A | Poisoning by unspecified antipsychotics and neuroleptics, accidental (unintentional), initial encounter | | |
| T43.502A | Poisoning by unspecified antipsychotics and neuroleptics, intentional self-harm, initial encounter | | |

© 2019 Vashi AA et al. *JAMA Network Open.*

| | | | |
|---|---|---|---|
| T43.503A | Poisoning by unspecified antipsychotics and neuroleptics, assault, initial encounter | | |
| T43.504A | Poisoning by unspecified antipsychotics and neuroleptics, undetermined, initial encounter | | |
| T43.505A | Adverse effect of unspecified antipsychotics and neuroleptics, initial encounter | | |
| T43.591A | Poisoning by other antipsychotics and neuroleptics, accidental (unintentional), initial encounter | | |
| T43.592A | Poisoning by other antipsychotics and neuroleptics, intentional self-harm, initial encounter | | |
| T43.593A | Poisoning by other antipsychotics and neuroleptics, assault, initial encounter | | |
| T43.594A | Poisoning by other antipsychotics and neuroleptics, undetermined, initial encounter | | |
| T43.595A | Adverse effect of other antipsychotics and neuroleptics, initial encounter | | |
| T43.601A | Poisoning by unspecified psychostimulants, accidental (unintentional), initial encounter | | |
| T43.602A | Poisoning by unspecified psychostimulants, intentional self-harm, initial encounter | | |
| T43.603A | Poisoning by unspecified psychostimulants, assault, initial encounter | | |
| T43.604A | Poisoning by unspecified psychostimulants, undetermined, initial encounter | | |
| T43.605A | Adverse effect of unspecified psychostimulants, initial encounter | | |
| T43.611A | Poisoning by caffeine, accidental (unintentional), initial encounter | | |
| T43.612A | Poisoning by caffeine, intentional self-harm, initial encounter | | |
| T43.613A | Poisoning by caffeine, assault, initial encounter | | |
| T43.614A | Poisoning by caffeine, undetermined, initial encounter | | |
| T43.615A | Adverse effect of caffeine, initial encounter | | |
| T43.621A | Poisoning by amphetamines, accidental (unintentional), initial encounter | | |
| T43.622A | Poisoning by amphetamines, intentional self-harm, initial encounter | | |
| T43.623A | Poisoning by amphetamines, assault, initial encounter | | |

© 2019 Vashi AA et al. *JAMA Network Open.*

| | | | |
|---|---|---|---|
| T43.624A | Poisoning by amphetamines, undetermined, initial encounter | | |
| T43.625A | Adverse effect of amphetamines, initial encounter | | |
| T43.631A | Poisoning by methylphenidate, accidental (unintentional), initial encounter | | |
| T43.632A | Poisoning by methylphenidate, intentional self-harm, initial encounter | | |
| T43.633A | Poisoning by methylphenidate, assault, initial encounter | | |
| T43.634A | Poisoning by methylphenidate, undetermined, initial encounter | | |
| T43.635A | Adverse effect of methylphenidate, initial encounter | | |
| T43.691A | Poisoning by other psychostimulants, accidental (unintentional), initial encounter | | |
| T43.692A | Poisoning by other psychostimulants, intentional self-harm, initial encounter | | |
| T43.693A | Poisoning by other psychostimulants, assault, initial encounter | | |
| T43.694A | Poisoning by other psychostimulants, undetermined, initial encounter | | |
| T43.695A | Adverse effect of other psychostimulants, initial encounter | | |
| T43.8X1A | Poisoning by other psychotropic drugs, accidental (unintentional), initial encounter | | |
| T43.8X2A | Poisoning by other psychotropic drugs, intentional self-harm, initial encounter | | |
| T43.8X3A | Poisoning by other psychotropic drugs, assault, initial encounter | | |
| T43.8X4A | Poisoning by other psychotropic drugs, undetermined, initial encounter | | |
| T43.8X5A | Adverse effect of other psychotropic drugs, initial encounter | | |
| T43.91XA | Poisoning by unspecified psychotropic drug, accidental (unintentional), initial encounter | | |
| T43.92XA | Poisoning by unspecified psychotropic drug, intentional self-harm, initial encounter | | |
| T43.93XA | Poisoning by unspecified psychotropic drug, assault, initial encounter | | |
| T43.94XA | Poisoning by unspecified psychotropic drug, undetermined, initial encounter | | |

© 2019 Vashi AA et al. *JAMA Network Open.*

| | | | |
|---|---|---|---|
| T43.95XA | Adverse effect of unspecified psychotropic drug, initial encounter | | |
| T44.0X1A | Poisoning by anticholinesterase agents, accidental (unintentional), initial encounter | | |
| T44.0X2A | Poisoning by anticholinesterase agents, intentional self-harm, initial encounter | | |
| T44.0X3A | Poisoning by anticholinesterase agents, assault, initial encounter | | |
| T44.0X4A | Poisoning by anticholinesterase agents, undetermined, initial encounter | | |
| T44.0X5A | Adverse effect of anticholinesterase agents, initial encounter | | |
| T44.1X1A | Poisoning by other parasympathomimetics [cholinergics], accidental (unintentional), initial encounter | | |
| T44.1X2A | Poisoning by other parasympathomimetics [cholinergics], intentional self-harm, initial encounter | | |
| T44.1X3A | Poisoning by other parasympathomimetics [cholinergics], assault, initial encounter | | |
| T44.1X4A | Poisoning by other parasympathomimetics [cholinergics], undetermined, initial encounter | | |
| T44.1X5A | Adverse effect of other parasympathomimetics [cholinergics], initial encounter | | |
| T44.2X1A | Poisoning by ganglionic blocking drugs, accidental (unintentional), initial encounter | | |
| T44.2X2A | Poisoning by ganglionic blocking drugs, intentional self-harm, initial encounter | | |
| T44.2X3A | Poisoning by ganglionic blocking drugs, assault, initial encounter | | |
| T44.2X4A | Poisoning by ganglionic blocking drugs, undetermined, initial encounter | | |
| T44.2X5A | Adverse effect of ganglionic blocking drugs, initial encounter | | |
| T44.3X1A | Poisoning by other parasympatholytics [anticholinergics and antimuscarinics] and spasmolytics, accidental (unintentional), initial encounter | | |
| T44.3X2A | Poisoning by other parasympatholytics [anticholinergics and antimuscarinics] and spasmolytics, intentional self-harm, initial encounter | | |

© 2019 Vashi AA et al. *JAMA Network Open.*

| | | | |
|---|---|---|---|
| T44.3X3A | Poisoning by other parasympatholytics [anticholinergics and antimuscarinics] and spasmolytics, assault, initial encounter | | |
| T44.3X4A | Poisoning by other parasympatholytics [anticholinergics and antimuscarinics] and spasmolytics, undetermined, initial encounter | | |
| T44.3X5A | Adverse effect of other parasympatholytics [anticholinergics and antimuscarinics] and spasmolytics, initial encounter | | |
| T44.4X1A | Poisoning by predominantly alpha-adrenoreceptor agonists, accidental (unintentional), initial encounter | | |
| T44.4X2A | Poisoning by predominantly alpha-adrenoreceptor agonists, intentional self-harm, initial encounter | | |
| T44.4X3A | Poisoning by predominantly alpha-adrenoreceptor agonists, assault, initial encounter | | |
| T44.4X4A | Poisoning by predominantly alpha-adrenoreceptor agonists, undetermined, initial encounter | | |
| T44.4X5A | Adverse effect of predominantly alpha-adrenoreceptor agonists, initial encounter | | |
| T44.5X1A | Poisoning by predominantly beta-adrenoreceptor agonists, accidental (unintentional), initial encounter | | |
| T44.5X2A | Poisoning by predominantly beta-adrenoreceptor agonists, intentional self-harm, initial encounter | | |
| T44.5X3A | Poisoning by predominantly beta-adrenoreceptor agonists, assault, initial encounter | | |
| T44.5X4A | Poisoning by predominantly beta-adrenoreceptor agonists, undetermined, initial encounter | | |
| T44.5X5A | Adverse effect of predominantly beta-adrenoreceptor agonists, initial encounter | | |
| T44.6X1A | Poisoning by alpha-adrenoreceptor antagonists, accidental (unintentional), initial encounter | | |
| T44.6X2A | Poisoning by alpha-adrenoreceptor antagonists, intentional self-harm, initial encounter | | |
| T44.6X3A | Poisoning by alpha-adrenoreceptor antagonists, assault, initial encounter | | |
| T44.6X4A | Poisoning by alpha-adrenoreceptor antagonists, undetermined, initial encounter | | |
| T44.6X5A | Adverse effect of alpha-adrenoreceptor antagonists, initial encounter | | |

© 2019 Vashi AA et al. *JAMA Network Open.*

| | | | |
|---|---|---|---|
| T44.7X1A | Poisoning by beta-adrenoreceptor antagonists, accidental (unintentional), initial encounter | | |
| T44.7X2A | Poisoning by beta-adrenoreceptor antagonists, intentional self-harm, initial encounter | | |
| T44.7X3A | Poisoning by beta-adrenoreceptor antagonists, assault, initial encounter | | |
| T44.7X4A | Poisoning by beta-adrenoreceptor antagonists, undetermined, initial encounter | | |
| T44.7X5A | Adverse effect of beta-adrenoreceptor antagonists, initial encounter | | |
| T44.8X1A | Poisoning by centrally-acting and adrenergic-neuron-blocking agents, accidental (unintentional), initial encounter | | |
| T44.8X2A | Poisoning by centrally-acting and adrenergic-neuron-blocking agents, intentional self-harm, initial encounter | | |
| T44.8X3A | Poisoning by centrally-acting and adrenergic-neuron-blocking agents, assault, initial encounter | | |
| T44.8X4A | Poisoning by centrally-acting and adrenergic-neuron-blocking agents, undetermined, initial encounter | | |
| T44.8X5A | Adverse effect of centrally-acting and adrenergic-neuron-blocking agents, initial encounter | | |
| T44.901A | Poisoning by unspecified drugs primarily affecting the autonomic nervous system, accidental (unintentional), initial encounter | | |
| T44.902A | Poisoning by unspecified drugs primarily affecting the autonomic nervous system, intentional self-harm, initial encounter | | |
| T44.903A | Poisoning by unspecified drugs primarily affecting the autonomic nervous system, assault, initial encounter | | |
| T44.904A | Poisoning by unspecified drugs primarily affecting the autonomic nervous system, undetermined, initial encounter | | |
| T44.905A | Adverse effect of unspecified drugs primarily affecting the autonomic nervous system, initial encounter | | |
| T44.991A | Poisoning by other drug primarily affecting the autonomic nervous system, accidental (unintentional), initial encounter | | |
| T44.992A | Poisoning by other drug primarily affecting the autonomic nervous system, intentional self-harm, initial encounter | | |

© 2019 Vashi AA et al. *JAMA Network Open.*

| | | | |
|---|---|---|---|
| T44.993A | Poisoning by other drug primarily affecting the autonomic nervous system, assault, initial encounter | | |
| T44.994A | Poisoning by other drug primarily affecting the autonomic nervous system, undetermined, initial encounter | | |
| T44.995A | Adverse effect of other drug primarily affecting the autonomic nervous system, initial encounter | | |
| T46.0X1A | Poisoning by cardiac-stimulant glycosides and drugs of similar action, accidental (unintentional), initial encounter | | |
| T46.0X2A | Poisoning by cardiac-stimulant glycosides and drugs of similar action, intentional self-harm, initial encounter | | |
| T46.0X3A | Poisoning by cardiac-stimulant glycosides and drugs of similar action, assault, initial encounter | | |
| T46.0X4A | Poisoning by cardiac-stimulant glycosides and drugs of similar action, undetermined, initial encounter | | |
| T46.0X5A | Adverse effect of cardiac-stimulant glycosides and drugs of similar action, initial encounter | | |
| T46.1X1A | Poisoning by calcium-channel blockers, accidental (unintentional), initial encounter | | |
| T46.1X2A | Poisoning by calcium-channel blockers, intentional self-harm, initial encounter | | |
| T46.1X3A | Poisoning by calcium-channel blockers, assault, initial encounter | | |
| T46.1X4A | Poisoning by calcium-channel blockers, undetermined, initial encounter | | |
| T46.1X5A | Adverse effect of calcium-channel blockers, initial encounter | | |
| T46.2X1A | Poisoning by other antidysrhythmic drugs, accidental (unintentional), initial encounter | | |
| T46.2X2A | Poisoning by other antidysrhythmic drugs, intentional self-harm, initial encounter | | |
| T46.2X3A | Poisoning by other antidysrhythmic drugs, assault, initial encounter | | |
| T46.2X4A | Poisoning by other antidysrhythmic drugs, undetermined, initial encounter | | |
| T46.2X5A | Adverse effect of other antidysrhythmic drugs, initial encounter | | |
| T46.3X1A | Poisoning by coronary vasodilators, accidental (unintentional), initial encounter | | |

© 2019 Vashi AA et al. *JAMA Network Open.*

| | | | |
|---|---|---|---|
| T46.3X2A | Poisoning by coronary vasodilators, intentional self-harm, initial encounter | | |
| T46.3X3A | Poisoning by coronary vasodilators, assault, initial encounter | | |
| T46.3X4A | Poisoning by coronary vasodilators, undetermined, initial encounter | | |
| T46.3X5A | Adverse effect of coronary vasodilators, initial encounter | | |
| T46.4X1A | Poisoning by angiotensin-converting-enzyme inhibitors, accidental (unintentional), initial encounter | | |
| T46.4X2A | Poisoning by angiotensin-converting-enzyme inhibitors, intentional self-harm, initial encounter | | |
| T46.4X3A | Poisoning by angiotensin-converting-enzyme inhibitors, assault, initial encounter | | |
| T46.4X4A | Poisoning by angiotensin-converting-enzyme inhibitors, undetermined, initial encounter | | |
| T46.4X5A | Adverse effect of angiotensin-converting-enzyme inhibitors, initial encounter | | |
| T46.5X1A | Poisoning by other antihypertensive drugs, accidental (unintentional), initial encounter | | |
| T46.5X2A | Poisoning by other antihypertensive drugs, intentional self-harm, initial encounter | | |
| T46.5X3A | Poisoning by other antihypertensive drugs, assault, initial encounter | | |
| T46.5X4A | Poisoning by other antihypertensive drugs, undetermined, initial encounter | | |
| T46.5X5A | Adverse effect of other antihypertensive drugs, initial encounter | | |
| T46.6X1A | Poisoning by antihyperlipidemic and antiarteriosclerotic drugs, accidental (unintentional), initial encounter | | |
| T46.6X2A | Poisoning by antihyperlipidemic and antiarteriosclerotic drugs, intentional self-harm, initial encounter | | |
| T46.6X3A | Poisoning by antihyperlipidemic and antiarteriosclerotic drugs, assault, initial encounter | | |
| T46.6X4A | Poisoning by antihyperlipidemic and antiarteriosclerotic drugs, undetermined, initial encounter | | |
| T46.6X5A | Adverse effect of antihyperlipidemic and antiarteriosclerotic drugs, initial encounter | | |
| T46.7X1A | Poisoning by peripheral vasodilators, accidental (unintentional), initial encounter | | |

© 2019 Vashi AA et al. *JAMA Network Open.*

| | | | |
|---|---|---|---|
| T46.7X2A | Poisoning by peripheral vasodilators, intentional self-harm, initial encounter | | |
| T46.7X3A | Poisoning by peripheral vasodilators, assault, initial encounter | | |
| T46.7X4A | Poisoning by peripheral vasodilators, undetermined, initial encounter | | |
| T46.7X5A | Adverse effect of peripheral vasodilators, initial encounter | | |
| T46.8X1A | Poisoning by antivaricose drugs, including sclerosing agents, accidental (unintentional), initial encounter | | |
| T46.8X2A | Poisoning by antivaricose drugs, including sclerosing agents, intentional self-harm, initial encounter | | |
| T46.8X3A | Poisoning by antivaricose drugs, including sclerosing agents, assault, initial encounter | | |
| T46.8X4A | Poisoning by antivaricose drugs, including sclerosing agents, undetermined, initial encounter | | |
| T46.8X5A | Adverse effect of antivaricose drugs, including sclerosing agents, initial encounter | | |
| T46.901A | Poisoning by unspecified agents primarily affecting the cardiovascular system, accidental (unintentional), initial encounter | | |
| T46.902A | Poisoning by unspecified agents primarily affecting the cardiovascular system, intentional self-harm, initial encounter | | |
| T46.903A | Poisoning by unspecified agents primarily affecting the cardiovascular system, assault, initial encounter | | |
| T46.904A | Poisoning by unspecified agents primarily affecting the cardiovascular system, undetermined, initial encounter | | |
| T46.905A | Adverse effect of unspecified agents primarily affecting the cardiovascular system, initial encounter | | |
| T46.991A | Poisoning by other agents primarily affecting the cardiovascular system, accidental (unintentional), initial encounter | | |
| T46.992A | Poisoning by other agents primarily affecting the cardiovascular system, intentional self-harm, initial encounter | | |
| T46.993A | Poisoning by other agents primarily affecting the cardiovascular system, assault, initial encounter | | |

© 2019 Vashi AA et al. *JAMA Network Open.*

| | | | |
|---|---|---|---|
| T46.994A | Poisoning by other agents primarily affecting the cardiovascular system, undetermined, initial encounter | | |
| T46.995A | Adverse effect of other agents primarily affecting the cardiovascular system, initial encounter | | |
| T51.0X1A | Toxic effect of ethanol, accidental (unintentional), initial encounter | | |
| T51.0X2A | Toxic effect of ethanol, intentional self-harm, initial encounter | | |
| T51.0X3A | Toxic effect of ethanol, assault, initial encounter | | |
| T51.0X4A | Toxic effect of ethanol, undetermined, initial encounter | | |
| T51.1X1A | Toxic effect of methanol, accidental (unintentional), initial encounter | | |
| T51.1X2A | Toxic effect of methanol, intentional self-harm, initial encounter | | |
| T51.1X3A | Toxic effect of methanol, assault, initial encounter | | |
| T51.1X4A | Toxic effect of methanol, undetermined, initial encounter | | |
| T51.2X1A | Toxic effect of 2-Propanol, accidental (unintentional), initial encounter | | |
| T51.2X2A | Toxic effect of 2-Propanol, intentional self-harm, initial encounter | | |
| T51.2X3A | Toxic effect of 2-Propanol, assault, initial encounter | | |
| T51.2X4A | Toxic effect of 2-Propanol, undetermined, initial encounter | | |
| T51.3X1A | Toxic effect of fusel oil, accidental (unintentional), initial encounter | | |
| T51.3X2A | Toxic effect of fusel oil, intentional self-harm, initial encounter | | |
| T51.3X3A | Toxic effect of fusel oil, assault, initial encounter | | |
| T51.3X4A | Toxic effect of fusel oil, undetermined, initial encounter | | |
| T51.8X1A | Toxic effect of other alcohols, accidental (unintentional), initial encounter | | |
| T51.8X2A | Toxic effect of other alcohols, intentional self-harm, initial encounter | | |
| T51.8X3A | Toxic effect of other alcohols, assault, initial encounter | | |
| T51.8X4A | Toxic effect of other alcohols, undetermined, initial encounter | | |
| T51.91XA | Toxic effect of unspecified alcohol, accidental (unintentional), initial encounter | | |

© 2019 Vashi AA et al. *JAMA Network Open.*

| | | | |
|---|---|---|---|
| T51.92XA | Toxic effect of unspecified alcohol, intentional self-harm, initial encounter | | |
| T51.93XA | Toxic effect of unspecified alcohol, assault, initial encounter | | |
| T51.94XA | Toxic effect of unspecified alcohol, undetermined, initial encounter | | |
| T52.0X1A | Toxic effect of petroleum products, accidental (unintentional), initial encounter | | |
| T52.0X2A | Toxic effect of petroleum products, intentional self-harm, initial encounter | | |
| T52.0X3A | Toxic effect of petroleum products, assault, initial encounter | | |
| T52.0X4A | Toxic effect of petroleum products, undetermined, initial encounter | | |
| T52.1X1A | Toxic effect of benzene, accidental (unintentional), initial encounter | | |
| T52.1X2A | Toxic effect of benzene, intentional self-harm, initial encounter | | |
| T52.1X3A | Toxic effect of benzene, assault, initial encounter | | |
| T52.1X4A | Toxic effect of benzene, undetermined, initial encounter | | |
| T52.2X1A | Toxic effect of homologues of benzene, accidental (unintentional), initial encounter | | |
| T52.2X2A | Toxic effect of homologues of benzene, intentional self-harm, initial encounter | | |
| T52.2X3A | Toxic effect of homologues of benzene, assault, initial encounter | | |
| T52.2X4A | Toxic effect of homologues of benzene, undetermined, initial encounter | | |
| T52.3X1A | Toxic effect of glycols, accidental (unintentional), initial encounter | | |
| T52.3X2A | Toxic effect of glycols, intentional self-harm, initial encounter | | |
| T52.3X3A | Toxic effect of glycols, assault, initial encounter | | |
| T52.3X4A | Toxic effect of glycols, undetermined, initial encounter | | |
| T52.4X1A | Toxic effect of ketones, accidental (unintentional), initial encounter | | |
| T52.4X2A | Toxic effect of ketones, intentional self-harm, initial encounter | | |
| T52.4X3A | Toxic effect of ketones, assault, initial encounter | | |

© 2019 Vashi AA et al. *JAMA Network Open.*

| | | | |
|---|---|---|---|
| T52.4X4A | Toxic effect of ketones, undetermined, initial encounter | | |
| T52.8X1A | Toxic effect of other organic solvents, accidental (unintentional), initial encounter | | |
| T52.8X2A | Toxic effect of other organic solvents, intentional self-harm, initial encounter | | |
| T52.8X3A | Toxic effect of other organic solvents, assault, initial encounter | | |
| T52.8X4A | Toxic effect of other organic solvents, undetermined, initial encounter | | |
| T52.91XA | Toxic effect of unspecified organic solvent, accidental (unintentional), initial encounter | | |
| T52.92XA | Toxic effect of unspecified organic solvent, intentional self-harm, initial encounter | | |
| T52.93XA | Toxic effect of unspecified organic solvent, assault, initial encounter | | |
| T52.94XA | Toxic effect of unspecified organic solvent, undetermined, initial encounter | | |
| T53.0X1A | Toxic effect of carbon tetrachloride, accidental (unintentional), initial encounter | | |
| T53.0X2A | Toxic effect of carbon tetrachloride, intentional self-harm, initial encounter | | |
| T53.0X3A | Toxic effect of carbon tetrachloride, assault, initial encounter | | |
| T53.0X4A | Toxic effect of carbon tetrachloride, undetermined, initial encounter | | |
| T53.1X1A | Toxic effect of chloroform, accidental (unintentional), initial encounter | | |
| T53.1X2A | Toxic effect of chloroform, intentional self-harm, initial encounter | | |
| T53.1X3A | Toxic effect of chloroform, assault, initial encounter | | |
| T53.1X4A | Toxic effect of chloroform, undetermined, initial encounter | | |
| T53.2X1A | Toxic effect of trichloroethylene, accidental (unintentional), initial encounter | | |
| T53.2X2A | Toxic effect of trichloroethylene, intentional self-harm, initial encounter | | |
| T53.2X3A | Toxic effect of trichloroethylene, assault, initial encounter | | |
| T53.2X4A | Toxic effect of trichloroethylene, undetermined, initial encounter | | |

© 2019 Vashi AA et al. *JAMA Network Open.*

| | | | |
|---|---|---|---|
| T53.3X1A | Toxic effect of tetrachloroethylene, accidental (unintentional), initial encounter | | |
| T53.3X2A | Toxic effect of tetrachloroethylene, intentional self-harm, initial encounter | | |
| T53.3X3A | Toxic effect of tetrachloroethylene, assault, initial encounter | | |
| T53.3X4A | Toxic effect of tetrachloroethylene, undetermined, initial encounter | | |
| T53.4X1A | Toxic effect of dichloromethane, accidental (unintentional), initial encounter | | |
| T53.4X2A | Toxic effect of dichloromethane, intentional self-harm, initial encounter | | |
| T53.4X3A | Toxic effect of dichloromethane, assault, initial encounter | | |
| T53.4X4A | Toxic effect of dichloromethane, undetermined, initial encounter | | |
| T53.5X1A | Toxic effect of chlorofluorocarbons, accidental (unintentional), initial encounter | | |
| T53.5X2A | Toxic effect of chlorofluorocarbons, intentional self-harm, initial encounter | | |
| T53.5X3A | Toxic effect of chlorofluorocarbons, assault, initial encounter | | |
| T53.5X4A | Toxic effect of chlorofluorocarbons, undetermined, initial encounter | | |
| T53.6X1A | Toxic effect of other halogen derivatives of aliphatic hydrocarbons, accidental (unintentional), initial encounter | | |
| T53.6X2A | Toxic effect of other halogen derivatives of aliphatic hydrocarbons, intentional self-harm, initial encounter | | |
| T53.6X3A | Toxic effect of other halogen derivatives of aliphatic hydrocarbons, assault, initial encounter | | |
| T53.6X4A | Toxic effect of other halogen derivatives of aliphatic hydrocarbons, undetermined, initial encounter | | |
| T53.7X1A | Toxic effect of other halogen derivatives of aromatic hydrocarbons, accidental (unintentional), initial encounter | | |
| T53.7X2A | Toxic effect of other halogen derivatives of aromatic hydrocarbons, intentional self-harm, initial encounter | | |
| T53.7X3A | Toxic effect of other halogen derivatives of aromatic hydrocarbons, assault, initial encounter | | |
| T53.7X4A | Toxic effect of other halogen derivatives of aromatic hydrocarbons, undetermined, initial encounter | | |

© 2019 Vashi AA et al. *JAMA Network Open.*

| | | | |
|---|---|---|---|
| T53.91XA | Toxic effect of unspecified halogen derivatives of aliphatic and aromatic hydrocarbons, accidental (unintentional), initial encounter | | |
| T53.92XA | Toxic effect of unspecified halogen derivatives of aliphatic and aromatic hydrocarbons, intentional self-harm, initial encounter | | |
| T53.93XA | Toxic effect of unspecified halogen derivatives of aliphatic and aromatic hydrocarbons, assault, initial encounter | | |
| T53.94XA | Toxic effect of unspecified halogen derivatives of aliphatic and aromatic hydrocarbons, undetermined, initial encounter | | |
| T54.0X1A | Toxic effect of phenol and phenol homologues, accidental (unintentional), initial encounter | | |
| T54.0X2A | Toxic effect of phenol and phenol homologues, intentional self-harm, initial encounter | | |
| T54.0X3A | Toxic effect of phenol and phenol homologues, assault, initial encounter | | |
| T54.0X4A | Toxic effect of phenol and phenol homologues, undetermined, initial encounter | | |
| T54.1X1A | Toxic effect of other corrosive organic compounds, accidental (unintentional), initial encounter | | |
| T54.1X2A | Toxic effect of other corrosive organic compounds, intentional self-harm, initial encounter | | |
| T54.1X3A | Toxic effect of other corrosive organic compounds, assault, initial encounter | | |
| T54.1X4A | Toxic effect of other corrosive organic compounds, undetermined, initial encounter | | |
| T54.2X1A | Toxic effect of corrosive acids and acid-like substances, accidental (unintentional), initial encounter | | |
| T54.2X2A | Toxic effect of corrosive acids and acid-like substances, intentional self-harm, initial encounter | | |
| T54.2X3A | Toxic effect of corrosive acids and acid-like substances, assault, initial encounter | | |
| T54.2X4A | Toxic effect of corrosive acids and acid-like substances, undetermined, initial encounter | | |
| T54.3X1A | Toxic effect of corrosive alkalis and alkali-like substances, accidental (unintentional), initial encounter | | |

© 2019 Vashi AA et al. *JAMA Network Open.*

| | | | |
|---|---|---|---|
| T54.3X2A | Toxic effect of corrosive alkalis and alkali-like substances, intentional self-harm, initial encounter | | |
| T54.3X3A | Toxic effect of corrosive alkalis and alkali-like substances, assault, initial encounter | | |
| T54.3X4A | Toxic effect of corrosive alkalis and alkali-like substances, undetermined, initial encounter | | |
| T54.91XA | Toxic effect of unspecified corrosive substance, accidental (unintentional), initial encounter | | |
| T54.92XA | Toxic effect of unspecified corrosive substance, intentional self-harm, initial encounter | | |
| T54.93XA | Toxic effect of unspecified corrosive substance, assault, initial encounter | | |
| T54.94XA | Toxic effect of unspecified corrosive substance, undetermined, initial encounter | | |
| T57.0X1A | Toxic effect of arsenic and its compounds, accidental (unintentional), initial encounter | | |
| T57.0X2A | Toxic effect of arsenic and its compounds, intentional self-harm, initial encounter | | |
| T57.0X3A | Toxic effect of arsenic and its compounds, assault, initial encounter | | |
| T57.0X4A | Toxic effect of arsenic and its compounds, undetermined, initial encounter | | |
| T57.1X1A | Toxic effect of phosphorus and its compounds, accidental (unintentional), initial encounter | | |
| T57.1X2A | Toxic effect of phosphorus and its compounds, intentional self-harm, initial encounter | | |
| T57.1X3A | Toxic effect of phosphorus and its compounds, assault, initial encounter | | |
| T57.1X4A | Toxic effect of phosphorus and its compounds, undetermined, initial encounter | | |
| T57.2X1A | Toxic effect of manganese and its compounds, accidental (unintentional), initial encounter | | |
| T57.2X2A | Toxic effect of manganese and its compounds, intentional self-harm, initial encounter | | |
| T57.2X3A | Toxic effect of manganese and its compounds, assault, initial encounter | | |
| T57.2X4A | Toxic effect of manganese and its compounds, undetermined, initial encounter | | |

© 2019 Vashi AA et al. *JAMA Network Open.*

| | | | |
|---|---|---|---|
| T57.3X1A | Toxic effect of hydrogen cyanide, accidental (unintentional), initial encounter | | |
| T57.3X2A | Toxic effect of hydrogen cyanide, intentional self-harm, initial encounter | | |
| T57.3X3A | Toxic effect of hydrogen cyanide, assault, initial encounter | | |
| T57.3X4A | Toxic effect of hydrogen cyanide, undetermined, initial encounter | | |
| T57.8X1A | Toxic effect of other specified inorganic substances, accidental (unintentional), initial encounter | | |
| T57.8X2A | Toxic effect of other specified inorganic substances, intentional self-harm, initial encounter | | |
| T57.8X3A | Toxic effect of other specified inorganic substances, assault, initial encounter | | |
| T57.8X4A | Toxic effect of other specified inorganic substances, undetermined, initial encounter | | |
| T57.91XA | Toxic effect of unspecified inorganic substance, accidental (unintentional), initial encounter | | |
| T57.92XA | Toxic effect of unspecified inorganic substance, intentional self-harm, initial encounter | | |
| T57.93XA | Toxic effect of unspecified inorganic substance, assault, initial encounter | | |
| T57.94XA | Toxic effect of unspecified inorganic substance, undetermined, initial encounter | | |
| T58.01XA | Toxic effect of carbon monoxide from motor vehicle exhaust, accidental (unintentional), initial encounter | | |
| T58.02XA | Toxic effect of carbon monoxide from motor vehicle exhaust, intentional self-harm, initial encounter | | |
| T58.03XA | Toxic effect of carbon monoxide from motor vehicle exhaust, assault, initial encounter | | |
| T58.04XA | Toxic effect of carbon monoxide from motor vehicle exhaust, undetermined, initial encounter | | |
| T58.11XA | Toxic effect of carbon monoxide from utility gas, accidental (unintentional), initial encounter | | |
| T58.12XA | Toxic effect of carbon monoxide from utility gas, intentional self-harm, initial encounter | | |
| T58.13XA | Toxic effect of carbon monoxide from utility gas, assault, initial encounter | | |

© 2019 Vashi AA et al. *JAMA Network Open.*

| | | | |
|---|---|---|---|
| T58.14XA | Toxic effect of carbon monoxide from utility gas, undetermined, initial encounter | | |
| T58.2X1A | Toxic effect of carbon monoxide from incomplete combustion of other domestic fuels, accidental (unintentional), initial encounter | | |
| T58.2X2A | Toxic effect of carbon monoxide from incomplete combustion of other domestic fuels, intentional self-harm, initial encounter | | |
| T58.2X3A | Toxic effect of carbon monoxide from incomplete combustion of other domestic fuels, assault, initial encounter | | |
| T58.2X4A | Toxic effect of carbon monoxide from incomplete combustion of other domestic fuels, undetermined, initial encounter | | |
| T58.8X1A | Toxic effect of carbon monoxide from other source, accidental (unintentional), initial encounter | | |
| T58.8X2A | Toxic effect of carbon monoxide from other source, intentional self-harm, initial encounter | | |
| T58.8X3A | Toxic effect of carbon monoxide from other source, assault, initial encounter | | |
| T58.8X4A | Toxic effect of carbon monoxide from other source, undetermined, initial encounter | | |
| T58.91XA | Toxic effect of carbon monoxide from unspecified source, accidental (unintentional), initial encounter | | |
| T58.92XA | Toxic effect of carbon monoxide from unspecified source, intentional self-harm, initial encounter | | |
| T58.93XA | Toxic effect of carbon monoxide from unspecified source, assault, initial encounter | | |
| T58.94XA | Toxic effect of carbon monoxide from unspecified source, undetermined, initial encounter | | |
| T59.0X1A | Toxic effect of nitrogen oxides, accidental (unintentional), initial encounter | | |
| T59.0X2A | Toxic effect of nitrogen oxides, intentional self-harm, initial encounter | | |
| T59.0X3A | Toxic effect of nitrogen oxides, assault, initial encounter | | |
| T59.0X4A | Toxic effect of nitrogen oxides, undetermined, initial encounter | | |
| T59.1X1A | Toxic effect of sulfur dioxide, accidental (unintentional), initial encounter | | |

© 2019 Vashi AA et al. *JAMA Network Open.*

| | | | |
|---|---|---|---|
| T59.1X2A | Toxic effect of sulfur dioxide, intentional self-harm, initial encounter | | |
| T59.1X3A | Toxic effect of sulfur dioxide, assault, initial encounter | | |
| T59.1X4A | Toxic effect of sulfur dioxide, undetermined, initial encounter | | |
| T59.2X1A | Toxic effect of formaldehyde, accidental (unintentional), initial encounter | | |
| T59.2X2A | Toxic effect of formaldehyde, intentional self-harm, initial encounter | | |
| T59.2X3A | Toxic effect of formaldehyde, assault, initial encounter | | |
| T59.2X4A | Toxic effect of formaldehyde, undetermined, initial encounter | | |
| T59.3X1A | Toxic effect of lacrimogenic gas, accidental (unintentional), initial encounter | | |
| T59.3X2A | Toxic effect of lacrimogenic gas, intentional self-harm, initial encounter | | |
| T59.3X3A | Toxic effect of lacrimogenic gas, assault, initial encounter | | |
| T59.3X4A | Toxic effect of lacrimogenic gas, undetermined, initial encounter | | |
| T59.4X1A | Toxic effect of chlorine gas, accidental (unintentional), initial encounter | | |
| T59.4X2A | Toxic effect of chlorine gas, intentional self-harm, initial encounter | | |
| T59.4X3A | Toxic effect of chlorine gas, assault, initial encounter | | |
| T59.4X4A | Toxic effect of chlorine gas, undetermined, initial encounter | | |
| T59.5X1A | Toxic effect of fluorine gas and hydrogen fluoride, accidental (unintentional), initial encounter | | |
| T59.5X2A | Toxic effect of fluorine gas and hydrogen fluoride, intentional self-harm, initial encounter | | |
| T59.5X3A | Toxic effect of fluorine gas and hydrogen fluoride, assault, initial encounter | | |
| T59.5X4A | Toxic effect of fluorine gas and hydrogen fluoride, undetermined, initial encounter | | |
| T59.6X1A | Toxic effect of hydrogen sulfide, accidental (unintentional), initial encounter | | |
| T59.6X2A | Toxic effect of hydrogen sulfide, intentional self-harm, initial encounter | | |
| T59.6X3A | Toxic effect of hydrogen sulfide, assault, initial encounter | | |

© 2019 Vashi AA et al. *JAMA Network Open.*

| | | | |
|---|---|---|---|
| T59.6X4A | Toxic effect of hydrogen sulfide, undetermined, initial encounter | | |
| T59.7X1A | Toxic effect of carbon dioxide, accidental (unintentional), initial encounter | | |
| T59.7X2A | Toxic effect of carbon dioxide, intentional self-harm, initial encounter | | |
| T59.7X3A | Toxic effect of carbon dioxide, assault, initial encounter | | |
| T59.7X4A | Toxic effect of carbon dioxide, undetermined, initial encounter | | |
| T59.811A | Toxic effect of smoke, accidental (unintentional), initial encounter | | |
| T59.812A | Toxic effect of smoke, intentional self-harm, initial encounter | | |
| T59.813A | Toxic effect of smoke, assault, initial encounter | | |
| T59.814A | Toxic effect of smoke, undetermined, initial encounter | | |
| T59.891A | Toxic effect of other specified gases, fumes and vapors, accidental (unintentional), initial encounter | | |
| T59.892A | Toxic effect of other specified gases, fumes and vapors, intentional self-harm, initial encounter | | |
| T59.893A | Toxic effect of other specified gases, fumes and vapors, assault, initial encounter | | |
| T59.894A | Toxic effect of other specified gases, fumes and vapors, undetermined, initial encounter | | |
| T59.91XA | Toxic effect of unspecified gases, fumes and vapors, accidental (unintentional), initial encounter | | |
| T59.92XA | Toxic effect of unspecified gases, fumes and vapors, intentional self-harm, initial encounter | | |
| T59.93XA | Toxic effect of unspecified gases, fumes and vapors, assault, initial encounter | | |
| T59.94XA | Toxic effect of unspecified gases, fumes and vapors, undetermined, initial encounter | | |
| T60.0X1A | Toxic effect of organophosphate and carbamate insecticides, accidental (unintentional), initial encounter | | |
| T60.0X2A | Toxic effect of organophosphate and carbamate insecticides, intentional self-harm, initial encounter | | |
| T60.0X3A | Toxic effect of organophosphate and carbamate insecticides, assault, initial encounter | | |
| T60.0X4A | Toxic effect of organophosphate and carbamate insecticides, undetermined, initial encounter | | |

© 2019 Vashi AA et al. *JAMA Network Open.*

| | | | |
|---|---|---|---|
| T60.1X1A | Toxic effect of halogenated insecticides, accidental (unintentional), initial encounter | | |
| T60.1X2A | Toxic effect of halogenated insecticides, intentional self-harm, initial encounter | | |
| T60.1X3A | Toxic effect of halogenated insecticides, assault, initial encounter | | |
| T60.1X4A | Toxic effect of halogenated insecticides, undetermined, initial encounter | | |
| T60.2X1A | Toxic effect of other insecticides, accidental (unintentional), initial encounter | | |
| T60.2X2A | Toxic effect of other insecticides, intentional self-harm, initial encounter | | |
| T60.2X3A | Toxic effect of other insecticides, assault, initial encounter | | |
| T60.2X4A | Toxic effect of other insecticides, undetermined, initial encounter | | |
| T60.3X1A | Toxic effect of herbicides and fungicides, accidental (unintentional), initial encounter | | |
| T60.3X2A | Toxic effect of herbicides and fungicides, intentional self-harm, initial encounter | | |
| T60.3X3A | Toxic effect of herbicides and fungicides, assault, initial encounter | | |
| T60.3X4A | Toxic effect of herbicides and fungicides, undetermined, initial encounter | | |
| T60.4X1A | Toxic effect of rodenticides, accidental (unintentional), initial encounter | | |
| T60.4X2A | Toxic effect of rodenticides, intentional self-harm, initial encounter | | |
| T60.4X3A | Toxic effect of rodenticides, assault, initial encounter | | |
| T60.4X4A | Toxic effect of rodenticides, undetermined, initial encounter | | |
| T60.8X1A | Toxic effect of other pesticides, accidental (unintentional), initial encounter | | |
| T60.8X2A | Toxic effect of other pesticides, intentional self-harm, initial encounter | | |
| T60.8X3A | Toxic effect of other pesticides, assault, initial encounter | | |
| T60.8X4A | Toxic effect of other pesticides, undetermined, initial encounter | | |

© 2019 Vashi AA et al. *JAMA Network Open.*

| | | | |
|---|---|---|---|
| T60.91XA | Toxic effect of unspecified pesticide, accidental (unintentional), initial encounter | | |
| T60.92XA | Toxic effect of unspecified pesticide, intentional self-harm, initial encounter | | |
| T60.93XA | Toxic effect of unspecified pesticide, assault, initial encounter | | |
| T60.94XA | Toxic effect of unspecified pesticide, undetermined, initial encounter | | |

© 2019 Vashi AA et al. *JAMA Network Open.*

**Included and Excluded ICD-10-CM Diagnosis Subcodes for Paralytic Ileus and Intestinal Obstruction without Hernia Condition Group**

| Included ICD-10-CM diagnosis subcodes (n=10) | | Excluded ICD-10-CM diagnosis subcodes (n=0) | |
|---|---|---|---|
| **Subcode** | **Subcode description** | **Subcode** | **Subcode description** |
| K56.0 | Paralytic ileus | | |
| K56.1 | Intussusception | | |
| K56.2 | Volvulus | | |
| K56.3 | Gallstone ileus | | |
| K56.41 | Fecal impaction | | |
| K56.49 | Other impaction of intestine | | |
| K56.5 | Intestinal adhesions [bands] with obstruction (postprocedural) (postinfection) | | |
| K56.60 | Unspecified intestinal obstruction | | |
| K56.69 | Other intestinal obstruction | | |
| K56.7 | Ileus, unspecified | | |

© 2019 Vashi AA et al. *JAMA Network Open.*

## Included and Excluded ICD-10-CM Diagnosis Subcodes for Pericardial Disease, Endocarditis, and Myocarditis Condition Group

| Included ICD-10-CM diagnosis subcodes (n=26) | | Excluded ICD-10-CM diagnosis subcodes (n=0) | |
|---|---|---|---|
| **Subcode** | **Subcode description** | **Subcode** | **Subcode description** |
| A01.02 | Typhoid fever with heart involvement | | |
| A32.82 | Listerial endocarditis | | |
| A39.50 | Meningococcal carditis, unspecified | | |
| A39.51 | Meningococcal endocarditis | | |
| A39.52 | Meningococcal myocarditis | | |
| A39.53 | Meningococcal pericarditis | | |
| B37.6 | Candidal endocarditis | | |
| I30.0 | Acute nonspecific idiopathic pericarditis | | |
| I30.1 | Infective pericarditis | | |
| I30.8 | Other forms of acute pericarditis | | |
| I30.9 | Acute pericarditis, unspecified | | |
| I31.0 | Chronic adhesive pericarditis | | |
| I31.1 | Chronic constrictive pericarditis | | |
| I31.2 | Hemopericardium, not elsewhere classified | | |
| I31.3 | Pericardial effusion (noninflammatory) | | |
| I31.4 | Cardiac tamponade | | |
| I31.8 | Other specified diseases of pericardium | | |
| I31.9 | Disease of pericardium, unspecified | | |
| I32. | Pericarditis in diseases classified elsewhere | | |
| I33.0 | Acute and subacute infective endocarditis | | |
| I33.9 | Acute and subacute endocarditis, unspecified | | |
| I40.0 | Infective myocarditis | | |
| I40.1 | Isolated myocarditis | | |
| I40.8 | Other acute myocarditis | | |
| I40.9 | Acute myocarditis, unspecified | | |
| I41. | Myocarditis in diseases classified elsewhere | | |

© 2019 Vashi AA et al. *JAMA Network Open.*

## Included and Excluded ICD-10-CM Diagnosis Subcodes for Peritonitis Condition Group

| Included ICD-10-CM diagnosis subcodes (n=8) | | Excluded ICD-10-CM diagnosis subcodes (n=0) | |
|---|---|---|---|
| **Subcode** | **Subcode description** | **Subcode** | **Subcode description** |
| A54.85 | Gonococcal peritonitis | | |
| K65.0 | Generalized (acute) peritonitis | | |
| K65.1 | Peritoneal abscess | | |
| K65.2 | Spontaneous bacterial peritonitis | | |
| K65.3 | Choleperitonitis | | |
| K65.4 | Sclerosing mesenteritis | | |
| K65.8 | Other peritonitis | | |
| K65.9 | Peritonitis, unspecified | | |

© 2019 Vashi AA et al. *JAMA Network Open.*

## Included and Excluded ICD-10-CM Diagnosis Subcodes for Pneumonia Condition Group

| Included ICD-10-CM diagnosis subcodes (n=37) | | Excluded ICD-10-CM diagnosis subcodes (n=0) | |
|---|---|---|---|
| **Subcode** | **Subcode description** | **Subcode** | **Subcode description** |
| A01.03 | Typhoid pneumonia | | |
| A02.22 | Salmonella pneumonia | | |
| A20.2 | Pneumonic plague | | |
| A22.1 | Pulmonary anthrax | | |
| A42.0 | Pulmonary actinomycosis | | |
| A54.84 | Gonococcal pneumonia | | |
| B37.1 | Pulmonary candidiasis | | |
| J12.0 | Adenoviral pneumonia | | |
| J12.1 | Respiratory syncytial virus pneumonia | | |
| J12.2 | Parainfluenza virus pneumonia | | |
| J12.3 | Human metapneumovirus pneumonia | | |
| J12.81 | Pneumonia due to SARS-associated coronavirus | | |
| J12.89 | Other viral pneumonia | | |
| J12.9 | Viral pneumonia, unspecified | | |
| J13. | Pneumonia due to Streptococcus pneumoniae | | |
| J14. | Pneumonia due to Hemophilus influenzae | | |
| J15.0 | Pneumonia due to Klebsiella pneumoniae | | |
| J15.1 | Pneumonia due to Pseudomonas | | |
| J15.20 | Pneumonia due to staphylococcus, unspecified | | |
| J15.211 | Pneumonia due to Methicillin susceptible Staphylococcus aureus | | |
| J15.212 | Pneumonia due to Methicillin resistant Staphylococcus aureus | | |
| J15.29 | Pneumonia due to other staphylococcus | | |
| J15.3 | Pneumonia due to streptococcus, group B | | |
| J15.4 | Pneumonia due to other streptococci | | |
| J15.5 | Pneumonia due to Escherichia coli | | |
| J15.6 | Pneumonia due to other aerobic Gram-negative bacteria | | |
| J15.7 | Pneumonia due to Mycoplasma pneumoniae | | |
| J15.8 | Pneumonia due to other specified bacteria | | |
| J15.9 | Unspecified bacterial pneumonia | | |

© 2019 Vashi AA et al. *JAMA Network Open.*

| | | | |
|---|---|---|---|
| J16.0 | Chlamydial pneumonia | | |
| J16.8 | Pneumonia due to other specified infectious organisms | | |
| J17. | Pneumonia in diseases classified elsewhere | | |
| J18.0 | Bronchopneumonia, unspecified organism | | |
| J18.1 | Lobar pneumonia, unspecified organism | | |
| J18.2 | Hypostatic pneumonia, unspecified organism | | |
| J18.8 | Other pneumonia, unspecified organism | | |
| J18.9 | Pneumonia, unspecified organism | | |

## Included and Excluded ICD-10-CM Diagnosis Subcodes for Pneumothorax Condition Group

| **Included ICD-10-CM diagnosis subcodes (n=5)** | | **Excluded ICD-10-CM diagnosis subcodes (n=2)** | |
|---|---|---|---|
| **Subcode** | **Subcode description** | **Subcode** | **Subcode description** |
| J93.0 | Spontaneous tension pneumothorax | J93.81 | Chronic pneumothorax |
| J93.11 | Primary spontaneous pneumothorax | J93.82 | Other air leak |
| J93.12 | Secondary spontaneous pneumothorax | | |
| J93.83 | Other pneumothorax | | |
| J93.9 | Pneumothorax, unspecified | | |

## Included and Excluded ICD-10-CM Diagnosis Subcodes for Postpartum Hemorrhage Condition Group

| **Included ICD-10-CM diagnosis subcodes (n=4)** | | **Excluded ICD-10-CM diagnosis subcodes (n=0)** | |
|---|---|---|---|
| **Subcode** | **Subcode description** | **Subcode** | **Subcode description** |
| O72.0 | Third-stage hemorrhage | | |
| O72.1 | Other immediate postpartum hemorrhage | | |
| O72.2 | Delayed and secondary postpartum hemorrhage | | |
| O72.3 | Postpartum coagulation defects | | |

© 2019 Vashi AA et al. *JAMA Network Open.*

## Included and Excluded ICD-10-CM Diagnosis Subcodes for Pre-eclampsia/Eclampsia Condition Group

| Included ICD-10-CM diagnosis subcodes (n=18) | | Excluded ICD-10-CM diagnosis subcodes (n=0) | |
|---|---|---|---|
| **Subcode** | **Subcode description** | **Subcode** | **Subcode description** |
| O14.00 | Mild to moderate pre-eclampsia, unspecified trimester | | |
| O14.02 | Mild to moderate pre-eclampsia, second trimester | | |
| O14.03 | Mild to moderate pre-eclampsia, third trimester | | |
| O14.10 | Severe pre-eclampsia, unspecified trimester | | |
| O14.12 | Severe pre-eclampsia, second trimester | | |
| O14.13 | Severe pre-eclampsia, third trimester | | |
| O14.20 | HELLP syndrome (HELLP), unspecified trimester | | |
| O14.22 | HELLP syndrome (HELLP), second trimester | | |
| O14.23 | HELLP syndrome (HELLP), third trimester | | |
| O14.90 | Unspecified pre-eclampsia, unspecified trimester | | |
| O14.92 | Unspecified pre-eclampsia, second trimester | | |
| O14.93 | Unspecified pre-eclampsia, third trimester | | |
| O15.00 | Eclampsia in pregnancy, unspecified trimester | | |
| O15.02 | Eclampsia in pregnancy, second trimester | | |
| O15.03 | Eclampsia in pregnancy, third trimester | | |
| O15.1 | Eclampsia in labor | | |
| O15.2 | Eclampsia in the puerperium | | |
| O15.9 | Eclampsia, unspecified as to time period | | |

© 2019 Vashi AA et al. *JAMA Network Open.*

## Included and Excluded ICD-10-CM Diagnosis Subcodes for Pulmonary Embolism Condition Group

| Included ICD-10-CM diagnosis subcodes (n=6) | | Excluded ICD-10-CM diagnosis subcodes (n=0) | |
|---|---|---|---|
| **Subcode** | **Subcode description** | **Subcode** | **Subcode description** |
| I26.01 | Septic pulmonary embolism with acute cor pulmonale | | |
| I26.02 | Saddle embolus of pulmonary artery with acute cor pulmonale | | |
| I26.09 | Other pulmonary embolism with acute cor pulmonale | | |
| I26.90 | Septic pulmonary embolism without acute cor pulmonale | | |
| I26.92 | Saddle embolus of pulmonary artery without acute cor pulmonale | | |
| I26.99 | Other pulmonary embolism without acute cor pulmonale | | |

## Included and Excluded ICD-10-CM Diagnosis Subcodes for Respiratory Failure Condition Group

| Included ICD-10-CM diagnosis subcodes (n=12) | | Excluded ICD-10-CM diagnosis subcodes (n=0) | |
|---|---|---|---|
| **Subcode** | **Subcode description** | **Subcode** | **Subcode description** |
| J96.00 | Acute respiratory failure, unspecified whether with hypoxia or hypercapnia | | |
| J96.01 | Acute respiratory failure with hypoxia | | |
| J96.02 | Acute respiratory failure with hypercapnia | | |
| J96.10 | Chronic respiratory failure, unspecified whether with hypoxia or hypercapnia | | |
| J96.11 | Chronic respiratory failure with hypoxia | | |
| J96.12 | Chronic respiratory failure with hypercapnia | | |
| J96.20 | Acute and chronic respiratory failure, unspecified whether with hypoxia or hypercapnia | | |
| J96.21 | Acute and chronic respiratory failure with hypoxia | | |
| J96.22 | Acute and chronic respiratory failure with hypercapnia | | |
| J96.90 | Respiratory failure, unspecified, unspecified whether with hypoxia or hypercapnia | | |
| J96.91 | Respiratory failure, unspecified with hypoxia | | |
| J96.92 | Respiratory failure, unspecified with hypercapnia | | |

© 2019 Vashi AA et al. *JAMA Network Open.*

## Included and Excluded ICD-10-CM Diagnosis Subcodes for Sepsis and Systemic Inflammatory Response Syndrome Condition Group

| Included ICD-10-CM diagnosis subcodes (n=35) | | Excluded ICD-10-CM diagnosis subcodes (n=5) | |
|---|---|---|---|
| **Subcode** | **Subcode description** | **Subcode** | **Subcode description** |
| A02.1 | Salmonella sepsis | A39.3 | Chronic meningococcemia |
| A20.7 | Septicemic plague | A39.82 | Meningococcal retrobulbar neuritis |
| A22.7 | Anthrax sepsis | A39.84 | Postmeningococcal arthritis |
| A26.7 | Erysipelothrix sepsis | A39.89 | Other meningococcal infections |
| A32.7 | Listerial sepsis | A39.9 | Meningococcal infection, unspecified |
| A39.1 | Waterhouse-Friderichsen syndrome | | |
| A39.2 | Acute meningococcemia | | |
| A39.4 | Meningococcemia, unspecified | | |
| A40.0 | Sepsis due to streptococcus, group A | | |
| A40.1 | Sepsis due to streptococcus, group B | | |
| A40.3 | Sepsis due to Streptococcus pneumoniae | | |
| A40.8 | Other streptococcal sepsis | | |
| A40.9 | Streptococcal sepsis, unspecified | | |
| A41.01 | Sepsis due to Methicillin susceptible Staphylococcus aureus | | |
| A41.02 | Sepsis due to Methicillin resistant Staphylococcus aureus | | |
| A41.1 | Sepsis due to other specified staphylococcus | | |
| A41.2 | Sepsis due to unspecified staphylococcus | | |
| A41.3 | Sepsis due to Hemophilus influenzae | | |
| A41.4 | Sepsis due to anaerobes | | |
| A41.50 | Gram-negative sepsis, unspecified | | |
| A41.51 | Sepsis due to Escherichia coli [E. coli] | | |
| A41.52 | Sepsis due to Pseudomonas | | |
| A41.53 | Sepsis due to Serratia | | |
| A41.59 | Other Gram-negative sepsis | | |
| A41.81 | Sepsis due to Enterococcus | | |
| A41.89 | Other specified sepsis | | |
| A41.9 | Sepsis, unspecified organism | | |
| A42.7 | Actinomycotic sepsis | | |

© 2019 Vashi AA et al. *JAMA Network Open.*

| | | | |
|---|---|---|---|
| A54.86 | Gonococcal sepsis | | |
| B37.7 | Candidal sepsis | | |
| R65.10 | Systemic inflammatory response syndrome (SIRS) of non-infectious origin without acute organ dysfunction | | |
| R65.11 | Systemic inflammatory response syndrome (SIRS) of non-infectious origin with acute organ dysfunction | | |
| R65.20 | Severe sepsis without septic shock | | |
| R65.21 | Severe sepsis with septic shock | | |
| R78.81 | Bacteremia | | |

© 2019 Vashi AA et al. *JAMA Network Open.*

## Included and Excluded ICD-10-CM Diagnosis Subcodes for Septic Arthritis Condition Group

| Included ICD-10-CM diagnosis subcodes (n=122) | | Excluded ICD-10-CM diagnosis subcodes (n=0) | |
|---|---|---|---|
| **Subcode** | **Subcode description** | **Subcode** | **Subcode description** |
| A39.83 | Meningococcal arthritis | | |
| M00.00 | Staphylococcal arthritis, unspecified joint | | |
| M00.011 | Staphylococcal arthritis, right shoulder | | |
| M00.012 | Staphylococcal arthritis, left shoulder | | |
| M00.019 | Staphylococcal arthritis, unspecified shoulder | | |
| M00.021 | Staphylococcal arthritis, right elbow | | |
| M00.022 | Staphylococcal arthritis, left elbow | | |
| M00.029 | Staphylococcal arthritis, unspecified elbow | | |
| M00.031 | Staphylococcal arthritis, right wrist | | |
| M00.032 | Staphylococcal arthritis, left wrist | | |
| M00.039 | Staphylococcal arthritis, unspecified wrist | | |
| M00.041 | Staphylococcal arthritis, right hand | | |
| M00.042 | Staphylococcal arthritis, left hand | | |
| M00.049 | Staphylococcal arthritis, unspecified hand | | |
| M00.051 | Staphylococcal arthritis, right hip | | |
| M00.052 | Staphylococcal arthritis, left hip | | |
| M00.059 | Staphylococcal arthritis, unspecified hip | | |
| M00.061 | Staphylococcal arthritis, right knee | | |
| M00.062 | Staphylococcal arthritis, left knee | | |
| M00.069 | Staphylococcal arthritis, unspecified knee | | |
| M00.071 | Staphylococcal arthritis, right ankle and foot | | |
| M00.072 | Staphylococcal arthritis, left ankle and foot | | |
| M00.079 | Staphylococcal arthritis, unspecified ankle and foot | | |
| M00.08 | Staphylococcal arthritis, vertebrae | | |
| M00.09 | Staphylococcal polyarthritis | | |
| M00.10 | Pneumococcal arthritis, unspecified joint | | |
| M00.111 | Pneumococcal arthritis, right shoulder | | |
| M00.112 | Pneumococcal arthritis, left shoulder | | |
| M00.119 | Pneumococcal arthritis, unspecified shoulder | | |
| M00.121 | Pneumococcal arthritis, right elbow | | |

© 2019 Vashi AA et al. *JAMA Network Open.*

| | | | |
|---|---|---|---|
| M00.122 | Pneumococcal arthritis, left elbow | | |
| M00.129 | Pneumococcal arthritis, unspecified elbow | | |
| M00.131 | Pneumococcal arthritis, right wrist | | |
| M00.132 | Pneumococcal arthritis, left wrist | | |
| M00.139 | Pneumococcal arthritis, unspecified wrist | | |
| M00.141 | Pneumococcal arthritis, right hand | | |
| M00.142 | Pneumococcal arthritis, left hand | | |
| M00.149 | Pneumococcal arthritis, unspecified hand | | |
| M00.151 | Pneumococcal arthritis, right hip | | |
| M00.152 | Pneumococcal arthritis, left hip | | |
| M00.159 | Pneumococcal arthritis, unspecified hip | | |
| M00.161 | Pneumococcal arthritis, right knee | | |
| M00.162 | Pneumococcal arthritis, left knee | | |
| M00.169 | Pneumococcal arthritis, unspecified knee | | |
| M00.171 | Pneumococcal arthritis, right ankle and foot | | |
| M00.172 | Pneumococcal arthritis, left ankle and foot | | |
| M00.179 | Pneumococcal arthritis, unspecified ankle and foot | | |
| M00.18 | Pneumococcal arthritis, vertebrae | | |
| M00.19 | Pneumococcal polyarthritis | | |
| M00.20 | Other streptococcal arthritis, unspecified joint | | |
| M00.211 | Other streptococcal arthritis, right shoulder | | |
| M00.212 | Other streptococcal arthritis, left shoulder | | |
| M00.219 | Other streptococcal arthritis, unspecified shoulder | | |
| M00.221 | Other streptococcal arthritis, right elbow | | |
| M00.222 | Other streptococcal arthritis, left elbow | | |
| M00.229 | Other streptococcal arthritis, unspecified elbow | | |
| M00.231 | Other streptococcal arthritis, right wrist | | |
| M00.232 | Other streptococcal arthritis, left wrist | | |
| M00.239 | Other streptococcal arthritis, unspecified wrist | | |
| M00.241 | Other streptococcal arthritis, right hand | | |
| M00.242 | Other streptococcal arthritis, left hand | | |
| M00.249 | Other streptococcal arthritis, unspecified hand | | |
| M00.251 | Other streptococcal arthritis, right hip | | |
| M00.252 | Other streptococcal arthritis, left hip | | |

© 2019 Vashi AA et al. *JAMA Network Open.*

| | | | |
|---|---|---|---|
| M00.259 | Other streptococcal arthritis, unspecified hip | | |
| M00.261 | Other streptococcal arthritis, right knee | | |
| M00.262 | Other streptococcal arthritis, left knee | | |
| M00.269 | Other streptococcal arthritis, unspecified knee | | |
| M00.271 | Other streptococcal arthritis, right ankle and foot | | |
| M00.272 | Other streptococcal arthritis, left ankle and foot | | |
| M00.279 | Other streptococcal arthritis, unspecified ankle and foot | | |
| M00.28 | Other streptococcal arthritis, vertebrae | | |
| M00.29 | Other streptococcal polyarthritis | | |
| M00.80 | Arthritis due to other bacteria, unspecified joint | | |
| M00.811 | Arthritis due to other bacteria, right shoulder | | |
| M00.812 | Arthritis due to other bacteria, left shoulder | | |
| M00.819 | Arthritis due to other bacteria, unspecified shoulder | | |
| M00.821 | Arthritis due to other bacteria, right elbow | | |
| M00.822 | Arthritis due to other bacteria, left elbow | | |
| M00.829 | Arthritis due to other bacteria, unspecified elbow | | |
| M00.831 | Arthritis due to other bacteria, right wrist | | |
| M00.832 | Arthritis due to other bacteria, left wrist | | |
| M00.839 | Arthritis due to other bacteria, unspecified wrist | | |
| M00.841 | Arthritis due to other bacteria, right hand | | |
| M00.842 | Arthritis due to other bacteria, left hand | | |
| M00.849 | Arthritis due to other bacteria, unspecified hand | | |
| M00.851 | Arthritis due to other bacteria, right hip | | |
| M00.852 | Arthritis due to other bacteria, left hip | | |
| M00.859 | Arthritis due to other bacteria, unspecified hip | | |
| M00.861 | Arthritis due to other bacteria, right knee | | |
| M00.862 | Arthritis due to other bacteria, left knee | | |
| M00.869 | Arthritis due to other bacteria, unspecified knee | | |
| M00.871 | Arthritis due to other bacteria, right ankle and foot | | |
| M00.872 | Arthritis due to other bacteria, left ankle and foot | | |
| M00.879 | Arthritis due to other bacteria, unspecified ankle and foot | | |
| M00.88 | Arthritis due to other bacteria, vertebrae | | |
| M00.89 | Polyarthritis due to other bacteria | | |
| M00.9 | Pyogenic arthritis, unspecified | | |

© 2019 Vashi AA et al. *JAMA Network Open.*

| | | | |
|---|---|---|---|
| M01.X0 | Direct infection of unspecified joint in infectious and parasitic diseases classified elsewhere | | |
| M01.X11 | Direct infection of right shoulder in infectious and parasitic diseases classified elsewhere | | |
| M01.X12 | Direct infection of left shoulder in infectious and parasitic diseases classified elsewhere | | |
| M01.X19 | Direct infection of unspecified shoulder in infectious and parasitic diseases classified elsewhere | | |
| M01.X21 | Direct infection of right elbow in infectious and parasitic diseases classified elsewhere | | |
| M01.X22 | Direct infection of left elbow in infectious and parasitic diseases classified elsewhere | | |
| M01.X29 | Direct infection of unspecified elbow in infectious and parasitic diseases classified elsewhere | | |
| M01.X31 | Direct infection of right wrist in infectious and parasitic diseases classified elsewhere | | |
| M01.X32 | Direct infection of left wrist in infectious and parasitic diseases classified elsewhere | | |
| M01.X39 | Direct infection of unspecified wrist in infectious and parasitic diseases classified elsewhere | | |
| M01.X41 | Direct infection of right hand in infectious and parasitic diseases classified elsewhere | | |
| M01.X42 | Direct infection of left hand in infectious and parasitic diseases classified elsewhere | | |
| M01.X49 | Direct infection of unspecified hand in infectious and parasitic diseases classified elsewhere | | |
| M01.X51 | Direct infection of right hip in infectious and parasitic diseases classified elsewhere | | |
| M01.X52 | Direct infection of left hip in infectious and parasitic diseases classified elsewhere | | |
| M01.X59 | Direct infection of unspecified hip in infectious and parasitic diseases classified elsewhere | | |
| M01.X61 | Direct infection of right knee in infectious and parasitic diseases classified elsewhere | | |
| M01.X62 | Direct infection of left knee in infectious and parasitic diseases classified elsewhere | | |
| M01.X69 | Direct infection of unspecified knee in infectious and parasitic diseases classified elsewhere | | |

© 2019 Vashi AA et al. *JAMA Network Open.*

| | | | |
|---|---|---|---|
| M01.X71 | Direct infection of right ankle and foot in infectious and parasitic diseases classified elsewhere | | |
| M01.X72 | Direct infection of left ankle and foot in infectious and parasitic diseases classified elsewhere | | |
| M01.X79 | Direct infection of unspecified ankle and foot in infectious and parasitic diseases classified elsewhere | | |
| M01.X8 | Direct infection of vertebrae in infectious and parasitic diseases classified elsewhere | | |
| M01.X9 | Direct infection of multiple joints in infectious and parasitic diseases classified elsewhere | | |

## Included and Excluded ICD-10-CM Diagnosis Subcodes for Shock Condition Group

| **Included ICD-10-CM diagnosis subcodes (n=4)** | | **Excluded ICD-10-CM diagnosis subcodes (n=0)** | |
|---|---|---|---|
| **Subcode** | **Subcode description** | **Subcode** | **Subcode description** |
| R57.0 | Cardiogenic shock | | |
| R57.1 | Hypovolemic shock | | |
| R57.8 | Other shock | | |
| R57.9 | Shock, unspecified | | |

© 2019 Vashi AA et al. *JAMA Network Open.*

## Included and Excluded ICD-10-CM Diagnosis Subcodes for Thoracic Injuries Condition Group

| Included ICD-10-CM diagnosis subcodes (n=391) | | Excluded ICD-10-CM diagnosis subcodes (n=9) | |
|---|---|---|---|
| **Subcode** | **Subcode description** | **Subcode** | **Subcode description** |
| S21.001A | Unspecified open wound of right breast, initial encounter | S22.31XA | Fracture of one rib, right side, initial encounter for closed fracture |
| S21.002A | Unspecified open wound of left breast, initial encounter | S22.31XB | Fracture of one rib, right side, initial encounter for open fracture |
| S21.009A | Unspecified open wound of unspecified breast, initial encounter | S22.32XA | Fracture of one rib, left side, initial encounter for closed fracture |
| S21.011A | Laceration without foreign body of right breast, initial encounter | S22.32XB | Fracture of one rib, left side, initial encounter for open fracture |
| S21.012A | Laceration without foreign body of left breast, initial encounter | S22.39XA | Fracture of one rib, unspecified side, initial encounter for closed fracture |
| S21.019A | Laceration without foreign body of unspecified breast, initial encounter | S22.39XB | Fracture of one rib, unspecified side, initial encounter for open fracture |
| S21.021A | Laceration with foreign body of right breast, initial encounter | S29.011A | Strain of muscle and tendon of front wall of thorax, initial encounter |
| S21.022A | Laceration with foreign body of left breast, initial encounter | S29.012A | Strain of muscle and tendon of back wall of thorax, initial encounter |
| S21.029A | Laceration with foreign body of unspecified breast, initial encounter | S29.019A | Strain of muscle and tendon of unspecified wall of thorax, initial encounter |
| S21.031A | Puncture wound without foreign body of right breast, initial encounter | | |
| S21.032A | Puncture wound without foreign body of left breast, initial encounter | | |
| S21.039A | Puncture wound without foreign body of unspecified breast, initial encounter | | |
| S21.041A | Puncture wound with foreign body of right breast, initial encounter | | |
| S21.042A | Puncture wound with foreign body of left breast, initial encounter | | |
| S21.049A | Puncture wound with foreign body of unspecified breast, initial encounter | | |
| S21.051A | Open bite of right breast, initial encounter | | |
| S21.052A | Open bite of left breast, initial encounter | | |
| S21.059A | Open bite of unspecified breast, initial encounter | | |

© 2019 Vashi AA et al. *JAMA Network Open.*

| | | | |
|---|---|---|---|
| S21.101A | Unspecified open wound of right front wall of thorax without penetration into thoracic cavity, initial encounter | | |
| S21.102A | Unspecified open wound of left front wall of thorax without penetration into thoracic cavity, initial encounter | | |
| S21.109A | Unspecified open wound of unspecified front wall of thorax without penetration into thoracic cavity, initial encounter | | |
| S21.111A | Laceration without foreign body of right front wall of thorax without penetration into thoracic cavity, initial encounter | | |
| S21.112A | Laceration without foreign body of left front wall of thorax without penetration into thoracic cavity, initial encounter | | |
| S21.119A | Laceration without foreign body of unspecified front wall of thorax without penetration into thoracic cavity, initial encounter | | |
| S21.121A | Laceration with foreign body of right front wall of thorax without penetration into thoracic cavity, initial encounter | | |
| S21.122A | Laceration with foreign body of left front wall of thorax without penetration into thoracic cavity, initial encounter | | |
| S21.129A | Laceration with foreign body of unspecified front wall of thorax without penetration into thoracic cavity, initial encounter | | |
| S21.131A | Puncture wound without foreign body of right front wall of thorax without penetration into thoracic cavity, initial encounter | | |
| S21.132A | Puncture wound without foreign body of left front wall of thorax without penetration into thoracic cavity, initial encounter | | |
| S21.139A | Puncture wound without foreign body of unspecified front wall of thorax without penetration into thoracic cavity, initial encounter | | |
| S21.141A | Puncture wound with foreign body of right front wall of thorax without penetration into thoracic cavity, initial encounter | | |
| S21.142A | Puncture wound with foreign body of left front wall of thorax without penetration into thoracic cavity, initial encounter | | |
| S21.149A | Puncture wound with foreign body of unspecified front wall of thorax without penetration into thoracic cavity, initial encounter | | |

© 2019 Vashi AA et al. *JAMA Network Open.*

| | | | |
|---|---|---|---|
| S21.151A | Open bite of right front wall of thorax without penetration into thoracic cavity, initial encounter | | |
| S21.152A | Open bite of left front wall of thorax without penetration into thoracic cavity, initial encounter | | |
| S21.159A | Open bite of unspecified front wall of thorax without penetration into thoracic cavity, initial encounter | | |
| S21.201A | Unspecified open wound of right back wall of thorax without penetration into thoracic cavity, initial encounter | | |
| S21.202A | Unspecified open wound of left back wall of thorax without penetration into thoracic cavity, initial encounter | | |
| S21.209A | Unspecified open wound of unspecified back wall of thorax without penetration into thoracic cavity, initial encounter | | |
| S21.211A | Laceration without foreign body of right back wall of thorax without penetration into thoracic cavity, initial encounter | | |
| S21.212A | Laceration without foreign body of left back wall of thorax without penetration into thoracic cavity, initial encounter | | |
| S21.219A | Laceration without foreign body of unspecified back wall of thorax without penetration into thoracic cavity, initial encounter | | |
| S21.221A | Laceration with foreign body of right back wall of thorax without penetration into thoracic cavity, initial encounter | | |
| S21.222A | Laceration with foreign body of left back wall of thorax without penetration into thoracic cavity, initial encounter | | |
| S21.229A | Laceration with foreign body of unspecified back wall of thorax without penetration into thoracic cavity, initial encounter | | |
| S21.231A | Puncture wound without foreign body of right back wall of thorax without penetration into thoracic cavity, initial encounter | | |
| S21.232A | Puncture wound without foreign body of left back wall of thorax without penetration into thoracic cavity, initial encounter | | |
| S21.239A | Puncture wound without foreign body of unspecified back wall of thorax without penetration into thoracic cavity, initial encounter | | |

© 2019 Vashi AA et al. *JAMA Network Open.*

| | | | |
|---|---|---|---|
| S21.241A | Puncture wound with foreign body of right back wall of thorax without penetration into thoracic cavity, initial encounter | | |
| S21.242A | Puncture wound with foreign body of left back wall of thorax without penetration into thoracic cavity, initial encounter | | |
| S21.249A | Puncture wound with foreign body of unspecified back wall of thorax without penetration into thoracic cavity, initial encounter | | |
| S21.251A | Open bite of right back wall of thorax without penetration into thoracic cavity, initial encounter | | |
| S21.252A | Open bite of left back wall of thorax without penetration into thoracic cavity, initial encounter | | |
| S21.259A | Open bite of unspecified back wall of thorax without penetration into thoracic cavity, initial encounter | | |
| S21.301A | Unspecified open wound of right front wall of thorax with penetration into thoracic cavity, initial encounter | | |
| S21.302A | Unspecified open wound of left front wall of thorax with penetration into thoracic cavity, initial encounter | | |
| S21.309A | Unspecified open wound of unspecified front wall of thorax with penetration into thoracic cavity, initial encounter | | |
| S21.311A | Laceration without foreign body of right front wall of thorax with penetration into thoracic cavity, initial encounter | | |
| S21.312A | Laceration without foreign body of left front wall of thorax with penetration into thoracic cavity, initial encounter | | |
| S21.319A | Laceration without foreign body of unspecified front wall of thorax with penetration into thoracic cavity, initial encounter | | |
| S21.321A | Laceration with foreign body of right front wall of thorax with penetration into thoracic cavity, initial encounter | | |
| S21.322A | Laceration with foreign body of left front wall of thorax with penetration into thoracic cavity, initial encounter | | |
| S21.329A | Laceration with foreign body of unspecified front wall of thorax with penetration into thoracic cavity, initial encounter | | |

© 2019 Vashi AA et al. *JAMA Network Open.*

| | | | |
|---|---|---|---|
| S21.331A | Puncture wound without foreign body of right front wall of thorax with penetration into thoracic cavity, initial encounter | | |
| S21.332A | Puncture wound without foreign body of left front wall of thorax with penetration into thoracic cavity, initial encounter | | |
| S21.339A | Puncture wound without foreign body of unspecified front wall of thorax with penetration into thoracic cavity, initial encounter | | |
| S21.341A | Puncture wound with foreign body of right front wall of thorax with penetration into thoracic cavity, initial encounter | | |
| S21.342A | Puncture wound with foreign body of left front wall of thorax with penetration into thoracic cavity, initial encounter | | |
| S21.349A | Puncture wound with foreign body of unspecified front wall of thorax with penetration into thoracic cavity, initial encounter | | |
| S21.351A | Open bite of right front wall of thorax with penetration into thoracic cavity, initial encounter | | |
| S21.352A | Open bite of left front wall of thorax with penetration into thoracic cavity, initial encounter | | |
| S21.359A | Open bite of unspecified front wall of thorax with penetration into thoracic cavity, initial encounter | | |
| S21.401A | Unspecified open wound of right back wall of thorax with penetration into thoracic cavity, initial encounter | | |
| S21.402A | Unspecified open wound of left back wall of thorax with penetration into thoracic cavity, initial encounter | | |
| S21.409A | Unspecified open wound of unspecified back wall of thorax with penetration into thoracic cavity, initial encounter | | |
| S21.411A | Laceration without foreign body of right back wall of thorax with penetration into thoracic cavity, initial encounter | | |
| S21.412A | Laceration without foreign body of left back wall of thorax with penetration into thoracic cavity, initial encounter | | |
| S21.419A | Laceration without foreign body of unspecified back wall of thorax with penetration into thoracic cavity, initial encounter | | |

© 2019 Vashi AA et al. *JAMA Network Open.*

| | | | |
|---|---|---|---|
| S21.421A | Laceration with foreign body of right back wall of thorax with penetration into thoracic cavity, initial encounter | | |
| S21.422A | Laceration with foreign body of left back wall of thorax with penetration into thoracic cavity, initial encounter | | |
| S21.429A | Laceration with foreign body of unspecified back wall of thorax with penetration into thoracic cavity, initial encounter | | |
| S21.431A | Puncture wound without foreign body of right back wall of thorax with penetration into thoracic cavity, initial encounter | | |
| S21.432A | Puncture wound without foreign body of left back wall of thorax with penetration into thoracic cavity, initial encounter | | |
| S21.439A | Puncture wound without foreign body of unspecified back wall of thorax with penetration into thoracic cavity, initial encounter | | |
| S21.441A | Puncture wound with foreign body of right back wall of thorax with penetration into thoracic cavity, initial encounter | | |
| S21.442A | Puncture wound with foreign body of left back wall of thorax with penetration into thoracic cavity, initial encounter | | |
| S21.449A | Puncture wound with foreign body of unspecified back wall of thorax with penetration into thoracic cavity, initial encounter | | |
| S21.451A | Open bite of right back wall of thorax with penetration into thoracic cavity, initial encounter | | |
| S21.452A | Open bite of left back wall of thorax with penetration into thoracic cavity, initial encounter | | |
| S21.459A | Open bite of unspecified back wall of thorax with penetration into thoracic cavity, initial encounter | | |
| S21.90XA | Unspecified open wound of unspecified part of thorax, initial encounter | | |
| S21.91XA | Laceration without foreign body of unspecified part of thorax, initial encounter | | |
| S21.92XA | Laceration with foreign body of unspecified part of thorax, initial encounter | | |
| S21.93XA | Puncture wound without foreign body of unspecified part of thorax, initial encounter | | |

© 2019 Vashi AA et al. *JAMA Network Open.*

| | | | |
|---|---|---|---|
| S21.94XA | Puncture wound with foreign body of unspecified part of thorax, initial encounter | | |
| S21.95XA | Open bite of unspecified part of thorax, initial encounter | | |
| S22.000A | Wedge compression fracture of unspecified thoracic vertebra, initial encounter for closed fracture | | |
| S22.000B | Wedge compression fracture of unspecified thoracic vertebra, initial encounter for open fracture | | |
| S22.001A | Stable burst fracture of unspecified thoracic vertebra, initial encounter for closed fracture | | |
| S22.001B | Stable burst fracture of unspecified thoracic vertebra, initial encounter for open fracture | | |
| S22.002A | Unstable burst fracture of unspecified thoracic vertebra, initial encounter for closed fracture | | |
| S22.002B | Unstable burst fracture of unspecified thoracic vertebra, initial encounter for open fracture | | |
| S22.008A | Other fracture of unspecified thoracic vertebra, initial encounter for closed fracture | | |
| S22.008B | Other fracture of unspecified thoracic vertebra, initial encounter for open fracture | | |
| S22.009A | Unspecified fracture of unspecified thoracic vertebra, initial encounter for closed fracture | | |
| S22.009B | Unspecified fracture of unspecified thoracic vertebra, initial encounter for open fracture | | |
| S22.010A | Wedge compression fracture of first thoracic vertebra, initial encounter for closed fracture | | |
| S22.010B | Wedge compression fracture of first thoracic vertebra, initial encounter for open fracture | | |
| S22.011A | Stable burst fracture of first thoracic vertebra, initial encounter for closed fracture | | |
| S22.011B | Stable burst fracture of first thoracic vertebra, initial encounter for open fracture | | |
| S22.012A | Unstable burst fracture of first thoracic vertebra, initial encounter for closed fracture | | |
| S22.012B | Unstable burst fracture of first thoracic vertebra, initial encounter for open fracture | | |
| S22.018A | Other fracture of first thoracic vertebra, initial encounter for closed fracture | | |
| S22.018B | Other fracture of first thoracic vertebra, initial encounter for open fracture | | |

© 2019 Vashi AA et al. *JAMA Network Open.*

| | | | |
|---|---|---|---|
| S22.019A | Unspecified fracture of first thoracic vertebra, initial encounter for closed fracture | | |
| S22.019B | Unspecified fracture of first thoracic vertebra, initial encounter for open fracture | | |
| S22.020A | Wedge compression fracture of second thoracic vertebra, initial encounter for closed fracture | | |
| S22.020B | Wedge compression fracture of second thoracic vertebra, initial encounter for open fracture | | |
| S22.021A | Stable burst fracture of second thoracic vertebra, initial encounter for closed fracture | | |
| S22.021B | Stable burst fracture of second thoracic vertebra, initial encounter for open fracture | | |
| S22.022A | Unstable burst fracture of second thoracic vertebra, initial encounter for closed fracture | | |
| S22.022B | Unstable burst fracture of second thoracic vertebra, initial encounter for open fracture | | |
| S22.028A | Other fracture of second thoracic vertebra, initial encounter for closed fracture | | |
| S22.028B | Other fracture of second thoracic vertebra, initial encounter for open fracture | | |
| S22.029A | Unspecified fracture of second thoracic vertebra, initial encounter for closed fracture | | |
| S22.029B | Unspecified fracture of second thoracic vertebra, initial encounter for open fracture | | |
| S22.030A | Wedge compression fracture of third thoracic vertebra, initial encounter for closed fracture | | |
| S22.030B | Wedge compression fracture of third thoracic vertebra, initial encounter for open fracture | | |
| S22.031A | Stable burst fracture of third thoracic vertebra, initial encounter for closed fracture | | |
| S22.031B | Stable burst fracture of third thoracic vertebra, initial encounter for open fracture | | |
| S22.032A | Unstable burst fracture of third thoracic vertebra, initial encounter for closed fracture | | |
| S22.032B | Unstable burst fracture of third thoracic vertebra, initial encounter for open fracture | | |
| S22.038A | Other fracture of third thoracic vertebra, initial encounter for closed fracture | | |

© 2019 Vashi AA et al. *JAMA Network Open.*

| | | | |
|---|---|---|---|
| S22.038B | Other fracture of third thoracic vertebra, initial encounter for open fracture | | |
| S22.039A | Unspecified fracture of third thoracic vertebra, initial encounter for closed fracture | | |
| S22.039B | Unspecified fracture of third thoracic vertebra, initial encounter for open fracture | | |
| S22.040A | Wedge compression fracture of fourth thoracic vertebra, initial encounter for closed fracture | | |
| S22.040B | Wedge compression fracture of fourth thoracic vertebra, initial encounter for open fracture | | |
| S22.041A | Stable burst fracture of fourth thoracic vertebra, initial encounter for closed fracture | | |
| S22.041B | Stable burst fracture of fourth thoracic vertebra, initial encounter for open fracture | | |
| S22.042A | Unstable burst fracture of fourth thoracic vertebra, initial encounter for closed fracture | | |
| S22.042B | Unstable burst fracture of fourth thoracic vertebra, initial encounter for open fracture | | |
| S22.048A | Other fracture of fourth thoracic vertebra, initial encounter for closed fracture | | |
| S22.048B | Other fracture of fourth thoracic vertebra, initial encounter for open fracture | | |
| S22.049A | Unspecified fracture of fourth thoracic vertebra, initial encounter for closed fracture | | |
| S22.049B | Unspecified fracture of fourth thoracic vertebra, initial encounter for open fracture | | |
| S22.050A | Wedge compression fracture of T5-T6 vertebra, initial encounter for closed fracture | | |
| S22.050B | Wedge compression fracture of T5-T6 vertebra, initial encounter for open fracture | | |
| S22.051A | Stable burst fracture of T5-T6 vertebra, initial encounter for closed fracture | | |
| S22.051B | Stable burst fracture of T5-T6 vertebra, initial encounter for open fracture | | |
| S22.052A | Unstable burst fracture of T5-T6 vertebra, initial encounter for closed fracture | | |
| S22.052B | Unstable burst fracture of T5-T6 vertebra, initial encounter for open fracture | | |

© 2019 Vashi AA et al. *JAMA Network Open.*

| | | | |
|---|---|---|---|
| S22.058A | Other fracture of T5-T6 vertebra, initial encounter for closed fracture | | |
| S22.058B | Other fracture of T5-T6 vertebra, initial encounter for open fracture | | |
| S22.059A | Unspecified fracture of T5-T6 vertebra, initial encounter for closed fracture | | |
| S22.059B | Unspecified fracture of T5-T6 vertebra, initial encounter for open fracture | | |
| S22.060A | Wedge compression fracture of T7-T8 vertebra, initial encounter for closed fracture | | |
| S22.060B | Wedge compression fracture of T7-T8 vertebra, initial encounter for open fracture | | |
| S22.061A | Stable burst fracture of T7-T8 vertebra, initial encounter for closed fracture | | |
| S22.061B | Stable burst fracture of T7-T8 vertebra, initial encounter for open fracture | | |
| S22.062A | Unstable burst fracture of T7-T8 vertebra, initial encounter for closed fracture | | |
| S22.062B | Unstable burst fracture of T7-T8 vertebra, initial encounter for open fracture | | |
| S22.068A | Other fracture of T7-T8 thoracic vertebra, initial encounter for closed fracture | | |
| S22.068B | Other fracture of T7-T8 thoracic vertebra, initial encounter for open fracture | | |
| S22.069A | Unspecified fracture of T7-T8 vertebra, initial encounter for closed fracture | | |
| S22.069B | Unspecified fracture of T7-T8 vertebra, initial encounter for open fracture | | |
| S22.070A | Wedge compression fracture of T9-T10 vertebra, initial encounter for closed fracture | | |
| S22.070B | Wedge compression fracture of T9-T10 vertebra, initial encounter for open fracture | | |
| S22.071A | Stable burst fracture of T9-T10 vertebra, initial encounter for closed fracture | | |
| S22.071B | Stable burst fracture of T9-T10 vertebra, initial encounter for open fracture | | |
| S22.072A | Unstable burst fracture of T9-T10 vertebra, initial encounter for closed fracture | | |

© 2019 Vashi AA et al. *JAMA Network Open.*

| | | | |
|---|---|---|---|
| S22.072B | Unstable burst fracture of T9-T10 vertebra, initial encounter for open fracture | | |
| S22.078A | Other fracture of T9-T10 vertebra, initial encounter for closed fracture | | |
| S22.078B | Other fracture of T9-T10 vertebra, initial encounter for open fracture | | |
| S22.079A | Unspecified fracture of T9-T10 vertebra, initial encounter for closed fracture | | |
| S22.079B | Unspecified fracture of T9-T10 vertebra, initial encounter for open fracture | | |
| S22.080A | Wedge compression fracture of T11-T12 vertebra, initial encounter for closed fracture | | |
| S22.080B | Wedge compression fracture of T11-T12 vertebra, initial encounter for open fracture | | |
| S22.081A | Stable burst fracture of T11-T12 vertebra, initial encounter for closed fracture | | |
| S22.081B | Stable burst fracture of T11-T12 vertebra, initial encounter for open fracture | | |
| S22.082A | Unstable burst fracture of T11-T12 vertebra, initial encounter for closed fracture | | |
| S22.082B | Unstable burst fracture of T11-T12 vertebra, initial encounter for open fracture | | |
| S22.088A | Other fracture of T11-T12 vertebra, initial encounter for closed fracture | | |
| S22.088B | Other fracture of T11-T12 vertebra, initial encounter for open fracture | | |
| S22.089A | Unspecified fracture of T11-T12 vertebra, initial encounter for closed fracture | | |
| S22.089B | Unspecified fracture of T11-T12 vertebra, initial encounter for open fracture | | |
| S22.20XA | Unspecified fracture of sternum, initial encounter for closed fracture | | |
| S22.20XB | Unspecified fracture of sternum, initial encounter for open fracture | | |
| S22.21XA | Fracture of manubrium, initial encounter for closed fracture | | |
| S22.21XB | Fracture of manubrium, initial encounter for open fracture | | |
| S22.22XA | Fracture of body of sternum, initial encounter for closed fracture | | |

© 2019 Vashi AA et al. *JAMA Network Open.*

| | | | |
|---|---|---|---|
| S22.22XB | Fracture of body of sternum, initial encounter for open fracture | | |
| S22.23XA | Sternal manubrial dissociation, initial encounter for closed fracture | | |
| S22.23XB | Sternal manubrial dissociation, initial encounter for open fracture | | |
| S22.24XA | Fracture of xiphoid process, initial encounter for closed fracture | | |
| S22.24XB | Fracture of xiphoid process, initial encounter for open fracture | | |
| S22.41XA | Multiple fractures of ribs, right side, initial encounter for closed fracture | | |
| S22.41XB | Multiple fractures of ribs, right side, initial encounter for open fracture | | |
| S22.42XA | Multiple fractures of ribs, left side, initial encounter for closed fracture | | |
| S22.42XB | Multiple fractures of ribs, left side, initial encounter for open fracture | | |
| S22.43XA | Multiple fractures of ribs, bilateral, initial encounter for closed fracture | | |
| S22.43XB | Multiple fractures of ribs, bilateral, initial encounter for open fracture | | |
| S22.49XA | Multiple fractures of ribs, unspecified side, initial encounter for closed fracture | | |
| S22.49XB | Multiple fractures of ribs, unspecified side, initial encounter for open fracture | | |
| S22.5XXA | Flail chest, initial encounter for closed fracture | | |
| S22.5XXB | Flail chest, initial encounter for open fracture | | |
| S22.9XXA | Fracture of bony thorax, part unspecified, initial encounter for closed fracture | | |
| S22.9XXB | Fracture of bony thorax, part unspecified, initial encounter for open fracture | | |
| S24.0XXA | Concussion and edema of thoracic spinal cord, initial encounter | | |
| S24.101A | Unspecified injury at T1 level of thoracic spinal cord, initial encounter | | |
| S24.102A | Unspecified injury at T2-T6 level of thoracic spinal cord, initial encounter | | |

© 2019 Vashi AA et al. *JAMA Network Open.*

| | | | |
|---|---|---|---|
| S24.103A | Unspecified injury at T7-T10 level of thoracic spinal cord, initial encounter | | |
| S24.104A | Unspecified injury at T11-T12 level of thoracic spinal cord, initial encounter | | |
| S24.109A | Unspecified injury at unspecified level of thoracic spinal cord, initial encounter | | |
| S24.111A | Complete lesion at T1 level of thoracic spinal cord, initial encounter | | |
| S24.112A | Complete lesion at T2-T6 level of thoracic spinal cord, initial encounter | | |
| S24.113A | Complete lesion at T7-T10 level of thoracic spinal cord, initial encounter | | |
| S24.114A | Complete lesion at T11-T12 level of thoracic spinal cord, initial encounter | | |
| S24.119A | Complete lesion at unspecified level of thoracic spinal cord, initial encounter | | |
| S24.131A | Anterior cord syndrome at T1 level of thoracic spinal cord, initial encounter | | |
| S24.132A | Anterior cord syndrome at T2-T6 level of thoracic spinal cord, initial encounter | | |
| S24.133A | Anterior cord syndrome at T7-T10 level of thoracic spinal cord, initial encounter | | |
| S24.134A | Anterior cord syndrome at T11-T12 level of thoracic spinal cord, initial encounter | | |
| S24.139A | Anterior cord syndrome at unspecified level of thoracic spinal cord, initial encounter | | |
| S24.141A | Brown-Sequard syndrome at T1 level of thoracic spinal cord, initial encounter | | |
| S24.142A | Brown-Sequard syndrome at T2-T6 level of thoracic spinal cord, initial encounter | | |
| S24.143A | Brown-Sequard syndrome at T7-T10 level of thoracic spinal cord, initial encounter | | |
| S24.144A | Brown-Sequard syndrome at T11-T12 level of thoracic spinal cord, initial encounter | | |
| S24.149A | Brown-Sequard syndrome at unspecified level of thoracic spinal cord, initial encounter | | |
| S24.151A | Other incomplete lesion at T1 level of thoracic spinal cord, initial encounter | | |

© 2019 Vashi AA et al. *JAMA Network Open.*

| | | | |
|---|---|---|---|
| S24.152A | Other incomplete lesion at T2-T6 level of thoracic spinal cord, initial encounter | | |
| S24.153A | Other incomplete lesion at T7-T10 level of thoracic spinal cord, initial encounter | | |
| S24.154A | Other incomplete lesion at T11-T12 level of thoracic spinal cord, initial encounter | | |
| S24.159A | Other incomplete lesion at unspecified level of thoracic spinal cord, initial encounter | | |
| S24.2XXA | Injury of nerve root of thoracic spine, initial encounter | | |
| S24.3XXA | Injury of peripheral nerves of thorax, initial encounter | | |
| S24.4XXA | Injury of thoracic sympathetic nervous system, initial encounter | | |
| S24.8XXA | Injury of other specified nerves of thorax, initial encounter | | |
| S24.9XXA | Injury of unspecified nerve of thorax, initial encounter | | |
| S25.00XA | Unspecified injury of thoracic aorta, initial encounter | | |
| S25.01XA | Minor laceration of thoracic aorta, initial encounter | | |
| S25.02XA | Major laceration of thoracic aorta, initial encounter | | |
| S25.09XA | Other specified injury of thoracic aorta, initial encounter | | |
| S25.101A | Unspecified injury of right innominate or subclavian artery, initial encounter | | |
| S25.102A | Unspecified injury of left innominate or subclavian artery, initial encounter | | |
| S25.109A | Unspecified injury of unspecified innominate or subclavian artery, initial encounter | | |
| S25.111A | Minor laceration of right innominate or subclavian artery, initial encounter | | |
| S25.112A | Minor laceration of left innominate or subclavian artery, initial encounter | | |
| S25.119A | Minor laceration of unspecified innominate or subclavian artery, initial encounter | | |
| S25.121A | Major laceration of right innominate or subclavian artery, initial encounter | | |
| S25.122A | Major laceration of left innominate or subclavian artery, initial encounter | | |
| S25.129A | Major laceration of unspecified innominate or subclavian artery, initial encounter | | |
| S25.191A | Other specified injury of right innominate or subclavian artery, initial encounter | | |

© 2019 Vashi AA et al. *JAMA Network Open.*

| | | | |
|---|---|---|---|
| S25.192A | Other specified injury of left innominate or subclavian artery, initial encounter | | |
| S25.199A | Other specified injury of unspecified innominate or subclavian artery, initial encounter | | |
| S25.20XA | Unspecified injury of superior vena cava, initial encounter | | |
| S25.21XA | Minor laceration of superior vena cava, initial encounter | | |
| S25.22XA | Major laceration of superior vena cava, initial encounter | | |
| S25.29XA | Other specified injury of superior vena cava, initial encounter | | |
| S25.301A | Unspecified injury of right innominate or subclavian vein, initial encounter | | |
| S25.302A | Unspecified injury of left innominate or subclavian vein, initial encounter | | |
| S25.309A | Unspecified injury of unspecified innominate or subclavian vein, initial encounter | | |
| S25.311A | Minor laceration of right innominate or subclavian vein, initial encounter | | |
| S25.312A | Minor laceration of left innominate or subclavian vein, initial encounter | | |
| S25.319A | Minor laceration of unspecified innominate or subclavian vein, initial encounter | | |
| S25.321A | Major laceration of right innominate or subclavian vein, initial encounter | | |
| S25.322A | Major laceration of left innominate or subclavian vein, initial encounter | | |
| S25.329A | Major laceration of unspecified innominate or subclavian vein, initial encounter | | |
| S25.391A | Other specified injury of right innominate or subclavian vein, initial encounter | | |
| S25.392A | Other specified injury of left innominate or subclavian vein, initial encounter | | |
| S25.399A | Other specified injury of unspecified innominate or subclavian vein, initial encounter | | |
| S25.401A | Unspecified injury of right pulmonary blood vessels, initial encounter | | |
| S25.402A | Unspecified injury of left pulmonary blood vessels, initial encounter | | |
| S25.409A | Unspecified injury of unspecified pulmonary blood vessels, initial encounter | | |

© 2019 Vashi AA et al. *JAMA Network Open.*

| | | | |
|---|---|---|---|
| S25.411A | Minor laceration of right pulmonary blood vessels, initial encounter | | |
| S25.412A | Minor laceration of left pulmonary blood vessels, initial encounter | | |
| S25.419A | Minor laceration of unspecified pulmonary blood vessels, initial encounter | | |
| S25.421A | Major laceration of right pulmonary blood vessels, initial encounter | | |
| S25.422A | Major laceration of left pulmonary blood vessels, initial encounter | | |
| S25.429A | Major laceration of unspecified pulmonary blood vessels, initial encounter | | |
| S25.491A | Other specified injury of right pulmonary blood vessels, initial encounter | | |
| S25.492A | Other specified injury of left pulmonary blood vessels, initial encounter | | |
| S25.499A | Other specified injury of unspecified pulmonary blood vessels, initial encounter | | |
| S25.501A | Unspecified injury of intercostal blood vessels, right side, initial encounter | | |
| S25.502A | Unspecified injury of intercostal blood vessels, left side, initial encounter | | |
| S25.509A | Unspecified injury of intercostal blood vessels, unspecified side, initial encounter | | |
| S25.511A | Laceration of intercostal blood vessels, right side, initial encounter | | |
| S25.512A | Laceration of intercostal blood vessels, left side, initial encounter | | |
| S25.519A | Laceration of intercostal blood vessels, unspecified side, initial encounter | | |
| S25.591A | Other specified injury of intercostal blood vessels, right side, initial encounter | | |
| S25.592A | Other specified injury of intercostal blood vessels, left side, initial encounter | | |
| S25.599A | Other specified injury of intercostal blood vessels, unspecified side, initial encounter | | |
| S25.801A | Unspecified injury of other blood vessels of thorax, right side, initial encounter | | |

© 2019 Vashi AA et al. *JAMA Network Open.*

| | | | |
|---|---|---|---|
| S25.802A | Unspecified injury of other blood vessels of thorax, left side, initial encounter | | |
| S25.809A | Unspecified injury of other blood vessels of thorax, unspecified side, initial encounter | | |
| S25.811A | Laceration of other blood vessels of thorax, right side, initial encounter | | |
| S25.812A | Laceration of other blood vessels of thorax, left side, initial encounter | | |
| S25.819A | Laceration of other blood vessels of thorax, unspecified side, initial encounter | | |
| S25.891A | Other specified injury of other blood vessels of thorax, right side, initial encounter | | |
| S25.892A | Other specified injury of other blood vessels of thorax, left side, initial encounter | | |
| S25.899A | Other specified injury of other blood vessels of thorax, unspecified side, initial encounter | | |
| S25.90XA | Unspecified injury of unspecified blood vessel of thorax, initial encounter | | |
| S25.91XA | Laceration of unspecified blood vessel of thorax, initial encounter | | |
| S25.99XA | Other specified injury of unspecified blood vessel of thorax, initial encounter | | |
| S26.00XA | Unspecified injury of heart with hemopericardium, initial encounter | | |
| S26.01XA | Contusion of heart with hemopericardium, initial encounter | | |
| S26.020A | Mild laceration of heart with hemopericardium, initial encounter | | |
| S26.021A | Moderate laceration of heart with hemopericardium, initial encounter | | |
| S26.022A | Major laceration of heart with hemopericardium, initial encounter | | |
| S26.09XA | Other injury of heart with hemopericardium, initial encounter | | |
| S26.10XA | Unspecified injury of heart without hemopericardium, initial encounter | | |
| S26.11XA | Contusion of heart without hemopericardium, initial encounter | | |

© 2019 Vashi AA et al. *JAMA Network Open.*

| | | | |
|---|---|---|---|
| S26.12XA | Laceration of heart without hemopericardium, initial encounter | | |
| S26.19XA | Other injury of heart without hemopericardium, initial encounter | | |
| S26.90XA | Unspecified injury of heart, unspecified with or without hemopericardium, initial encounter | | |
| S26.91XA | Contusion of heart, unspecified with or without hemopericardium, initial encounter | | |
| S26.92XA | Laceration of heart, unspecified with or without hemopericardium, initial encounter | | |
| S26.99XA | Other injury of heart, unspecified with or without hemopericardium, initial encounter | | |
| S27.0XXA | Traumatic pneumothorax, initial encounter | | |
| S27.1XXA | Traumatic hemothorax, initial encounter | | |
| S27.2XXA | Traumatic hemopneumothorax, initial encounter | | |
| S27.301A | Unspecified injury of lung, unilateral, initial encounter | | |
| S27.302A | Unspecified injury of lung, bilateral, initial encounter | | |
| S27.309A | Unspecified injury of lung, unspecified, initial encounter | | |
| S27.311A | Primary blast injury of lung, unilateral, initial encounter | | |
| S27.312A | Primary blast injury of lung, bilateral, initial encounter | | |
| S27.319A | Primary blast injury of lung, unspecified, initial encounter | | |
| S27.321A | Contusion of lung, unilateral, initial encounter | | |
| S27.322A | Contusion of lung, bilateral, initial encounter | | |
| S27.329A | Contusion of lung, unspecified, initial encounter | | |
| S27.331A | Laceration of lung, unilateral, initial encounter | | |
| S27.332A | Laceration of lung, bilateral, initial encounter | | |
| S27.339A | Laceration of lung, unspecified, initial encounter | | |
| S27.391A | Other injuries of lung, unilateral, initial encounter | | |
| S27.392A | Other injuries of lung, bilateral, initial encounter | | |
| S27.399A | Other injuries of lung, unspecified, initial encounter | | |
| S27.401A | Unspecified injury of bronchus, unilateral, initial encounter | | |
| S27.402A | Unspecified injury of bronchus, bilateral, initial encounter | | |
| S27.409A | Unspecified injury of bronchus, unspecified, initial encounter | | |
| S27.411A | Primary blast injury of bronchus, unilateral, initial encounter | | |

© 2019 Vashi AA et al. *JAMA Network Open.*

| | | | |
|---|---|---|---|
| S27.412A | Primary blast injury of bronchus, bilateral, initial encounter | | |
| S27.419A | Primary blast injury of bronchus, unspecified, initial encounter | | |
| S27.421A | Contusion of bronchus, unilateral, initial encounter | | |
| S27.422A | Contusion of bronchus, bilateral, initial encounter | | |
| S27.429A | Contusion of bronchus, unspecified, initial encounter | | |
| S27.431A | Laceration of bronchus, unilateral, initial encounter | | |
| S27.432A | Laceration of bronchus, bilateral, initial encounter | | |
| S27.439A | Laceration of bronchus, unspecified, initial encounter | | |
| S27.491A | Other injury of bronchus, unilateral, initial encounter | | |
| S27.492A | Other injury of bronchus, bilateral, initial encounter | | |
| S27.499A | Other injury of bronchus, unspecified, initial encounter | | |
| S27.50XA | Unspecified injury of thoracic trachea, initial encounter | | |
| S27.51XA | Primary blast injury of thoracic trachea, initial encounter | | |
| S27.52XA | Contusion of thoracic trachea, initial encounter | | |
| S27.53XA | Laceration of thoracic trachea, initial encounter | | |
| S27.59XA | Other injury of thoracic trachea, initial encounter | | |
| S27.60XA | Unspecified injury of pleura, initial encounter | | |
| S27.63XA | Laceration of pleura, initial encounter | | |
| S27.69XA | Other injury of pleura, initial encounter | | |
| S27.802A | Contusion of diaphragm, initial encounter | | |
| S27.803A | Laceration of diaphragm, initial encounter | | |
| S27.808A | Other injury of diaphragm, initial encounter | | |
| S27.809A | Unspecified injury of diaphragm, initial encounter | | |
| S27.812A | Contusion of esophagus (thoracic part), initial encounter | | |
| S27.813A | Laceration of esophagus (thoracic part), initial encounter | | |
| S27.818A | Other injury of esophagus (thoracic part), initial encounter | | |
| S27.819A | Unspecified injury of esophagus (thoracic part), initial encounter | | |
| S27.892A | Contusion of other specified intrathoracic organs, initial encounter | | |
| S27.893A | Laceration of other specified intrathoracic organs, initial encounter | | |

© 2019 Vashi AA et al. *JAMA Network Open.*

| | | | |
|---|---|---|---|
| S27.898A | Other injury of other specified intrathoracic organs, initial encounter | | |
| S27.899A | Unspecified injury of other specified intrathoracic organs, initial encounter | | |
| S27.9XXA | Injury of unspecified intrathoracic organ, initial encounter | | |
| S28.0XXA | Crushed chest, initial encounter | | |
| S28.1XXA | Traumatic amputation (partial) of part of thorax, except breast, initial encounter | | |
| S28.211A | Complete traumatic amputation of right breast, initial encounter | | |
| S28.212A | Complete traumatic amputation of left breast, initial encounter | | |
| S28.219A | Complete traumatic amputation of unspecified breast, initial encounter | | |
| S28.221A | Partial traumatic amputation of right breast, initial encounter | | |
| S28.222A | Partial traumatic amputation of left breast, initial encounter | | |
| S28.229A | Partial traumatic amputation of unspecified breast, initial encounter | | |
| S29.001A | Unspecified injury of muscle and tendon of front wall of thorax, initial encounter | | |
| S29.002A | Unspecified injury of muscle and tendon of back wall of thorax, initial encounter | | |
| S29.009A | Unspecified injury of muscle and tendon of unspecified wall of thorax, initial encounter | | |
| S29.021A | Laceration of muscle and tendon of front wall of thorax, initial encounter | | |
| S29.022A | Laceration of muscle and tendon of back wall of thorax, initial encounter | | |
| S29.029A | Laceration of muscle and tendon of unspecified wall of thorax, initial encounter | | |
| S29.091A | Other injury of muscle and tendon of front wall of thorax, initial encounter | | |
| S29.092A | Other injury of muscle and tendon of back wall of thorax, initial encounter | | |
| S29.099A | Other injury of muscle and tendon of unspecified wall of thorax, initial encounter | | |
| S29.8XXA | Other specified injuries of thorax, initial encounter | | |

© 2019 Vashi AA et al. *JAMA Network Open.*

| | | | |
|---|---|---|---|
| S29.9XXA | Unspecified injury of thorax, initial encounter | | |

## Included and Excluded ICD-10-CM Diagnosis Subcodes for Volume Depletion Condition Group

| Included ICD-10-CM diagnosis subcodes (n=3) | | Excluded ICD-10-CM diagnosis subcodes (n=0) | |
|---|---|---|---|
| **Subcode** | **Subcode description** | **Subcode** | **Subcode description** |
| E86.0 | Dehydration | | |
| E86.1 | Hypovolemia | | |
| E86.9 | Volume depletion, unspecified | | |

© 2019 Vashi AA et al. *JAMA Network Open.*

**eTable 1.** Characteristics of the Panelists

| **Characteristic** | **Panelists (n=8)** |
|---|---|
| Male, n (%) | 5 (63) |
| Years since last training program, mean (SD) | 15.8 (9.3) |
| Expertise | |
| Primary Care | 1 |
| Hospitalist | 4 |
| Emergency Medicine | 3 |
| Practice location by US Census region | |
| Northeast | 1 |
| South | 1 |
| Midwest | 2 |
| West | 4 |
| Nominating professional association | |
| ACEP | 1 |
| SGIM | 3 |
| SHM | 2 |

Abbreviations: ACEP, American College of Emergency Physicians; SGIM, Society of General Internal Medicine; SHM, Society of Hospital Medicine

© 2019 Vashi AA et al. *JAMA Network Open.*

**eTable 2.** Most Common Conditions Among Non-ECSC Visits, 2016

| | **Non-ECSC ED visits N= 98,289,685[a]** |
|---|---|
| **CCS category name** | **n (%)** |
| 251 Abdominal pain | 5,255,926 (5.3) |
| 102 Nonspecific chest pain | 5,082,227 (5.2) |
| 232 Sprains and strains | 4,376,179 (4.5) |
| 205 Spondylosis; intervertebral disc disorders; other back problems | 4,203,711 (4.3) |
| 239 Superficial injury; contusion | 3,928,481 (4.0) |
| 159 Urinary tract infections | 3,238,549 (3.3) |
| 126 Other upper respiratory infections | 2,953,253 (3.0) |
| 84 Headache; including migraine | 2,892,901 (2.9) |
| 197 Skin and subcutaneous tissue infections | 2,856,414 (2.9) |
| 236 Open wounds of extremities | 2,521,996 (2.6) |
| 211 Other connective tissue disease | 2,331,981 (2.4) |
| 244 Other injuries and conditions due to external causes | 2,045,524 (2.1) |
| 204 Other non-traumatic joint disorders | 2,030,368 (2.1) |
| 181 Other complications of pregnancy | 1,943,594 (2.0) |
| 136 Disorders of teeth and jaw | 1,773,408 (1.8) |
| 133 Other lower respiratory disease | 1,498,586 (1.5) |
| 95 Other nervous system disorders | 1,422,926 (1.4) |
| 250 Nausea and vomiting | 1,410,090 (1.4) |
| 155 Other gastrointestinal disorders | 1,379,411 (1.4) |
| 660 Alcohol-related disorders | 1,307,836 (1.3) |
| 93 Conditions associated with dizziness or vertigo | 1,274,965 (1.3) |
| 259 Residual codes; unclassified | 1,197,021 (1.2) |
| 229 Fracture of upper limb | 1,155,798 (1.2) |
| 235 Open wounds of head; neck; and trunk | 1,147,195 (1.2) |
| 245 Syncope | 1,127,832 (1.1) |
| 125 Acute bronchitis | 1,017,299 (1.0) |
| 253 Allergic reactions | 1,010,908 (1.0) |
| 651 Anxiety disorders | 998,806 (1.0) |
| 98 Essential hypertension | 955,243 (1.0) |
| 160 Calculus of urinary tract | 954,898 (1.0) |
| 657 Mood disorders | 950,667 (1.0) |
| 154 Noninfectious gastroenteritis | 918,891 (0.9) |
| 83 Epilepsy; convulsions | 854,479 (0.9) |
| 230 Fracture of lower limb | 812,726 (0.8) |
| 163 Genitourinary symptoms and ill-defined conditions | 807,672 (0.8) |
| 7 Viral infection | 791,951 (0.8) |
| 200 Other skin disorders | 777,434 (0.8) |

© 2019 Vashi AA et al. *JAMA Network Open.*

| | |
|---|---|
| 106 Cardiac dysrhythmias | 732,876 (0.7) |
| 127 Chronic obstructive pulmonary disease and bronchiectasis | 697,562 (0.7) |
| 149 Biliary tract disease | 669,777 (0.7) |
| 661 Substance-related disorders | 659,807 (0.7) |
| 134 Other upper respiratory disease | 644,894 (0.7) |
| 182 Hemorrhage during pregnancy; abruptio placenta; placenta previa | 644,615 (0.7) |
| 252 Malaise and fatigue | 597,221 (0.6) |
| 659 Schizophrenia and other psychotic disorders | 574,624 (0.6) |
| 146 Diverticulosis and diverticulitis | 550,902 (0.6) |
| 99 Hypertension with complications and secondary hypertension | 540,923 (0.6) |
| 157 Acute and unspecified renal failure | 528,804 (0.5) |
| 90 Inflammation; infection of eye (except that caused by tuberculosis or sexually transmitted disease) | 527,184 (0.5) |
| 175 Other female genital disorders | 518,139 (0.5) |

[a]The table lists only the top 50 most common CCS categories for the non-ECSC visits. 19,153,714 (19.5%) non-ECSC visits were classified to CCS categories that are not listed. 41,500 (0.0%) non-ECSC visits were missing a CCS category.

© 2019 Vashi AA et al. *JAMA Network Open.*
